# Supplementary material for: Estimating the power of sequence covariation for detecting conserved RNA structure
Source: Bioinformatics. 2020 Feb 7;36(10):3072–6. doi: 10.1093/bioinformatics/btaa080 (PMC7214042; doi:10.1093/bioinformatics/btaa080)
Supplement: btaa080_Supplementary_Data [file btaa080_supplementary_data.zip › btaa080-Suppl_Data/Figures/FigureS1S2.pdf]

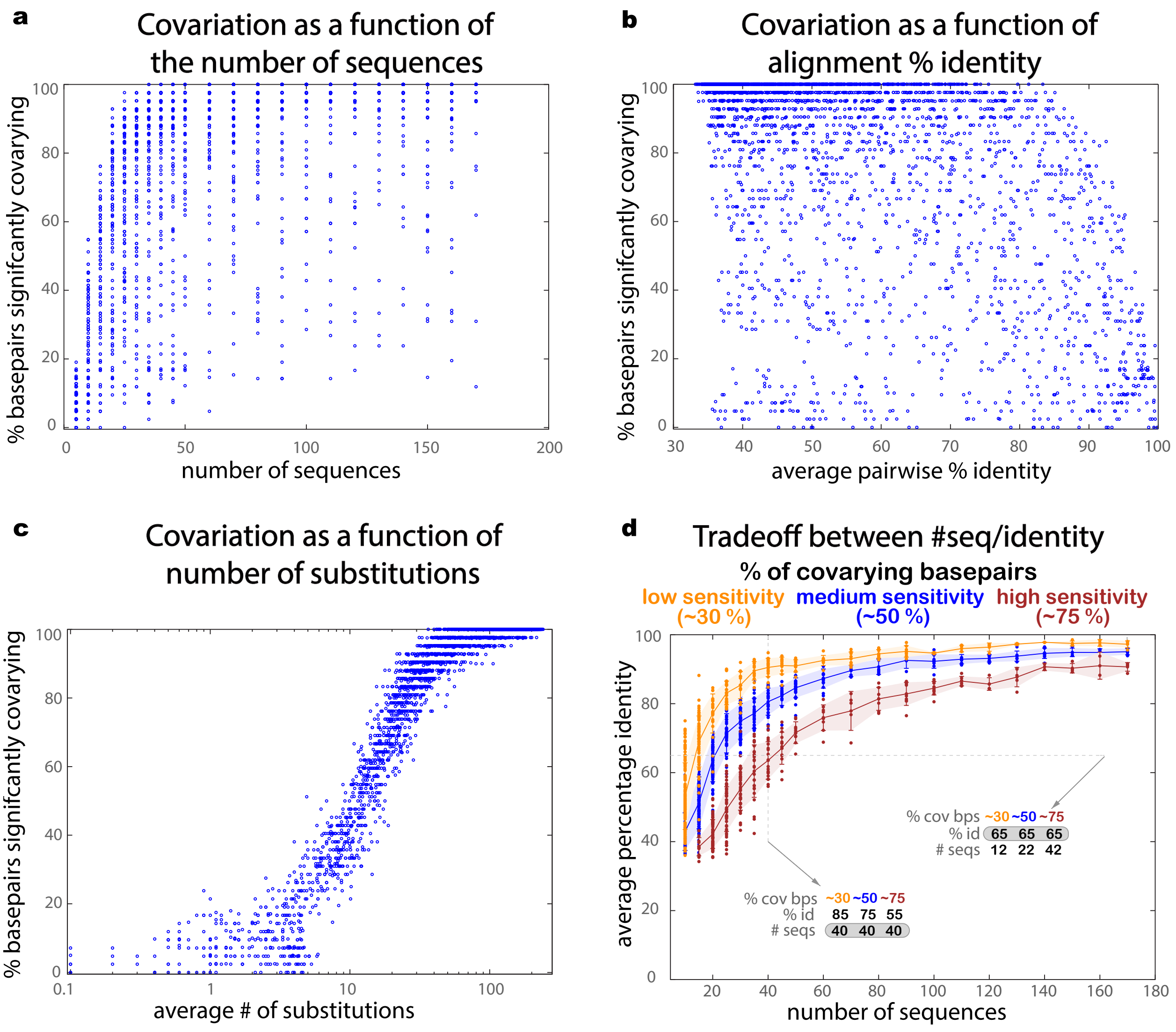
a
Covariation as a function of the number of sequences
% basepairs signifcantly covarying
number of sequences
b
Covariation as a function of alignment % identity
% basepairs signifcantly covarying
average pairwise % identity
c
Covariation as a function of number of substitutions
% basepairs signifcantly covarying
average # of substitutions
d
Tradeoff between #seq/identity
% of covarying basepairs
low sensitivity (~30 %)
medium sensitivity (~50 %)
high sensitivity (~75 %)
average percentage identity
number of sequences
% cov bps ~30 ~50 ~75
% id 65 65 65
# seqs 12 22 42
% cov bps ~30 ~50 ~75
% id 85 75 55
# seqs 40 40 40

Figure S1

Figure S1. **Correspondence between sequence variability and covariation on RNA structural simulated alignments produced using simulated phylogenetic trees.** Each point is a simulated alignment derived from the Cobalamin riboswitch Rfam seed alignment (RF00174) with 42 annotated basepairs. For each simulated alignment, we show the fraction of basepairs that covary with E-value < 0.05 (observed sensitivity) as a function of: **(b)** the number of sequences in the alignment; **(c)** the average percentage identity in the alignment; **(d)** the alignment average substitutions per basepair. **(e)** This panel shows the number of sequences and the average percentage identity required to achieve a low (yellow $\sim 27 - 32\%$), medium (blue $\sim 47 - 52\%$) or high (red $\sim 74 - 76\%$) covariation sensitivity level.

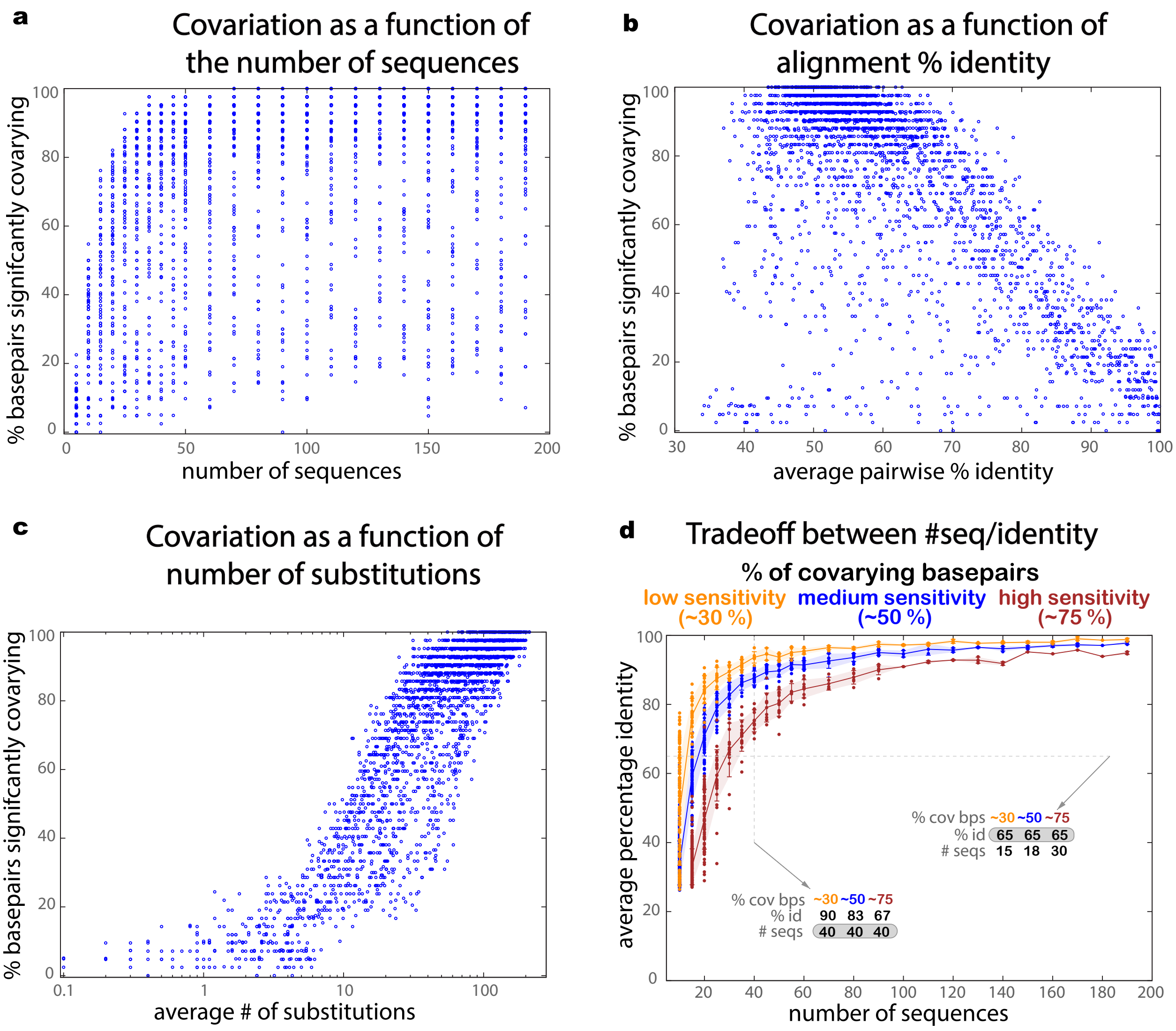
a
Covariation as a function of the number of sequences
% basepairs signifcantly covarying
number of sequences
b
Covariation as a function of alignment % identity
% basepairs signifcantly covarying
average pairwise % identity
c
Covariation as a function of number of substitutions
% basepairs signifcantly covarying
average # of substitutions
d
Tradeoff between #seq/identity
% of covarying basepairs
low sensitivity (~30 %)
medium sensitivity (~50 %)
high sensitivity (~75 %)
average percentage identity
number of sequences
% cov bps ~30 ~50 ~75
% id 65 65 65
# seqs 15 18 30
% cov bps ~30 ~50 ~75
% id 90 83 67
# seqs 40 40 40

Figure S2

Figure S2. **Correspondence between sequence variability and covariation on RNA structural simulated alignments, assuming all sequences are independent from each other.** Each point is a simulated alignment derived from the Cobalamin riboswitch Rfam seed alignment (RF00174) with 42 annotated basepairs. For each simulated alignment, we show the fraction of basepairs that covary with E-value < 0.05 (observed sensitivity) as a function of: **(b)** the number of sequences in the alignment; **(c)** the average percentage identity in the alignment; **(d)** the alignment average substitutions per basepair. **(e)** This panel shows the number of sequences and the average percentage identity required to achieve a low (yellow $\sim 27-32\%$), medium (blue $\sim 47-52\%$) or high (red $\sim 74-76\%$) covariation sensitivity level.
