# Supplementary material for: Estimating the power of sequence covariation for detecting conserved RNA structure
Source: Bioinformatics. 2020 Feb 7;36(10):3072–6. doi: 10.1093/bioinformatics/btaa080 (PMC7214042; doi:10.1093/bioinformatics/btaa080)
Supplement: btaa080_Supplementary_Data [file btaa080_supplementary_data.zip › btaa080-Suppl_Data/Tables/Table_S1.pdf]

1,411 RNA families with power, 1,031 with structural covariations

Table S1. Power of covariation for the complete Rfam v14.1 3,016 RNA families. The families are ordered by decreasing number of covariations and decreasing power. "bps" refers to basepairs annotated in the Rfam alignment; "pairs" refers to pairs annotated by R-scape as significant (E-value < 0.05). We used Rfam v14.1 seed alignments.

	RNA family (seed alignment)	**Sensitivity** annotated bpairs that covary % (cov_bps/bps)	**Power** average power %	**Positive Predictive Value** covarying pairs in structure % (cov_bps/cov_pairs)	average substitutions per bpair	avg pairwise identity %	number of sequences
1	RF00005 tRNA	100.0 (21/21)	100.0	56.8 (21/37)	1299.7	44.4	954
2	RF01717 PhotoRC-II	100.0 (13/13)	99.2	92.8 (13/14)	482.2	61.8	445
3	RF00026 U6	100.0 (5/5)	96.0	100.0 (5/5)	171.0	70.9	188
4	RF02944 c4-2	100.0 (24/24)	83.3	82.8 (24/29)	122.5	49.8	224
5	RF02996 int-alpA	100.0 (23/23)	80.0	79.3 (23/29)	105.6	51.2	167
6	RF02965 CyVA-1	100.0 (18/18)	67.8	100.0 (18/18)	75.7	53.8	123
7	RF02925 6A	100.0 (10/10)	56.0	100.0 (10/10)	58.3	67.7	273
8	RF00049 SNORD36	100.0 (4/4)	42.5	100.0 (4/4)	42.8	66.9	59
9	RF00532 snoMe18S-Um1356	100.0 (3/3)	33.3	100.0 (3/3)	29.3	73.6	21
10	RF00537 snoMe28S-Am2634	100.0 (3/3)	23.3	100.0 (3/3)	22.3	72.5	18
11	RF00089 SNORD31	100.0 (2/2)	20.0	100.0 (2/2)	19.0	76.7	26
12	RF01218 snR41	100.0 (3/3)	13.3	100.0 (3/3)	12.3	76.8	18
13	RF00526 snoMe28S-U3344	100.0 (3/3)	10.0	100.0 (3/3)	12.7	77.8	8
14	RF00529 snoMe28S-Am2589	100.0 (3/3)	6.7	100.0 (3/3)	9.3	70.3	16
15	RF01196 snoZ30a	100.0 (2/2)	5.0	100.0 (2/2)	7.0	78.4	10
16	RF00282 SNORD48	100.0 (2/2)	0.0	100.0 (2/2)	4.5	90.4	8
17	RF00270 SNORD61	100.0 (2/2)	0.0	100.0 (2/2)	5.5	71.3	36
18	RF01593 plasmodium snoR16	100.0 (2/2)	0.0	100.0 (2/2)	3.0	77.2	3
19	RF03000 LOOT	95.3 (41/43)	92.8	47.7 (41/86)	317.2	48.3	368
20	RF00442 ykkC-yxkD	95.0 (19/20)	83.5	100.0 (19/19)	129.0	63.1	97
21	RF03064 RAGATH-18	94.4 (17/18)	99.4	73.9 (17/23)	443.7	61.5	1420
22	RF00031 SECIS 1	94.4 (17/18)	58.3	100.0 (17/17)	57.8	41.9	61
23	RF03019 RT-16	94.1 (32/34)	78.8	94.1 (32/34)	108.7	48.5	104
24	RF02947 cow-rumen-2	94.1 (16/17)	31.2	84.2 (16/19)	29.1	64.3	32
25	RF01731 TwoAYGGAY	93.3 (42/45)	83.5	93.3 (42/45)	130.8	48.8	210
26	RF02967 DUF3800-VII	93.1 (27/29)	61.0	100.0 (27/27)	70.3	52.0	139
27	RF02924 skipping-rope	92.8 (26/28)	95.7	100.0 (26/26)	451.8	53.5	1426
28	RF02012 group-II-D1D4-7	92.7 (38/41)	98.8	97.4 (38/39)	328.4	51.6	244
29	RF02958 drum	92.6 (25/27)	90.4	89.3 (25/28)	152.1	58.1	464
30	RF02913 pemK	92.3 (12/13)	95.4	92.3 (12/13)	321.6	65.0	1542
31	RF01699 Clostridiales-1	91.9 (57/62)	80.2	100.0 (57/57)	116.5	56.7	194
32	RF02921 RT-14	91.9 (34/37)	69.2	100.0 (34/34)	77.6	57.6	143
33	RF02990 gut-2	91.7 (11/12)	52.5	91.7 (11/12)	52.7	55.2	73
34	RF03074 Rhodo-rpoB	91.3 (21/23)	80.9	95.5 (21/22)	138.5	54.9	261
35	RF03044 Proteo-phage-1	90.9 (20/22)	35.5	100.0 (20/20)	34.5	60.2	67
36	RF02679 Pistol	90.5 (19/21)	40.5	100.0 (19/19)	38.6	59.2	45
37	RF00029 Intron gpII	89.5 (17/19)	65.3	100.0 (17/17)	81.8	54.0	92
38	RF02991 GP20-a	89.5 (17/19)	39.5	100.0 (17/17)	37.5	53.1	54
39	RF02003 group-II-D1D4-4	88.9 (24/27)	82.2	92.3 (24/26)	118.0	50.7	90
40	RF03037 PAGEV	88.1 (37/42)	48.6	97.4 (37/38)	47.8	53.6	86
41	RF03057 nhaA-I	87.5 (14/16)	82.5	100.0 (14/14)	182.9	58.7	281
42	RF02401 ClpQY promoter	87.5 (7/8)	27.5	100.0 (7/7)	26.5	70.6	39
43	RF00020 U5	86.7 (26/30)	88.3	78.8 (26/33)	167.9	52.7	180
44	RF00004 U2	86.7 (39/45)	81.5	88.6 (39/44)	180.1	59.4	208
45	RF02840 Ref68	86.7 (26/30)	38.3	100.0 (26/26)	36.2	58.9	78
46	RF00167 Purine	86.4 (19/22)	85.5	100.0 (19/19)	151.8	54.7	133
47	RF00023 tmRNA	85.7 (102/119)	99.2	85.0 (102/120)	635.3	44.6	477
48	RF03052 RAGATH-28	85.7 (12/14)	41.4	100.0 (12/12)	40.5	57.8	85
49	RF03027 RT-6	85.7 (12/14)	29.3	100.0 (12/12)	27.6	57.4	59
50	RF00230 T-box	85.1 (40/47)	55.1	100.0 (40/40)	57.5	49.6	48
51	RF02968 DUF3800-IX	85.0 (17/20)	84.5	85.0 (17/20)	150.8	52.9	229
52	RF00169 Bacteria small SRP	84.8 (28/33)	99.7	90.3 (28/31)	367.9	54.1	261
53	RF03003 GP20-b	84.6 (11/13)	70.8	68.8 (11/16)	93.7	56.3	210
54	RF00519 suhB	84.6 (11/13)	66.2	64.7 (11/17)	81.8	56.2	87
55	RF02937 Clostridiales-2	84.6 (11/13)	20.8	100.0 (11/11)	20.6	64.3	44
56	RF02986 FuFi-1	84.5 (49/58)	75.3	100.0 (49/49)	94.8	51.6	170
57	RF00174 Cobalamin	83.3 (35/42)	81.7	74.5 (35/47)	380.0	52.2	430
58	RF00133 SNORD33	83.3 (5/6)	66.7	100.0 (5/5)	71.8	62.9	72
59	RF01852 tRNA-Sec	83.3 (25/30)	48.0	92.6 (25/27)	49.2	57.0	109
60	RF03015 Transposase-2	83.3 (15/18)	45.5	100.0 (15/15)	48.7	65.8	151
61	RF02969 DUF3800-I	82.3 (28/34)	79.7	90.3 (28/31)	133.9	54.3	321
62	RF03084 DUF2815	81.8 (9/11)	16.4	100.0 (9/9)	16.5	65.7	22
63	RF02004 group-II-D1D4-5	81.5 (44/54)	88.9	93.6 (44/47)	160.9	57.0	139
64	RF02955 EGFOA	81.2 (26/32)	43.1	100.0 (26/26)	42.8	52.7	73
65	RF03047 RAGATH-16	81.2 (13/16)	12.5	86.7 (13/15)	13.2	70.2	18
66	RF00015 U4	80.6 (25/31)	89.7	92.6 (25/27)	158.9	58.7	170
67	RF02344 ar14	80.6 (25/31)	83.2	100.0 (25/25)	125.5	60.9	118
68	RF02932 Betaproteobacteria-1	80.5 (29/36)	61.7	93.5 (29/31)	72.0	55.7	102
69	RF01734 Fluoride	80.0 (8/10)	84.0	80.0 (8/10)	214.1	57.8	287
70	RF02221 sRNA-Xcc1	80.0 (16/20)	46.0	80.0 (16/20)	46.1	59.9	74
71	RF00309 snosnR60 Z15	80.0 (4/5)	30.0	100.0 (4/4)	28.2	65.3	23
72	RF02929 algC	80.0 (4/5)	20.0	100.0 (4/4)	20.8	88.0	492
73	RF00055 SNORD96	80.0 (4/5)	6.0	100.0 (4/4)	7.0	62.1	9

Continued on next page

	RNA family (seed alignment)	**Sensitivity** annotated bpairs that covary % (cov_bps/bps)	**Power** average power %	**Positive Predictive Value** covarying pairs in structure % (cov_bps/cov_pairs)	average substitutions per bpair	avg pairwise identity %	number of sequences
74	RF00010 RNaseP bact a	79.8 (91/114)	91.0	93.8 (91/97)	440.2	60.3	458
75	RF00013 6S	79.6 (39/49)	84.1	97.5 (39/40)	167.7	43.8	149
76	RF02005 group-II-D1D4-6	79.3 (46/58)	88.1	95.8 (46/48)	194.5	51.4	174
77	RF02953 DUF1874	79.3 (23/29)	45.2	95.8 (23/24)	44.9	61.8	76
78	RF03082 dinG	79.2 (19/24)	39.6	100.0 (19/19)	38.2	54.9	86
79	RF00009 RNaseP nuc	79.0 (49/62)	77.2	100.0 (49/49)	101.4	49.1	116
80	RF01705 Flavo-1	78.9 (15/19)	81.0	38.5 (15/39)	201.7	59.1	201
81	RF00557 L10 leader	78.9 (15/19)	73.2	100.0 (15/15)	110.6	47.6	97
82	RF01701 Cyano-1	78.6 (11/14)	89.3	100.0 (11/11)	266.4	61.7	265
83	RF01695 C4	77.8 (21/27)	88.5	70.0 (21/30)	393.3	56.0	456
84	RF01704 Downstream-peptide	77.8 (14/18)	83.3	100.0 (14/14)	528.4	61.6	627
85	RF03077 RT-2	77.8 (35/45)	73.1	92.1 (35/38)	102.7	49.5	195
86	RF03081 DUF805	77.8 (7/9)	20.0	100.0 (7/7)	19.2	69.8	46
87	RF01998 group-II-D1D4-1	77.3 (17/22)	84.1	100.0 (17/17)	275.0	57.6	237
88	RF01051 c-di-GMP-I	77.3 (17/22)	75.5	100.0 (17/17)	125.0	59.6	155
89	RF01761 wcaG	77.3 (17/22)	70.0	89.5 (17/19)	98.7	68.2	118
90	RF03014 Transposase-1	76.9 (10/13)	40.0	90.9 (10/11)	39.5	66.0	176
91	RF01690 Bacillaceae-1	76.5 (13/17)	72.9	100.0 (13/13)	94.3	63.7	95
92	RF01999 group-II-D1D4-2	76.3 (29/38)	79.7	96.7 (29/30)	134.8	60.4	144
93	RF02987 GA-cis	76.2 (16/21)	81.9	80.0 (16/20)	119.6	59.9	481
94	RF01749 pan	76.2 (16/21)	57.6	100.0 (16/16)	62.1	52.6	74
95	RF03075 DUF3800-VIII	76.0 (19/25)	72.0	95.0 (19/20)	102.4	59.5	209
96	RF00373 RNaseP arch	75.6 (62/82)	56.0	100.0 (62/62)	60.1	55.2	70
97	RF02001 group-II-D1D4-3	75.0 (36/48)	96.7	94.7 (36/38)	440.1	54.8	406
98	RF02960 DUF2800	75.0 (30/40)	50.2	96.8 (30/31)	55.2	58.9	155
99	RF00536 snoMe28S-Cm3227	75.0 (3/4)	15.0	100.0 (3/3)	15.5	90.4	13
100	RF00531 snoMe28S-Gm3113	75.0 (3/4)	12.5	100.0 (3/3)	13.0	75.9	15
101	RF00474 snosnR57	75.0 (3/4)	5.0	100.0 (3/3)	7.8	75.9	5
102	RF00471 snosnR48	75.0 (3/4)	2.5	100.0 (3/3)	4.8	75.9	6
103	RF02440 SpF59 sRNA	72.7 (8/11)	72.7	100.0 (8/8)	166.2	72.1	410
104	RF03070 ssNA-helicase	72.7 (8/11)	47.3	100.0 (8/8)	48.0	70.9	104
105	RF02916 atpB	72.7 (16/22)	42.7	100.0 (16/16)	42.0	72.3	72
106	RF01725 SAM-I-IV-variant	72.4 (21/29)	82.4	100.0 (21/21)	430.9	56.3	437
107	RF03069 malK-I	72.2 (39/54)	74.8	100.0 (39/39)	137.0	58.5	322
108	RF02914 DUF805b	71.4 (5/7)	88.6	100.0 (5/5)	450.4	73.0	1230
109	RF00059 TPP	71.4 (20/28)	70.7	100.0 (20/20)	117.6	55.0	109
110	RF02964 DUF3800-V	71.4 (20/28)	50.7	90.9 (20/22)	51.0	51.6	79
111	RF00619 U6atac	71.4 (10/14)	45.7	83.3 (10/12)	43.7	71.5	62
112	RF03032 narK	71.4 (5/7)	41.4	62.5 (5/8)	42.0	70.1	308
113	RF01748 nuoG	71.4 (5/7)	35.7	100.0 (5/5)	33.0	67.5	31
114	RF03080 RT-9	71.4 (10/14)	24.3	100.0 (10/10)	22.9	62.0	48
115	RF02926 DUF2693-FD	71.4 (10/14)	24.3	100.0 (10/10)	23.9	77.9	91
116	RF02977 folE	71.4 (5/7)	15.7	100.0 (5/5)	15.1	80.1	45
117	RF02980 freshwater-2	70.6 (12/17)	61.8	80.0 (12/15)	94.3	61.4	200
118	RF00011 RNaseP bact b	70.5 (74/105)	65.3	97.4 (74/76)	99.9	66.8	114
119	RF00162 SAM	70.4 (19/27)	72.6	67.8 (19/28)	273.8	64.1	433
120	RF03021 RT-18	70.0 (14/20)	53.0	100.0 (14/14)	53.5	62.1	141
121	RF03026 RT-5	70.0 (21/30)	41.3	95.5 (21/22)	41.5	55.5	71
122	RF01831 THF	69.4 (25/36)	70.0	100.0 (25/25)	97.4	60.3	97
123	RF03087 ROOL	69.2 (119/172)	93.4	60.7 (119/196)	180.1	44.6	290
124	RF01739 glnA	69.2 (9/13)	69.2	90.0 (9/10)	674.7	70.8	956
125	RF01687 Acido-Lenti-1	69.2 (9/13)	57.7	100.0 (9/9)	66.2	64.9	87
126	RF00030 RNase MRP	68.9 (51/74)	57.4	98.1 (51/52)	61.3	49.7	66
127	RF02032 GOLLD	68.4 (65/95)	48.8	83.3 (65/78)	47.9	49.2	35
128	RF02984 DUF3800-X	68.4 (13/19)	42.6	100.0 (13/13)	43.1	65.7	88
129	RF00127 t44	68.2 (15/22)	50.0	100.0 (15/15)	58.1	63.7	78
130	RF00080 yybP-ykoY	68.0 (17/25)	34.0	94.4 (17/18)	31.8	39.2	29
131	RF01055 MOCO RNA motif	66.7 (28/42)	71.4	93.3 (28/30)	109.8	56.9	160
132	RF02957 EFASI	66.7 (24/36)	54.2	100.0 (24/24)	58.6	58.9	448
133	RF00548 U11	66.7 (28/42)	50.5	80.0 (28/35)	53.1	58.9	72
134	RF00095 snoPyro CD	66.7 (2/3)	46.7	100.0 (2/2)	41.7	60.4	25
135	RF00032 Histone3	66.7 (4/6)	33.3	100.0 (4/4)	32.2	48.3	46
136	RF03020 RT-17	66.7 (8/12)	28.3	100.0 (8/8)	26.8	56.3	63
137	RF00008 Hammerhead 3	66.7 (10/15)	25.3	100.0 (10/10)	24.0	69.3	82
138	RF02684 Twister-P5	66.7 (12/18)	13.9	100.0 (12/12)	14.4	57.9	15
139	RF01192 SNORD11B	66.7 (4/6)	13.3	100.0 (4/4)	13.7	76.7	37
140	RF00274 SNORD57	66.7 (2/3)	6.7	100.0 (2/2)	9.3	78.4	23
141	RF00132 snoR24	66.7 (2/3)	6.7	100.0 (2/2)	7.0	80.6	12
142	RF03092 Ocean-VII	66.7 (4/6)	5.0	100.0 (4/4)	7.2	83.4	16
143	RF01637 ceN47	66.7 (2/3)	3.3	100.0 (2/2)	8.0	76.0	6
144	RF00539 snoMe28S-Cm788	66.7 (2/3)	0.0	100.0 (2/2)	2.0	89.7	5
145	RF00070 SNORD29	66.7 (2/3)	0.0	100.0 (2/2)	2.0	75.2	10
146	RF02034 IMES-1	65.7 (23/35)	86.6	100.0 (23/23)	207.8	71.6	219
147	RF01764 yjdF	65.2 (15/23)	54.3	100.0 (15/15)	59.4	67.3	62
148	RF00001 5S rRNA	64.7 (22/34)	98.5	71.0 (22/31)	306.1	56.1	712
149	RF01685 6S-Flavo	64.7 (11/17)	54.7	100.0 (11/11)	65.3	71.5	82
150	RF03022 RT-10	64.5 (20/31)	44.8	100.0 (20/20)	44.6	58.1	113
151	RF00168 Lysine	64.2 (34/53)	54.1	100.0 (34/34)	55.4	49.2	47
152	RF03055 RAGATH-30	63.6 (7/11)	15.4	100.0 (7/7)	16.0	69.2	33
153	RF01754 radC	62.9 (22/35)	72.3	88.0 (22/25)	105.7	58.9	130
154	RF02033 HEARO	62.9 (22/35)	62.9	100.0 (22/22)	84.9	59.5	110
155	RF03013 nadA	62.5 (15/24)	69.2	100.0 (15/15)	115.5	58.7	182
156	RF02940 COG3860	62.5 (5/8)	23.8	100.0 (5/5)	23.0	76.1	42
157	RF02976 Flavobacterium-1	62.2 (23/37)	42.4	95.8 (23/24)	44.8	62.3	210

Continued on next page

	RNA family (seed alignment)	Sensitivity annotated bpairs that covary % (cov_bps/bps)	Power average power %	Positive Predictive Value covarying pairs in structure % (cov_bps/cov_pairs)	average substitutions per bpair	avg pairwise identity %	number of sequences
158	RF02933 ARRPOF	62.2 (46/74)	23.1	100.0 (46/46)	22.3	56.2	35
159	RF02956 DUF2693	62.1 (18/29)	15.2	100.0 (18/18)	15.5	65.0	63
160	RF01706 Gut-1	61.9 (13/21)	21.4	100.0 (13/13)	20.4	62.5	38
161	RF02541 LSU rRNA bacteria	61.6 (521/846)	57.5	95.9 (521/543)	83.5	69.4	102
162	RF03054 NMT1	61.5 (8/13)	60.0	100.0 (8/8)	128.2	62.3	382
163	RF01068 mini-ykkC	61.5 (8/13)	55.4	100.0 (8/8)	72.8	55.2	72
164	RF01057 SAH riboswitch	61.5 (8/13)	50.8	100.0 (8/8)	56.3	61.7	52
165	RF02253 IRE II	61.5 (8/13)	32.3	100.0 (8/8)	29.8	58.4	29
166	RF03068 RT-3	60.6 (20/33)	54.5	71.4 (20/28)	59.2	66.9	231
167	RF01707 JUMPstart	60.0 (3/5)	58.0	100.0 (3/3)	71.8	78.2	128
168	RF02915 DUF3800-VI	60.0 (12/20)	40.5	100.0 (12/12)	40.2	60.8	87
169	RF01727 SAM-SAH	60.0 (6/10)	36.0	100.0 (6/6)	37.4	67.0	53
170	RF02966 DUF3268	60.0 (21/35)	35.7	100.0 (21/21)	34.3	65.3	108
171	RF00003 U1	60.0 (24/40)	34.2	100.0 (24/24)	32.0	63.7	100
172	RF00522 PreQ1	60.0 (3/5)	24.0	100.0 (3/3)	21.2	65.7	35
173	RF01718 Polynucleobacter-1	60.0 (18/30)	16.0	100.0 (18/18)	15.9	54.5	16
174	RF00149 snoZ103	60.0 (3/5)	6.0	75.0 (3/4)	7.8	74.5	12
175	RF01198 snoR69Y	60.0 (3/5)	6.0	100.0 (3/3)	8.8	66.6	7
176	RF00380 ykoK	59.6 (28/47)	77.2	93.3 (28/30)	128.3	58.9	157
177	RF00504 Glycine	59.1 (13/22)	45.0	100.0 (13/13)	47.8	54.3	44
178	RF02683 NiCo	59.1 (13/22)	40.0	100.0 (13/13)	42.5	64.1	82
179	RF01786 c-di-GMP-II	58.6 (17/29)	56.9	100.0 (17/17)	63.1	55.2	54
180	RF03005 lysM-TM7	58.6 (17/29)	31.0	100.0 (17/17)	30.2	64.7	129
181	RF01746 mraW	58.3 (21/36)	50.0	100.0 (21/21)	52.1	50.7	45
182	RF01763 ykkC-III	58.3 (7/12)	36.7	100.0 (7/7)	35.7	62.8	39
183	RF02962 DUF3800-III	58.1 (18/31)	29.7	94.7 (18/19)	29.0	61.5	55
184	RF01750 ZMP-ZTP	57.9 (11/19)	75.3	73.3 (11/15)	174.4	57.7	182
185	RF01849 alpha tmRNA	57.4 (31/54)	58.7	96.9 (31/32)	83.4	62.5	111
186	RF02540 LSU rRNA archaea	57.4 (451/786)	56.8	97.4 (451/463)	73.1	68.3	91
187	RF00007 U12	57.1 (24/42)	42.9	100.0 (24/24)	42.4	62.2	62
188	RF03034 nrdJ	57.1 (12/21)	34.3	100.0 (12/12)	34.4	70.5	73
189	RF01712 Methylobacterium-1	57.1 (16/28)	32.1	100.0 (16/16)	30.4	61.9	34
190	RF00335 snoZ13 snr52	57.1 (4/7)	27.1	100.0 (4/4)	29.4	73.3	42
191	RF02982 gltS	57.1 (8/14)	19.3	100.0 (8/8)	18.9	75.2	108
192	RF03072 raiA	56.9 (33/58)	62.8	82.5 (33/40)	122.1	69.0	488
193	RF00050 FMN	56.5 (13/23)	51.3	81.2 (13/16)	82.2	69.2	144
194	RF00233 Tymo tRNA-like	56.5 (13/23)	25.2	100.0 (13/13)	23.6	69.0	28
195	RF03071 DUF1646	56.2 (9/16)	37.5	100.0 (9/9)	37.2	62.6	146
196	RF03012 Mu-gpT-DE	55.5 (5/9)	43.3	62.5 (5/8)	78.7	74.2	316
197	RF03085 abiF	55.5 (5/9)	32.2	100.0 (5/5)	33.7	74.9	126
198	RF03050 RAGATH-25	55.5 (5/9)	7.8	100.0 (5/5)	9.6	69.3	21
199	RF02948 cow-rumen-4	55.5 (10/18)	3.9	100.0 (10/10)	6.4	61.8	8
200	RF00379 ydaO-yuaA	53.8 (14/26)	52.7	82.3 (14/17)	70.6	58.8	106
201	RF03067 terC	53.3 (8/15)	50.0	100.0 (8/8)	57.9	61.2	129
202	RF03100 RAGATH-27	53.3 (8/15)	7.3	100.0 (8/8)	9.3	69.9	15
203	RF03110 throat-1	53.1 (34/64)	14.1	100.0 (34/34)	15.0	71.5	27
204	RF03046 Pseudomonadales-1	52.5 (21/40)	23.5	87.5 (21/24)	22.5	53.0	54
205	RF00012 U3	52.4 (33/63)	48.7	100.0 (33/33)	50.8	60.1	87
206	RF01857 Archaea SRP	52.3 (69/132)	58.6	98.6 (69/70)	62.4	52.5	53
207	RF00177 SSU rRNA bacteria	52.2 (241/462)	50.8	99.6 (241/242)	70.7	73.3	99
208	RF02954 DUF3577	52.2 (12/23)	30.9	100.0 (12/12)	30.4	65.1	86
209	RF01794 sok	51.9 (14/27)	48.9	82.3 (14/17)	59.8	58.4	91
210	RF02951 DABA-DC-AT	51.5 (17/33)	32.4	100.0 (17/17)	31.5	71.7	126
211	RF01745 manA	50.0 (29/58)	75.5	61.7 (29/47)	119.9	75.2	189
212	RF00559 L21 leader	50.0 (9/18)	40.0	100.0 (9/9)	40.0	55.3	38
213	RF01708 L17DE	50.0 (11/22)	37.3	100.0 (11/11)	39.0	63.7	52
214	RF02278 Betaproteobacteria toxic sRNA	50.0 (8/16)	27.5	88.9 (8/9)	27.7	70.8	51
215	RF00538 snoCD11	50.0 (3/6)	21.7	100.0 (3/3)	20.0	77.3	26
216	RF00325 SNORD53 SNORD92	50.0 (2/4)	17.5	100.0 (2/2)	18.0	74.5	37
217	RF02961 DUF3800-II	50.0 (10/20)	16.0	90.9 (10/11)	15.7	63.6	23
218	RF00151 SNORD58	50.0 (2/4)	12.5	100.0 (2/2)	14.0	79.5	33
219	RF01209 snR76	50.0 (2/4)	10.0	100.0 (2/2)	12.2	74.3	15
220	RF00287 SNORD44	50.0 (1/2)	10.0	100.0 (1/1)	11.0	84.4	12
221	RF00345 snoR1	50.0 (1/2)	10.0	100.0 (1/1)	12.0	73.7	13
222	RF00071 SNORD73	50.0 (2/4)	7.5	100.0 (2/2)	9.0	79.1	25
223	RF00086 SNORD27	50.0 (2/4)	7.5	66.7 (2/3)	8.8	75.6	8
224	RF00300 snoZ221 snoR21b	50.0 (2/4)	5.0	100.0 (2/2)	8.0	74.6	12
225	RF00221 SNORD43	50.0 (2/4)	2.5	100.0 (2/2)	4.0	85.3	4
226	RF00069 SNORD24	50.0 (2/4)	2.5	100.0 (2/2)	5.2	71.8	14
227	RF01302 snoU36a	50.0 (2/4)	2.5	100.0 (2/2)	3.5	76.2	15
228	RF01221 snoU6-47	50.0 (2/4)	2.5	100.0 (2/2)	2.8	62.8	5
229	RF00211 SNORD35	50.0 (2/4)	0.0	100.0 (2/2)	1.5	72.1	8
230	RF01826 SAM V	50.0 (2/4)	0.0	100.0 (2/2)	3.2	78.7	6
231	RF01284 snoR8a	50.0 (2/4)	0.0	100.0 (2/2)	2.8	73.3	7
232	RF00525 Flavivirus DB	47.4 (9/19)	54.7	100.0 (9/9)	81.8	73.0	111
233	RF02917 Burkholderiales-2	47.4 (9/19)	4.7	100.0 (9/9)	5.9	76.6	19
234	RF02943 Bacteroidales-2	47.3 (35/74)	8.2	100.0 (35/35)	10.0	74.4	43
235	RF00618 U4atac	46.7 (14/30)	34.0	100.0 (14/14)	33.7	62.5	61
236	RF01844 SmY	46.7 (7/15)	34.0	100.0 (7/7)	34.2	60.1	61
237	RF02496 Atu At1	46.4 (13/28)	25.0	100.0 (13/13)	23.5	64.6	33
238	RF00515 PyrR	46.3 (19/41)	51.7	95.0 (19/20)	52.9	48.6	41
239	RF01960 SSU rRNA eukarya	45.9 (205/447)	53.9	98.1 (205/209)	70.1	62.6	91
240	RF02988 GEBRO	45.8 (33/72)	13.5	100.0 (33/33)	14.1	69.8	42
241	RF01793 ffh	45.5 (5/11)	38.2	100.0 (5/5)	38.9	64.4	89

Continued on next page

	RNA family (seed alignment)	**Sensitivity** annotated bpairs that covary % (cov_bps/bps)	**Power** average power %	**Positive Predictive Value** covarying pairs in structure % (cov_bps/cov_pairs)	average substitutions per bpair	avg pairwise identity %	number of sequences
242	RF00066 U7	45.5 (5/11)	36.4	100.0 (5/5)	34.9	66.7	49
243	RF03079 MISL	45.5 (90/198)	28.6	100.0 (90/90)	27.1	57.0	44
244	RF00634 SAM-IV	45.2 (14/31)	23.2	100.0 (14/14)	24.0	70.4	40
245	RF02379 csRNA	45.0 (9/20)	23.5	100.0 (9/9)	22.7	79.3	48
246	RF02345 ar15	44.8 (13/29)	33.4	100.0 (13/13)	35.0	71.4	61
247	RF03025 RT-4	44.8 (13/29)	19.0	92.8 (13/14)	18.3	68.5	47
248	RF00234 glmS	44.7 (17/38)	19.5	100.0 (17/17)	18.7	59.1	18
249	RF03018 RT-15	44.6 (25/56)	27.9	100.0 (25/25)	28.2	66.9	64
250	RF01394 isrK	44.4 (8/18)	30.0	80.0 (8/10)	30.9	60.6	52
251	RF03039 Peptidase-S11	44.4 (8/18)	23.3	100.0 (8/8)	23.3	63.1	135
252	RF01069 purD	44.4 (12/27)	22.6	100.0 (12/12)	21.9	58.9	21
253	RF01691 Bacillus-plasmid	44.4 (4/9)	16.7	100.0 (4/4)	16.3	68.4	31
254	RF00002 5 8S rRNA	44.0 (11/25)	40.4	100.0 (11/11)	42.8	67.9	61
255	RF01959 SSU rRNA archaea	43.1 (197/457)	44.3	100.0 (197/197)	54.2	75.5	86
256	RF01689 AdoCbl-variant	42.9 (15/35)	52.3	100.0 (15/15)	64.4	78.6	144
257	RF00213 snoR38	42.9 (3/7)	24.3	100.0 (3/3)	22.4	58.6	19
258	RF03051 RAGATH-26	42.9 (3/7)	4.3	100.0 (3/3)	6.4	79.0	8
259	RF03111 Zeta-pan	42.9 (3/7)	0.0	100.0 (3/3)	3.0	80.3	11
260	RF02942 Clostridiales-3	42.6 (23/54)	28.9	100.0 (23/23)	28.3	70.3	157
261	RF02543 LSU rRNA eukarya	42.4 (370/872)	52.8	97.9 (370/378)	68.9	65.9	88
262	RF03028 RT-7	42.0 (21/50)	23.8	100.0 (21/21)	22.9	56.6	54
263	RF03065 IS605-orfB-I	41.9 (26/62)	60.0	65.0 (26/40)	77.9	61.2	259
264	RF01709 Lacto-rpoB	41.7 (5/12)	40.8	100.0 (5/5)	37.9	61.4	31
265	RF00164 s2m	41.7 (5/12)	22.5	100.0 (5/5)	22.8	78.4	38
266	RF02946 cow-rumen-1	41.7 (5/12)	15.8	100.0 (5/5)	16.3	74.8	50
267	RF00110 RybB	41.7 (5/12)	2.5	100.0 (5/5)	4.6	82.5	8
268	RF01688 Actino-pnp	40.9 (9/22)	45.0	100.0 (9/9)	56.1	67.6	77
269	RF01846 Fungi U3	40.5 (32/79)	60.0	94.1 (32/34)	69.0	56.4	66
270	RF00140 Alpha RBS	40.0 (8/20)	35.5	88.9 (8/9)	35.9	65.4	39
271	RF00324 snoMBII-202	40.0 (2/5)	28.0	66.7 (2/3)	25.8	73.4	30
272	RF02682 HDV-F-prausnitzii	40.0 (6/15)	26.7	100.0 (6/6)	27.5	66.0	48
273	RF02998 IS605-orfB-II	40.0 (18/45)	20.7	100.0 (18/18)	20.9	69.9	56
274	RF01072 TMV UPD-PK3	40.0 (4/10)	15.0	100.0 (4/4)	14.8	81.5	25
275	RF00608 SNORD99	40.0 (2/5)	12.0	100.0 (2/2)	10.8	77.0	23
276	RF00613 SNORD94	40.0 (2/5)	10.0	100.0 (2/2)	11.0	79.0	21
277	RF00150 SNORD42	40.0 (2/5)	4.0	100.0 (2/2)	5.8	73.9	7
278	RF00458 IRES Cripavirus	40.0 (24/60)	3.7	100.0 (24/24)	6.4	54.8	7
279	RF00204 snoR12	40.0 (2/5)	2.0	66.7 (2/3)	5.8	79.7	9
280	RF02784 SorX	40.0 (6/15)	2.0	100.0 (6/6)	3.5	78.5	8
281	RF01317 CRISPR-DR4	40.0 (2/5)	2.0	100.0 (2/2)	3.6	83.6	33
282	RF00203 snoR160	40.0 (2/5)	0.0	100.0 (2/2)	2.0	74.3	9
283	RF01084 TLS-PK3	39.5 (17/43)	3.7	100.0 (17/17)	5.9	58.6	8
284	RF02035 IMES-2	38.5 (15/39)	63.3	93.8 (15/16)	189.4	79.3	366
285	RF00114 S15	38.5 (5/13)	36.9	100.0 (5/5)	38.2	61.0	78
286	RF01290 SNORD10	38.5 (5/13)	18.5	62.5 (5/8)	18.7	64.9	22
287	RF02923 HTH-XRE	38.5 (5/13)	15.4	83.3 (5/6)	15.8	77.4	60
288	RF00057 RyhB	38.5 (5/13)	11.5	100.0 (5/5)	10.7	75.3	26
289	RF03058 sul1	38.5 (5/13)	10.0	100.0 (5/5)	11.2	77.0	54
290	RF03016 RT-12	37.8 (14/37)	19.4	93.3 (14/15)	19.2	56.3	42
291	RF03086 chrB-b	37.5 (6/16)	51.2	100.0 (6/6)	79.0	68.9	313
292	RF01692 Bacteroid-trp	37.5 (9/24)	37.1	100.0 (9/9)	35.8	54.2	35
293	RF00558 L20 leader	37.5 (9/24)	30.8	100.0 (9/9)	30.2	53.4	43
294	RF02952 dfrA-dnaX	37.5 (3/8)	17.5	100.0 (3/3)	19.1	74.7	138
295	RF02930 aspS	37.5 (9/24)	16.7	100.0 (9/9)	16.7	68.0	58
296	RF00617 cHP	37.5 (3/8)	8.8	100.0 (3/3)	10.2	72.7	59
297	RF03041 poplar-1	37.5 (12/32)	8.4	92.3 (12/13)	10.0	69.1	22
298	RF00173 Hairpin	37.5 (3/8)	0.0	100.0 (3/3)	1.2	82.2	5
299	RF03007 Mahella-1	36.4 (8/22)	7.7	88.9 (8/9)	9.6	64.2	19
300	RF03062 xerDC	35.9 (14/39)	13.1	100.0 (14/14)	13.9	75.9	72
301	RF00053 mir-7	35.7 (10/28)	33.9	100.0 (10/10)	36.5	65.2	57
302	RF02276 Hammerhead II	35.3 (6/17)	17.6	100.0 (6/6)	17.4	65.3	24
303	RF01732 asd	34.9 (15/43)	29.5	100.0 (15/15)	28.2	70.4	31
304	RF03045 proV	34.6 (9/26)	6.5	100.0 (9/9)	8.4	71.2	16
305	RF01820 RsaE	34.5 (10/29)	7.6	100.0 (10/10)	8.1	75.9	15
306	RF01760 traJ-II	33.3 (11/33)	40.0	91.7 (11/12)	39.5	70.3	47
307	RF00530 snoMe28S-Cm2645	33.3 (1/3)	30.0	100.0 (1/1)	28.0	79.0	20
308	RF01848 ACEA U3	33.3 (17/51)	29.4	100.0 (17/17)	27.5	47.6	28
309	RF03078 chrB-a	33.3 (4/12)	25.0	66.7 (4/6)	27.7	68.7	163
310	RF00609 SNORD100	33.3 (2/6)	20.0	100.0 (2/2)	20.2	76.1	26
311	RF03053 OTKONC	33.3 (10/30)	9.0	55.5 (10/18)	10.5	74.9	43
312	RF03049 RAGATH-24	33.3 (9/27)	7.8	100.0 (9/9)	9.4	73.3	22
313	RF03006 M23	33.3 (4/12)	7.5	100.0 (4/4)	7.5	73.0	31
314	RF01183 SNORD19B	33.3 (1/3)	6.7	100.0 (1/1)	9.0	77.5	17
315	RF00533 snoMe18S-Gm1358	33.3 (1/3)	6.7	100.0 (1/1)	9.7	81.2	12
316	RF02918 MDR-NUDIX	33.3 (5/15)	6.0	100.0 (5/5)	7.1	70.0	15
317	RF03010 mcrA	33.3 (2/6)	5.0	100.0 (2/2)	8.5	80.9	16
318	RF02451 ncr1241	33.3 (5/15)	3.3	100.0 (5/5)	5.5	72.0	8
319	RF00295 snoTBR7	33.3 (1/3)	3.3	100.0 (1/1)	6.3	78.5	10
320	RF00521 SAM alpha	33.3 (2/6)	3.3	100.0 (2/2)	5.8	70.5	40
321	RF00477 snosnR66	33.3 (2/6)	3.3	100.0 (2/2)	6.5	83.7	4
322	RF00276 SNORD52	33.3 (1/3)	3.3	100.0 (1/1)	8.0	81.1	20
323	RF00360 snoZ107 R87	33.3 (2/6)	1.7	100.0 (2/2)	4.3	74.3	9
324	RF01179 snoU83	33.3 (1/3)	0.0	100.0 (1/1)	2.7	64.1	7
325	RF01594 snoR18	33.3 (1/3)	0.0	100.0 (1/1)	1.3	83.7	3

Continued on next page

	RNA family (seed alignment)	Sensitivity annotated bpairs that covary % (cov_bps/bps)	Power average power %	Positive Predictive Value covarying pairs in structure % (cov_bps/cov_pairs)	average substitutions per bpair	avg pairwise identity %	number of sequences
326	RF01711 Lnt	33.3 (2/6)	0.0	100.0 (2/2)	2.8	76.5	10
327	RF01348 CRISPR-DR38	33.3 (1/3)	0.0	100.0 (1/1)	0.7	81.7	5
328	RF01599 plasmodium snoR24	33.3 (1/3)	0.0	100.0 (1/1)	2.7	79.6	4
329	RF01285 snoU31b	33.3 (1/3)	0.0	100.0 (1/1)	3.3	70.2	7
330	RF01288 snoR31	33.3 (1/3)	0.0	100.0 (1/1)	2.7	70.8	6
331	RF00017 Metazoa SRP	32.5 (39/120)	60.6	100.0 (39/39)	71.5	68.1	91
332	RF02928 Actinomyces-1	32.4 (11/34)	40.9	100.0 (11/11)	44.0	70.4	236
333	RF00685 mir-36	32.1 (9/28)	31.1	100.0 (9/9)	30.2	53.9	46
334	RF01066 6C	32.0 (8/25)	10.4	100.0 (8/8)	11.9	71.8	18
335	RF02194 HPnc0260	31.8 (7/22)	21.8	100.0 (7/7)	21.4	56.5	31
336	RF02542 SSU rRNA microsporidia	31.4 (115/366)	34.9	99.1 (115/116)	36.1	67.6	46
337	RF03009 malK-III	31.2 (5/16)	6.2	100.0 (5/5)	6.5	69.7	39
338	RF02941 COG3943	31.0 (9/29)	11.0	100.0 (9/9)	11.4	69.7	27
339	RF01747 msiK	30.8 (4/13)	60.0	100.0 (4/4)	81.5	68.2	120
340	RF01071 OLE	30.8 (49/159)	21.9	96.1 (49/51)	21.2	62.8	20
341	RF02931 Bacilli-1	30.8 (4/13)	10.0	100.0 (4/4)	11.9	86.2	145
342	RF02963 DUF3800-IV	30.6 (11/36)	4.2	91.7 (11/12)	6.7	59.6	10
343	RF01693 Bacteroidales-1	30.4 (7/23)	23.9	100.0 (7/7)	23.2	75.2	41
344	RF03097 RAGATH-21	30.4 (7/23)	10.4	100.0 (7/7)	11.0	76.3	38
345	RF01728 STAXI	30.3 (10/33)	32.1	76.9 (10/13)	30.4	57.6	30
346	RF01859 Phe leader	30.0 (12/40)	37.8	100.0 (12/12)	37.0	64.2	71
347	RF03099 RAGATH-23	30.0 (6/20)	2.0	100.0 (6/6)	3.9	78.5	11
348	RF02993 ilvH	29.4 (5/17)	4.7	100.0 (5/5)	5.6	81.3	17
349	RF03030 salivarius-1	29.4 (5/17)	3.5	100.0 (5/5)	6.7	72.5	19
350	RF00711 mir-449	28.6 (8/28)	40.7	100.0 (8/8)	41.5	57.7	56
351	RF00604 SNORD88	28.6 (2/7)	20.0	100.0 (2/2)	19.6	75.4	35
352	RF03036 osmY	28.6 (4/14)	9.3	100.0 (4/4)	10.6	70.6	38
353	RF03001 leuA-Halobacteria	28.6 (2/7)	8.6	100.0 (2/2)	8.3	76.1	152
354	RF00580 SNORD91	28.6 (2/7)	5.7	100.0 (2/2)	7.7	79.1	24
355	RF00288 snoZ30	28.6 (2/7)	5.7	100.0 (2/2)	6.6	82.7	20
356	RF02959 DUF3085	27.8 (5/18)	5.0	100.0 (5/5)	6.7	69.9	16
357	RF00016 SNORD14	27.3 (3/11)	9.1	100.0 (3/3)	11.2	67.7	18
358	RF02927 Actino-ugpB	27.3 (3/11)	0.9	100.0 (3/3)	3.5	72.4	12
359	RF01847 Plant U3	27.1 (19/70)	23.1	100.0 (19/19)	22.5	61.6	24
360	RF03103 RAGATH-34	26.9 (7/26)	8.1	100.0 (7/7)	10.4	73.9	24
361	RF02949 Cupriavidus-1	26.9 (7/26)	0.0	100.0 (7/7)	2.7	62.7	5
362	RF00037 IRE I	26.7 (4/15)	33.3	100.0 (4/4)	35.4	76.7	61
363	RF03063 Streptomyces-metK	26.7 (4/15)	11.3	100.0 (4/4)	11.7	76.4	38
364	RF03048 RAGATH-20	26.7 (4/15)	2.0	100.0 (4/4)	4.9	82.5	9
365	RF02342 ar7	26.5 (9/34)	20.9	100.0 (9/9)	19.8	65.7	29
366	RF00006 Vault	26.3 (5/19)	25.8	100.0 (5/5)	25.3	59.3	73
367	RF02938 COG2827	26.3 (5/19)	18.4	100.0 (5/5)	18.4	70.1	48
368	RF00616 LhrC	26.3 (5/19)	2.6	100.0 (5/5)	3.7	90.6	12
369	RF01702 Cyano-2	26.1 (12/46)	36.1	100.0 (12/12)	37.3	63.5	57
370	RF01724 SAM-Chlorobi	26.1 (6/23)	2.2	100.0 (6/6)	3.9	76.6	9
371	RF02972 engA	25.9 (7/27)	20.7	87.5 (7/8)	21.3	70.3	154
372	RF00025 Telomerase-cil	25.7 (9/35)	27.4	100.0 (9/9)	25.6	54.6	20
373	RF02995 IMES-5	25.6 (11/43)	13.5	100.0 (11/11)	14.0	69.4	52
374	RF00024 Telomerase-vert	25.5 (27/106)	18.0	100.0 (27/27)	17.6	61.5	37
375	RF00096 U8	25.0 (6/24)	43.3	100.0 (6/6)	43.2	67.5	55
376	RF01067 ATPC	25.0 (6/24)	40.4	100.0 (6/6)	42.2	73.8	90
377	RF00391 RtT	25.0 (6/24)	26.2	75.0 (6/8)	25.7	68.3	57
378	RF00555 L13 leader	25.0 (4/16)	25.0	100.0 (4/4)	24.2	68.6	26
379	RF00517 serC	25.0 (3/12)	21.7	100.0 (3/3)	22.5	75.3	70
380	RF00099 SNORD22	25.0 (1/4)	20.0	100.0 (1/1)	19.2	79.7	49
381	RF03059 raiA-hairpin	25.0 (2/8)	17.5	100.0 (2/2)	18.5	81.0	80
382	RF01762 whalefall-1	25.0 (4/16)	13.1	100.0 (4/4)	12.7	71.6	14
383	RF03060 uup	25.0 (2/8)	11.2	100.0 (2/2)	11.0	69.7	203
384	RF00280 SNORD51	25.0 (1/4)	10.0	100.0 (1/1)	12.2	86.6	14
385	RF00368 sroB	25.0 (4/16)	10.0	100.0 (4/4)	10.7	67.9	16
386	RF02685 RAGATH-5	25.0 (3/12)	9.2	60.0 (3/5)	9.3	74.7	39
387	RF02992 hya	25.0 (3/12)	7.5	100.0 (3/3)	9.8	77.4	27
388	RF00528 snoMe28S-Gm1083	25.0 (1/4)	7.5	100.0 (1/1)	9.5	85.7	11
389	RF00572 SNORD66	25.0 (1/4)	7.5	100.0 (1/1)	9.0	81.9	20
390	RF00085 SNORD28	25.0 (1/4)	7.5	100.0 (1/1)	7.5	75.9	20
391	RF00273 SNORD59	25.0 (1/4)	5.0	100.0 (1/1)	8.0	77.9	24
392	RF00588 SNORD41	25.0 (1/4)	5.0	100.0 (1/1)	5.8	77.2	20
393	RF03008 malK-II	25.0 (2/8)	5.0	100.0 (2/2)	7.5	80.5	94
394	RF00479 snosnR71	25.0 (1/4)	5.0	100.0 (1/1)	8.5	73.7	7
395	RF01316 CRISPR-DR3	25.0 (1/4)	5.0	100.0 (1/1)	4.0	84.0	20
396	RF03073 RT-19	25.0 (4/16)	3.1	100.0 (4/4)	4.7	80.8	23
397	RF01170 snoU61	25.0 (1/4)	2.5	100.0 (1/1)	4.8	74.7	8
398	RF00160 snoZ159	25.0 (1/4)	2.5	100.0 (1/1)	4.5	82.0	10
399	RF02459 Virus CITE 4	25.0 (6/24)	1.7	100.0 (6/6)	3.5	72.0	5
400	RF02844 RefA9	25.0 (4/16)	1.2	100.0 (4/4)	2.9	62.2	4
401	RF02939 COG2908	25.0 (3/12)	0.8	100.0 (3/3)	2.0	82.2	9
402	RF00356 snoR32 R81	25.0 (1/4)	0.0	100.0 (1/1)	1.0	83.2	6
403	RF01426 snoR126	25.0 (1/4)	0.0	100.0 (1/1)	1.2	67.3	4
404	RF02519 ToxI	25.0 (1/4)	0.0	100.0 (1/1)	0.5	87.8	4
405	RF00153 SNORD62	25.0 (1/4)	0.0	100.0 (1/1)	1.2	86.2	14
406	RF01866 ceN33	25.0 (1/4)	0.0	100.0 (1/1)	2.5	90.0	5
407	RF01280 snoR14	25.0 (1/4)	0.0	100.0 (1/1)	0.5	79.3	7
408	RF00475 snosnR69	25.0 (1/4)	0.0	100.0 (1/1)	2.5	64.1	6
409	RF02896 S774	25.0 (3/12)	0.0	100.0 (3/3)	2.8	74.8	4

Continued on next page

	RNA family (seed alignment)	Sensitivity annotated bpairs that covary % (cov_bps/bps)	Power average power %	Positive Predictive Value covarying pairs in structure % (cov_bps/cov_pairs)	average substitutions per bpair	avg pairwise identity %	number of sequences
410	RF00612 SNORD75	25.0 (1/4)	0.0	100.0 (1/1)	3.0	74.5	5
411	RF00281 SNORD47	25.0 (1/4)	0.0	100.0 (1/1)	3.5	82.5	20
412	RF01502 Fungi SRP	24.8 (29/117)	66.7	100.0 (29/29)	72.5	43.6	50
413	RF01256 snR43	24.1 (13/54)	16.7	100.0 (13/13)	16.3	64.3	20
414	RF01730 Termite-leu	23.8 (5/21)	3.3	100.0 (5/5)	6.2	65.6	20
415	RF01054 preQ1-II	23.5 (4/17)	8.8	100.0 (4/4)	9.8	70.5	14
416	RF00435 ROSE	23.5 (8/34)	5.0	100.0 (8/8)	6.8	61.8	13
417	RF02680 PreQ1-III	23.1 (6/26)	13.5	100.0 (6/6)	13.3	81.9	28
418	RF03076 Streptomyces-metH	23.1 (3/13)	12.3	75.0 (3/4)	11.9	66.4	43
419	RF03083 cow-rumen-3	23.1 (9/39)	5.1	100.0 (9/9)	7.0	69.2	8
420	RF03093 queA	23.1 (3/13)	3.1	100.0 (3/3)	4.8	71.9	8
421	RF02950 D12-methyl	23.1 (3/13)	2.3	100.0 (3/3)	3.8	84.7	19
422	RF03066 COG3610-DE	22.9 (8/35)	10.6	100.0 (8/8)	11.5	73.6	59
423	RF00645 MIR169 2	22.7 (5/22)	37.7	83.3 (5/6)	44.2	52.9	100
424	RF01735 epsC	22.5 (9/40)	14.2	100.0 (9/9)	14.3	76.7	30
425	RF00353 snoR31 Z110 Z27	22.2 (2/9)	5.5	100.0 (2/2)	8.1	67.2	8
426	RF02435 SpF41 sRNA	22.2 (6/27)	5.2	100.0 (6/6)	6.9	69.3	9
427	RF03040 PGK	22.2 (4/18)	2.8	100.0 (4/4)	4.9	70.9	18
428	RF02399 NsiR1	21.4 (3/14)	10.7	100.0 (3/3)	10.9	73.3	17
429	RF00163 Hammerhead 1	21.4 (3/14)	10.7	100.0 (3/3)	12.1	67.8	29
430	RF01721 Pseudomon-groES	21.4 (6/28)	7.1	100.0 (6/6)	7.4	71.1	18
431	RF00507 Corona FSE	21.1 (4/19)	4.7	80.0 (4/5)	6.7	66.5	23
432	RF00236 ctRNA pGA1	20.8 (5/24)	7.1	100.0 (5/5)	7.8	74.2	15
433	RF02678 Hatchet	20.8 (5/24)	2.5	100.0 (5/5)	5.2	54.9	8
434	RF02999 ivy-DE	20.7 (6/29)	21.4	100.0 (6/6)	21.6	76.0	248
435	RF01867 CC2171	20.7 (6/29)	7.6	100.0 (6/6)	8.1	66.8	13
436	RF02457 Virus CITE 2	20.3 (12/59)	3.2	100.0 (12/12)	5.4	71.9	9
437	RF02975 DUF3800-XI	20.0 (2/10)	40.0	66.7 (2/3)	43.7	72.1	256
438	RF00181 SNORD113	20.0 (1/5)	28.0	100.0 (1/1)	25.8	68.0	59
439	RF00284 SNORD74	20.0 (1/5)	26.0	100.0 (1/1)	24.4	78.5	22
440	RF01116 Yfr1	20.0 (3/15)	25.3	75.0 (3/4)	24.4	69.0	37
441	RF00054 SNORD25	20.0 (1/5)	10.0	100.0 (1/1)	11.6	68.1	12
442	RF01178 snoR77Y	20.0 (1/5)	6.0	100.0 (1/1)	7.0	70.0	21
443	RF03017 RT-13	20.0 (4/20)	4.0	100.0 (4/4)	6.2	71.6	13
444	RF02974 Fibro-purF	20.0 (3/15)	2.7	100.0 (3/3)	4.0	81.8	12
445	RF00186 SNORD101	20.0 (1/5)	2.0	100.0 (1/1)	3.6	85.0	26
446	RF00350 snoZ152	20.0 (1/5)	2.0	100.0 (1/1)	4.0	72.1	9
447	RF02979 freshwater-1	20.0 (3/15)	2.0	75.0 (3/4)	4.1	75.4	8
448	RF00349 snoR11	20.0 (1/5)	0.0	100.0 (1/1)	1.8	86.0	5
449	RF03091 Clostridium-PBP	20.0 (1/5)	0.0	100.0 (1/1)	4.6	81.1	11
450	RF00187 SNORD102	20.0 (1/5)	0.0	100.0 (1/1)	1.8	88.2	11
451	RF01816 RsaA	19.5 (8/41)	1.7	100.0 (8/8)	4.1	66.3	7
452	RF00566 SNORA35	19.4 (6/31)	11.9	100.0 (6/6)	12.6	65.9	29
453	RF00638 MIR159	19.2 (5/26)	22.3	83.3 (5/6)	22.9	53.1	66
454	RF00022 GcvB	19.1 (9/47)	21.9	100.0 (9/9)	21.3	64.6	27
455	RF00239 mir-124	19.0 (4/21)	25.2	100.0 (4/4)	25.5	73.7	56
456	RF01989 SECIS 3	18.8 (3/16)	8.8	100.0 (3/3)	9.6	81.7	24
457	RF01767 SMK box riboswitch	18.8 (3/16)	6.2	100.0 (3/3)	6.9	72.3	25
458	RF02973 Enterococcus-1	18.2 (8/44)	10.0	100.0 (8/8)	11.4	78.5	78
459	RF01759 sucC	18.2 (2/11)	9.1	100.0 (2/2)	10.0	83.1	18
460	RF02902 AaHKsRNA22	18.2 (6/33)	3.0	100.0 (6/6)	4.6	67.3	7
461	RF00321 snoZ247	18.2 (2/11)	0.0	50.0 (2/4)	2.5	82.2	8
462	RF01714 Ocean-V	18.2 (2/11)	0.0	100.0 (2/2)	0.8	81.5	3
463	RF02030 tp2	17.9 (5/28)	12.5	100.0 (5/5)	12.9	64.5	24
464	RF00257 mir-194	17.9 (5/28)	11.4	100.0 (5/5)	11.4	69.8	29
465	RF00694 mir-137	17.6 (6/34)	17.1	100.0 (6/6)	16.4	63.2	28
466	RF00630 P26	17.6 (3/17)	8.8	100.0 (3/3)	9.6	70.4	27
467	RF00171 Tombus 5	17.6 (6/34)	2.6	100.0 (6/6)	4.0	76.2	9
468	RF00386 Entero 5 CRE	17.2 (5/29)	38.3	100.0 (5/5)	49.5	83.7	160
469	RF01065 23S-methyl	17.1 (6/35)	16.0	100.0 (6/6)	15.9	61.6	19
470	RF01736 flg-Rhizobiales	17.1 (6/35)	5.7	100.0 (6/6)	7.3	69.2	13
471	RF00185 Flavi CRE	16.7 (4/24)	42.1	100.0 (4/4)	42.3	65.9	84
472	RF01716 PhotoRC-I	16.7 (2/12)	23.3	100.0 (2/2)	24.3	81.8	65
473	RF00514 His leader	16.7 (6/36)	18.9	100.0 (6/6)	18.6	64.4	33
474	RF00147 SNORD34	16.7 (1/6)	16.7	100.0 (1/1)	16.2	69.9	14
475	RF00575 SNORD70	16.7 (2/12)	9.2	100.0 (2/2)	10.6	78.9	21
476	RF01410 BsrC	16.7 (2/12)	8.3	100.0 (2/2)	10.7	76.1	29
477	RF01723 Rhizobiales-2	16.7 (5/30)	7.7	100.0 (5/5)	8.7	78.0	15
478	RF00188 SNORD103	16.7 (1/6)	6.7	100.0 (1/1)	9.0	80.7	41
479	RF01315 CRISPR-DR2	16.7 (1/6)	3.3	100.0 (1/1)	4.5	78.0	19
480	RF03108 Methylosinus-1	16.7 (6/36)	2.5	100.0 (6/6)	5.2	77.0	12
481	RF00202 snoR66	16.7 (1/6)	1.7	100.0 (1/1)	2.8	87.3	6
482	RF01299 SNORD2	16.7 (1/6)	1.7	100.0 (1/1)	4.5	76.0	17
483	RF02877 NmsRb	16.7 (4/24)	1.2	100.0 (4/4)	3.6	76.0	6
484	RF01660 ceN88	16.7 (6/36)	0.6	100.0 (6/6)	1.3	88.6	6
485	RF02067 STnc310	16.7 (2/12)	0.0	100.0 (2/2)	1.4	90.1	8
486	RF00344 snoZ267	16.7 (1/6)	0.0	100.0 (1/1)	2.7	81.0	5
487	RF01854 Bacteria large SRP	16.3 (16/98)	78.8	100.0 (16/16)	103.2	75.3	92
488	RF00091 SNORA62	16.0 (4/25)	4.4	100.0 (4/4)	5.5	76.0	18
489	RF01733 atoC	15.8 (3/19)	1.6	100.0 (3/3)	4.4	65.4	8
490	RF01544 TB11Cs5H3	15.8 (3/19)	0.5	100.0 (3/3)	2.5	67.3	5
491	RF01263 snR191	15.6 (12/77)	4.0	100.0 (12/12)	6.1	68.7	11
492	RF00556 L19 leader	15.4 (2/13)	6.2	100.0 (2/2)	7.1	65.2	10
493	RF02887 mgtC leader	15.4 (2/13)	0.0	100.0 (2/2)	2.2	74.2	4

Continued on next page

	RNA family (seed alignment)	**Sensitivity** annotated bpairs that covary % (cov_bps/bps)	**Power** average power %	**Positive Predictive Value** covarying pairs in structure % (cov_bps/cov_pairs)	average substitutions per bpair	avg pairwise identity %	number of sequences
494	RF00396 SNORA13	15.0 (6/40)	27.5	100.0 (6/6)	27.4	67.7	50
495	RF00111 SdsR RyeB	15.0 (3/20)	7.5	100.0 (3/3)	8.4	75.6	17
496	RF00573 SNORD67	15.0 (3/20)	5.5	75.0 (3/4)	7.3	77.4	15
497	RF01720 Pseudomon-Rho	15.0 (3/20)	4.5	100.0 (3/3)	6.0	78.1	16
498	RF00695 MIR398	15.0 (3/20)	2.5	100.0 (3/3)	4.0	58.9	14
499	RF01710 Lacto-usp	15.0 (3/20)	2.5	100.0 (3/3)	4.8	65.9	6
500	RF02898 AaHKsRNA20	15.0 (3/20)	0.5	100.0 (3/3)	2.3	74.8	6
501	RF00641 mir-154	14.8 (4/27)	75.2	100.0 (4/4)	95.5	59.6	121
502	RF00018 CsrB	14.8 (8/54)	21.1	100.0 (8/8)	20.2	71.5	38
503	RF01070 sucA	14.8 (4/27)	15.9	100.0 (4/4)	15.9	71.2	35
504	RF01089 PK-repBA	14.8 (4/27)	3.3	100.0 (4/4)	5.0	62.9	7
505	RF00166 PrrB RsmZ	14.7 (5/34)	18.8	100.0 (5/5)	18.4	74.4	37
506	RF01752 psaA	14.5 (9/62)	20.2	90.0 (9/10)	19.2	75.8	31
507	RF00156 SNORA70	14.3 (3/21)	15.2	100.0 (3/3)	15.1	77.1	43
508	RF02348 tracrRNA	14.3 (1/7)	12.8	100.0 (1/1)	12.6	80.3	35
509	RF01755 rmf	14.3 (4/28)	11.4	100.0 (4/4)	11.7	72.0	16
510	RF00343 snoZ122	14.3 (1/7)	11.4	100.0 (1/1)	11.6	65.1	23
511	RF01225 ACA64	14.3 (4/28)	10.3	80.0 (4/5)	11.5	72.3	30
512	RF00191 SNORA57	14.3 (4/28)	7.8	100.0 (4/4)	9.1	78.8	26
513	RF00136 SNORD81	14.3 (1/7)	5.7	100.0 (1/1)	7.4	82.0	22
514	RF03033 NLPC-P60	14.3 (3/21)	5.2	100.0 (3/3)	7.5	70.2	17
515	RF02402 Afu 263	14.3 (2/14)	5.0	100.0 (2/2)	5.7	77.2	19
516	RF00444 PrrF	14.3 (4/28)	4.6	100.0 (4/4)	5.7	81.4	18
517	RF02978 folP	14.3 (4/28)	3.9	100.0 (4/4)	5.0	81.4	28
518	RF01737 flpD	14.3 (2/14)	3.6	100.0 (2/2)	6.5	68.8	8
519	RF03061 uxuA	14.3 (2/14)	3.6	100.0 (2/2)	6.4	77.5	33
520	RF01168 SNORD126	14.3 (1/7)	2.9	100.0 (1/1)	4.1	84.6	6
521	RF02922 RAGATH-32	14.3 (2/14)	2.1	100.0 (2/2)	4.5	81.5	19
522	RF00135 snoZ223	14.3 (1/7)	1.4	100.0 (1/1)	4.9	83.4	5
523	RF01287 snoR27	14.3 (1/7)	0.0	100.0 (1/1)	1.9	69.0	6
524	RF01186 SNORD127	14.3 (1/7)	0.0	100.0 (1/1)	0.3	86.6	13
525	RF00577 SNORD72	14.3 (1/7)	0.0	100.0 (1/1)	2.1	85.7	16
526	RF02732 JA04	14.3 (2/14)	0.0	100.0 (2/2)	1.5	85.5	6
527	RF01856 Protozoa SRP	13.8 (8/58)	31.4	100.0 (8/8)	28.7	45.4	18
528	RF01411 BsrF	13.8 (4/29)	4.8	100.0 (4/4)	7.0	74.1	11
529	RF00838 mir-252	13.8 (4/29)	3.4	100.0 (4/4)	5.9	54.4	10
530	RF00190 SNORA16	13.6 (3/22)	10.4	100.0 (3/3)	12.0	72.9	29
531	RF00075 mir-166	13.6 (3/22)	10.0	100.0 (3/3)	10.3	57.6	31
532	RF02347 ar45	13.6 (6/44)	8.4	100.0 (6/6)	9.5	85.9	36
533	RF00546 snopsi28S-3316	13.5 (5/37)	21.4	100.0 (5/5)	20.5	70.3	33
534	RF03098 RAGATH-22	13.5 (5/37)	2.4	100.0 (5/5)	4.9	80.5	18
535	RF01729 Termite-flg	13.3 (4/30)	4.3	100.0 (4/4)	6.2	68.4	13
536	RF02920 Fusobacteriales-1	13.3 (4/30)	1.3	100.0 (4/4)	3.8	71.7	13
537	RF02396 sau-5949	13.3 (2/15)	0.7	100.0 (2/2)	1.5	85.5	7
538	RF02838 Ref55	13.3 (2/15)	0.0	100.0 (2/2)	1.4	68.2	4
539	RF01742 lactis-plasmid	13.2 (5/38)	5.3	100.0 (5/5)	5.6	83.8	14
540	RF00229 IRES Picorna	13.1 (8/61)	25.6	100.0 (8/8)	29.6	83.5	92
541	RF00210 IRES Picorna 2	13.1 (16/122)	11.4	100.0 (16/16)	12.7	82.6	92
542	RF00028 Intron gpI	13.1 (8/61)	10.5	100.0 (8/8)	11.9	44.8	12
543	RF00553 SCARNA1	13.0 (6/46)	15.4	100.0 (6/6)	16.0	71.0	29
544	RF00238 ctRNA pND324	13.0 (3/23)	7.0	100.0 (3/3)	8.3	73.0	48
545	RF02985 ftsZ-DE	13.0 (3/23)	1.3	100.0 (3/3)	3.4	82.2	10
546	RF02983 Fibrobacter-1	13.0 (3/23)	0.4	75.0 (3/4)	1.4	74.8	4
547	RF00027 let-7	12.9 (4/31)	34.5	100.0 (4/4)	36.3	67.8	63
548	RF02553 YrlA	12.9 (4/31)	1.6	100.0 (4/4)	3.6	66.0	5
549	RF00430 SNORA54	12.9 (4/31)	0.6	100.0 (4/4)	2.3	60.2	5
550	RF00584 SNORD105	12.5 (1/8)	6.2	100.0 (1/1)	8.6	82.1	15
551	RF00283 SCARNA18	12.5 (1/8)	5.0	100.0 (1/1)	6.5	86.5	20
552	RF03023 rpfG	12.5 (2/16)	4.4	100.0 (2/2)	6.9	85.2	28
553	RF01482 AdoCbl riboswitch	12.5 (5/40)	3.8	83.3 (5/6)	6.0	64.5	7
554	RF02051 STnc450	12.5 (2/16)	1.9	100.0 (2/2)	3.5	77.2	12
555	RF03095 RAGATH-17	12.5 (2/16)	1.2	100.0 (2/2)	3.1	79.0	7
556	RF03109 Thermales-rpoB	12.5 (2/16)	0.6	50.0 (2/4)	3.1	87.4	14
557	RF00137 SNORD83	12.5 (1/8)	0.0	100.0 (1/1)	1.4	84.2	7
558	RF01074 RF site1	12.5 (1/8)	0.0	100.0 (1/1)	0.8	81.8	4
559	RF00524 R2 retro el	12.2 (5/41)	9.5	100.0 (5/5)	10.8	67.5	15
560	RF02500 Atu C6	12.0 (3/25)	11.6	75.0 (3/4)	12.3	68.3	32
561	RF00209 IRES Pesti	12.0 (9/75)	7.1	100.0 (9/9)	7.4	88.4	25
562	RF03038 nhaA-II	12.0 (3/25)	5.2	75.0 (3/4)	6.2	77.8	16
563	RF01751 potC	11.8 (2/17)	40.6	66.7 (2/3)	48.9	81.2	136
564	RF01845 enod40	11.8 (2/17)	8.8	100.0 (2/2)	11.1	55.8	35
565	RF03105 RAGATH-7-assoc	11.8 (4/34)	5.0	80.0 (4/5)	7.4	74.9	26
566	RF01248 snR8	11.8 (4/34)	4.1	80.0 (4/5)	5.7	69.2	11
567	RF00063 SscA	11.8 (2/17)	2.4	100.0 (2/2)	3.4	71.2	5
568	RF01537 TB11Cs2H1	11.8 (2/17)	0.6	100.0 (2/2)	2.0	73.7	7
569	RF01686 Acido-1	11.8 (2/17)	0.6	100.0 (2/2)	2.6	68.8	4
570	RF02362 Yfr10	11.8 (2/17)	0.0	100.0 (2/2)	1.5	72.6	6
571	RF01830 StyR-44	11.5 (3/26)	14.2	100.0 (3/3)	15.1	76.8	34
572	RF01591 snoR15	11.5 (3/26)	0.0	100.0 (3/3)	1.6	81.3	4
573	RF00706 mir-263	11.1 (3/27)	21.5	100.0 (3/3)	20.3	58.4	23
574	RF03031 ssnA	11.1 (1/9)	15.6	100.0 (1/1)	15.9	69.0	118
575	RF00377 snoU6-53	11.1 (1/9)	13.3	100.0 (1/1)	14.3	75.2	25
576	RF03115 KDPG-aldolase	11.1 (2/18)	6.7	100.0 (2/2)	8.6	73.8	43
577	RF00271 SNORD60	11.1 (1/9)	3.3	50.0 (1/2)	4.8	79.6	22

Continued on next page

	RNA family (seed alignment)	Sensitivity annotated bpairs that covary % (cov_bps/bps)	Power average power %	Positive Predictive Value covarying pairs in structure % (cov_bps/cov_pairs)	average substitutions per bpair	avg pairwise identity %	number of sequences
578	RF01322 CRISPR-DR9	11.1 (1/9)	3.3	100.0 (1/1)	4.3	77.7	7
579	RF02971 emrB-Lactobacillus	11.1 (2/18)	2.8	100.0 (2/2)	5.2	82.9	39
580	RF01744 livK	11.1 (3/27)	2.2	100.0 (3/3)	3.6	69.1	8
581	RF00359 snoZ102 R77	11.1 (1/9)	2.2	100.0 (1/1)	3.3	87.2	9
582	RF00369 sroC	11.1 (4/36)	1.7	100.0 (4/4)	2.9	75.0	6
583	RF01713 Moco-II	11.1 (1/9)	1.1	100.0 (1/1)	3.1	70.2	7
584	RF03035 nqrA-Marinomonas	11.1 (4/36)	0.8	100.0 (4/4)	2.6	72.3	5
585	RF02595 EBv-sisRNA-1	11.1 (1/9)	0.0	100.0 (1/1)	1.1	85.8	8
586	RF01258 snR10	10.9 (7/64)	7.3	100.0 (7/7)	8.2	74.1	15
587	RF00021 Spot 42	10.8 (4/37)	3.2	100.0 (4/4)	4.6	79.3	19
588	RF03089 cold-seep-1	10.8 (4/37)	2.4	66.7 (4/6)	4.9	71.8	15
589	RF00411 SNORA9	10.7 (3/28)	10.7	100.0 (3/3)	11.8	77.6	36
590	RF00996 mir-631	10.7 (3/28)	1.4	100.0 (3/3)	2.9	71.0	7
591	RF03090 lysM-Actino	10.6 (5/47)	6.6	100.0 (5/5)	8.1	79.4	56
592	RF01412 BsrG	10.6 (7/66)	2.0	100.0 (7/7)	5.0	53.8	6
593	RF00782 MIR480	10.5 (2/19)	54.7	100.0 (2/2)	57.1	68.3	69
594	RF01694 Bacteroides-1	10.5 (2/19)	17.9	100.0 (2/2)	17.8	75.9	40
595	RF01497 ALIL	10.5 (2/19)	7.4	100.0 (2/2)	8.0	67.8	26
596	RF02544 mt-tmRNA	10.5 (2/19)	1.6	100.0 (2/2)	3.5	79.0	11
597	RF02910 Coronavirus 5p sl 1 2	10.5 (2/19)	0.0	100.0 (2/2)	1.5	73.8	4
598	RF00757 mir-275	10.3 (3/29)	5.9	100.0 (3/3)	6.9	68.5	15
599	RF00045 SNORA73	10.0 (5/50)	33.4	100.0 (5/5)	32.9	72.9	66
600	RF01738 gabT	10.0 (2/20)	12.5	100.0 (2/2)	12.4	67.9	17
601	RF01191 SNORD121A	10.0 (1/10)	9.0	100.0 (1/1)	9.9	80.9	26
602	RF03112 Staphylococcus-1	10.0 (4/40)	8.5	100.0 (4/4)	9.2	75.1	21
603	RF00079 OmrA-B	10.0 (2/20)	7.5	100.0 (2/2)	7.8	76.6	23
604	RF00200 snoZ199	10.0 (1/10)	7.0	100.0 (1/1)	8.7	82.4	17
605	RF01776 RatA	10.0 (1/10)	6.0	100.0 (1/1)	6.7	67.8	16
606	RF02523 ROSE 3	10.0 (2/20)	5.0	100.0 (2/2)	5.6	71.4	16
607	RF00831 mir-73	10.0 (3/30)	2.0	100.0 (3/3)	3.2	68.6	7
608	RF01535 TB10Cs5H2	10.0 (1/10)	1.0	100.0 (1/1)	2.3	73.2	5
609	RF01774 rpsL ricks	10.0 (3/30)	0.3	100.0 (3/3)	1.7	87.4	7
610	RF00436 UnaL2	10.0 (1/10)	0.0	100.0 (1/1)	1.8	76.8	30
611	RF02889 Sr006	10.0 (3/30)	0.0	75.0 (3/4)	1.2	64.9	3
612	RF01700 Collinsella-1	9.6 (5/52)	2.9	71.4 (5/7)	4.8	71.3	6
613	RF00667 mir-33	9.5 (2/21)	17.1	100.0 (2/2)	17.7	69.5	50
614	RF00513 Trp leader	9.5 (2/21)	13.3	100.0 (2/2)	13.9	60.0	22
615	RF01684 mascRNA-menRNA	9.5 (2/21)	10.5	100.0 (2/2)	11.0	79.1	53
616	RF03113 Poribacteria-1	9.5 (2/21)	1.4	100.0 (2/2)	3.3	68.0	6
617	RF00161 nos TCE	9.5 (2/21)	1.4	100.0 (2/2)	2.4	80.3	9
618	RF01698 Chloroflexi-1	9.5 (2/21)	0.0	100.0 (2/2)	0.8	82.1	3
619	RF02357 RNaseP-T	9.4 (5/53)	1.5	100.0 (5/5)	3.3	82.0	9
620	RF02414 rli60	9.3 (4/43)	4.9	100.0 (4/4)	6.9	63.7	11
621	RF02410 snoR136	9.3 (4/43)	3.2	100.0 (4/4)	5.2	63.6	8
622	RF02452 ncr1575	9.2 (5/54)	8.3	100.0 (5/5)	9.1	71.2	29
623	RF00175 HIV-1 DIS	9.1 (1/11)	7.3	100.0 (1/1)	8.5	87.1	70
624	RF01415 Flavivirus SLIV	9.1 (2/22)	5.0	100.0 (2/2)	5.8	80.5	20
625	RF03056 RAGATH-35	9.1 (1/11)	1.8	100.0 (1/1)	4.4	78.2	16
626	RF01508 BYDV 5 UTR	9.1 (3/33)	1.2	100.0 (3/3)	2.8	82.3	4
627	RF01257 snR31	9.1 (5/55)	0.7	100.0 (5/5)	2.6	74.0	8
628	RF01823 rspL	9.1 (2/22)	0.5	100.0 (2/2)	1.1	88.7	6
629	RF02860 Arrc14	9.1 (4/44)	0.0	100.0 (4/4)	1.5	72.9	4
630	RF00516 ylbH	9.1 (3/33)	0.0	100.0 (3/3)	2.6	55.7	3
631	RF01080 UPD-PKib	9.1 (1/11)	0.0	100.0 (1/1)	1.9	83.3	4
632	RF00365 mir-BHRF1-1	9.1 (2/22)	0.0	100.0 (2/2)	0.8	85.7	5
633	RF02909 rli117	9.0 (7/78)	0.1	100.0 (7/7)	1.7	59.4	3
634	RF00102 VA	8.9 (4/45)	30.0	100.0 (4/4)	31.0	70.2	54
635	RF02346 ar35	8.9 (4/45)	3.8	100.0 (4/4)	5.3	72.3	13
636	RF01296 snoU85	8.8 (5/57)	24.2	100.0 (5/5)	22.8	63.6	21
637	RF02520 mir-965	8.8 (3/34)	19.1	100.0 (3/3)	19.0	64.6	32
638	RF00754 mir-279	8.8 (3/34)	5.6	100.0 (3/3)	7.5	66.5	12
639	RF01796 frnS	8.8 (3/34)	5.0	100.0 (3/3)	6.0	77.8	16
640	RF03096 RAGATH-19	8.8 (3/34)	3.2	100.0 (3/3)	5.9	67.7	11
641	RF00019 Y RNA	8.7 (2/23)	28.7	66.7 (2/3)	29.1	65.4	104
642	RF00648 MIR396	8.7 (2/23)	13.0	100.0 (2/2)	12.7	58.6	26
643	RF02981 FTHFS	8.7 (2/23)	5.7	100.0 (2/2)	8.7	72.9	27
644	RF00291 snoR639	8.7 (2/23)	0.9	100.0 (2/2)	2.5	80.5	9
645	RF01292 snoR2	8.6 (3/35)	3.4	100.0 (3/3)	4.9	73.7	11
646	RF02076 STnc100	8.5 (4/47)	13.0	100.0 (4/4)	13.5	62.4	24
647	RF01726 SAM-II long loops	8.3 (1/12)	20.0	50.0 (1/2)	27.0	75.3	126
648	RF02354 BjrC80	8.3 (4/48)	9.2	80.0 (4/5)	10.2	67.4	15
649	RF00113 SIB RNA	8.3 (2/24)	4.6	100.0 (2/2)	4.7	72.7	17
650	RF00713 mir-239	8.3 (2/24)	3.8	100.0 (2/2)	4.8	59.0	9
651	RF00543 snopsi18S-1377	8.3 (2/24)	2.5	100.0 (2/2)	3.9	72.5	15
652	RF01536 TB10Cs5H3	8.3 (1/12)	1.7	100.0 (1/1)	3.7	68.6	7
653	RF01504 Afu 254	8.3 (1/12)	1.7	50.0 (1/2)	3.8	55.9	6
654	RF00954 MIR1446	8.3 (3/36)	1.7	75.0 (3/4)	3.5	69.7	7
655	RF02945 Corio-PBP	8.3 (2/24)	1.2	100.0 (2/2)	2.3	86.8	14
656	RF01838 sobemo FSE	8.3 (1/12)	0.8	100.0 (1/1)	1.4	68.2	5
657	RF00172 CAESAR	8.3 (1/12)	0.8	100.0 (1/1)	2.2	79.4	9
658	RF02796 Pab160	8.3 (2/24)	0.0	100.0 (2/2)	1.1	86.2	4
659	RF01855 Plant SRP	8.1 (10/123)	51.7	100.0 (10/10)	55.0	64.6	63
660	RF01470 rli38	8.1 (3/37)	17.3	100.0 (3/3)	17.3	63.2	20
661	RF01610 ceN104	8.1 (3/37)	0.8	75.0 (3/4)	2.8	68.3	5

Continued on next page

	RNA family (seed alignment)	Sensitivity annotated bpairs that covary % (cov_bps/bps)	Power average power %	Positive Predictive Value covarying pairs in structure % (cov_bps/cov_pairs)	average substitutions per bpair	avg pairwise identity %	number of sequences
662	RF00378 Qrr	8.0 (2/25)	8.4	100.0 (2/2)	8.7	80.2	30
663	RF00813 mir-11	8.0 (2/25)	4.8	100.0 (2/2)	6.2	70.6	15
664	RF00051 mir-17	8.0 (2/25)	2.0	100.0 (2/2)	3.6	70.1	54
665	RF01047 HBV epsilon	8.0 (2/25)	2.0	100.0 (2/2)	2.4	97.0	32
666	RF02364 Yfr13	8.0 (2/25)	0.8	100.0 (2/2)	2.0	82.6	7
667	RF03107 saliva-tongue-1	8.0 (2/25)	0.4	100.0 (2/2)	2.9	77.8	10
668	RF01758 sucA-II	7.9 (5/63)	8.1	100.0 (5/5)	9.0	73.4	17
669	RF02263 MAT2A D	7.7 (1/13)	18.5	100.0 (1/1)	18.6	78.1	35
670	RF02934 caiA	7.7 (1/13)	4.6	100.0 (1/1)	7.0	73.0	14
671	RF00947 mir-929	7.7 (2/26)	3.5	66.7 (2/3)	3.8	74.3	15
672	RF03094 LAGLIDADG-2	7.7 (3/39)	2.8	75.0 (3/4)	4.6	82.9	25
673	RF00488 U1 yeast	7.7 (6/78)	1.4	100.0 (6/6)	3.4	63.1	5
674	RF02343 ar9	7.5 (3/40)	8.5	100.0 (3/3)	8.1	76.6	28
675	RF01254 snR34	7.5 (3/40)	0.0	100.0 (3/3)	0.7	89.8	4
676	RF01740 gyrA	7.4 (2/27)	15.9	100.0 (2/2)	15.7	64.5	18
677	RF02037 IMES-4	7.4 (2/27)	9.6	100.0 (2/2)	10.2	87.3	30
678	RF00722 mir-451	7.4 (2/27)	7.0	100.0 (2/2)	7.3	77.7	19
679	RF00637 mir-276	7.4 (2/27)	3.3	100.0 (2/2)	3.4	73.1	16
680	RF00437 HLE	7.4 (2/27)	1.1	100.0 (2/2)	2.6	83.1	5
681	RF02384 FasX	7.3 (5/68)	2.2	100.0 (5/5)	3.9	74.2	8
682	RF02411 snoR138	7.3 (3/41)	1.2	100.0 (3/3)	3.5	68.4	7
683	RF02809 RsmW	7.3 (3/41)	0.0	100.0 (3/3)	1.6	57.0	3
684	RF00199 SL2	7.1 (2/28)	17.1	100.0 (2/2)	16.6	76.9	30
685	RF00260 HepC CRE	7.1 (1/14)	10.7	100.0 (1/1)	10.3	86.9	52
686	RF00078 MicA	7.1 (1/14)	5.7	100.0 (1/1)	5.9	82.1	17
687	RF01722 Pyrobac-1	7.1 (1/14)	5.0	100.0 (1/1)	5.8	65.4	8
688	RF02807 PyrR209	7.1 (1/14)	0.7	100.0 (1/1)	2.1	74.7	4
689	RF01539 TB11Cs4H1	7.1 (1/14)	0.7	100.0 (1/1)	1.6	77.2	5
690	RF00823 lsy-6	7.1 (2/28)	0.3	100.0 (2/2)	1.2	77.3	4
691	RF01643 ceN56	7.1 (2/28)	0.0	100.0 (2/2)	0.9	83.9	4
692	RF02803 PyrR205	7.1 (1/14)	0.0	100.0 (1/1)	0.7	87.3	5
693	RF00370 sroD	7.1 (1/14)	0.0	100.0 (1/1)	0.4	85.4	5
694	RF00545 snopsi18S-841	7.0 (3/43)	15.8	100.0 (3/3)	15.3	69.0	24
695	RF01756 rne-II	6.9 (5/72)	7.1	100.0 (5/5)	7.4	75.6	16
696	RF01809 symR	6.9 (2/29)	3.8	100.0 (2/2)	4.8	78.0	20
697	RF00422 SCARNA24	6.9 (2/29)	3.1	100.0 (2/2)	5.0	83.1	23
698	RF03043 Prevotella-2	6.9 (2/29)	2.1	100.0 (2/2)	5.2	74.5	20
699	RF00014 DsrA	6.9 (2/29)	0.7	100.0 (2/2)	2.1	83.3	5
700	RF00949 mir-983	6.9 (2/29)	0.0	100.0 (2/2)	0.9	77.2	3
701	RF01247 snR32	6.8 (3/44)	4.8	100.0 (3/3)	6.8	66.9	11
702	RF00534 SgrS	6.8 (4/59)	4.1	100.0 (4/4)	7.3	48.0	8
703	RF01782 ASpks	6.7 (1/15)	25.3	100.0 (1/1)	25.6	78.5	77
704	RF00112 CyaR RyeE	6.7 (1/15)	6.0	100.0 (1/1)	6.3	77.6	17
705	RF01213 snoR103	6.7 (1/15)	3.3	100.0 (1/1)	4.3	66.8	11
706	RF00170 msr	6.7 (1/15)	1.3	100.0 (1/1)	4.1	63.0	9
707	RF02437 SpF44 sRNA	6.7 (2/30)	1.3	100.0 (2/2)	3.1	83.7	12
708	RF01557 TB9Cs4H1	6.7 (1/15)	0.7	100.0 (1/1)	1.8	72.2	4
709	RF01251 snR3	6.7 (3/45)	0.2	100.0 (3/3)	1.1	86.8	4
710	RF02806 PyrR208	6.7 (1/15)	0.0	100.0 (1/1)	1.1	72.4	4
711	RF01073 GP knot1	6.7 (1/15)	0.0	100.0 (1/1)	1.9	76.6	7
712	RF00341 snoZ39	6.7 (1/15)	0.0	100.0 (1/1)	0.7	88.4	5
713	RF01548 TB6Cs1H4	6.7 (1/15)	0.0	100.0 (1/1)	1.4	79.2	4
714	RF01554 TB9Cs2H1	6.7 (1/15)	0.0	100.0 (1/1)	0.5	84.6	4
715	RF00094 HDV ribozyme	6.5 (2/31)	7.7	100.0 (2/2)	8.8	74.0	33
716	RF00789 mir-286	6.5 (2/31)	7.4	100.0 (2/2)	7.8	69.2	14
717	RF00540 snopsi18S-1854	6.5 (2/31)	3.2	100.0 (2/2)	4.1	78.5	16
718	RF01608 ceN102	6.5 (2/31)	0.3	100.0 (2/2)	1.0	75.3	4
719	RF00820 mir-248	6.5 (2/31)	0.3	100.0 (2/2)	1.4	70.0	4
720	RF00810 mir-85	6.5 (2/31)	0.3	100.0 (2/2)	1.3	67.3	5
721	RF02905 sRNA41	6.5 (2/31)	0.0	100.0 (2/2)	1.7	67.3	3
722	RF02997 HOLDH	6.3 (6/95)	2.3	100.0 (6/6)	4.6	74.2	13
723	RF02502 Atu C8	6.2 (2/32)	11.6	66.7 (2/3)	12.0	69.1	27
724	RF01456 VrrA	6.2 (2/32)	4.7	100.0 (2/2)	6.3	86.2	11
725	RF00720 mir-317	6.2 (2/32)	3.8	100.0 (2/2)	5.3	72.2	12
726	RF03011 Methylophilales-1	6.2 (2/32)	3.4	100.0 (2/2)	4.5	86.0	14
727	RF01835 HIV FS2	6.2 (1/16)	3.1	100.0 (1/1)	4.2	86.4	19
728	RF00374 Gammaretro CES	6.2 (2/32)	2.2	100.0 (2/2)	2.9	89.3	22
729	RF01555 TB9Cs3H1	6.2 (1/16)	1.9	100.0 (1/1)	3.5	69.8	6
730	RF00624 P9	6.2 (1/16)	1.9	100.0 (1/1)	2.4	77.6	17
731	RF02846 Ref84	6.2 (1/16)	0.6	100.0 (1/1)	2.3	81.0	5
732	RF01802 HSUR	6.2 (1/16)	0.6	100.0 (1/1)	2.8	74.2	4
733	RF02716 sno ncR3	6.2 (1/16)	0.6	100.0 (1/1)	1.9	82.1	5
734	RF01532 TB10Cs3H2	6.2 (1/16)	0.6	100.0 (1/1)	1.8	77.8	6
735	RF02790 sodF sRNA	6.2 (2/32)	0.3	100.0 (2/2)	1.5	82.8	6
736	RF02848 RefB11	6.2 (3/48)	0.2	100.0 (3/3)	2.0	62.8	4
737	RF00800 mir-353	6.2 (2/32)	0.0	100.0 (2/2)	1.4	77.6	4
738	RF00033 MicF	6.2 (1/16)	0.0	100.0 (1/1)	1.9	84.0	4
739	RF01489 sbrA	6.2 (1/16)	0.0	100.0 (1/1)	0.9	91.3	6
740	RF02096 mir-2973	6.2 (2/32)	0.0	100.0 (2/2)	1.5	80.6	7
741	RF00797 mir-355	6.2 (2/32)	0.0	100.0 (2/2)	1.0	72.4	4
742	RF01227 snoR83	6.1 (2/33)	1.8	66.7 (2/3)	3.2	72.6	7
743	RF03104 RAGATH-36	6.1 (2/33)	1.5	100.0 (2/2)	4.2	80.2	14
744	RF03088 Parabacteroides-1	6.1 (2/33)	1.5	100.0 (2/2)	3.9	78.1	15
745	RF02834 VqmR	6.1 (2/33)	0.3	100.0 (2/2)	1.6	72.4	4

Continued on next page

	RNA family (seed alignment)	Sensitivity annotated bpairs that covary % (cov_bps/bps)	Power average power %	Positive Predictive Value covarying pairs in structure % (cov_bps/cov_pairs)	average substitutions per bpair	avg pairwise identity %	number of sequences
746	RF00083 GlmZ SraJ	6.0 (3/50)	3.6	100.0 (3/3)	4.0	79.0	21
747	RF01715 Pedo-repair	5.9 (4/68)	0.6	100.0 (4/4)	2.2	79.2	6
748	RF01046 WLE3	5.9 (1/17)	0.6	100.0 (1/1)	2.4	84.3	10
749	RF03102 RAGATH-33	5.9 (1/17)	0.6	100.0 (1/1)	2.6	82.5	7
750	RF01543 TB11Cs5H2	5.9 (1/17)	0.6	100.0 (1/1)	1.8	71.7	5
751	RF00794 mir-42	5.9 (2/34)	0.3	100.0 (2/2)	1.1	78.2	5
752	RF02900 AaHKsRNA82	5.9 (1/17)	0.0	100.0 (1/1)	1.9	66.8	4
753	RF02431 SpF22 sRNA	5.7 (2/35)	7.4	100.0 (2/2)	8.4	75.3	22
754	RF02413 snoR145	5.7 (2/35)	6.3	100.0 (2/2)	7.4	69.1	19
755	RF02469 Ms IGR-7	5.7 (2/35)	3.1	100.0 (2/2)	5.0	72.5	14
756	RF00263 SNORA68	5.5 (2/36)	15.3	100.0 (2/2)	15.6	70.9	26
757	RF00668 mir-302	5.5 (1/18)	14.4	100.0 (1/1)	14.9	72.7	44
758	RF00643 MIR171 1	5.5 (1/18)	12.2	100.0 (1/1)	14.0	59.8	57
759	RF01696 Chlorobi-1	5.5 (1/18)	7.8	100.0 (1/1)	9.1	74.6	15
760	RF02919 ilvB-OMG	5.5 (3/55)	7.3	100.0 (3/3)	8.1	79.5	28
761	RF00591 SNORD77	5.5 (1/18)	6.7	100.0 (1/1)	8.0	82.4	21
762	RF02509 PYLIS 2	5.5 (1/18)	5.5	100.0 (1/1)	6.5	75.1	23
763	RF02144 rsmX	5.5 (2/36)	4.7	66.7 (2/3)	6.1	79.0	16
764	RF01295 SCARNA7	5.5 (1/18)	2.8	100.0 (1/1)	5.0	81.3	18
765	RF00165 Corona pk3	5.5 (1/18)	2.2	100.0 (1/1)	4.9	69.3	14
766	RF02349 psRNA2	5.5 (3/55)	2.2	100.0 (3/3)	4.1	64.0	7
767	RF02266 XIST intron	5.5 (1/18)	2.2	100.0 (1/1)	2.7	91.3	20
768	RF02275 Hammerhead HH9	5.5 (1/18)	1.7	100.0 (1/1)	2.4	89.3	33
769	RF00520 ybhL	5.5 (1/18)	1.1	100.0 (1/1)	2.8	74.5	6
770	RF00688 MIR394	5.5 (1/18)	1.1	100.0 (1/1)	1.6	70.7	19
771	RF00481 HCV X3	5.5 (2/36)	1.1	100.0 (2/2)	1.7	95.8	22
772	RF01768 flavi FSE	5.5 (1/18)	0.6	100.0 (1/1)	1.6	87.0	7
773	RF01620 ceN125	5.5 (2/36)	0.6	50.0 (2/4)	1.2	82.6	4
774	RF01430 snoR134	5.5 (2/36)	0.3	100.0 (2/2)	1.8	68.3	5
775	RF00795 mir-43	5.5 (2/36)	0.0	100.0 (2/2)	0.9	73.2	4
776	RF00293 snoM1	5.5 (1/18)	0.0	100.0 (1/1)	1.4	89.1	7
777	RF00389 satBaMV CRE	5.3 (2/38)	6.3	100.0 (2/2)	6.5	93.1	42
778	RF00303 snoR86	5.3 (1/19)	3.7	100.0 (1/1)	4.3	76.6	11
779	RF02353 BjrC68	5.3 (1/19)	3.7	100.0 (1/1)	4.8	80.7	12
780	RF03101 RAGATH-31	5.3 (1/19)	1.6	100.0 (1/1)	2.7	87.8	26
781	RF01807 GIR1	5.3 (3/56)	1.6	100.0 (3/3)	3.8	76.5	12
782	RF00138 SNORD16	5.3 (1/19)	1.1	100.0 (1/1)	2.2	79.5	5
783	RF03004 Lacto-phage-1	5.3 (1/19)	0.5	100.0 (1/1)	2.6	77.3	9
784	RF02823 LPR69	5.3 (1/19)	0.5	100.0 (1/1)	2.3	75.8	5
785	RF02530 URE2 IRES	5.3 (1/19)	0.0	100.0 (1/1)	1.2	86.7	5
786	RF00056 SNORA71	5.1 (2/39)	13.6	100.0 (2/2)	14.6	78.6	40
787	RF00791 mir-357	5.1 (2/39)	1.0	100.0 (2/2)	2.9	66.7	5
788	RF02029 sraA	5.0 (1/20)	5.5	50.0 (1/2)	7.0	73.9	20
789	RF02269 HPnc0580	5.0 (1/20)	0.5	100.0 (1/1)	2.2	85.5	7
790	RF02681 Twister-sister	5.0 (1/20)	0.5	100.0 (1/1)	1.4	78.4	4
791	RF00906 MIR1122	4.9 (2/41)	2.2	100.0 (2/2)	3.7	71.6	12
792	RF01612 ceN105	4.9 (2/41)	0.2	100.0 (2/2)	1.6	66.4	3
793	RF00657 mir-184	4.8 (1/21)	6.2	100.0 (1/1)	6.8	68.4	32
794	RF01252 snR5	4.8 (2/42)	1.9	100.0 (2/2)	4.1	76.2	11
795	RF00220 Rhino CRE	4.8 (1/21)	1.9	50.0 (1/2)	3.8	81.4	12
796	RF00043 Plasmid R1162	4.8 (1/21)	1.4	100.0 (1/1)	2.4	75.9	8
797	RF01819 RsaD	4.8 (2/42)	1.2	100.0 (2/2)	2.1	84.7	8
798	RF01815 rpsB	4.8 (1/21)	0.5	100.0 (1/1)	3.7	41.4	4
799	RF02832 Scr5676	4.8 (2/42)	0.2	100.0 (2/2)	1.0	86.5	5
800	RF01014 mir-1306	4.8 (1/21)	0.0	100.0 (1/1)	0.7	89.5	8
801	RF00512 Leu leader	4.7 (2/43)	1.2	100.0 (2/2)	3.4	64.1	6
802	RF00647 MIR164	4.5 (1/22)	9.5	100.0 (1/1)	10.4	61.1	39
803	RF00407 SNORA50	4.5 (1/22)	3.2	100.0 (1/1)	5.5	72.0	14
804	RF01719 Pseudomon-1	4.5 (1/22)	3.2	100.0 (1/1)	4.4	89.0	19
805	RF00052 lin-4	4.5 (1/22)	2.7	100.0 (1/1)	4.4	69.1	12
806	RF00518 speF	4.5 (1/22)	1.8	100.0 (1/1)	3.7	59.3	6
807	RF02464 Ms AS-4	4.5 (1/22)	0.9	100.0 (1/1)	3.0	79.1	9
808	RF02885 SAM VI	4.5 (1/22)	0.9	100.0 (1/1)	2.0	85.5	7
809	RF01077 TLS-PK2	4.5 (1/22)	0.5	100.0 (1/1)	1.0	82.8	4
810	RF01020 mir-572	4.5 (1/22)	0.0	100.0 (1/1)	0.1	90.2	4
811	RF02772 AilA thermometer	4.5 (1/22)	0.0	100.0 (1/1)	0.7	84.9	5
812	RF01413 miR-430	4.3 (1/23)	22.2	100.0 (1/1)	21.5	59.8	46
813	RF02935 che1	4.3 (1/23)	16.9	100.0 (1/1)	16.5	82.1	98
814	RF00103 mir-1	4.3 (1/23)	16.5	100.0 (1/1)	16.9	68.6	66
815	RF00640 MIR167 1	4.3 (1/23)	15.7	100.0 (1/1)	15.7	57.5	42
816	RF00649 mir-128	4.3 (1/23)	3.9	100.0 (1/1)	4.5	73.3	18
817	RF02688 RAGATH-13	4.3 (1/23)	2.2	50.0 (1/2)	4.2	74.1	20
818	RF01042 mir-891	4.3 (1/23)	0.4	100.0 (1/1)	1.3	84.6	7
819	RF02989 gntR-DTE	4.3 (1/23)	0.4	100.0 (1/1)	2.5	75.0	7
820	RF01246 snR81	4.3 (2/46)	0.0	100.0 (2/2)	0.2	88.5	3
821	RF00250 mir-TAR	4.2 (1/24)	10.8	100.0 (1/1)	12.7	84.7	45
822	RF00420 SNORA61	4.2 (1/24)	6.2	100.0 (1/1)	8.2	73.8	25
823	RF00724 mir-282	4.2 (1/24)	2.5	100.0 (1/1)	3.5	67.9	15
824	RF01224 snoR80	4.2 (1/24)	2.5	50.0 (1/2)	3.6	70.9	9
825	RF00073 mir-156	4.2 (1/24)	1.7	100.0 (1/1)	2.2	73.1	10
826	RF01260 snR11	4.2 (3/71)	0.6	100.0 (3/3)	2.8	76.4	6
827	RF01239 snR49	4.2 (2/47)	0.6	100.0 (2/2)	2.3	76.0	6
828	RF02869 ncS025	4.2 (1/24)	0.0	100.0 (1/1)	0.5	93.5	4
829	RF02082 STnc540	4.2 (2/47)	0.0	100.0 (2/2)	0.4	90.9	3

Continued on next page

	RNA family (seed alignment)	Sensitivity annotated bpairs that covary % (cov_bps/bps)	Power average power %	Positive Predictive Value covarying pairs in structure % (cov_bps/cov_pairs)	average substitutions per bpair	avg pairwise identity %	number of sequences
830	RF00115 McaS	4.2 (1/24)	0.0	50.0 (1/2)	1.1	83.7	4
831	RF01808 MicX	4.1 (2/49)	1.6	100.0 (2/2)	3.3	74.7	10
832	RF00061 IRES HCV	4.0 (3/75)	33.6	100.0 (3/3)	37.0	87.3	79
833	RF00042 CopA	4.0 (1/25)	7.2	100.0 (1/1)	7.1	80.4	37
834	RF01899 mir-2241	4.0 (1/25)	0.8	100.0 (1/1)	2.7	73.9	10
835	RF00846 mir-64	4.0 (1/25)	0.4	100.0 (1/1)	1.5	58.1	4
836	RF00830 mir-74	4.0 (1/25)	0.0	100.0 (1/1)	1.0	66.4	4
837	RF01242 snR36	3.9 (2/51)	3.1	100.0 (2/2)	4.9	70.4	10
838	RF02842 RefA1	3.9 (2/51)	0.0	100.0 (2/2)	0.9	91.2	4
839	RF01769 greA	3.8 (1/26)	9.2	100.0 (1/1)	10.2	72.8	25
840	RF00413 SNORA19	3.8 (1/26)	6.5	100.0 (1/1)	7.6	79.0	34
841	RF00655 mir-28	3.8 (1/26)	5.4	100.0 (1/1)	6.5	77.0	29
842	RF00716 mir-3	3.8 (1/26)	3.8	50.0 (1/2)	5.4	70.0	11
843	RF00248 mir-148	3.8 (1/26)	0.8	100.0 (1/1)	1.8	72.5	5
844	RF00750 mir-458	3.8 (1/26)	0.4	100.0 (1/1)	1.2	81.0	7
845	RF00890 mir-668	3.8 (1/26)	0.4	100.0 (1/1)	1.4	89.2	6
846	RF02489 GlsR26	3.8 (1/26)	0.0	100.0 (1/1)	1.0	84.0	3
847	RF02725 sno ZL8	3.8 (1/26)	0.0	100.0 (1/1)	0.7	91.0	6
848	RF00392 SNORA5	3.8 (1/26)	0.0	100.0 (1/1)	1.6	75.8	6
849	RF00225 IRES Tobamo	3.8 (1/26)	0.0	100.0 (1/1)	0.9	87.5	7
850	RF01675 CrcZ	3.7 (2/54)	9.4	100.0 (2/2)	9.7	68.5	19
851	RF00403 SNORA41	3.7 (1/27)	9.2	100.0 (1/1)	10.3	77.7	31
852	RF00510 Tombus IRE	3.7 (1/27)	7.8	100.0 (1/1)	9.3	84.3	23
853	RF01255 snR35	3.7 (2/54)	4.1	100.0 (2/2)	5.7	76.4	11
854	RF02370 Trp leader 2	3.7 (1/27)	1.9	100.0 (1/1)	3.5	75.4	9
855	RF00062 HgcC	3.7 (1/27)	1.5	25.0 (1/4)	3.3	69.7	5
856	RF00256 mir-196	3.7 (1/27)	1.5	100.0 (1/1)	3.4	73.7	14
857	RF00725 mir-iab-4	3.7 (1/27)	0.7	100.0 (1/1)	1.6	85.0	8
858	RF02378 SurC	3.7 (2/54)	0.6	100.0 (2/2)	1.4	85.3	4
859	RF02366 Yfr19	3.7 (1/27)	0.0	100.0 (1/1)	0.7	86.8	6
860	RF01919 mir-1419	3.7 (1/27)	0.0	100.0 (1/1)	1.1	79.3	5
861	RF01492 rli28	3.6 (1/28)	18.9	100.0 (1/1)	18.9	55.5	21
862	RF00130 mir-192	3.6 (1/28)	11.1	100.0 (1/1)	11.7	68.0	41
863	RF00950 mir-927	3.6 (1/28)	5.3	100.0 (1/1)	5.7	77.2	14
864	RF00286 SCARNA8	3.6 (1/28)	3.9	100.0 (1/1)	5.8	79.5	22
865	RF01233 snoU109	3.6 (1/28)	3.6	100.0 (1/1)	4.9	83.2	26
866	RF00717 mir-315	3.6 (1/28)	2.9	50.0 (1/2)	4.6	75.9	16
867	RF01850 beta tmRNA	3.6 (2/55)	2.5	100.0 (2/2)	4.8	71.1	7
868	RF00418 SNORA58	3.6 (1/28)	2.5	100.0 (1/1)	4.3	80.9	20
869	RF00082 SraG	3.6 (1/28)	2.1	100.0 (1/1)	2.9	71.7	7
870	RF01039 mir-937	3.6 (1/28)	1.1	100.0 (1/1)	3.0	73.8	9
871	RF02547 mtPerm-5S	3.6 (1/28)	0.3	100.0 (1/1)	1.3	84.4	6
872	RF01087 PK-repZ	3.6 (1/28)	0.3	100.0 (1/1)	1.1	89.5	6
873	RF01788 drz-agam-2-2	3.6 (2/56)	0.2	100.0 (2/2)	1.6	71.8	5
874	RF00927 mir-582	3.6 (1/28)	0.0	100.0 (1/1)	0.4	91.6	7
875	RF00064 HgcG	3.5 (2/57)	0.2	66.7 (2/3)	2.2	68.4	5
876	RF00434 BTE	3.4 (1/29)	12.4	100.0 (1/1)	12.4	72.9	17
877	RF01496 Afu 182	3.4 (1/29)	8.6	100.0 (1/1)	9.5	65.3	19
878	RF00842 MIR403	3.4 (1/29)	3.1	100.0 (1/1)	4.6	67.3	13
879	RF02728 HrrF	3.4 (1/29)	1.4	100.0 (1/1)	2.2	82.0	7
880	RF02779 PepN thermometer	3.4 (1/29)	0.3	100.0 (1/1)	1.4	81.8	5
881	RF00765 mir-337	3.4 (1/29)	0.3	100.0 (1/1)	1.1	83.8	6
882	RF01056 Mg sensor	3.4 (1/29)	0.0	100.0 (1/1)	0.9	75.7	4
883	RF00809 mir-241	3.4 (1/29)	0.0	50.0 (1/2)	0.8	76.0	4
884	RF01517 iscRS	3.4 (1/29)	0.0	100.0 (1/1)	0.9	87.3	4
885	RF01853 mtDNA ssA	3.3 (1/30)	22.0	100.0 (1/1)	21.6	67.6	53
886	RF00892 mir-551	3.3 (1/30)	8.0	100.0 (1/1)	8.5	73.3	20
887	RF00650 mir-153	3.3 (1/30)	3.0	100.0 (1/1)	4.4	78.6	18
888	RF00730 mir-277	3.3 (1/30)	2.7	100.0 (1/1)	4.2	69.1	12
889	RF01997 mir-969	3.3 (1/30)	1.3	100.0 (1/1)	2.4	76.1	8
890	RF00768 MIR405	3.3 (1/30)	0.7	100.0 (1/1)	2.2	78.1	13
891	RF00760 mir-342	3.3 (1/30)	0.7	100.0 (1/1)	1.1	85.9	10
892	RF02450 ncr1175	3.3 (1/30)	0.0	100.0 (1/1)	0.6	86.3	4
893	RF02092 mir-2970	3.3 (1/30)	0.0	100.0 (1/1)	0.6	89.5	5
894	RF00774 mir-360	3.3 (1/30)	0.0	100.0 (1/1)	0.9	67.4	5
895	RF00672 mir-190	3.2 (1/31)	16.1	50.0 (1/2)	15.9	68.0	29
896	RF00628 RgsA	3.2 (1/31)	11.3	100.0 (1/1)	11.9	73.3	27
897	RF02994 IMPDH	3.2 (1/31)	7.7	50.0 (1/2)	9.0	81.6	81
898	RF00629 P24	3.2 (2/63)	5.1	100.0 (2/2)	6.1	75.5	14
899	RF01916 mir-988	3.2 (1/31)	0.3	50.0 (1/2)	1.7	77.2	4
900	RF00826 mir-55	3.2 (1/31)	0.3	100.0 (1/1)	1.4	72.6	5
901	RF00936 mir-744	3.2 (1/31)	0.0	100.0 (1/1)	0.4	94.2	5
902	RF00833 mir-70	3.2 (1/31)	0.0	100.0 (1/1)	1.2	58.0	4
903	RF00825 mir-344	3.1 (1/32)	20.3	50.0 (1/2)	20.6	67.2	35
904	RF01267 snR37	3.1 (4/128)	2.8	100.0 (4/4)	4.8	72.0	9
905	RF00302 SNORA65	3.1 (1/32)	2.5	50.0 (1/2)	5.2	72.8	14
906	RF00698 mir-489	3.1 (1/32)	1.9	100.0 (1/1)	4.2	79.1	19
907	RF02094 mir-1803	3.1 (1/32)	0.9	100.0 (1/1)	2.6	66.3	6
908	RF01230 snoR77	3.1 (1/32)	0.6	100.0 (1/1)	2.1	73.9	5
909	RF00828 mir-75	3.1 (1/32)	0.3	100.0 (1/1)	0.9	71.6	4
910	RF00568 SNORA26	3.0 (1/33)	24.8	100.0 (1/1)	24.5	78.2	76
911	RF01851 cyano tmRNA	3.0 (2/67)	10.4	100.0 (2/2)	11.2	83.2	27
912	RF00503 RNAIII	3.0 (4/134)	6.6	100.0 (4/4)	8.8	62.2	10
913	RF00625 P11	3.0 (1/33)	4.8	100.0 (1/1)	5.7	69.0	15

Continued on next page

	RNA family (seed alignment)	Sensitivity annotated bpairs that covary % (cov_bps/bps)	Power average power %	Positive Predictive Value covarying pairs in structure % (cov_bps/cov_pairs)	average substitutions per bpair	avg pairwise identity %	number of sequences
914	RF00957 mir-663	3.0 (1/33)	3.6	100.0 (1/1)	5.4	74.5	12
915	RF00767 mir-150	3.0 (1/33)	3.0	100.0 (1/1)	4.6	80.5	17
916	RF02535 ODC IRES	3.0 (1/33)	3.0	100.0 (1/1)	5.0	79.7	13
917	RF00858 mir-306	3.0 (1/33)	1.2	100.0 (1/1)	3.0	70.2	7
918	RF00653 mir-22	3.0 (1/33)	0.9	100.0 (1/1)	2.5	76.9	12
919	RF02899 AaHKsRNA54	3.0 (1/33)	0.3	100.0 (1/1)	1.7	76.9	6
920	RF01633 ceN43	3.0 (1/33)	0.3	100.0 (1/1)	1.2	86.7	4
921	RF00773 mir-298	3.0 (1/33)	0.3	100.0 (1/1)	2.7	71.3	5
922	RF01910 mir-506	3.0 (1/33)	0.3	100.0 (1/1)	0.8	92.5	8
923	RF00786 mir-289	3.0 (1/33)	0.0	100.0 (1/1)	1.0	85.7	3
924	RF02748 FtrB	3.0 (1/33)	0.0	100.0 (1/1)	1.3	87.8	5
925	RF01418 HIV POL-1 SL	2.9 (1/34)	10.0	100.0 (1/1)	11.0	81.6	29
926	RF00425 SNORA18	2.9 (1/34)	9.1	100.0 (1/1)	10.5	76.7	29
927	RF00746 mir-454	2.9 (1/34)	5.6	100.0 (1/1)	6.4	66.7	17
928	RF00398 SNORA15	2.9 (1/35)	5.4	100.0 (1/1)	7.0	81.1	22
929	RF00565 SCARNA3	2.9 (1/34)	3.8	100.0 (1/1)	5.7	75.9	23
930	RF00544 snopsi28S-3327	2.9 (1/35)	3.4	100.0 (1/1)	4.5	78.4	14
931	RF00562 SNORA49	2.9 (1/34)	3.2	100.0 (1/1)	4.6	81.3	21
932	RF00627 P15	2.9 (1/35)	3.1	100.0 (1/1)	4.3	79.4	22
933	RF01029 mir-649	2.9 (1/35)	0.8	100.0 (1/1)	2.9	74.1	5
934	RF01914 mir-932	2.9 (1/34)	0.6	100.0 (1/1)	2.4	64.9	5
935	RF00448 IRES EBNA	2.9 (1/35)	0.3	100.0 (1/1)	0.8	83.2	6
936	RF00124 IS102	2.9 (1/35)	0.3	100.0 (1/1)	1.2	88.9	5
937	RF00948 mir-996	2.9 (1/35)	0.3	100.0 (1/1)	1.4	77.7	5
938	RF01269 snR80	2.9 (1/34)	0.3	100.0 (1/1)	1.1	87.1	6
939	RF00764 mir-191	2.9 (1/34)	0.0	100.0 (1/1)	0.6	88.3	5
940	RF00340 SNORA36	2.9 (1/34)	0.0	100.0 (1/1)	1.2	85.1	6
941	RF03106 RT-11	2.9 (1/34)	0.0	100.0 (1/1)	1.3	70.5	3
942	RF03114 RT-1	2.9 (1/35)	0.0	100.0 (1/1)	1.0	87.7	5
943	RF00995 mir-616	2.9 (1/34)	0.0	100.0 (1/1)	0.8	79.0	3
944	RF00388 Anti-Q RNA	2.9 (1/34)	0.0	100.0 (1/1)	1.9	72.7	6
945	RF00761 mir-340	2.8 (1/36)	22.5	100.0 (1/1)	21.9	64.0	31
946	RF00614 SNORA11	2.8 (1/36)	8.1	100.0 (1/1)	9.6	82.8	35
947	RF00106 RNAI	2.8 (1/36)	2.5	100.0 (1/1)	3.8	74.9	10
948	RF01655 ceN84	2.8 (1/36)	1.4	100.0 (1/1)	3.7	74.5	5
949	RF00827 mir-77	2.8 (1/36)	0.3	100.0 (1/1)	1.2	78.5	5
950	RF00785 mir-90	2.8 (1/36)	0.3	100.0 (1/1)	1.2	76.3	5
951	RF00449 IRES HIF1	2.7 (1/37)	6.2	50.0 (1/2)	8.0	75.9	17
952	RF01656 ceN72-3 ceN74-2	2.7 (1/37)	5.7	100.0 (1/1)	7.4	70.9	13
953	RF00397 SNORA14	2.7 (1/37)	4.9	100.0 (1/1)	6.5	77.8	18
954	RF00409 SNORA7	2.7 (1/37)	3.8	50.0 (1/2)	5.5	78.6	31
955	RF00415 SNORA30	2.7 (1/37)	3.0	100.0 (1/1)	5.2	72.6	11
956	RF00077 SraB	2.7 (1/37)	0.8	100.0 (1/1)	1.8	85.6	5
957	RF00822 mir-274	2.7 (1/37)	0.8	100.0 (1/1)	1.7	81.6	7
958	RF02714 snopsi28S-3378	2.7 (1/37)	0.0	100.0 (1/1)	1.1	85.9	8
959	RF01649 ceN67	2.7 (1/37)	0.0	100.0 (1/1)	0.9	65.1	3
960	RF02356 BjrC1505	2.6 (1/39)	14.6	100.0 (1/1)	13.9	76.0	25
961	RF01229 SNORA84	2.6 (1/39)	8.7	100.0 (1/1)	9.5	75.4	28
962	RF02471 Ms IGR-5	2.6 (1/39)	8.2	100.0 (1/1)	8.8	73.6	21
963	RF00600 SNORA79	2.6 (1/38)	5.0	100.0 (1/1)	6.9	78.9	25
964	RF00561 SNORA40	2.6 (1/39)	4.1	100.0 (1/1)	5.9	83.6	28
965	RF02235 asX1	2.6 (1/38)	3.1	100.0 (1/1)	5.0	81.1	15
966	RF02079 STnc180	2.6 (1/39)	1.3	100.0 (1/1)	3.5	64.0	10
967	RF00128 GlmY tke1	2.6 (1/38)	1.1	100.0 (1/1)	2.3	76.8	17
968	RF01664 ceN101	2.6 (1/39)	0.8	50.0 (1/2)	1.4	87.7	4
969	RF00401 SNORA20	2.6 (1/39)	0.5	100.0 (1/1)	2.3	87.1	17
970	RF02774 KatA thermometer	2.6 (1/38)	0.5	100.0 (1/1)	1.5	84.8	5
971	RF01653 ceN80	2.6 (1/39)	0.2	100.0 (1/1)	0.9	80.5	5
972	RF01408 sraL	2.6 (1/38)	0.0	100.0 (1/1)	1.3	81.8	6
973	RF01440 S pombe snR42	2.6 (1/38)	0.0	100.0 (1/1)	1.4	73.7	3
974	RF02666 PsiU2-35.45	2.6 (1/38)	0.0	100.0 (1/1)	0.8	84.3	3
975	RF01925 MIR1428	2.6 (1/39)	0.0	100.0 (1/1)	0.5	90.3	5
976	RF00334 SNORA3	2.5 (1/40)	0.8	50.0 (1/2)	2.1	74.4	6
977	RF01259 snR63	2.5 (2/80)	0.2	100.0 (2/2)	1.0	93.1	6
978	RF00888 mir-770	2.5 (1/40)	0.0	100.0 (1/1)	1.4	71.2	4
979	RF02485 GlsR22	2.5 (1/40)	0.0	100.0 (1/1)	0.5	86.4	3
980	RF02036 IMES-3	2.4 (1/42)	15.7	100.0 (1/1)	14.8	81.6	32
981	RF00779 MIR474	2.4 (1/41)	8.3	100.0 (1/1)	10.3	55.9	11
982	RF01766 cspA	2.4 (3/124)	5.4	100.0 (3/3)	7.3	74.5	15
983	RF00470 Toga 5 CRE	2.4 (1/42)	5.2	100.0 (1/1)	5.9	81.0	33
984	RF01405 STnc490	2.4 (1/41)	3.6	100.0 (1/1)	4.8	94.0	76
985	RF01671 P18	2.4 (1/41)	0.2	50.0 (1/2)	1.7	74.6	4
986	RF01663 ceN93	2.4 (1/41)	0.0	100.0 (1/1)	0.6	84.0	4
987	RF02363 Yfr11	2.4 (1/41)	0.0	50.0 (1/2)	2.0	66.7	4
988	RF00235 Plasmid RNAIII	2.4 (1/41)	0.0	100.0 (1/1)	0.6	94.0	7
989	RF00560 SNORA17	2.3 (1/44)	16.8	100.0 (1/1)	17.0	79.6	53
990	RF00100 7SK	2.3 (2/87)	11.6	100.0 (2/2)	12.6	81.7	45
991	RF02355 BjrC174	2.3 (1/43)	10.9	100.0 (1/1)	11.3	75.6	15
992	RF02936 Chloroflexus-1	2.3 (1/43)	0.0	33.3 (1/3)	0.9	79.0	4
993	RF01434 S pombe snR3	2.3 (1/43)	0.0	100.0 (1/1)	1.5	78.2	3
994	RF02462 Telomerase Asco	2.2 (2/89)	0.8	50.0 (2/4)	2.1	69.9	4
995	RF02376 SR1	2.2 (1/45)	0.4	100.0 (1/1)	2.7	74.2	6
996	RF00552 rncO	2.1 (1/47)	4.2	100.0 (1/1)	5.9	83.7	18
997	RF02970 EGFOA-assoc-1	2.1 (1/47)	0.6	100.0 (1/1)	3.0	80.7	10

Continued on next page

	RNA family (seed alignment)	Sensitivity annotated bpairs that covary % (cov_bps/bps)	Power average power %	Positive Predictive Value covarying pairs in structure % (cov_bps/cov_pairs)	average substitutions per bpair	avg pairwise identity %	number of sequences
998	RF01476 rliF	2.1 (1/47)	0.4	100.0 (1/1)	1.4	76.0	5
999	RF01005 MIR530	2.1 (1/47)	0.0	100.0 (1/1)	0.4	73.6	3
1000	RF01581 RUF6-5	2.0 (1/51)	7.6	100.0 (1/1)	9.1	82.1	20
1001	RF01452 S pombe snR99	2.0 (1/50)	0.6	100.0 (1/1)	1.9	84.5	5
1002	RF02792 sRNA162	2.0 (1/50)	0.0	50.0 (1/2)	1.0	67.4	3
1003	RF01789 EBER1	2.0 (1/51)	0.0	100.0 (1/1)	1.0	76.5	3
1004	RF02870 ncS035	2.0 (1/51)	0.0	100.0 (1/1)	0.8	77.4	3
1005	RF02081 STnc550	1.9 (1/53)	1.3	50.0 (1/2)	3.3	79.0	11
1006	RF00125 IS128	1.9 (1/52)	1.1	100.0 (1/1)	2.0	87.7	5
1007	RF02695 NrsZ	1.9 (1/53)	0.9	100.0 (1/1)	2.4	80.1	4
1008	RF01253 snR46	1.8 (1/55)	0.2	100.0 (1/1)	1.5	79.9	6
1009	RF02876 AS-pc02	1.8 (1/57)	0.0	100.0 (1/1)	0.5	92.5	4
1010	RF00231 SCARNA13	1.6 (1/62)	3.7	100.0 (1/1)	5.5	78.9	24
1011	RF02687 RAGATH-8	1.6 (1/63)	0.0	100.0 (1/1)	0.4	91.6	3
1012	RF00090 SNORA74	1.5 (1/67)	7.5	100.0 (1/1)	9.0	77.8	24
1013	RF02423 Bp1 781	1.5 (1/67)	3.3	100.0 (1/1)	4.7	74.8	15
1014	RF02442 SpF66 sRNA	1.4 (1/71)	1.0	100.0 (1/1)	2.9	84.5	9
1015	RF02532 MNV 3UTR	1.4 (1/74)	0.3	50.0 (1/2)	0.9	91.9	10
1016	RF02691 BTH s19	1.4 (1/70)	0.1	100.0 (1/1)	1.6	81.5	7
1017	RF02757 Erse	1.4 (1/74)	0.1	100.0 (1/1)	1.2	88.2	6
1018	RF00563 SNORA53	1.3 (1/75)	6.5	100.0 (1/1)	8.6	80.1	28
1019	RF00551 bicoid 3	1.3 (2/156)	6.0	100.0 (2/2)	7.3	77.7	15
1020	RF00040 rne5	1.2 (1/84)	0.8	100.0 (1/1)	2.9	65.6	6
1021	RF01390 isrG	1.1 (1/90)	0.4	100.0 (1/1)	1.7	85.6	5
1022	RF02858 Arrc08	1.1 (1/95)	0.1	100.0 (1/1)	1.1	76.6	3
1023	RF01570 Dictyostelium SRP	1.1 (1/92)	0.1	100.0 (1/1)	1.3	71.9	3
1024	RF01670 P17	1.1 (1/88)	0.0	100.0 (1/1)	0.4	92.0	3
1025	RF00885 MIR821	0.9 (1/113)	0.1	100.0 (1/1)	0.4	88.6	5
1026	RF02737 Rev13	0.9 (1/112)	0.0	50.0 (1/2)	0.6	79.3	3
1027	RF01473 rli41	0.8 (1/128)	0.1	50.0 (1/2)	0.4	93.1	6
1028	RF01491 rli54	0.7 (1/146)	0.0	100.0 (1/1)	0.2	95.5	5
1029	RF01270 snR84	0.6 (1/157)	0.7	100.0 (1/1)	2.2	79.6	6
1030	RF01050 Sacc telomerase	0.5 (1/191)	4.0	100.0 (1/1)	5.3	73.9	13
1031	RF02576 tsr1	0.5 (1/204)	0.1	100.0 (1/1)	1.2	94.3	3
1032	RF01419 IsrR	0.0 (0/11)	77.3	0.0 (0/3)	157.2	66.9	308
1033	RF01942 mir-1937	0.0 (0/11)	66.4	0.0 (0/0)	84.6	65.8	171
1034	RF00523 Prion pknot	0.0 (0/6)	50.0	0.0 (0/0)	53.0	85.0	148
1035	RF00485 K chan RES	0.0 (0/24)	41.2	0.0 (0/3)	45.1	68.7	85
1036	RF00468 HCV SLVII	0.0 (0/17)	38.8	0.0 (0/0)	42.9	74.3	110
1037	RF00693 mir-147	0.0 (0/15)	29.3	0.0 (0/0)	28.5	68.5	64
1038	RF00480 HIV FE	0.0 (0/10)	27.0	0.0 (0/0)	32.2	84.0	145
1039	RF00093 SNORD18	0.0 (0/4)	22.5	0.0 (0/0)	21.5	69.9	16
1040	RF00376 HIV GSL3	0.0 (0/8)	21.2	0.0 (0/0)	22.0	81.4	72
1041	RF01753 psbNH	0.0 (0/12)	20.8	0.0 (0/0)	21.4	76.6	39
1042	RF00469 HCV SLIV	0.0 (0/15)	20.7	0.0 (0/0)	21.3	86.3	110
1043	RF00047 mir-2	0.0 (0/21)	20.0	0.0 (0/0)	20.4	66.6	56
1044	RF00550 HepE CRE	0.0 (0/41)	17.1	0.0 (0/0)	16.7	84.2	46
1045	RF00535 snoMe28S-Am982	0.0 (0/3)	16.7	0.0 (0/0)	16.3	76.2	13
1046	RF00104 mir-10	0.0 (0/24)	16.7	0.0 (0/0)	16.6	68.1	36
1047	RF00736 mir-320	0.0 (0/19)	16.3	0.0 (0/0)	17.1	68.0	55
1048	RF00654 mir-216	0.0 (0/18)	16.1	0.0 (0/0)	15.8	61.5	33
1049	RF00134 snoZ196	0.0 (0/7)	15.7	0.0 (0/0)	16.7	68.4	22
1050	RF00490 S-element	0.0 (0/22)	15.4	0.0 (0/0)	15.7	75.8	29
1051	RF02510 PYLIS 3	0.0 (0/8)	15.0	0.0 (0/0)	16.5	63.0	23
1052	RF02002 mir-720	0.0 (0/26)	15.0	0.0 (0/0)	15.1	81.2	35
1053	RF02027 MIR2907	0.0 (0/16)	15.0	0.0 (0/0)	15.6	76.9	52
1054	RF00651 mir-221	0.0 (0/21)	14.8	0.0 (0/0)	14.7	73.9	47
1055	RF00041 Entero OriR	0.0 (0/35)	14.0	0.0 (0/0)	14.1	88.0	60
1056	RF01803 GABA3	0.0 (0/21)	13.8	0.0 (0/1)	14.0	84.5	52
1057	RF00665 mir-290	0.0 (0/25)	13.6	0.0 (0/0)	13.7	65.9	27
1058	RF01518 pRNA	0.0 (0/22)	13.6	0.0 (0/0)	14.0	57.2	23
1059	RF00424 SCARNA16	0.0 (0/54)	13.0	0.0 (0/0)	14.3	75.9	37
1060	RF00451 mir-395	0.0 (0/30)	12.7	0.0 (0/0)	12.9	65.0	25
1061	RF00679 mir-210	0.0 (0/27)	12.6	0.0 (0/0)	12.4	61.6	26
1062	RF00670 mir-105	0.0 (0/29)	12.4	0.0 (0/0)	13.3	67.3	20
1063	RF00357 snoR44 J54	0.0 (0/5)	12.0	0.0 (0/0)	11.2	72.1	29
1064	RF02447 SpR19 sRNA	0.0 (0/30)	12.0	0.0 (0/1)	13.0	74.8	23
1065	RF01982 PYLIS 1	0.0 (0/15)	11.3	0.0 (0/0)	10.9	71.7	20
1066	RF00639 mir-515	0.0 (0/19)	11.1	0.0 (0/0)	12.4	80.2	40
1067	RF02516 mir-393	0.0 (0/29)	11.0	0.0 (0/1)	10.9	63.8	27
1068	RF00691 mir-146	0.0 (0/17)	10.6	0.0 (0/0)	10.4	63.1	33
1069	RF00034 RprA	0.0 (0/18)	10.6	0.0 (0/0)	10.4	66.8	13
1070	RF02031 tpke11	0.0 (0/16)	10.6	0.0 (0/0)	10.3	68.9	28
1071	RF00446 mir-133	0.0 (0/20)	10.5	0.0 (0/0)	10.7	67.6	46
1072	RF00311 snoZ188	0.0 (0/6)	10.0	0.0 (0/0)	12.3	78.8	21
1073	RF01304 sR5	0.0 (0/3)	10.0	0.0 (0/0)	12.3	62.1	11
1074	RF01214 snR51	0.0 (0/3)	10.0	0.0 (0/0)	12.3	76.1	17
1075	RF01772 rnk pseudo	0.0 (0/23)	9.6	0.0 (0/0)	10.2	57.3	15
1076	RF02446 SpR18 sRNA	0.0 (0/28)	9.6	0.0 (0/0)	10.8	77.6	23
1077	RF02271 uc 338	0.0 (0/50)	9.2	0.0 (0/0)	10.5	76.1	34
1078	RF00426 SCARNA15	0.0 (0/36)	8.9	0.0 (0/0)	10.4	72.1	22
1079	RF02162 XIST A REPEAT	0.0 (0/7)	8.6	0.0 (0/0)	8.7	84.1	54
1080	RF02025 mir-3017	0.0 (0/23)	8.3	0.0 (0/0)	9.6	84.6	36
1081	RF00246 mir-135	0.0 (0/28)	8.2	0.0 (0/0)	8.5	72.1	32

Continued on next page

	RNA family (seed alignment)	Sensitivity annotated bpairs that covary % (cov_bps/bps)	Power average power %	Positive Predictive Value covarying pairs in structure % (cov_bps/cov_pairs)	average substitutions per bpair	avg pairwise identity %	number of sequences
1082	RF01697 Chlorobi-RRM	0.0 (0/22)	8.2	0.0 (0/0)	9.5	77.5	18
1083	RF01016 mir-584	0.0 (0/37)	8.1	0.0 (0/0)	9.4	63.3	16
1084	RF01059 mir-598	0.0 (0/30)	8.0	0.0 (0/0)	9.4	68.9	24
1085	RF00494 snoU2 19	0.0 (0/9)	7.8	0.0 (0/0)	9.8	79.8	29
1086	RF00245 mir-19	0.0 (0/22)	7.7	0.0 (0/0)	8.1	81.8	46
1087	RF00602 SCARNA21	0.0 (0/31)	7.7	0.0 (0/0)	9.3	75.0	24
1088	RF01396 isrN	0.0 (0/25)	7.6	0.0 (0/0)	9.9	61.4	17
1089	RF01421 snoR114	0.0 (0/4)	7.5	0.0 (0/0)	8.8	64.9	24
1090	RF00157 SNORD39	0.0 (0/4)	7.5	0.0 (0/0)	8.2	82.6	20
1091	RF01791 F6	0.0 (0/20)	7.5	0.0 (0/0)	8.5	71.3	16
1092	RF00582 SCARNA14	0.0 (0/38)	7.4	0.0 (0/0)	8.8	76.9	22
1093	RF01268 SCARNA2	0.0 (0/88)	7.4	0.0 (0/0)	9.4	77.7	20
1094	RF01797 fstAT	0.0 (0/18)	7.2	0.0 (0/1)	9.1	75.9	22
1095	RF01294 snoU89	0.0 (0/72)	7.1	0.0 (0/0)	8.4	75.3	16
1096	RF00131 mir-30	0.0 (0/21)	7.1	0.0 (0/0)	7.4	75.2	49
1097	RF00697 mir-186	0.0 (0/34)	7.0	0.0 (0/0)	8.6	75.0	19
1098	RF00464 mir-92	0.0 (0/26)	6.9	0.0 (0/0)	7.2	76.2	23
1099	RF02534 Noro CRE	0.0 (0/13)	6.9	0.0 (0/0)	8.1	83.1	21
1100	RF00402 SNORA25	0.0 (0/34)	6.8	0.0 (0/0)	8.7	77.6	30
1101	RF00419 SNORA52	0.0 (0/31)	6.8	0.0 (0/0)	7.9	72.5	21
1102	RF00506 Thr leader	0.0 (0/35)	6.8	0.0 (0/0)	8.1	77.1	25
1103	RF00723 mir-448	0.0 (0/30)	6.7	0.0 (0/0)	7.6	74.9	19
1104	RF00144 mir-199	0.0 (0/30)	6.7	0.0 (0/0)	7.4	74.8	40
1105	RF00749 mir-208	0.0 (0/26)	6.5	0.0 (0/0)	7.5	71.9	17
1106	RF00599 SNORA77	0.0 (0/34)	6.5	0.0 (0/0)	7.7	75.1	19
1107	RF00460 U1A PIE	0.0 (0/14)	6.4	0.0 (0/0)	8.0	74.3	39
1108	RF00237 mir-9	0.0 (0/19)	6.3	0.0 (0/0)	7.1	78.4	43
1109	RF01512 Afu 309	0.0 (0/90)	6.3	0.0 (0/0)	8.0	64.3	10
1110	RF00547 IRES TrkB	0.0 (0/117)	6.3	0.0 (0/0)	8.5	74.4	16
1111	RF00139 SNORA72	0.0 (0/30)	6.3	0.0 (0/0)	8.0	82.8	29
1112	RF01673 PhrS	0.0 (0/21)	6.2	0.0 (0/0)	8.3	64.8	13
1113	RF00304 snoZ279 R105 R108	0.0 (0/26)	6.2	0.0 (0/0)	7.6	73.7	20
1114	RF00567 SNORD17	0.0 (0/85)	6.1	0.0 (0/0)	7.9	78.3	19
1115	RF00620 HCV ARF SL	0.0 (0/46)	6.1	0.0 (0/0)	6.7	88.0	36
1116	RF02084 STnc130	0.0 (0/10)	6.0	0.0 (0/1)	5.8	66.3	13
1117	RF00206 U54	0.0 (0/5)	6.0	0.0 (0/1)	5.0	70.1	21
1118	RF00586 SNORA12	0.0 (0/30)	6.0	0.0 (0/0)	7.3	77.1	23
1119	RF00593 snoU83B	0.0 (0/5)	6.0	0.0 (0/0)	9.6	81.6	21
1120	RF01234 SNORA47	0.0 (0/31)	5.8	0.0 (0/0)	7.1	76.0	20
1121	RF00478 SCARNA6	0.0 (0/57)	5.6	0.0 (0/0)	7.3	73.3	17
1122	RF02000 MIR1846	0.0 (0/32)	5.6	0.0 (0/0)	7.5	77.3	21
1123	RF00564 SCARNA11	0.0 (0/41)	5.6	0.0 (0/0)	7.4	79.8	24
1124	RF00445 mir-399	0.0 (0/27)	5.5	0.0 (0/0)	6.5	59.4	13
1125	RF00675 mir-145	0.0 (0/31)	5.5	0.0 (0/0)	5.6	79.3	13
1126	RF00671 mir-138	0.0 (0/24)	5.4	0.0 (0/0)	5.1	79.6	24
1127	RF01813 rdlD	0.0 (0/17)	5.3	0.0 (0/0)	6.0	83.7	52
1128	RF00400 SNORA28	0.0 (0/31)	5.2	0.0 (0/0)	7.0	79.7	26
1129	RF00253 mir-101	0.0 (0/23)	5.2	0.0 (0/0)	5.6	82.9	24
1130	RF00414 SNORA22	0.0 (0/39)	5.1	0.0 (0/0)	7.3	79.4	31
1131	RF00440 SNORD37	0.0 (0/2)	5.0	0.0 (0/0)	7.0	78.9	6
1132	RF01241 SNORA81	0.0 (0/36)	5.0	0.0 (0/0)	7.3	83.6	28
1133	RF00656 mir-205	0.0 (0/24)	5.0	0.0 (0/0)	5.9	73.3	29
1134	RF02419 Spd-sr37	0.0 (0/12)	5.0	0.0 (0/1)	6.1	79.6	25
1135	RF00105 SNORD115	0.0 (0/8)	5.0	0.0 (0/0)	7.4	89.1	32
1136	RF00429 SNORA29	0.0 (0/16)	5.0	0.0 (0/0)	6.2	79.4	26
1137	RF00255 mir-218	0.0 (0/28)	5.0	0.0 (0/0)	5.1	77.3	18
1138	RF01140 sR20	0.0 (0/2)	5.0	0.0 (0/0)	9.5	64.9	6
1139	RF00455 mir-15	0.0 (0/22)	5.0	0.0 (0/0)	5.9	73.3	17
1140	RF00097 snoR71	0.0 (0/8)	5.0	0.0 (0/0)	6.6	82.0	25
1141	RF01781 ASdes	0.0 (0/21)	4.8	0.0 (0/0)	7.0	80.1	26
1142	RF00406 SNORA42	0.0 (0/35)	4.8	0.0 (0/0)	6.7	79.4	21
1143	RF00998 mir-562	0.0 (0/34)	4.7	0.0 (0/0)	6.8	72.8	14
1144	RF02375 Aar	0.0 (0/49)	4.7	0.0 (0/0)	6.3	72.9	13
1145	RF01743 leu-phe leader	0.0 (0/34)	4.7	0.0 (0/0)	6.3	72.5	9
1146	RF00143 mir-6	0.0 (0/24)	4.6	0.0 (0/0)	5.7	70.8	24
1147	RF00416 SNORA43	0.0 (0/26)	4.6	0.0 (0/0)	6.0	75.8	21
1148	RF01099 PK-IAV	0.0 (0/11)	4.5	0.0 (0/0)	5.7	88.2	32
1149	RF00708 mir-450	0.0 (0/29)	4.5	0.0 (0/0)	6.4	78.5	21
1150	RF00732 mir-305	0.0 (0/31)	4.5	0.0 (0/0)	5.3	75.2	14
1151	RF00267 snoR64	0.0 (0/11)	4.5	0.0 (0/1)	6.2	69.7	14
1152	RF02432 SpF25 sRNA	0.0 (0/34)	4.4	0.0 (0/0)	5.3	83.4	20
1153	RF00644 mir-27	0.0 (0/18)	4.4	0.0 (0/0)	5.2	67.3	32
1154	RF02506 Atu Ti1	0.0 (0/27)	4.4	0.0 (0/0)	6.1	72.6	14
1155	RF00129 mir-103	0.0 (0/18)	4.4	0.0 (0/0)	4.8	84.9	28
1156	RF02449 ncr1015	0.0 (0/23)	4.3	0.0 (0/0)	5.4	71.7	16
1157	RF00328 snoZ161 228	0.0 (0/7)	4.3	0.0 (0/0)	7.3	67.6	11
1158	RF00152 SNORD79	0.0 (0/7)	4.3	0.0 (0/0)	6.1	76.2	28
1159	RF01045 mir-544	0.0 (0/30)	4.3	0.0 (0/0)	6.3	73.1	24
1160	RF00241 mir-8	0.0 (0/21)	4.3	0.0 (0/0)	5.7	60.3	13
1161	RF00527 snoMe28S-G3255	0.0 (0/7)	4.3	0.0 (0/0)	4.7	78.8	10
1162	RF01380 HIV-1 SD	0.0 (0/7)	4.3	0.0 (0/0)	4.3	93.6	22
1163	RF00886 MIR807	0.0 (0/64)	4.2	0.0 (0/0)	5.9	85.0	30
1164	RF00431 SNORA55	0.0 (0/31)	4.2	0.0 (0/0)	5.3	81.3	29
1165	RF00076 mir-181	0.0 (0/24)	4.2	0.0 (0/0)	4.5	81.1	19

Continued on next page

	RNA family (seed alignment)	Sensitivity annotated bpairs that covary % (cov_bps/bps)	Power average power %	Positive Predictive Value covarying pairs in structure % (cov_bps/cov_pairs)	average substitutions per bpair	avg pairwise identity %	number of sequences
1166	RF02526 SSRC34 1	0.0 (0/36)	4.2	0.0 (0/1)	5.7	83.4	16
1167	RF00048 Entero CRE	0.0 (0/12)	4.2	0.0 (0/1)	5.2	81.7	56
1168	RF00495 IRES Hsp70	0.0 (0/54)	4.1	0.0 (0/1)	6.5	81.1	14
1169	RF00332 snoZ266	0.0 (0/10)	4.0	0.0 (0/0)	6.9	74.8	11
1170	RF00358 snoZ101	0.0 (0/10)	4.0	0.0 (0/0)	5.9	76.5	13
1171	RF00296 snoR16	0.0 (0/5)	4.0	0.0 (0/0)	4.8	74.7	18
1172	RF00554 SNORA48	0.0 (0/38)	3.9	0.0 (0/0)	6.8	78.5	24
1173	RF00646 mir-204	0.0 (0/28)	3.9	0.0 (0/0)	4.7	72.5	32
1174	RF00763 mir-339	0.0 (0/33)	3.9	0.0 (0/0)	4.9	75.1	14
1175	RF00917 mir-708	0.0 (0/31)	3.9	0.0 (0/0)	4.7	79.9	21
1176	RF00405 SNORA44	0.0 (0/21)	3.8	0.0 (0/0)	6.4	81.5	27
1177	RF01811 PtaRNA1	0.0 (0/21)	3.8	0.0 (0/0)	4.5	78.3	16
1178	RF02514 5 ureB sRNA	0.0 (0/90)	3.7	0.0 (0/0)	5.9	76.3	13
1179	RF00404 SNORA46	0.0 (0/41)	3.6	0.0 (0/0)	5.3	82.6	22
1180	RF00215 Tombus 3 III	0.0 (0/14)	3.6	0.0 (0/1)	6.6	79.5	28
1181	RF00493 snoU2-30	0.0 (0/11)	3.6	0.0 (0/0)	4.3	87.9	21
1182	RF00661 mir-31	0.0 (0/14)	3.6	0.0 (0/0)	4.6	63.6	28
1183	RF00623 P1	0.0 (0/28)	3.6	0.0 (0/1)	4.8	67.7	14
1184	RF00252 Alfamo CPB	0.0 (0/56)	3.6	0.0 (0/0)	4.0	89.6	18
1185	RF02060 STnc410	0.0 (0/17)	3.5	0.0 (0/0)	6.4	70.7	14
1186	RF00183 G-CSF SLDE	0.0 (0/26)	3.5	0.0 (0/0)	4.9	80.6	16
1187	RF00728 mir-81	0.0 (0/34)	3.5	0.0 (0/0)	5.0	64.5	9
1188	RF02548 Oskar OES	0.0 (0/21)	3.3	0.0 (0/0)	5.0	80.3	14
1189	RF02061 mir-301	0.0 (0/24)	3.3	0.0 (0/0)	4.2	77.5	17
1190	RF00581 SNORD12	0.0 (0/9)	3.3	0.0 (0/0)	7.2	71.1	8
1191	RF01203 snR47	0.0 (0/3)	3.3	0.0 (0/0)	4.0	73.9	17
1192	RF01237 snR161	0.0 (0/36)	3.3	0.0 (0/0)	5.2	65.2	10
1193	RF00427 SCARNA23	0.0 (0/36)	3.3	0.0 (0/0)	5.3	79.0	18
1194	RF00278 SNORD50	0.0 (0/3)	3.3	0.0 (0/0)	6.3	81.7	26
1195	RF00745 mir-499	0.0 (0/25)	3.2	0.0 (0/0)	3.5	77.9	19
1196	RF00662 mir-132	0.0 (0/16)	3.1	0.0 (0/1)	3.1	66.4	22
1197	RF01392 isrI	0.0 (0/13)	3.1	0.0 (0/0)	4.0	82.4	30
1198	RF01019 mir-922	0.0 (0/27)	3.0	0.0 (0/0)	4.8	80.2	12
1199	RF00456 mir-34	0.0 (0/30)	3.0	0.0 (0/0)	4.8	74.0	18
1200	RF00408 SNORA1	0.0 (0/24)	2.9	0.0 (0/0)	5.2	76.6	29
1201	RF00683 mir-143	0.0 (0/28)	2.9	0.0 (0/1)	3.0	78.4	18
1202	RF02434 SpF39 sRNA	0.0 (0/28)	2.9	0.0 (0/0)	4.9	83.2	13
1203	RF01879 TUSC7	0.0 (0/24)	2.9	0.0 (0/0)	5.2	87.5	25
1204	RF00265 SNORA69	0.0 (0/38)	2.9	0.0 (0/0)	5.1	83.9	16
1205	RF00316 snoR43	0.0 (0/7)	2.9	0.0 (0/0)	5.4	86.9	16
1206	RF02261 MAT2A B	0.0 (0/21)	2.9	0.0 (0/0)	4.1	94.0	28
1207	RF00598 SNORA76	0.0 (0/39)	2.8	0.0 (0/0)	4.5	83.7	22
1208	RF02095 mir-2985-2	0.0 (0/32)	2.8	0.0 (0/0)	4.0	83.8	20
1209	RF01010 mir-632	0.0 (0/32)	2.8	0.0 (0/0)	5.1	82.9	16
1210	RF00841 mir-384	0.0 (0/26)	2.7	0.0 (0/0)	4.8	72.2	16
1211	RF00423 SCARNA4	0.0 (0/33)	2.7	0.0 (0/0)	4.8	82.9	24
1212	RF01249 snR190	0.0 (0/62)	2.7	0.0 (0/0)	5.1	80.6	10
1213	RF00578 SNORD89	0.0 (0/33)	2.7	0.0 (0/0)	4.3	88.5	18
1214	RF02260 MAT2A A	0.0 (0/19)	2.6	0.0 (0/0)	4.7	89.7	20
1215	RF00410 SNORA2	0.0 (0/23)	2.6	0.0 (0/0)	4.4	75.8	18
1216	RF00876 mir-684	0.0 (0/27)	2.6	0.0 (0/0)	4.4	88.3	28
1217	RF02531 NRF2 IRES	0.0 (0/27)	2.6	0.0 (0/0)	5.1	82.3	20
1218	RF00682 mir-144	0.0 (0/27)	2.6	0.0 (0/0)	3.4	84.0	22
1219	RF01033 mir-767	0.0 (0/39)	2.6	0.0 (0/0)	4.0	82.7	14
1220	RF02701 PsrR1	0.0 (0/8)	2.5	0.0 (0/1)	5.1	65.9	13
1221	RF01182 SNORD11	0.0 (0/4)	2.5	0.0 (0/0)	6.8	85.2	19
1222	RF00279 SNORD45	0.0 (0/4)	2.5	0.0 (0/0)	4.5	78.8	11
1223	RF00339 snoR60	0.0 (0/4)	2.5	0.0 (0/0)	2.5	84.5	10
1224	RF02234 sX15	0.0 (0/48)	2.5	0.0 (0/0)	3.2	82.6	14
1225	RF01832 ROSE 2	0.0 (0/16)	2.5	0.0 (0/0)	3.1	83.7	14
1226	RF00438 SNORA33	0.0 (0/24)	2.5	0.0 (0/0)	4.7	77.8	28
1227	RF00607 SNORD98	0.0 (0/4)	2.5	0.0 (0/0)	4.8	81.6	10
1228	RF00733 mir-296	0.0 (0/33)	2.4	0.0 (0/0)	3.7	83.2	12
1229	RF00840 mir-374	0.0 (0/29)	2.4	0.0 (0/0)	3.8	81.7	13
1230	RF01770 rimP	0.0 (0/13)	2.3	0.0 (0/0)	2.8	71.2	46
1231	RF02100 tfoR	0.0 (0/30)	2.3	0.0 (0/0)	3.9	81.8	10
1232	RF00108 SNORD116	0.0 (0/13)	2.3	0.0 (0/0)	3.7	82.0	48
1233	RF00214 Retro dr1	0.0 (0/22)	2.3	0.0 (0/0)	3.4	89.2	26
1234	RF00983 mir-662	0.0 (0/35)	2.3	0.0 (0/0)	3.9	84.8	14
1235	RF00875 mir-692	0.0 (0/22)	2.3	0.0 (0/0)	4.5	78.0	11
1236	RF03024 Rothia-sucC	0.0 (0/9)	2.2	0.0 (0/0)	4.6	82.5	28
1237	RF00155 SNORA66	0.0 (0/23)	2.2	0.0 (0/0)	3.7	81.8	25
1238	RF00362 Pospi RY	0.0 (0/23)	2.2	0.0 (0/0)	2.7	92.2	16
1239	RF00333 snoZ157	0.0 (0/18)	2.2	0.0 (0/0)	5.3	76.3	10
1240	RF01200 SNORD125	0.0 (0/9)	2.2	0.0 (0/1)	4.0	81.4	16
1241	RF00465 JEV hairpin	0.0 (0/18)	2.2	0.0 (0/0)	3.3	86.5	20
1242	RF01911 MIR2118	0.0 (0/43)	2.1	0.0 (0/0)	4.3	65.3	7
1243	RF01173 snoU105B	0.0 (0/14)	2.1	0.0 (0/0)	3.9	78.2	13
1244	RF00542 snopsi28S-1192	0.0 (0/30)	2.0	0.0 (0/0)	3.0	81.5	15
1245	RF01414 class I RNA	0.0 (0/10)	2.0	0.0 (0/0)	3.6	77.4	21
1246	RF01320 CRISPR-DR7	0.0 (0/5)	2.0	0.0 (0/0)	2.2	76.9	10
1247	RF02227 sX8	0.0 (0/25)	2.0	0.0 (0/0)	4.2	79.2	12
1248	RF00673 mir-217	0.0 (0/30)	2.0	0.0 (0/0)	3.6	78.2	27
1249	RF00579 SNORD90	0.0 (0/30)	2.0	0.0 (0/0)	3.4	86.7	18

Continued on next page

	RNA family (seed alignment)	Sensitivity annotated bpairs that covary % (cov_bps/bps)	Power average power %	Positive Predictive Value covarying pairs in structure % (cov_bps/cov_pairs)	average substitutions per bpair	avg pairwise identity %	number of sequences
1250	RF01231 snoR74	0.0 (0/25)	2.0	0.0 (0/0)	4.0	68.0	9
1251	RF03002 lysM-Prevotella	0.0 (0/10)	2.0	0.0 (0/0)	4.3	79.6	47
1252	RF00951 mir-1302	0.0 (0/15)	2.0	0.0 (0/0)	4.6	82.8	24
1253	RF00428 SNORA38	0.0 (0/25)	2.0	0.0 (0/0)	3.6	83.9	25
1254	RF00570 SNORD64	0.0 (0/15)	2.0	0.0 (0/0)	4.9	80.5	17
1255	RF00074 mir-29	0.0 (0/21)	1.9	0.0 (0/0)	2.4	74.5	10
1256	RF01540 TB11Cs4H2	0.0 (0/16)	1.9	0.0 (0/0)	3.2	75.0	6
1257	RF02064 STnc370	0.0 (0/16)	1.9	0.0 (0/0)	3.0	78.9	10
1258	RF00228 IRES HepA	0.0 (0/102)	1.9	0.0 (0/0)	2.4	96.4	23
1259	RF00482 snoF1 F2	0.0 (0/32)	1.9	0.0 (0/0)	2.8	86.5	8
1260	RF00664 mir-223	0.0 (0/31)	1.9	0.0 (0/0)	2.6	77.5	19
1261	RF02057 STnc40	0.0 (0/16)	1.9	0.0 (0/0)	3.2	68.5	17
1262	RF02222 sX2	0.0 (0/31)	1.9	0.0 (0/0)	2.9	88.8	9
1263	RF01668 P10	0.0 (0/16)	1.9	0.0 (0/0)	3.1	71.6	8
1264	RF01840 ovine lenti FSE	0.0 (0/17)	1.8	0.0 (0/0)	2.6	88.1	14
1265	RF00126 ryfA	0.0 (0/83)	1.8	0.0 (0/0)	3.5	74.1	9
1266	RF03029 RT-8	0.0 (0/22)	1.8	0.0 (0/0)	4.6	81.0	9
1267	RF02228 sX9	0.0 (0/17)	1.8	0.0 (0/0)	4.1	76.8	16
1268	RF02422 Bp1 738	0.0 (0/33)	1.8	0.0 (0/0)	3.3	75.0	21
1269	RF00753 mir-503	0.0 (0/28)	1.8	0.0 (0/0)	2.6	87.8	14
1270	RF01757 sbcD	0.0 (0/51)	1.8	0.0 (0/0)	3.2	77.5	6
1271	RF00576 SNORD71	0.0 (0/22)	1.8	0.0 (0/0)	2.5	83.3	18
1272	RF00783 mir-484	0.0 (0/23)	1.7	0.0 (0/0)	4.8	83.1	15
1273	RF00180 REN-SRE	0.0 (0/6)	1.7	0.0 (0/0)	4.2	89.4	13
1274	RF00109 Vimentin3	0.0 (0/18)	1.7	0.0 (0/0)	4.1	75.5	19
1275	RF02265 MAT2A F	0.0 (0/23)	1.7	0.0 (0/0)	2.7	94.3	26
1276	RF00731 mir-155	0.0 (0/23)	1.7	0.0 (0/0)	3.3	77.7	17
1277	RF02495 ohsC RNA	0.0 (0/24)	1.7	0.0 (0/0)	3.8	90.2	34
1278	RF02264 MAT2A E	0.0 (0/24)	1.7	0.0 (0/0)	3.0	93.0	28
1279	RF00674 mir-187	0.0 (0/23)	1.7	0.0 (0/0)	2.2	76.5	20
1280	RF00443 SNORA27	0.0 (0/24)	1.7	0.0 (0/0)	4.2	84.3	24
1281	RF00727 bantam	0.0 (0/25)	1.6	0.0 (0/0)	3.1	71.2	11
1282	RF00928 mir-590	0.0 (0/32)	1.6	0.0 (0/0)	3.8	76.6	10
1283	RF01018 mir-569	0.0 (0/31)	1.6	0.0 (0/0)	3.4	85.8	13
1284	RF00747 mir-283	0.0 (0/25)	1.6	0.0 (0/0)	3.4	69.7	11
1285	RF00669 mir-96	0.0 (0/26)	1.5	0.0 (0/0)	2.5	89.2	25
1286	RF00457 IRES mnt	0.0 (0/33)	1.5	0.0 (0/0)	3.2	91.6	21
1287	RF00222 IRES Bag1	0.0 (0/52)	1.5	0.0 (0/0)	3.5	78.8	15
1288	RF01238 snR70	0.0 (0/52)	1.5	0.0 (0/0)	3.7	81.6	9
1289	RF00459 MPMV package	0.0 (0/57)	1.4	0.0 (0/0)	3.6	71.9	9
1290	RF01787 drz-agam-1	0.0 (0/22)	1.4	0.0 (0/0)	4.3	72.6	7
1291	RF00660 mir-214	0.0 (0/36)	1.4	0.0 (0/0)	2.3	77.9	8
1292	RF00145 snoZ105	0.0 (0/7)	1.4	0.0 (0/0)	4.0	65.3	11
1293	RF02808 PyrR210	0.0 (0/14)	1.4	0.0 (0/0)	2.7	81.0	6
1294	RF00621 CoTC ribozyme	0.0 (0/49)	1.4	0.0 (0/0)	3.2	89.7	10
1295	RF00390 UPSK	0.0 (0/7)	1.4	0.0 (0/0)	1.6	93.6	6
1296	RF02465 Ms AS-5	0.0 (0/7)	1.4	0.0 (0/0)	2.6	84.6	9
1297	RF00712 mir-460	0.0 (0/21)	1.4	0.0 (0/0)	1.7	77.5	8
1298	RF03042 porB	0.0 (0/7)	1.4	0.0 (0/0)	4.6	82.7	85
1299	RF00952 mir-650	0.0 (0/29)	1.4	0.0 (0/0)	3.7	83.8	11
1300	RF01036 mir-567	0.0 (0/28)	1.4	0.0 (0/0)	3.9	72.3	9
1301	RF00319 SNORA23	0.0 (0/45)	1.3	0.0 (0/0)	3.0	83.0	9
1302	RF00081 ArcZ	0.0 (0/15)	1.3	0.0 (0/0)	2.5	77.2	9
1303	RF02052 STnc630	0.0 (0/31)	1.3	0.0 (0/1)	2.5	76.8	10
1304	RF01927 MIR1222	0.0 (0/39)	1.3	0.0 (0/0)	3.5	57.8	5
1305	RF00705 mir-202	0.0 (0/30)	1.3	0.0 (0/0)	2.1	81.2	13
1306	RF00622 CPEB3 ribozyme	0.0 (0/23)	1.3	0.0 (0/0)	2.7	84.5	12
1307	RF01093 RF site5	0.0 (0/16)	1.2	0.0 (0/0)	2.5	76.4	12
1308	RF00989 mir-492	0.0 (0/32)	1.2	0.0 (0/0)	4.0	82.0	14
1309	RF00198 SL1	0.0 (0/24)	1.2	0.0 (0/0)	1.7	92.1	28
1310	RF01990 SECIS 4	0.0 (0/8)	1.2	0.0 (0/0)	1.9	88.4	24
1311	RF02230 sX11	0.0 (0/43)	1.2	0.0 (0/0)	2.5	84.9	10
1312	RF00696 mir-203	0.0 (0/25)	1.2	0.0 (0/0)	2.0	79.6	10
1313	RF00247 mir-160	0.0 (0/25)	1.2	0.0 (0/0)	2.6	65.8	7
1314	RF00704 MIR397	0.0 (0/25)	1.2	0.0 (0/0)	2.5	60.1	7
1315	RF01546 TB6Cs1H1	0.0 (0/16)	1.2	0.0 (0/1)	1.6	79.5	6
1316	RF01558 TB9Cs4H2	0.0 (0/16)	1.2	0.0 (0/0)	2.4	83.7	5
1317	RF01903 mir-500	0.0 (0/25)	1.2	0.0 (0/0)	2.9	78.5	26
1318	RF00487 IRES Cx43	0.0 (0/56)	1.1	0.0 (0/0)	3.2	87.2	14
1319	RF00226 IRES n-myc	0.0 (0/36)	1.1	0.0 (0/0)	2.5	72.2	6
1320	RF02552 RcsR1	0.0 (0/28)	1.1	0.0 (0/1)	2.6	81.9	10
1321	RF01387 isrC	0.0 (0/37)	1.1	0.0 (0/0)	3.1	90.9	16
1322	RF00381 Antizyme FSE	0.0 (0/18)	1.1	0.0 (0/0)	1.6	84.2	13
1323	RF00751 mir-12	0.0 (0/27)	1.1	0.0 (0/0)	2.1	77.5	7
1324	RF00658 mir-21	0.0 (0/27)	1.1	0.0 (0/0)	2.6	80.3	11
1325	RF00375 HIV PBS	0.0 (0/18)	1.1	0.0 (0/0)	2.7	90.9	130
1326	RF02518 mir-2494	0.0 (0/38)	1.1	0.0 (0/0)	3.0	81.8	10
1327	RF00541 snopsi28S-2876	0.0 (0/28)	1.1	0.0 (0/0)	3.1	80.5	8
1328	RF01775 RsaOG	0.0 (0/36)	1.1	0.0 (0/1)	2.5	84.7	7
1329	RF01549 TB8Cs2H1	0.0 (0/18)	1.1	0.0 (0/0)	3.5	69.9	6
1330	RF00195 RsmY	0.0 (0/19)	1.1	0.0 (0/1)	2.1	77.0	11
1331	RF00240 RNA-OUT	0.0 (0/20)	1.0	0.0 (0/0)	3.0	86.8	16
1332	RF01031 mir-639	0.0 (0/31)	1.0	0.0 (0/0)	2.3	76.2	6
1333	RF01061 mir-548	0.0 (0/40)	1.0	0.0 (0/0)	2.6	84.8	11

Continued on next page

	RNA family (seed alignment)	Sensitivity annotated bpairs that covary % (cov_bps/bps)	Power average power %	Positive Predictive Value covarying pairs in structure % (cov_bps/cov_pairs)	average substitutions per bpair	avg pairwise identity %	number of sequences
1334	RF02420 Bp1 162	0.0 (0/20)	1.0	0.0 (0/0)	2.5	82.9	11
1335	RF01228 snoR111	0.0 (0/31)	1.0	0.0 (0/1)	2.5	66.7	6
1336	RF00242 ctRNA pT181	0.0 (0/20)	1.0	0.0 (0/0)	2.3	80.9	16
1337	RF02238 asX4	0.0 (0/87)	1.0	0.0 (0/0)	2.3	86.0	8
1338	RF02911 Baculoviridae NAE	0.0 (0/29)	1.0	0.0 (0/0)	2.9	72.3	6
1339	RF02438 SpF51 sRNA	0.0 (0/10)	1.0	0.0 (0/0)	1.5	90.1	8
1340	RF00452 mir-172	0.0 (0/23)	0.9	0.0 (0/0)	2.0	60.6	11
1341	RF02503 Atu C9	0.0 (0/21)	0.9	0.0 (0/0)	2.3	74.1	9
1342	RF01836 weev FSE	0.0 (0/11)	0.9	0.0 (0/0)	1.5	87.3	6
1343	RF00686 mir-338	0.0 (0/21)	0.9	0.0 (0/0)	1.9	73.0	21
1344	RF02786 snoTBR2	0.0 (0/33)	0.9	0.0 (0/1)	3.0	72.5	5
1345	RF01402 STnc150	0.0 (0/22)	0.9	0.0 (0/22)	3.0	91.2	9
1346	RF00417 SNORA56	0.0 (0/33)	0.9	0.0 (0/0)	3.5	82.6	17
1347	RF02424 Bp2 287	0.0 (0/44)	0.9	0.0 (0/0)	1.6	83.5	14
1348	RF00492 SCARNA17	0.0 (0/32)	0.9	0.0 (0/0)	3.2	76.4	6
1349	RF02053 STnc430	0.0 (0/33)	0.9	0.0 (0/0)	3.1	68.6	7
1350	RF01784 bablM	0.0 (0/11)	0.9	0.0 (0/0)	1.5	89.9	12
1351	RF01897 mir-188	0.0 (0/21)	0.9	0.0 (0/0)	3.0	71.2	14
1352	RF00036 RRE	0.0 (0/116)	0.9	0.0 (0/0)	1.6	97.0	65
1353	RF00994 mir-1255	0.0 (0/21)	0.9	0.0 (0/0)	2.5	83.1	10
1354	RF00121 MicC	0.0 (0/23)	0.9	0.0 (0/0)	2.0	71.5	8
1355	RF00663 mir-183	0.0 (0/23)	0.9	0.0 (0/0)	2.3	78.1	16
1356	RF02891 AgrB	0.0 (0/21)	0.9	0.0 (0/0)	1.7	77.4	4
1357	RF02546 LSU trypano mito	0.0 (0/58)	0.9	0.0 (0/0)	4.1	76.9	6
1358	RF01895 mir-193	0.0 (0/23)	0.9	0.0 (0/0)	2.1	72.1	17
1359	RF01824 RUF20	0.0 (0/76)	0.9	0.0 (0/0)	3.8	73.8	6
1360	RF02512 PYLIS 5	0.0 (0/22)	0.9	0.0 (0/0)	1.2	94.0	17
1361	RF00176 Tombus 3 IV	0.0 (0/25)	0.8	0.0 (0/0)	1.2	93.3	18
1362	RF01941 MIR1223	0.0 (0/25)	0.8	0.0 (0/0)	2.4	59.5	8
1363	RF01291 SNORD97	0.0 (0/13)	0.8	0.0 (0/1)	2.1	86.5	20
1364	RF01220 snoR104	0.0 (0/24)	0.8	0.0 (0/0)	3.4	66.4	8
1365	RF02371 PyrG leader	0.0 (0/12)	0.8	0.0 (0/0)	1.9	73.9	10
1366	RF02232 sX13	0.0 (0/37)	0.8	0.0 (0/0)	1.9	90.4	12
1367	RF01401 rseX	0.0 (0/13)	0.8	0.0 (0/0)	2.3	76.1	12
1368	RF00865 MIR169 5	0.0 (0/26)	0.8	0.0 (0/0)	2.6	65.1	6
1369	RF00636 NRON	0.0 (0/64)	0.8	0.0 (0/0)	2.0	91.9	24
1370	RF01052 Arthropod 7SK	0.0 (0/39)	0.8	0.0 (0/0)	2.5	67.0	19
1371	RF00735 mir-367	0.0 (0/25)	0.8	0.0 (0/0)	1.5	85.9	13
1372	RF00489 ctRNA p42d	0.0 (0/12)	0.8	0.0 (0/0)	2.2	86.2	10
1373	RF00394 SNORA4	0.0 (0/40)	0.8	0.0 (0/0)	2.5	76.4	7
1374	RF00142 snoZ118	0.0 (0/13)	0.8	0.0 (0/0)	2.8	84.2	7
1375	RF00467 RSV PBS	0.0 (0/15)	0.7	0.0 (0/0)	0.9	92.7	22
1376	RF00999 mir-924	0.0 (0/14)	0.7	0.0 (0/0)	2.1	81.5	4
1377	RF00772 mir-328	0.0 (0/28)	0.7	0.0 (0/0)	2.3	79.3	13
1378	RF00659 mir-365	0.0 (0/29)	0.7	0.0 (0/1)	2.2	80.4	9
1379	RF01996 mir-995	0.0 (0/30)	0.7	0.0 (0/0)	1.8	77.8	9
1380	RF00361 snoZ119	0.0 (0/15)	0.7	0.0 (0/0)	1.1	94.0	6
1381	RF00792 mir-490	0.0 (0/28)	0.7	0.0 (0/0)	2.9	78.5	19
1382	RF00678 mir-140	0.0 (0/30)	0.7	0.0 (0/0)	1.2	87.0	14
1383	RF01449 S pombe snR100	0.0 (0/57)	0.7	0.0 (0/0)	3.0	68.0	3
1384	RF02802 PyrR204	0.0 (0/15)	0.7	0.0 (0/0)	1.5	76.7	5
1385	RF01393 isrJ	0.0 (0/15)	0.7	0.0 (0/0)	3.0	76.3	5
1386	RF00756 mir-299	0.0 (0/28)	0.7	0.0 (0/0)	1.0	87.1	6
1387	RF01208 snoR99	0.0 (0/14)	0.7	0.0 (0/0)	2.2	70.0	9
1388	RF02466 Ms AS-8	0.0 (0/15)	0.7	0.0 (0/0)	2.1	78.9	10
1389	RF01790 htlv FSE	0.0 (0/14)	0.7	0.0 (0/0)	1.3	84.5	8
1390	RF01219 snoR100	0.0 (0/15)	0.7	0.0 (0/0)	2.3	71.8	9
1391	RF01703 Dictyoglomi-1	0.0 (0/81)	0.7	0.0 (0/0)	2.0	78.5	4
1392	RF00072 SNORA75	0.0 (0/28)	0.7	0.0 (0/0)	1.5	73.3	6
1393	RF00832 mir-71	0.0 (0/17)	0.6	0.0 (0/0)	2.2	74.2	12
1394	RF00801 mir-280	0.0 (0/35)	0.6	0.0 (0/0)	1.6	86.5	8
1395	RF01455 DPB	0.0 (0/18)	0.6	0.0 (0/0)	2.0	85.0	19
1396	RF00505 RydC	0.0 (0/16)	0.6	0.0 (0/0)	1.7	86.4	5
1397	RF00461 IRES VEGF A	0.0 (0/95)	0.6	0.0 (0/0)	1.7	90.7	7
1398	RF00737 mir-322	0.0 (0/31)	0.6	0.0 (0/0)	1.8	88.9	11
1399	RF01008 mir-636	0.0 (0/31)	0.6	0.0 (0/0)	1.9	78.3	5
1400	RF00959 mir-612	0.0 (0/35)	0.6	0.0 (0/0)	2.1	76.1	6
1401	RF01773 rpsL pseudo	0.0 (0/35)	0.6	0.0 (0/1)	2.5	80.6	9
1402	RF02374 YenS	0.0 (0/54)	0.6	0.0 (0/0)	1.9	82.1	7
1403	RF00700 mir-375	0.0 (0/17)	0.6	0.0 (0/0)	1.1	78.9	9
1404	RF02425 SpF01 sRNA	0.0 (0/16)	0.6	0.0 (0/0)	2.8	83.1	13
1405	RF02421 Bp1 684	0.0 (0/16)	0.6	0.0 (0/1)	1.1	86.3	14
1406	RF01012 mir-628	0.0 (0/32)	0.6	0.0 (0/1)	2.9	78.2	9
1407	RF02262 MAT2A C	0.0 (0/18)	0.6	0.0 (0/0)	2.1	90.9	18
1408	RF02551 DapZ	0.0 (0/18)	0.6	0.0 (0/0)	1.1	79.9	6
1409	RF00501 Rota CRE	0.0 (0/17)	0.6	0.0 (0/1)	1.6	86.6	14
1410	RF02819 V AS7	0.0 (0/53)	0.6	0.0 (0/0)	2.6	78.1	7
1411	RF00223 IRES Bip	0.0 (0/33)	0.6	0.0 (0/2)	2.4	81.1	9
1412	RF01524 TB10Cs1H3	0.0 (0/17)	0.6	0.0 (0/0)	1.4	80.8	5
1413	RF01023 mir-940	0.0 (0/34)	0.6	0.0 (0/0)	1.7	89.3	8
1414	RF00872 mir-652	0.0 (0/31)	0.6	0.0 (0/0)	2.5	76.9	13
1415	RF00065 snoR9	0.0 (0/18)	0.6	0.0 (0/1)	1.3	83.7	5
1416	RF00251 mir-219	0.0 (0/17)	0.6	0.0 (0/0)	1.9	83.4	13
1417	RF01215 snoR97	0.0 (0/17)	0.6	0.0 (0/0)	1.4	72.2	7

Continued on next page

	RNA family (seed alignment)	Sensitivity annotated bpairs that covary % (cov_bps/bps)	Power average power %	Positive Predictive Value covarying pairs in structure % (cov_bps/cov_pairs)	average substitutions per bpair	avg pairwise identity %	number of sequences
1418	RF00991 mir-599	0.0 (0/35)	0.6	0.0 (0/0)	2.3	86.2	11
1419	RF00912 mir-877	0.0 (0/18)	0.6	0.0 (0/0)	2.6	81.1	13
1420	RF01385 isrA	0.0 (0/20)	0.5	0.0 (0/0)	2.2	87.6	10
1421	RF00194 Rubella 3	0.0 (0/20)	0.5	0.0 (0/0)	2.4	95.3	25
1422	RF00844 mir-67	0.0 (0/19)	0.5	0.0 (0/0)	1.7	77.7	14
1423	RF01523 TB10Cs1H2	0.0 (0/19)	0.5	0.0 (0/0)	1.8	79.8	5
1424	RF00261 IRES L-myc	0.0 (0/75)	0.5	0.0 (0/0)	1.9	88.6	11
1425	RF02722 sca ncR27	0.0 (0/22)	0.5	0.0 (0/0)	1.5	80.3	4
1426	RF02524 sagA	0.0 (0/41)	0.5	0.0 (0/0)	2.1	69.4	6
1427	RF02270 nse sRNA	0.0 (0/19)	0.5	0.0 (0/0)	0.9	90.1	7
1428	RF00778 MIR473	0.0 (0/20)	0.5	0.0 (0/0)	1.8	59.0	6
1429	RF01940 hvt-mir-H	0.0 (0/20)	0.5	0.0 (0/0)	2.5	67.2	4
1430	RF02093 mir-2968	0.0 (0/39)	0.5	0.0 (0/0)	2.2	87.8	12
1431	RF02231 sX12	0.0 (0/21)	0.5	0.0 (0/0)	0.8	94.4	8
1432	RF00254 mir-16	0.0 (0/19)	0.5	0.0 (0/0)	1.3	72.9	12
1433	RF00689 MIR390	0.0 (0/20)	0.5	0.0 (0/0)	1.1	73.8	16
1434	RF02883 BcKCsr2	0.0 (0/38)	0.5	0.0 (0/0)	2.3	72.7	5
1435	RF01771 rnk leader	0.0 (0/19)	0.5	0.0 (0/0)	2.7	74.4	13
1436	RF02453 ncr952	0.0 (0/38)	0.5	0.0 (0/0)	2.0	83.3	6
1437	RF00264 SNORA64	0.0 (0/37)	0.5	0.0 (0/0)	2.5	70.8	9
1438	RF00942 mir-1224	0.0 (0/22)	0.5	0.0 (0/0)	0.7	85.9	11
1439	RF02711 TeloSII ncR49	0.0 (0/24)	0.4	0.0 (0/0)	1.5	92.5	3
1440	RF00897 mir-675	0.0 (0/26)	0.4	0.0 (0/0)	1.8	85.2	11
1441	RF00703 mir-139	0.0 (0/24)	0.4	0.0 (0/0)	1.0	81.0	5
1442	RF00035 OxyS	0.0 (0/26)	0.4	0.0 (0/0)	1.8	90.0	5
1443	RF00692 MIR171 2	0.0 (0/26)	0.4	0.0 (0/0)	1.3	70.5	7
1444	RF00549 IRES c-sis	0.0 (0/201)	0.4	0.0 (0/1)	1.9	93.9	10
1445	RF00690 MIR408	0.0 (0/24)	0.4	0.0 (0/0)	1.4	68.4	8
1446	RF00968 mir-626	0.0 (0/26)	0.4	0.0 (0/0)	2.0	73.8	6
1447	RF02111 IS009	0.0 (0/23)	0.4	0.0 (0/0)	2.2	76.0	11
1448	RF00677 MIR168	0.0 (0/23)	0.4	0.0 (0/0)	1.8	66.8	10
1449	RF00943 MIR824	0.0 (0/53)	0.4	0.0 (0/0)	0.8	90.7	12
1450	RF00882 MIR811	0.0 (0/81)	0.4	0.0 (0/1)	1.2	81.2	5
1451	RF00710 mir-44	0.0 (0/27)	0.4	0.0 (0/0)	1.1	75.2	6
1452	RF02225 sX6	0.0 (0/68)	0.4	0.0 (0/2)	1.3	82.8	8
1453	RF00684 mir-122	0.0 (0/24)	0.4	0.0 (0/0)	0.8	92.2	26
1454	RF01044 mir-345	0.0 (0/27)	0.4	0.0 (0/0)	1.6	85.7	10
1455	RF00243 traJ 5	0.0 (0/27)	0.4	0.0 (0/0)	1.0	86.2	6
1456	RF01043 MIR1023	0.0 (0/46)	0.4	0.0 (0/0)	1.9	59.8	4
1457	RF01827 SAR11 0636	0.0 (0/23)	0.4	0.0 (0/0)	2.1	91.3	13
1458	RF02773 TrxA thermometer	0.0 (0/25)	0.4	0.0 (0/0)	1.4	87.5	8
1459	RF00258 mir-130	0.0 (0/26)	0.4	0.0 (0/0)	1.4	86.7	9
1460	RF00486 mir-129	0.0 (0/25)	0.4	0.0 (0/1)	1.0	83.0	6
1461	RF02605 scr5239	0.0 (0/46)	0.4	0.0 (0/0)	1.5	82.7	6
1462	RF02405 P34	0.0 (0/99)	0.4	0.0 (0/0)	2.9	68.0	5
1463	RF00307 snoR98	0.0 (0/24)	0.4	0.0 (0/0)	1.7	86.7	4
1464	RF02528 SSRC41	0.0 (0/25)	0.4	0.0 (0/0)	2.0	81.0	8
1465	RF02895 S414	0.0 (0/36)	0.3	0.0 (0/0)	1.6	71.5	5
1466	RF00878 mir-456	0.0 (0/32)	0.3	0.0 (0/0)	1.8	68.8	5
1467	RF00777 mir-541	0.0 (0/30)	0.3	0.0 (0/1)	2.4	82.6	10
1468	RF01635 ceN45	0.0 (0/37)	0.3	0.0 (0/0)	1.6	78.5	4
1469	RF02268 MtlS	0.0 (0/38)	0.3	0.0 (0/0)	1.9	76.4	6
1470	RF01436 S pombe snR33	0.0 (0/33)	0.3	0.0 (0/0)	1.5	68.7	3
1471	RF02673 scr4677	0.0 (0/36)	0.3	0.0 (0/1)	0.6	74.8	12
1472	RF00807 mir-314	0.0 (0/32)	0.3	0.0 (0/0)	1.2	84.1	6
1473	RF02569 IhtA	0.0 (0/34)	0.3	0.0 (0/0)	1.7	83.6	5
1474	RF01661 ceN92	0.0 (0/30)	0.3	0.0 (0/0)	1.3	75.6	4
1475	RF00740 mir-370	0.0 (0/29)	0.3	0.0 (0/0)	1.2	91.3	6
1476	RF00432 SNORA51	0.0 (0/29)	0.3	0.0 (0/0)	2.6	82.3	9
1477	RF00594 SNORD86	0.0 (0/30)	0.3	0.0 (0/0)	2.1	77.2	6
1478	RF00911 mir-672	0.0 (0/30)	0.3	0.0 (0/0)	2.4	84.2	7
1479	RF02692 BTH s39	0.0 (0/34)	0.3	0.0 (0/0)	0.8	89.4	7
1480	RF00272 SNORA67	0.0 (0/33)	0.3	0.0 (0/1)	1.9	80.2	12
1481	RF00755 mir-542	0.0 (0/29)	0.3	0.0 (0/0)	0.9	89.9	5
1482	RF01435 S pombe snR5	0.0 (0/34)	0.3	0.0 (0/0)	1.3	81.4	3
1483	RF01444 S pombe snR92	0.0 (0/36)	0.3	0.0 (0/0)	1.5	70.4	3
1484	RF01943 mir-999	0.0 (0/32)	0.3	0.0 (0/0)	2.0	65.0	4
1485	RF00937 mir-653	0.0 (0/32)	0.3	0.0 (0/0)	1.4	84.9	12
1486	RF01431 snoR135	0.0 (0/28)	0.3	0.0 (0/0)	1.3	73.5	6
1487	RF00808 mir-86	0.0 (0/37)	0.3	0.0 (0/0)	1.6	79.5	5
1488	RF00775 mir-432	0.0 (0/28)	0.3	0.0 (0/0)	1.9	73.4	6
1489	RF00766 mir-335	0.0 (0/32)	0.3	0.0 (0/0)	0.9	90.2	9
1490	RF00848 mir-61	0.0 (0/32)	0.3	0.0 (0/0)	1.5	73.1	4
1491	RF00726 mir-87	0.0 (0/30)	0.3	0.0 (0/0)	0.8	85.9	8
1492	RF00884 MIR815	0.0 (0/32)	0.3	0.0 (0/0)	1.6	79.5	3
1493	RF00680 mir-224	0.0 (0/32)	0.3	0.0 (0/0)	1.2	86.7	5
1494	RF00788 mir-287	0.0 (0/29)	0.3	0.0 (0/0)	2.0	82.1	9
1495	RF02850 Ysr276	0.0 (0/56)	0.3	0.0 (0/0)	1.6	83.6	5
1496	RF00837 mir-251	0.0 (0/34)	0.3	0.0 (0/0)	0.7	80.1	4
1497	RF01607 ceN100	0.0 (0/35)	0.3	0.0 (0/3)	1.3	79.8	4
1498	RF02409 snoR125	0.0 (0/65)	0.3	0.0 (0/0)	1.3	86.4	5
1499	RF01669 P14	0.0 (0/36)	0.3	0.0 (0/4)	1.8	71.8	4
1500	RF02245 mir-788	0.0 (0/28)	0.3	0.0 (0/0)	1.1	75.4	4
1501	RF00850 mir-259	0.0 (0/30)	0.3	0.0 (0/0)	0.8	75.3	4

Continued on next page

	RNA family (seed alignment)	Sensitivity annotated bpairs that covary % (cov_bps/bps)	Power average power %	Positive Predictive Value covarying pairs in structure % (cov_bps/cov_pairs)	average substitutions per bpair	avg pairwise identity %	number of sequences
1502	RF01261 snR82	0.0 (0/72)	0.3	0.0 (0/0)	1.8	78.6	5
1503	RF00787 mir-288	0.0 (0/34)	0.3	0.0 (0/0)	1.4	90.5	7
1504	RF01240 snR85	0.0 (0/36)	0.3	0.0 (0/0)	0.9	80.6	5
1505	RF02820 V AS9	0.0 (0/33)	0.3	0.0 (0/0)	1.2	73.5	3
1506	RF00855 mir-254	0.0 (0/34)	0.3	0.0 (0/0)	1.0	74.9	4
1507	RF01629 ceN39	0.0 (0/39)	0.2	0.0 (0/0)	0.6	90.3	4
1508	RF02855 Ysr251	0.0 (0/52)	0.2	0.0 (0/0)	1.4	77.1	5
1509	RF02404 P33	0.0 (0/45)	0.2	0.0 (0/0)	0.9	91.4	3
1510	RF02718 sca ncR14	0.0 (0/41)	0.2	0.0 (0/0)	1.0	88.8	4
1511	RF00232 Spi-1	0.0 (0/53)	0.2	0.0 (0/0)	1.2	90.9	5
1512	RF02830 Scr1601	0.0 (0/61)	0.2	0.0 (0/0)	0.8	80.1	6
1513	RF00499 Parecho CRE	0.0 (0/43)	0.2	0.0 (0/0)	1.4	87.2	5
1514	RF02872 SrbA	0.0 (0/55)	0.2	0.0 (0/0)	1.6	62.8	3
1515	RF02836 Bcj14	0.0 (0/55)	0.2	0.0 (0/0)	0.8	82.3	3
1516	RF02566 Ms1	0.0 (0/96)	0.2	0.0 (0/0)	0.9	85.3	4
1517	RF02223 sX4	0.0 (0/42)	0.2	0.0 (0/0)	2.0	85.0	5
1518	RF01674 P27	0.0 (0/56)	0.2	0.0 (0/0)	0.8	85.3	4
1519	RF01400 istR	0.0 (0/44)	0.2	0.0 (0/1)	2.0	84.1	7
1520	RF02868 ncS037	0.0 (0/49)	0.2	0.0 (0/0)	1.2	74.0	3
1521	RF01417 RSV RNA	0.0 (0/53)	0.2	0.0 (0/0)	1.3	88.5	8
1522	RF02690 BTH s1	0.0 (0/62)	0.2	0.0 (0/0)	0.9	84.3	5
1523	RF02829 Scr4115	0.0 (0/39)	0.2	0.0 (0/0)	1.0	87.8	6
1524	RF00958 mir-498	0.0 (0/43)	0.2	0.0 (0/0)	2.2	65.6	5
1525	RF01865 Vg1 ribozyme	0.0 (0/41)	0.2	0.0 (0/3)	2.3	72.7	4
1526	RF02768 Ysr155 RyfD	0.0 (0/47)	0.2	0.0 (0/0)	1.5	85.1	9
1527	RF01262 snR44	0.0 (0/55)	0.2	0.0 (0/0)	1.7	86.0	7
1528	RF02675 Ysr141	0.0 (0/80)	0.1	0.0 (0/0)	1.2	84.5	4
1529	RF01271 snR30	0.0 (0/184)	0.1	0.0 (0/0)	0.7	90.8	5
1530	RF02746 AsrC	0.0 (0/266)	0.1	0.0 (0/3)	1.4	85.4	7
1531	RF02897 S808	0.0 (0/72)	0.1	0.0 (0/0)	1.4	82.4	4
1532	RF01825 RUF21	0.0 (0/74)	0.1	0.0 (0/0)	1.5	67.6	5
1533	RF00216 IRES c-myc	0.0 (0/73)	0.1	0.0 (0/0)	1.1	98.0	23
1534	RF02400 Hsp83 3 UTR	0.0 (0/143)	0.1	0.0 (0/0)	0.6	91.2	5
1535	RF01494 rliD	0.0 (0/83)	0.1	0.0 (0/0)	0.5	96.9	9
1536	RF01272 snR86	0.0 (0/334)	0.1	0.0 (0/0)	1.5	73.9	5
1537	RF01395 isrL	0.0 (0/94)	0.1	0.0 (0/0)	1.1	84.9	4
1538	RF01264 snR83	0.0 (0/85)	0.1	0.0 (0/1)	1.1	87.1	5
1539	RF02574 Ricks sRNA10	0.0 (0/89)	0.1	0.0 (0/1)	1.0	73.2	3
1540	RF01265 snR42	0.0 (0/107)	0.1	0.0 (0/0)	1.0	84.2	5
1541	RF00224 IRES FGF2	0.0 (0/96)	0.1	0.0 (0/1)	0.9	89.5	6
1542	RF01397 isrO	0.0 (0/64)	0.1	0.0 (0/0)	2.2	84.7	6
1543	RF00908 MIR529	0.0 (0/28)	0.0	0.0 (0/0)	0.4	76.5	3
1544	RF02074 STnc240	0.0 (0/11)	0.0	0.0 (0/0)	1.9	76.5	15
1545	RF01003 mir-563	0.0 (0/28)	0.0	0.0 (0/0)	2.6	75.7	7
1546	RF00285 snoZ6	0.0 (0/4)	0.0	0.0 (0/0)	2.0	91.2	7
1547	RF00804 mir-240	0.0 (0/30)	0.0	0.0 (0/1)	0.7	69.7	3
1548	RF02159 PART1 1	0.0 (0/0)	0.0	0.0 (0/0)	0.0	78.8	22
1549	RF01441 S pombe snR46	0.0 (0/39)	0.0	0.0 (0/0)	0.0	100.0	2
1550	RF02167 PVT1 4	0.0 (0/0)	0.0	0.0 (0/0)	0.0	70.4	10
1551	RF01886 HSR-omega 2	0.0 (0/0)	0.0	0.0 (0/0)	0.0	85.1	10
1552	RF02314 TtnuHACA7	0.0 (0/32)	0.0	0.0 (0/0)	0.3	76.6	2
1553	RF01983 Pinc	0.0 (0/0)	0.0	0.0 (0/0)	0.0	84.4	16
1554	RF01946 KCNQ1OT1 1	0.0 (0/0)	0.0	0.0 (0/0)	0.0	84.0	8
1555	RF01863 TB10Cs2H2	0.0 (0/16)	0.0	0.0 (0/0)	0.0	100.0	3
1556	RF01164 SNORD107	0.0 (0/4)	0.0	0.0 (0/0)	1.2	86.2	13
1557	RF02056 STnc390	0.0 (0/15)	0.0	0.0 (0/0)	0.0	96.6	2
1558	RF02158 NPPA-AS1 3	0.0 (0/0)	0.0	0.0 (0/0)	0.0	74.7	24
1559	RF01377 CRISPR-DR64	0.0 (0/4)	0.0	0.0 (0/0)	0.0	97.3	2
1560	RF02028 mir-1827	0.0 (0/21)	0.0	0.0 (0/0)	0.9	86.3	6
1561	RF02623 BSnc140	0.0 (0/21)	0.0	0.0 (0/0)	0.0	98.9	2
1562	RF02492 Gl U2	0.0 (0/27)	0.0	0.0 (0/0)	0.0	95.6	2
1563	RF00729 mir-278	0.0 (0/27)	0.0	0.0 (0/0)	1.1	78.6	9
1564	RF01923 mir-711	0.0 (0/24)	0.0	0.0 (0/0)	0.7	82.8	5
1565	RF00502 TCV Pr	0.0 (0/8)	0.0	0.0 (0/0)	0.2	91.9	4
1566	RF02742 Rev72	0.0 (0/122)	0.0	0.0 (0/0)	0.5	86.8	3
1567	RF02874 AS-traG	0.0 (0/24)	0.0	0.0 (0/0)	1.3	76.4	4
1568	RF00635 HAR1A	0.0 (0/0)	0.0	0.0 (0/0)	0.0	92.0	13
1569	RF01041 mir-604	0.0 (0/30)	0.0	0.0 (0/0)	0.1	94.7	2
1570	RF00956 MIR1444	0.0 (0/31)	0.0	0.0 (0/0)	0.4	82.3	3
1571	RF00201 snoZ278	0.0 (0/8)	0.0	0.0 (0/0)	0.8	94.1	7
1572	RF01486 rli62	0.0 (0/33)	0.0	0.0 (0/0)	0.3	58.8	2
1573	RF02046 Sphinx 1	0.0 (0/0)	0.0	0.0 (0/0)	0.0	88.0	3
1574	RF00652 MIR478	0.0 (0/0)	0.0	0.0 (0/0)	0.0	89.6	17
1575	RF01987 ZEB2 AS1 4	0.0 (0/0)	0.0	0.0 (0/1)	0.0	90.6	13
1576	RF01842 mycoplasma FSE	0.0 (0/11)	0.0	0.0 (0/0)	0.0	94.6	3
1577	RF02240 Xoo1	0.0 (0/24)	0.0	0.0 (0/0)	1.0	85.2	5
1578	RF02098 DGCR5	0.0 (0/0)	0.0	0.0 (0/0)	0.0	87.8	4
1579	RF01795 FourU	0.0 (0/21)	0.0	0.0 (0/0)	0.0	92.3	3
1580	RF00771 mir-185	0.0 (0/34)	0.0	0.0 (0/0)	0.6	91.7	5
1581	RF02555 hveRNA	0.0 (0/31)	0.0	0.0 (0/0)	0.7	100.0	7
1582	RF01531 TB10Cs3H1	0.0 (0/18)	0.0	0.0 (0/0)	0.8	80.7	4
1583	RF00384 Pox AX element	0.0 (0/22)	0.0	0.0 (0/0)	0.4	93.4	10
1584	RF01279 snoR53Y	0.0 (0/5)	0.0	0.0 (0/0)	1.2	71.8	7
1585	RF00762 mir-412	0.0 (0/34)	0.0	0.0 (0/0)	0.8	90.7	9

Continued on next page

	RNA family (seed alignment)	Sensitivity annotated bpairs that covary % (cov_bps/bps)	Power average power %	Positive Predictive Value covarying pairs in structure % (cov_bps/cov_pairs)	average substitutions per bpair	avg pairwise identity %	number of sequences
1586	RF02019 mir-1265	0.0 (0/35)	0.0	0.0 (0/0)	0.1	95.5	3
1587	RF00046 snoR30	0.0 (0/4)	0.0	0.0 (0/0)	1.5	87.0	6
1588	RF02247 Six3os1 2	0.0 (0/0)	0.0	0.0 (0/0)	0.0	85.6	15
1589	RF01330 CRISPR-DR16	0.0 (0/7)	0.0	0.0 (0/0)	0.7	87.0	6
1590	RF02439 SpF56 sRNA	0.0 (0/24)	0.0	0.0 (0/0)	0.0	94.9	2
1591	RF01601 plasmodium snoR26	0.0 (0/8)	0.0	0.0 (0/0)	0.0	92.8	3
1592	RF02499 Atu C4	0.0 (0/21)	0.0	0.0 (0/0)	0.0	98.6	2
1593	RF02782 CpoB ybgF thermometer	0.0 (0/38)	0.0	0.0 (0/0)	0.9	91.5	8
1594	RF01626 ceN30	0.0 (0/3)	0.0	0.0 (0/0)	0.0	89.8	4
1595	RF02766 Ysr49	0.0 (0/30)	0.0	0.0 (0/0)	0.4	93.5	3
1596	RF01007 mir-624	0.0 (0/38)	0.0	0.0 (0/0)	0.0	96.9	2
1597	RF02762 C1 109596F thermometer	0.0 (0/60)	0.0	0.0 (0/0)	0.0	98.7	2
1598	RF01423 snoR117	0.0 (0/4)	0.0	0.0 (0/0)	1.5	69.3	6
1599	RF02669 PsiU6-40	0.0 (0/46)	0.0	0.0 (0/0)	0.1	96.2	3
1600	RF01585 snoR07	0.0 (0/3)	0.0	0.0 (0/0)	0.0	85.9	4
1601	RF02783 IscS1 thermometer	0.0 (0/46)	0.0	0.0 (0/0)	1.4	78.6	5
1602	RF01355 CRISPR-DR26	0.0 (0/3)	0.0	0.0 (0/0)	0.0	93.3	4
1603	RF01125 sR4	0.0 (0/0)	0.0	0.0 (0/0)	0.0	86.9	3
1604	RF02859 Arrc11	0.0 (0/53)	0.0	0.0 (0/0)	0.2	77.2	2
1605	RF01132 sR48	0.0 (0/0)	0.0	0.0 (0/0)	0.0	76.4	2
1606	RF02233 sX14	0.0 (0/27)	0.0	0.0 (0/0)	0.1	96.0	8
1607	RF01551 TB8Cs4H2	0.0 (0/15)	0.0	0.0 (0/0)	0.1	69.1	2
1608	RF02292 TtnuCD16	0.0 (0/0)	0.0	0.0 (0/0)	0.0	96.5	2
1609	RF01298 snoU25	0.0 (0/9)	0.0	0.0 (0/0)	0.0	98.1	3
1610	RF02724 sno ZL2	0.0 (0/25)	0.0	0.0 (0/0)	1.2	86.5	6
1611	RF02058 STnc400	0.0 (0/43)	0.0	0.0 (0/0)	0.5	65.3	2
1612	RF02482 GlsR19	0.0 (0/21)	0.0	0.0 (0/0)	0.8	83.5	3
1613	RF01159 snoU18	0.0 (0/0)	0.0	0.0 (0/0)	0.0	82.1	16
1614	RF00990 mir-552	0.0 (0/34)	0.0	0.0 (0/0)	0.0	92.6	2
1615	RF02256 adapt33 2	0.0 (0/0)	0.0	0.0 (0/0)	0.0	82.6	2
1616	RF01283 snoU30	0.0 (0/2)	0.0	0.0 (0/0)	0.0	74.8	5
1617	RF00571 SNORD65	0.0 (0/0)	0.0	0.0 (0/0)	0.0	82.6	26
1618	RF02567 Va-907	0.0 (0/100)	0.0	0.0 (0/0)	0.2	95.9	3
1619	RF02048 STnc30	0.0 (0/33)	0.0	0.0 (0/0)	0.1	94.7	2
1620	RF02710 TeloSII ncR45	0.0 (0/26)	0.0	0.0 (0/0)	0.0	98.5	2
1621	RF01425 snoR121	0.0 (0/3)	0.0	0.0 (0/0)	0.0	88.2	2
1622	RF02181 ST7-AS2 1	0.0 (0/0)	0.0	0.0 (0/0)	0.0	89.9	4
1623	RF00610 SNORD110	0.0 (0/4)	0.0	0.0 (0/0)	1.0	87.7	16
1624	RF01477 rli43	0.0 (0/69)	0.0	0.0 (0/0)	0.4	93.5	4
1625	RF02587 HCV package-SL4629	0.0 (0/7)	0.0	0.0 (0/0)	0.0	95.7	2
1626	RF01483 rli56	0.0 (0/31)	0.0	0.0 (0/0)	0.0	97.5	6
1627	RF02129 GNAS-AS1 3	0.0 (0/0)	0.0	0.0 (0/0)	0.0	83.8	9
1628	RF01083 UPD-PK2	0.0 (0/8)	0.0	0.0 (0/0)	0.6	95.2	4
1629	RF01988 SECIS 2	0.0 (0/20)	0.0	0.0 (0/0)	0.8	89.1	4
1630	RF00039 DicF	0.0 (0/11)	0.0	0.0 (0/0)	0.5	74.6	5
1631	RF02839 Ref66	0.0 (0/34)	0.0	0.0 (0/0)	0.1	80.2	2
1632	RF02657 sot2652	0.0 (0/58)	0.0	0.0 (0/0)	0.2	86.0	2
1633	RF02763 IsrM	0.0 (0/81)	0.0	0.0 (0/0)	0.1	100.0	2
1634	RF02660 icaR 3p UTR	0.0 (0/126)	0.0	0.0 (0/0)	0.0	99.7	3
1635	RF01514 Afu 513	0.0 (0/0)	0.0	0.0 (0/0)	0.0	77.4	7
1636	RF02372 PyrC leader	0.0 (0/5)	0.0	0.0 (0/0)	0.2	81.7	9
1637	RF02863 sRNA 2410	0.0 (0/57)	0.0	0.0 (0/0)	0.7	74.7	3
1638	RF01210 snoU13	0.0 (0/0)	0.0	0.0 (0/0)	0.0	78.1	34
1639	RF02568 UptR	0.0 (0/20)	0.0	0.0 (0/0)	0.2	96.3	3
1640	RF01896 mir-142	0.0 (0/31)	0.0	0.0 (0/0)	0.1	93.4	13
1641	RF02556 snaR-A	0.0 (0/38)	0.0	0.0 (0/0)	0.3	95.8	5
1642	RF02328 TtnuHACA21	0.0 (0/34)	0.0	0.0 (0/0)	0.1	95.3	2
1643	RF01367 CRISPR-DR54	0.0 (0/6)	0.0	0.0 (0/0)	0.0	97.3	2
1644	RF02062 STnc361	0.0 (0/35)	0.0	0.0 (0/0)	0.2	79.9	2
1645	RF02072 STnc590	0.0 (0/24)	0.0	0.0 (0/0)	0.4	96.2	3
1646	RF01944 mir-2518	0.0 (0/29)	0.0	0.0 (0/0)	0.2	97.2	4
1647	RF01579 RUF2	0.0 (0/42)	0.0	0.0 (0/0)	0.4	79.1	3
1648	RF00758 mir-346	0.0 (0/27)	0.0	0.0 (0/0)	0.2	93.2	5
1649	RF02316 TtnuHACA9	0.0 (0/37)	0.0	0.0 (0/2)	0.5	79.3	3
1650	RF02291 TtnuCD15	0.0 (0/0)	0.0	0.0 (0/0)	0.0	88.5	2
1651	RF01166 sn3071	0.0 (0/4)	0.0	0.0 (0/0)	0.0	94.7	2
1652	RF01500 Afu 198	0.0 (0/0)	0.0	0.0 (0/0)	0.0	57.3	6
1653	RF00259 IFN gamma	0.0 (0/56)	0.0	0.0 (0/0)	0.6	90.4	5
1654	RF02018 mir-1207	0.0 (0/28)	0.0	0.0 (0/0)	0.1	97.7	3
1655	RF01286 snoR26	0.0 (0/7)	0.0	0.0 (0/0)	0.4	74.1	8
1656	RF01442 S pombe snR90	0.0 (0/36)	0.0	0.0 (0/0)	1.2	80.8	3
1657	RF02406 snoR120	0.0 (0/0)	0.0	0.0 (0/0)	0.0	87.7	4
1658	RF00268 snoZ7	0.0 (0/2)	0.0	0.0 (0/0)	0.0	90.5	5
1659	RF02390 sau-41	0.0 (0/35)	0.0	0.0 (0/0)	0.0	94.2	2
1660	RF00862 mir-491	0.0 (0/33)	0.0	0.0 (0/0)	1.0	88.8	5
1661	RF01303 sR49	0.0 (0/11)	0.0	0.0 (0/0)	0.0	87.5	3
1662	RF02351 psRNA14	0.0 (0/35)	0.0	0.0 (0/0)	1.7	58.2	3
1663	RF01172 sn2343	0.0 (0/4)	0.0	0.0 (0/0)	0.0	88.5	3
1664	RF00681 mir-198	0.0 (0/22)	0.0	0.0 (0/0)	0.3	90.3	3
1665	RF01281 snoR35	0.0 (0/5)	0.0	0.0 (0/0)	2.4	69.9	12
1666	RF02582 tsr33	0.0 (0/20)	0.0	0.0 (0/0)	0.1	95.3	2
1667	RF02815 Lig thermometer	0.0 (0/57)	0.0	0.0 (0/0)	0.0	99.5	2
1668	RF00877 mir-592	0.0 (0/34)	0.0	0.0 (0/0)	0.7	89.1	5
1669	RF01307 sR55	0.0 (0/2)	0.0	0.0 (0/0)	0.0	87.1	4

Continued on next page

	RNA family (seed alignment)	Sensitivity annotated bpairs that covary % (cov_bps/bps)	Power average power %	Positive Predictive Value covarying pairs in structure % (cov_bps/cov_pairs)	average substitutions per bpair	avg pairwise identity %	number of sequences
1670	RF02321 TtnuHACA14	0.0 (0/36)	0.0	0.0 (0/0)	0.2	92.0	3
1671	RF01665 P13	0.0 (0/26)	0.0	0.0 (0/0)	0.0	88.2	2
1672	RF02563 CbSR3	0.0 (0/51)	0.0	0.0 (0/0)	0.0	99.5	2
1673	RF00383 IS1222 FSE	0.0 (0/17)	0.0	0.0 (0/0)	0.8	92.7	5
1674	RF02202 UCA1	0.0 (0/0)	0.0	0.0 (0/0)	0.0	94.3	5
1675	RF02170 PVT1 7	0.0 (0/0)	0.0	0.0 (0/0)	0.0	65.0	8
1676	RF02394 sau-63	0.0 (0/26)	0.0	0.0 (0/0)	0.0	98.6	3
1677	RF00122 GadY	0.0 (0/28)	0.0	0.0 (0/0)	0.1	99.0	5
1678	RF01277 snoU54	0.0 (0/3)	0.0	0.0 (0/0)	0.0	77.6	28
1679	RF02184 ST7-OT3 2	0.0 (0/0)	0.0	0.0 (0/0)	0.0	91.6	5
1680	RF01568 DdR18	0.0 (0/37)	0.0	0.0 (0/0)	1.2	80.0	3
1681	RF02255 adapt33 1	0.0 (0/0)	0.0	0.0 (0/0)	0.0	77.3	3
1682	RF00932 mir-471	0.0 (0/25)	0.0	0.0 (0/0)	0.0	97.4	2
1683	RF00980 mir-643	0.0 (0/31)	0.0	0.0 (0/0)	0.0	90.7	2
1684	RF00931 mir-879	0.0 (0/27)	0.0	0.0 (0/0)	0.0	96.0	2
1685	RF02589 Spy779816	0.0 (0/26)	0.0	0.0 (0/0)	1.1	79.3	4
1686	RF01427 snoR127	0.0 (0/4)	0.0	0.0 (0/0)	0.0	79.6	3
1687	RF01562 DdR12	0.0 (0/0)	0.0	0.0 (0/0)	0.0	100.0	2
1688	RF00044 Phage pRNA	0.0 (0/43)	0.0	0.0 (0/0)	0.1	97.5	3
1689	RF02241 Xoo2	0.0 (0/20)	0.0	0.0 (0/0)	0.6	92.2	3
1690	RF02884 BcKCsr7	0.0 (0/35)	0.0	0.0 (0/0)	0.8	84.6	5
1691	RF02656 sot0042	0.0 (0/53)	0.0	0.0 (0/0)	0.0	92.5	2
1692	RF01128 sR43	0.0 (0/0)	0.0	0.0 (0/0)	0.0	86.0	6
1693	RF01117 ciona-mir-92	0.0 (0/32)	0.0	0.0 (0/0)	0.2	75.3	2
1694	RF01902 MIR439	0.0 (0/37)	0.0	0.0 (0/0)	0.5	95.8	5
1695	RF02248 Six3os1 3	0.0 (0/0)	0.0	0.0 (0/0)	0.0	82.8	10
1696	RF00606 SNORD93	0.0 (0/4)	0.0	0.0 (0/0)	0.0	88.7	17
1697	RF01464 rliA	0.0 (0/63)	0.0	0.0 (0/0)	0.2	94.6	4
1698	RF00058 HgcF	0.0 (0/38)	0.0	0.0 (0/0)	0.3	87.8	4
1699	RF02667 PsiU2-38.40.42	0.0 (0/34)	0.0	0.0 (0/0)	0.2	86.0	3
1700	RF01828 SprD	0.0 (0/47)	0.0	0.0 (0/0)	0.0	100.0	2
1701	RF01321 CRISPR-DR8	0.0 (0/7)	0.0	0.0 (0/0)	0.9	70.3	5
1702	RF00843 mir-228	0.0 (0/35)	0.0	0.0 (0/0)	0.5	84.1	4
1703	RF01175 snoU83D	0.0 (0/2)	0.0	0.0 (0/0)	3.5	85.9	8
1704	RF01979 HOTAIRM1 5	0.0 (0/0)	0.0	0.0 (0/0)	0.0	88.9	10
1705	RF00834 mir-268	0.0 (0/28)	0.0	0.0 (0/0)	2.2	80.3	4
1706	RF02216 ZNFX1-AS1 2	0.0 (0/0)	0.0	0.0 (0/0)	0.0	65.8	18
1707	RF02050 STnc470	0.0 (0/43)	0.0	0.0 (0/0)	0.2	78.2	2
1708	RF01468 rli32	0.0 (0/47)	0.0	0.0 (0/0)	0.7	93.9	5
1709	RF00084 CsrC	0.0 (0/59)	0.0	0.0 (0/2)	1.0	82.7	4
1710	RF02148 MESTIT1 1	0.0 (0/0)	0.0	0.0 (0/0)	0.0	71.9	16
1711	RF01336 CRISPR-DR23	0.0 (0/8)	0.0	0.0 (0/0)	0.0	92.6	3
1712	RF01513 Afu 335	0.0 (0/0)	0.0	0.0 (0/0)	0.0	76.5	5
1713	RF01662 ceN89	0.0 (0/4)	0.0	0.0 (0/1)	1.5	84.7	4
1714	RF02627 Ssr1	0.0 (0/203)	0.0	0.0 (0/0)	0.0	100.0	2
1715	RF01659 ceN86	0.0 (0/31)	0.0	0.0 (0/0)	0.1	77.9	2
1716	RF00915 mir-760	0.0 (0/26)	0.0	0.0 (0/0)	1.3	89.7	7
1717	RF02751 ES036	0.0 (0/8)	0.0	0.0 (0/0)	0.8	90.7	4
1718	RF02726 sno ZL63	0.0 (0/11)	0.0	0.0 (0/0)	0.5	93.8	3
1719	RF01596 plasmodium snoR20	0.0 (0/0)	0.0	0.0 (0/0)	0.0	86.2	2
1720	RF01163 snoR64a	0.0 (0/0)	0.0	0.0 (0/0)	0.0	76.2	4
1721	RF00821 mir-249	0.0 (0/38)	0.0	0.0 (0/0)	0.8	70.9	4
1722	RF01389 isrF	0.0 (0/39)	0.0	0.0 (0/0)	0.8	85.4	3
1723	RF01582 RUF4	0.0 (0/90)	0.0	0.0 (0/0)	0.0	96.0	2
1724	RF01378 CRISPR-DR65	0.0 (0/7)	0.0	0.0 (0/0)	0.0	86.5	2
1725	RF00196 AMV RNA1 SL	0.0 (0/12)	0.0	0.0 (0/0)	0.8	95.8	6
1726	RF01037 mir-644	0.0 (0/28)	0.0	0.0 (0/0)	0.1	92.5	3
1727	RF00854 mir-5	0.0 (0/26)	0.0	0.0 (0/0)	0.2	91.2	6
1728	RF01614 ceN110	0.0 (0/29)	0.0	0.0 (0/0)	0.4	87.2	3
1729	RF02557 CbSR1	0.0 (0/29)	0.0	0.0 (0/0)	0.0	100.0	2
1730	RF00336 snoJ26	0.0 (0/14)	0.0	0.0 (0/0)	0.1	95.1	5
1731	RF02168 PVT1 5	0.0 (0/0)	0.0	0.0 (0/0)	0.0	69.2	23
1732	RF00317 snoZ163	0.0 (0/5)	0.0	0.0 (0/0)	0.2	81.7	7
1733	RF02099 rivX	0.0 (0/66)	0.0	0.0 (0/0)	0.0	97.2	2
1734	RF00366 mir-BHRF1-2	0.0 (0/25)	0.0	0.0 (0/0)	1.3	84.2	5
1735	RF01484 rli59	0.0 (0/44)	0.0	0.0 (0/0)	0.2	96.2	5
1736	RF01814 rhtB	0.0 (0/13)	0.0	0.0 (0/0)	0.8	76.0	14
1737	RF01275 sR22	0.0 (0/12)	0.0	0.0 (0/0)	1.2	92.0	6
1738	RF02106 DLEU2 2	0.0 (0/0)	0.0	0.0 (0/0)	0.0	78.2	18
1739	RF00060 HgcE	0.0 (0/20)	0.0	0.0 (0/0)	0.8	79.0	4
1740	RF01969 RMST 8	0.0 (0/0)	0.0	0.0 (0/0)	0.0	81.7	22
1741	RF00511 IRES KSHV	0.0 (0/61)	0.0	0.0 (0/0)	0.0	99.3	5
1742	RF02607 AbsR25	0.0 (0/47)	0.0	0.0 (0/0)	0.0	99.4	2
1743	RF00969 mir-556	0.0 (0/29)	0.0	0.0 (0/0)	0.1	83.2	2
1744	RF02177 SMCR2 1	0.0 (0/0)	0.0	0.0 (0/0)	0.0	68.5	6
1745	RF01345 CRISPR-DR35	0.0 (0/8)	0.0	0.0 (0/0)	0.1	97.2	2
1746	RF02787 snoTBR4	0.0 (0/26)	0.0	0.0 (0/2)	1.2	77.3	3
1747	RF02577 tsr24	0.0 (0/68)	0.0	0.0 (0/0)	0.1	98.2	4
1748	RF02448 SpR20 sRNA	0.0 (0/11)	0.0	0.0 (0/0)	0.7	89.8	7
1749	RF00421 SNORA32	0.0 (0/23)	0.0	0.0 (0/0)	1.4	83.7	9
1750	RF01088 TLS-PK5	0.0 (0/23)	0.0	0.0 (0/0)	0.7	80.6	3
1751	RF01552 TB9Cs1H2	0.0 (0/8)	0.0	0.0 (0/0)	0.1	78.8	5
1752	RF01034 mir-618	0.0 (0/36)	0.0	0.0 (0/0)	0.4	94.7	5
1753	RF02600 BASRCI27	0.0 (0/57)	0.0	0.0 (0/0)	0.1	98.8	3

Continued on next page

	RNA family (seed alignment)	Sensitivity annotated bpairs that covary % (cov_bps/bps)	Power average power %	Positive Predictive Value covarying pairs in structure % (cov_bps/cov_pairs)	average substitutions per bpair	avg pairwise identity %	number of sequences
1754	RF01885 HSR-omega 1	0.0 (0/0)	0.0	0.0 (0/0)	0.0	92.6	13
1755	RF02598 EBv-sisRNA-2	0.0 (0/31)	0.0	0.0 (0/0)	0.1	94.5	2
1756	RF01098 RF site9	0.0 (0/12)	0.0	0.0 (0/0)	0.1	91.8	2
1757	RF01092 GP knot2	0.0 (0/14)	0.0	0.0 (0/0)	0.0	100.0	2
1758	RF02305 TtnuCD31	0.0 (0/0)	0.0	0.0 (0/0)	0.0	97.0	2
1759	RF02360 Yfr8	0.0 (0/63)	0.0	0.0 (0/0)	0.7	92.0	5
1760	RF00780 MIR477	0.0 (0/29)	0.0	0.0 (0/0)	1.0	61.9	3
1761	RF02498 Atu C3	0.0 (0/53)	0.0	0.0 (0/0)	0.0	92.9	2
1762	RF01113 BMV3 UPD-PK3	0.0 (0/7)	0.0	0.0 (0/0)	0.0	95.7	2
1763	RF00976 mir-583	0.0 (0/26)	0.0	0.0 (0/0)	0.2	96.4	3
1764	RF01030 mir-422	0.0 (0/33)	0.0	0.0 (0/0)	1.0	78.0	3
1765	RF02297 TtnuCD21	0.0 (0/0)	0.0	0.0 (0/0)	0.0	82.5	2
1766	RF01900 mir-2024	0.0 (0/30)	0.0	0.0 (0/0)	0.3	88.8	7
1767	RF01459 rliE	0.0 (0/48)	0.0	0.0 (0/1)	0.5	82.7	4
1768	RF02369 h2cR	0.0 (0/51)	0.0	0.0 (0/0)	1.5	87.9	7
1769	RF01487 rliI	0.0 (0/81)	0.0	0.0 (0/0)	0.1	98.3	5
1770	RF01679 P36	0.0 (0/21)	0.0	0.0 (0/0)	0.0	98.4	2
1771	RF02777 OppA thermometer	0.0 (0/82)	0.0	0.0 (0/0)	0.0	99.6	3
1772	RF01107 SBRMV1 UPD-PKf	0.0 (0/8)	0.0	0.0 (0/0)	0.0	100.0	2
1773	RF02073 STnc260	0.0 (0/36)	0.0	0.0 (0/0)	0.1	92.6	2
1774	RF02085 Yar 1	0.0 (0/0)	0.0	0.0 (0/0)	0.0	77.6	10
1775	RF00988 mir-657	0.0 (0/19)	0.0	0.0 (0/0)	0.1	89.8	2
1776	RF00817 mir-80	0.0 (0/34)	0.0	0.0 (0/0)	0.1	89.5	4
1777	RF02138 HOXA11-AS1 2	0.0 (0/0)	0.0	0.0 (0/0)	0.0	83.0	21
1778	RF02199 TTC28-AS1 2	0.0 (0/0)	0.0	0.0 (0/0)	0.0	71.4	8
1779	RF01379 CRISPR-DR66	0.0 (0/7)	0.0	0.0 (0/0)	0.4	94.1	4
1780	RF02901 AaHKsRNA41	0.0 (0/51)	0.0	0.0 (0/0)	0.4	46.4	2
1781	RF02293 TtnuCD17	0.0 (0/0)	0.0	0.0 (0/0)	0.0	97.0	2
1782	RF02258 adapt33 4	0.0 (0/0)	0.0	0.0 (0/0)	0.0	74.8	2
1783	RF02712 EBER2	0.0 (0/47)	0.0	0.0 (0/0)	0.0	97.3	3
1784	RF01216 snR87	0.0 (0/2)	0.0	0.0 (0/0)	0.5	89.9	4
1785	RF02640 MOSES4	0.0 (0/46)	0.0	0.0 (0/0)	0.0	99.2	3
1786	RF01091 PK-SPCSV	0.0 (0/17)	0.0	0.0 (0/0)	0.0	100.0	2
1787	RF02290 TtnuCD14	0.0 (0/0)	0.0	0.0 (0/0)	0.0	93.2	2
1788	RF01222 sn2417	0.0 (0/2)	0.0	0.0 (0/0)	0.5	92.3	3
1789	RF01308 sR58	0.0 (0/2)	0.0	0.0 (0/0)	0.5	87.1	5
1790	RF01129 sR44	0.0 (0/0)	0.0	0.0 (0/0)	0.0	84.8	3
1791	RF02218 ZNRD1-AS1 1	0.0 (0/0)	0.0	0.0 (0/0)	0.0	83.7	8
1792	RF01632 ceN42	0.0 (0/35)	0.0	0.0 (0/0)	0.4	79.1	3
1793	RF01359 CRISPR-DR46	0.0 (0/7)	0.0	0.0 (0/0)	0.0	84.2	2
1794	RF02717 sno ncR4	0.0 (0/24)	0.0	0.0 (0/0)	0.3	88.6	3
1795	RF02415 rliG	0.0 (0/56)	0.0	0.0 (0/0)	1.3	72.8	5
1796	RF02539 SNOR75	0.0 (0/4)	0.0	0.0 (0/0)	0.8	86.9	6
1797	RF01001 mir-609	0.0 (0/34)	0.0	0.0 (0/0)	0.1	94.7	2
1798	RF02795 Pab91	0.0 (0/19)	0.0	0.0 (0/0)	0.2	91.2	3
1799	RF01409 STnc250	0.0 (0/25)	0.0	0.0 (0/0)	0.1	97.9	3
1800	RF00603 SNORD23	0.0 (0/5)	0.0	0.0 (0/0)	1.6	83.8	15
1801	RF02281 TtnuCD4	0.0 (0/0)	0.0	0.0 (0/0)	0.0	82.2	3
1802	RF02087 Yar 3	0.0 (0/0)	0.0	0.0 (0/0)	0.0	87.8	4
1803	RF02368 Yfr21	0.0 (0/43)	0.0	0.0 (0/0)	0.2	88.7	2
1804	RF01109 SBRMV1 UPD-PKd	0.0 (0/8)	0.0	0.0 (0/0)	0.0	100.0	2
1805	RF02244 mir-785	0.0 (0/31)	0.0	0.0 (0/0)	0.9	70.8	4
1806	RF02284 TtnuCD7	0.0 (0/0)	0.0	0.0 (0/0)	0.0	94.4	2
1807	RF01028 mir-633	0.0 (0/19)	0.0	0.0 (0/0)	1.1	76.0	3
1808	RF00922 mir-673	0.0 (0/26)	0.0	0.0 (0/0)	0.1	97.4	2
1809	RF02639 EF0869 EF0870	0.0 (0/140)	0.0	0.0 (0/0)	0.0	99.5	4
1810	RF02811 FHbp thermometer	0.0 (0/18)	0.0	0.0 (0/0)	0.1	94.5	2
1811	RF02283 TtnuCD6	0.0 (0/0)	0.0	0.0 (0/0)	0.0	78.6	2
1812	RF02185 ST7-OT3 3	0.0 (0/0)	0.0	0.0 (0/0)	0.0	71.2	21
1813	RF01467 rli36	0.0 (0/23)	0.0	0.0 (0/0)	0.3	95.2	6
1814	RF01889 lincRNA-p21 1	0.0 (0/0)	0.0	0.0 (0/0)	0.0	88.1	2
1815	RF02179 ST7-AS1 1	0.0 (0/0)	0.0	0.0 (0/0)	0.0	76.6	25
1816	RF00212 SNORD38	0.0 (0/8)	0.0	0.0 (0/1)	1.2	80.8	7
1817	RF01201 snR40	0.0 (0/0)	0.0	0.0 (0/0)	0.0	76.9	18
1818	RF02642 Spy491311c	0.0 (0/41)	0.0	0.0 (0/0)	0.0	100.0	2
1819	RF01136 sR28	0.0 (0/0)	0.0	0.0 (0/0)	0.0	71.0	6
1820	RF00981 mir-939	0.0 (0/31)	0.0	0.0 (0/0)	0.0	99.2	3
1821	RF00874 mir-BART12	0.0 (0/31)	0.0	0.0 (0/0)	0.2	71.6	2
1822	RF01981 PCGEM1	0.0 (0/0)	0.0	0.0 (0/0)	0.0	86.1	26
1823	RF02110 DLEU2 6	0.0 (0/0)	0.0	0.0 (0/0)	0.0	71.5	32
1824	RF01818 RsaC	0.0 (0/116)	0.0	0.0 (0/0)	0.0	99.8	3
1825	RF00182 Corona package	0.0 (0/30)	0.0	0.0 (0/0)	1.0	78.3	3
1826	RF00277 SNORD49	0.0 (0/0)	0.0	0.0 (0/0)	0.0	76.9	28
1827	RF00192 BLV package	0.0 (0/12)	0.0	0.0 (0/0)	0.1	96.4	5
1828	RF02009 mir-987	0.0 (0/31)	0.0	0.0 (0/0)	0.0	100.0	3
1829	RF01938 mir-1251	0.0 (0/22)	0.0	0.0 (0/0)	0.0	99.4	4
1830	RF01606 plasmodium snoR31	0.0 (0/36)	0.0	0.0 (0/0)	0.4	80.9	3
1831	RF02644 Spy490380c	0.0 (0/23)	0.0	0.0 (0/0)	0.7	88.5	4
1832	RF02257 adapt33 3	0.0 (0/0)	0.0	0.0 (0/0)	0.0	73.7	3
1833	RF00363 mir-BART1	0.0 (0/24)	0.0	0.0 (0/0)	0.2	94.3	5
1834	RF02068 STnc480	0.0 (0/8)	0.0	0.0 (0/0)	0.1	82.4	6
1835	RF01332 CRISPR-DR19	0.0 (0/8)	0.0	0.0 (0/0)	0.2	77.6	4
1836	RF00945 mir-1226	0.0 (0/28)	0.0	0.0 (0/0)	0.1	86.7	2
1837	RF00301 snoZ256	0.0 (0/5)	0.0	0.0 (0/0)	1.4	83.2	5

Continued on next page

	RNA family (seed alignment)	Sensitivity annotated bpairs that covary % (cov_bps/bps)	Power average power %	Positive Predictive Value covarying pairs in structure % (cov_bps/cov_pairs)	average substitutions per bpair	avg pairwise identity %	number of sequences
1838	RF02609 BSR0602	0.0 (0/53)	0.0	0.0 (0/0)	0.0	98.8	2
1839	RF00721 MIR475	0.0 (0/41)	0.0	0.0 (0/0)	1.1	87.7	4
1840	RF02501 Atu C7	0.0 (0/26)	0.0	0.0 (0/0)	0.0	90.5	3
1841	RF01199 snR58	0.0 (0/0)	0.0	0.0 (0/0)	0.0	81.3	3
1842	RF02392 sau-53	0.0 (0/56)	0.0	0.0 (0/0)	0.7	89.5	4
1843	RF02606 AbsR28	0.0 (0/54)	0.0	0.0 (0/0)	0.1	98.3	2
1844	RF02104 DLEU1 2	0.0 (0/0)	0.0	0.0 (0/0)	0.0	73.3	16
1845	RF00814 mir-316	0.0 (0/35)	0.0	0.0 (0/0)	0.3	92.4	4
1846	RF02124 JPX 1	0.0 (0/0)	0.0	0.0 (0/0)	0.0	71.1	14
1847	RF01493 rli37	0.0 (0/57)	0.0	0.0 (0/0)	0.2	96.0	4
1848	RF01462 rli26	0.0 (0/47)	0.0	0.0 (0/0)	0.4	94.1	5
1849	RF01498 Afu 190	0.0 (0/0)	0.0	0.0 (0/0)	0.0	59.8	3
1850	RF01119 sR32	0.0 (0/0)	0.0	0.0 (0/0)	0.0	88.9	3
1851	RF02752 ES056	0.0 (0/28)	0.0	0.0 (0/0)	0.9	94.1	6
1852	RF01465 rli31	0.0 (0/31)	0.0	0.0 (0/0)	0.8	89.0	3
1853	RF01399 isrQ	0.0 (0/50)	0.0	0.0 (0/0)	0.2	97.4	6
1854	RF02904 AaHKsRNA69	0.0 (0/21)	0.0	0.0 (0/0)	0.7	71.6	3
1855	RF02592 Spy392987	0.0 (0/21)	0.0	0.0 (0/0)	0.0	95.7	3
1856	RF01822 RsaJ	0.0 (0/79)	0.0	0.0 (0/0)	0.1	97.2	3
1857	RF01105 SBWMV2 UPD-PKl	0.0 (0/9)	0.0	0.0 (0/0)	0.0	100.0	2
1858	RF02418 Spd-sr07	0.0 (0/16)	0.0	0.0 (0/0)	0.2	98.9	3
1859	RF00926 MIR1151	0.0 (0/61)	0.0	0.0 (0/0)	0.1	88.2	2
1860	RF01157 sn1185	0.0 (0/3)	0.0	0.0 (0/0)	0.0	92.1	5
1861	RF01457 rli22	0.0 (0/23)	0.0	0.0 (0/0)	0.0	98.0	6
1862	RF02172 RFPL3-AS1 2	0.0 (0/0)	0.0	0.0 (0/0)	0.0	81.7	3
1863	RF01888 DLX6-AS1 2	0.0 (0/0)	0.0	0.0 (0/0)	0.0	87.9	19
1864	RF01858 RsaF	0.0 (0/38)	0.0	0.0 (0/0)	0.1	98.6	4
1865	RF01587 snoR10	0.0 (0/4)	0.0	0.0 (0/0)	0.2	91.2	4
1866	RF01992 VIS1	0.0 (0/0)	0.0	0.0 (0/0)	0.0	86.3	27
1867	RF00741 mir-378	0.0 (0/24)	0.0	0.0 (0/0)	0.1	99.3	8
1868	RF01347 CRISPR-DR37	0.0 (0/7)	0.0	0.0 (0/0)	0.1	78.1	3
1869	RF02436 SpF43 sRNA	0.0 (0/34)	0.0	0.0 (0/0)	1.1	85.5	5
1870	RF02017 mir-1912	0.0 (0/26)	0.0	0.0 (0/0)	0.7	91.4	13
1871	RF00847 mir-62	0.0 (0/22)	0.0	0.0 (0/0)	0.3	92.2	4
1872	RF02123 FTX 4	0.0 (0/0)	0.0	0.0 (0/0)	0.0	79.4	13
1873	RF02086 Yar 2	0.0 (0/0)	0.0	0.0 (0/0)	0.0	87.7	7
1874	RF02788 snoTBR6	0.0 (0/13)	0.0	0.0 (0/0)	0.2	72.1	3
1875	RF02628 Hrs1	0.0 (0/20)	0.0	0.0 (0/0)	0.1	86.5	2
1876	RF01120 sR33	0.0 (0/0)	0.0	0.0 (0/0)	0.0	75.5	3
1877	RF01211 SNORD124	0.0 (0/7)	0.0	0.0 (0/0)	0.3	80.8	18
1878	RF02203 WT1-AS 1	0.0 (0/0)	0.0	0.0 (0/0)	0.0	86.6	10
1879	RF01506 Afu 294	0.0 (0/0)	0.0	0.0 (0/0)	0.0	71.8	10
1880	RF00953 mir-1497	0.0 (0/16)	0.0	0.0 (0/0)	0.0	82.2	2
1881	RF01424 snoR118	0.0 (0/0)	0.0	0.0 (0/0)	0.0	75.0	8
1882	RF02614 BSR1073	0.0 (0/67)	0.0	0.0 (0/0)	0.0	99.5	2
1883	RF02736 Rev11	0.0 (0/111)	0.0	0.0 (0/0)	0.2	89.0	3
1884	RF00327 snoZ194	0.0 (0/19)	0.0	0.0 (0/0)	0.0	98.8	5
1885	RF01373 CRISPR-DR60	0.0 (0/3)	0.0	0.0 (0/0)	0.0	83.8	2
1886	RF02416 TCV H4	0.0 (0/9)	0.0	0.0 (0/0)	0.1	84.0	2
1887	RF02759 LasI thermometer	0.0 (0/31)	0.0	0.0 (0/0)	0.0	97.0	3
1888	RF00856 mir-232	0.0 (0/29)	0.0	0.0 (0/0)	0.5	77.9	3
1889	RF00699 mir-134	0.0 (0/24)	0.0	0.0 (0/0)	1.1	87.8	6
1890	RF02713 MCS4	0.0 (0/31)	0.0	0.0 (0/0)	0.1	92.6	5
1891	RF02473 GlsR4	0.0 (0/0)	0.0	0.0 (0/0)	0.0	90.2	2
1892	RF00208 snoR72	0.0 (0/9)	0.0	0.0 (0/0)	0.0	97.2	5
1893	RF01904 HOTAIR 1	0.0 (0/0)	0.0	0.0 (0/0)	0.0	91.8	9
1894	RF00367 mir-BHRF1-3	0.0 (0/22)	0.0	0.0 (0/0)	0.0	100.0	4
1895	RF02852 Ysr206	0.0 (0/87)	0.0	0.0 (0/0)	0.2	72.7	2
1896	RF01276 sR53	0.0 (0/0)	0.0	0.0 (0/0)	0.0	90.3	3
1897	RF02497 Atu C10	0.0 (0/25)	0.0	0.0 (0/0)	0.0	98.9	2
1898	RF00798 mir-49	0.0 (0/34)	0.0	0.0 (0/0)	0.7	76.2	4
1899	RF02077 STnc220	0.0 (0/11)	0.0	0.0 (0/0)	0.3	90.9	4
1900	RF01779 AS1726	0.0 (0/23)	0.0	0.0 (0/0)	0.0	100.0	2
1901	RF01169 SNORD112	0.0 (0/6)	0.0	0.0 (0/0)	2.0	81.0	13
1902	RF02754 ES205	0.0 (0/15)	0.0	0.0 (0/0)	0.1	95.7	5
1903	RF02658 IRES RhPV	0.0 (0/179)	0.0	0.0 (0/0)	0.0	98.3	2
1904	RF01926 mir-981	0.0 (0/26)	0.0	0.0 (0/0)	1.0	57.7	3
1905	RF02139 HOXA11-AS1 3	0.0 (0/0)	0.0	0.0 (0/0)	0.0	83.7	24
1906	RF02267 Vax2os1 2	0.0 (0/0)	0.0	0.0 (0/0)	0.0	92.9	3
1907	RF02324 TtnuHACA17	0.0 (0/27)	0.0	0.0 (0/0)	0.4	86.2	3
1908	RF02871 ncS54	0.0 (0/22)	0.0	0.0 (0/0)	0.0	99.2	4
1909	RF01954 SOX2OT exon4	0.0 (0/0)	0.0	0.0 (0/0)	0.0	81.4	10
1910	RF01526 ceN106	0.0 (0/2)	0.0	0.0 (0/0)	0.0	93.5	3
1911	RF02013 mir-1280	0.0 (0/33)	0.0	0.0 (0/0)	0.0	100.0	3
1912	RF02393 sau-59	0.0 (0/27)	0.0	0.0 (0/0)	0.6	87.2	3
1913	RF01185 snR75	0.0 (0/0)	0.0	0.0 (0/0)	0.0	68.9	61
1914	RF00484 IRES Cx32	0.0 (0/32)	0.0	0.0 (0/0)	1.0	93.7	6
1915	RF01137 sR21	0.0 (0/0)	0.0	0.0 (0/0)	0.0	53.0	2
1916	RF02709 TeloSII ncR43	0.0 (0/70)	0.0	0.0 (0/0)	0.6	91.7	4
1917	RF01892 TUG1 4	0.0 (0/0)	0.0	0.0 (0/0)	0.0	87.1	25
1918	RF01038 mir-1307	0.0 (0/32)	0.0	0.0 (0/0)	0.2	96.4	7
1919	RF00934 mir-463	0.0 (0/23)	0.0	0.0 (0/0)	0.2	87.7	2
1920	RF01189 sn1502	0.0 (0/5)	0.0	0.0 (0/0)	1.0	94.4	4
1921	RF02114 FAM13A-AS1 1	0.0 (0/0)	0.0	0.0 (0/1)	0.0	67.3	10

Continued on next page

	RNA family (seed alignment)	Sensitivity annotated bpairs that covary % (cov_bps/bps)	Power average power %	Positive Predictive Value covarying pairs in structure % (cov_bps/cov_pairs)	average substitutions per bpair	avg pairwise identity %	number of sequences
1922	RF02689 hilD 3p UTR	0.0 (0/86)	0.0	0.0 (0/0)	0.8	88.1	5
1923	RF02214 mir-56	0.0 (0/0)	0.0	0.0 (0/0)	0.0	77.3	4
1924	RF01469 rli33	0.0 (0/143)	0.0	0.0 (0/0)	0.1	94.8	3
1925	RF01507 Afu 298	0.0 (0/0)	0.0	0.0 (0/0)	0.0	79.6	3
1926	RF01197 snR39	0.0 (0/2)	0.0	0.0 (0/0)	1.0	86.0	4
1927	RF01578 RUF1	0.0 (0/67)	0.0	0.0 (0/0)	0.1	98.6	3
1928	RF02120 FTX 2	0.0 (0/0)	0.0	0.0 (0/0)	0.0	74.3	18
1929	RF00509 snosnR64	0.0 (0/0)	0.0	0.0 (0/1)	0.0	81.4	10
1930	RF02398 sau-6072	0.0 (0/17)	0.0	0.0 (0/0)	0.0	92.5	2
1931	RF02224 sX5	0.0 (0/10)	0.0	0.0 (0/0)	0.9	94.8	10
1932	RF01945 mir-1388	0.0 (0/28)	0.0	0.0 (0/0)	1.2	70.5	4
1933	RF02020 mir-25	0.0 (0/27)	0.0	0.0 (0/0)	0.2	95.2	3
1934	RF02747 FtrA	0.0 (0/22)	0.0	0.0 (0/0)	0.2	97.3	5
1935	RF02133 HOXB13-AS1 2	0.0 (0/0)	0.0	0.0 (0/0)	0.0	71.9	14
1936	RF00905 mir-789	0.0 (0/36)	0.0	0.0 (0/0)	0.1	91.8	2
1937	RF02847 Ref64	0.0 (0/23)	0.0	0.0 (0/0)	0.8	85.5	3
1938	RF02560 CbSR9	0.0 (0/48)	0.0	0.0 (0/0)	0.0	99.5	2
1939	RF02192 TCL6 2	0.0 (0/0)	0.0	0.0 (0/0)	0.0	69.8	12
1940	RF02826 Scr6925	0.0 (0/43)	0.0	0.0 (0/0)	0.9	82.6	4
1941	RF02513 PYLIS 6	0.0 (0/11)	0.0	0.0 (0/0)	0.0	100.0	3
1942	RF00323 snoR79	0.0 (0/5)	0.0	0.0 (0/0)	0.8	99.4	5
1943	RF00973 mir-597	0.0 (0/32)	0.0	0.0 (0/0)	0.9	81.2	4
1944	RF00986 mir-920	0.0 (0/23)	0.0	0.0 (0/0)	1.2	77.1	4
1945	RF02140 HOXA11-AS1 4	0.0 (0/0)	0.0	0.0 (0/0)	0.0	86.7	20
1946	RF01622 ceN22	0.0 (0/4)	0.0	0.0 (0/0)	0.2	94.7	2
1947	RF00963 mir-642	0.0 (0/36)	0.0	0.0 (0/0)	0.0	91.8	2
1948	RF00193 CTV rep sig	0.0 (0/73)	0.0	0.0 (0/0)	0.4	98.1	9
1949	RF02426 SpF03 sRNA	0.0 (0/31)	0.0	0.0 (0/0)	0.4	93.2	3
1950	RF02625 WsnRNA46	0.0 (0/36)	0.0	0.0 (0/0)	0.4	73.8	3
1951	RF01883 TUG1 2	0.0 (0/0)	0.0	0.0 (0/0)	0.0	88.8	17
1952	RF02197 TP73-AS1	0.0 (0/0)	0.0	0.0 (0/0)	0.0	71.7	4
1953	RF00977 mir-600	0.0 (0/25)	0.0	0.0 (0/0)	0.9	83.5	3
1954	RF02789 snoTBR12	0.0 (0/32)	0.0	0.0 (0/0)	0.9	73.4	3
1955	RF01574 DdR6	0.0 (0/0)	0.0	0.0 (0/0)	0.0	91.4	3
1956	RF01516 v-snoRNA-1	0.0 (0/0)	0.0	0.0 (0/0)	0.0	83.1	2
1957	RF01324 CRISPR-DR11	0.0 (0/7)	0.0	0.0 (0/0)	1.4	91.1	5
1958	RF02288 TtnuCD11	0.0 (0/0)	0.0	0.0 (0/0)	0.0	76.1	3
1959	RF00894 mir-790	0.0 (0/23)	0.0	0.0 (0/0)	0.1	85.1	2
1960	RF02590 Spy1186876	0.0 (0/59)	0.0	0.0 (0/0)	0.2	95.3	3
1961	RF02864 sRNA 0030	0.0 (0/58)	0.0	0.0 (0/0)	0.3	97.6	3
1962	RF01388 isrD	0.0 (0/13)	0.0	0.0 (0/0)	0.0	98.0	2
1963	RF01022 mir-611	0.0 (0/17)	0.0	0.0 (0/0)	2.0	75.1	8
1964	RF02119 FTX 1	0.0 (0/0)	0.0	0.0 (0/0)	0.0	79.3	11
1965	RF00707 mir-197	0.0 (0/31)	0.0	0.0 (0/1)	1.0	85.0	5
1966	RF02303 TtnuCD28	0.0 (0/0)	0.0	0.0 (0/0)	0.0	95.2	2
1967	RF00867 mir-BART5	0.0 (0/29)	0.0	0.0 (0/0)	0.1	78.7	2
1968	RF01575 DdR7	0.0 (0/0)	0.0	0.0 (0/0)	0.0	85.0	3
1969	RF01603 snoR29	0.0 (0/4)	0.0	0.0 (0/0)	0.0	84.7	3
1970	RF01510 MFR	0.0 (0/20)	0.0	0.0 (0/0)	0.8	74.1	3
1971	RF01429 snoR130	0.0 (0/3)	0.0	0.0 (0/0)	0.0	85.7	2
1972	RF02191 TCL6 1	0.0 (0/0)	0.0	0.0 (0/0)	0.0	77.8	9
1973	RF01027 mir-765	0.0 (0/37)	0.0	0.0 (0/0)	0.1	92.1	2
1974	RF01250 snR189	0.0 (0/44)	0.0	0.0 (0/1)	0.3	88.7	4
1975	RF02812 asponA	0.0 (0/88)	0.0	0.0 (0/0)	0.6	82.2	3
1976	RF01647 ceN61	0.0 (0/3)	0.0	0.0 (0/0)	0.0	93.1	4
1977	RF01860 Afu 455	0.0 (0/0)	0.0	0.0 (0/0)	0.0	78.1	6
1978	RF01082 SBWMV1 UPD-PKh	0.0 (0/10)	0.0	0.0 (0/0)	0.2	80.0	2
1979	RF01563 DdR13	0.0 (0/0)	0.0	0.0 (0/0)	0.0	81.5	3
1980	RF01309 sR60	0.0 (0/2)	0.0	0.0 (0/0)	0.0	87.6	4
1981	RF01913 mir-2778	0.0 (0/29)	0.0	0.0 (0/0)	0.4	93.8	7
1982	RF00290 BaMV CRE	0.0 (0/30)	0.0	0.0 (0/0)	0.1	99.1	5
1983	RF01505 Afu 264	0.0 (0/0)	0.0	0.0 (0/0)	0.0	69.2	5
1984	RF01346 CRISPR-DR36	0.0 (0/6)	0.0	0.0 (0/0)	0.2	94.4	3
1985	RF01085 TLS-PK4	0.0 (0/38)	0.0	0.0 (0/0)	0.1	89.7	2
1986	RF02201 TTC28-AS1 4	0.0 (0/0)	0.0	0.0 (0/0)	0.0	71.9	18
1987	RF01232 snoR442	0.0 (0/8)	0.0	0.0 (0/0)	1.6	88.2	10
1988	RF02441 SpF61 sRNA	0.0 (0/12)	0.0	0.0 (0/0)	0.2	88.5	3
1989	RF02389 sau-31	0.0 (0/11)	0.0	0.0 (0/0)	0.2	97.0	3
1990	RF02097 mir-1662	0.0 (0/22)	0.0	0.0 (0/0)	0.0	86.4	2
1991	RF01009 mir-M7	0.0 (0/23)	0.0	0.0 (0/0)	0.3	74.0	2
1992	RF02674 AsdA	0.0 (0/23)	0.0	0.0 (0/0)	0.5	94.0	4
1993	RF02395 sau-66	0.0 (0/20)	0.0	0.0 (0/0)	0.1	97.5	3
1994	RF01026 MIR828	0.0 (0/19)	0.0	0.0 (0/0)	0.8	70.8	4
1995	RF01205 snR62	0.0 (0/3)	0.0	0.0 (0/0)	0.0	95.0	2
1996	RF00904 mir-392	0.0 (0/26)	0.0	0.0 (0/0)	0.6	70.0	3
1997	RF01206 snoR109	0.0 (0/19)	0.0	0.0 (0/0)	1.3	77.6	6
1998	RF01837 toga FSE	0.0 (0/11)	0.0	0.0 (0/0)	0.1	95.4	3
1999	RF02472 Gl RNase MRP	0.0 (0/22)	0.0	0.0 (0/0)	0.1	90.8	2
2000	RF01646 ceN63	0.0 (0/3)	0.0	0.0 (0/0)	0.3	97.0	3
2001	RF00107 FinP	0.0 (0/24)	0.0	0.0 (0/0)	0.2	90.7	6
2002	RF01968 RMST 7	0.0 (0/0)	0.0	0.0 (0/0)	0.0	84.8	22
2003	RF02189 ST7-OT4 3	0.0 (0/0)	0.0	0.0 (0/0)	0.0	73.6	20
2004	RF02888 BtsR1	0.0 (0/9)	0.0	0.0 (0/0)	0.9	89.5	3
2005	RF01344 CRISPR-DR34	0.0 (0/8)	0.0	0.0 (0/0)	1.5	89.2	5

Continued on next page

	RNA family (seed alignment)	Sensitivity annotated bpairs that covary % (cov_bps/bps)	Power average power %	Positive Predictive Value covarying pairs in structure % (cov_bps/cov_pairs)	average substitutions per bpair	avg pairwise identity %	number of sequences
2006	RF02428 SpF11 sRNA	0.0 (0/20)	0.0	0.0 (0/0)	0.2	90.9	3
2007	RF02237 asX3	0.0 (0/50)	0.0	0.0 (0/0)	0.9	90.7	4
2008	RF01306 sR52	0.0 (0/4)	0.0	0.0 (0/0)	0.0	86.2	2
2009	RF02740 Fwd6 3p UTR	0.0 (0/28)	0.0	0.0 (0/0)	0.1	97.7	3
2010	RF01188 snR56	0.0 (0/0)	0.0	0.0 (0/0)	0.0	70.5	16
2011	RF00835 mir-58	0.0 (0/25)	0.0	0.0 (0/1)	0.0	87.6	4
2012	RF00907 mir-941	0.0 (0/33)	0.0	0.0 (0/0)	0.1	94.4	2
2013	RF00920 MIR444	0.0 (0/48)	0.0	0.0 (0/1)	0.9	74.8	4
2014	RF01644 ceN59	0.0 (0/26)	0.0	0.0 (0/2)	0.2	72.1	3
2015	RF02892 SR6 antitoxin	0.0 (0/30)	0.0	0.0 (0/0)	0.4	88.4	3
2016	RF01871 MALAT1	0.0 (0/0)	0.0	0.0 (0/0)	0.0	84.2	17
2017	RF00836 mir-250	0.0 (0/27)	0.0	0.0 (0/0)	0.4	82.4	4
2018	RF02101 HULC	0.0 (0/0)	0.0	0.0 (0/0)	0.0	66.9	19
2019	RF01921 mir-1296	0.0 (0/32)	0.0	0.0 (0/0)	0.1	96.7	6
2020	RF00702 mir-182	0.0 (0/20)	0.0	0.0 (0/0)	0.2	85.0	17
2021	RF01984 ZEB2 AS1 1	0.0 (0/0)	0.0	0.0 (0/0)	0.0	88.4	11
2022	RF01337 CRISPR-DR24	0.0 (0/5)	0.0	0.0 (0/0)	0.4	85.6	4
2023	RF01515 Afu 514	0.0 (0/0)	0.0	0.0 (0/0)	0.0	72.0	5
2024	RF01617 ceN113	0.0 (0/3)	0.0	0.0 (0/0)	0.0	81.9	2
2025	RF00918 mir-872	0.0 (0/25)	0.0	0.0 (0/0)	0.6	85.7	6
2026	RF02083 OrzO-P	0.0 (0/17)	0.0	0.0 (0/0)	1.0	85.5	7
2027	RF02650 CncR1	0.0 (0/62)	0.0	0.0 (0/0)	0.0	98.4	3
2028	RF01341 CRISPR-DR30	0.0 (0/6)	0.0	0.0 (0/0)	0.0	80.7	3
2029	RF01433 snoR137	0.0 (0/31)	0.0	0.0 (0/1)	0.5	76.7	4
2030	RF02023 mir-1208	0.0 (0/24)	0.0	0.0 (0/0)	0.0	99.1	3
2031	RF01437 S pombe snR10	0.0 (0/34)	0.0	0.0 (0/0)	0.0	100.0	2
2032	RF02570 BM-sr0117	0.0 (0/25)	0.0	0.0 (0/0)	0.1	98.4	3
2033	RF02486 GlsR23	0.0 (0/17)	0.0	0.0 (0/0)	0.1	83.0	2
2034	RF02734 Cgb105	0.0 (0/43)	0.0	0.0 (0/0)	0.0	97.0	2
2035	RF02090 DAOA-AS1 1	0.0 (0/0)	0.0	0.0 (0/0)	0.0	76.2	24
2036	RF02699 Avashort thermometer	0.0 (0/13)	0.0	0.0 (0/0)	0.0	91.4	2
2037	RF01553 TB9Cs1H3	0.0 (0/11)	0.0	0.0 (0/0)	0.7	73.8	3
2038	RF01906 HOTAIR 3	0.0 (0/0)	0.0	0.0 (0/0)	0.0	87.0	17
2039	RF01876 MIAT exon5 2	0.0 (0/0)	0.0	0.0 (0/0)	0.0	89.1	10
2040	RF02659 ncRv12659	0.0 (0/50)	0.0	0.0 (0/0)	0.0	99.4	2
2041	RF00734 mir-52	0.0 (0/34)	0.0	0.0 (0/0)	0.8	80.1	5
2042	RF01370 CRISPR-DR57	0.0 (0/7)	0.0	0.0 (0/0)	0.0	80.2	3
2043	RF02586 HCV package-SL733	0.0 (0/7)	0.0	0.0 (0/0)	0.0	94.9	4
2044	RF02730 JA02	0.0 (0/35)	0.0	0.0 (0/0)	0.4	89.9	4
2045	RF02841 Ref70	0.0 (0/29)	0.0	0.0 (0/0)	0.9	80.9	3
2046	RF02341 ncrMT1302	0.0 (0/20)	0.0	0.0 (0/0)	1.4	78.1	7
2047	RF01297 sR40	0.0 (0/12)	0.0	0.0 (0/0)	0.0	98.4	2
2048	RF00853 mir-304	0.0 (0/33)	0.0	0.0 (0/0)	0.4	91.7	5
2049	RF01874 MIAT exon1	0.0 (0/0)	0.0	0.0 (0/0)	0.0	87.5	10
2050	RF02295 TtnuCD19	0.0 (0/0)	0.0	0.0 (0/0)	0.0	83.8	2
2051	RF01360 CRISPR-DR47	0.0 (0/4)	0.0	0.0 (0/0)	0.0	97.3	2
2052	RF02195 TP53TG1 1	0.0 (0/0)	0.0	0.0 (0/0)	0.0	69.3	16
2053	RF02117 FMR1-AS1 1	0.0 (0/0)	0.0	0.0 (0/0)	0.0	86.1	20
2054	RF02641 Spy490483c	0.0 (0/27)	0.0	0.0 (0/0)	0.1	98.6	3
2055	RF01676 P31	0.0 (0/18)	0.0	0.0 (0/0)	1.1	87.2	4
2056	RF01604 plasmodium snoR28	0.0 (0/4)	0.0	0.0 (0/0)	0.0	85.5	3
2057	RF01550 TB8Cs3H1	0.0 (0/16)	0.0	0.0 (0/0)	1.8	78.3	7
2058	RF01151 snoU82P	0.0 (0/0)	0.0	0.0 (0/0)	0.0	86.3	14
2059	RF01898 mir-363	0.0 (0/26)	0.0	0.0 (0/0)	0.4	95.9	7
2060	RF01955 NEAT1 1	0.0 (0/0)	0.0	0.0 (0/0)	0.0	83.7	17
2061	RF01605 plasmodium snoR30	0.0 (0/3)	0.0	0.0 (0/0)	0.0	86.5	3
2062	RF01152 sR1	0.0 (0/0)	0.0	0.0 (0/1)	0.0	64.5	12
2063	RF02135 HTT-AS1 2	0.0 (0/0)	0.0	0.0 (0/0)	0.0	90.4	3
2064	RF00364 mir-BART2	0.0 (0/21)	0.0	0.0 (0/0)	1.4	92.9	8
2065	RF01131 sR47	0.0 (0/0)	0.0	0.0 (0/0)	0.0	72.1	8
2066	RF02163 sR-tMet	0.0 (0/17)	0.0	0.0 (0/0)	0.1	81.4	2
2067	RF00483 IRES IGF2	0.0 (0/28)	0.0	0.0 (0/0)	0.6	94.0	9
2068	RF01138 sR23	0.0 (0/0)	0.0	0.0 (0/0)	0.0	78.9	6
2069	RF02770 Ysr224	0.0 (0/28)	0.0	0.0 (0/0)	0.7	84.3	5
2070	RF02296 TtnuCD20	0.0 (0/0)	0.0	0.0 (0/0)	0.0	97.0	2
2071	RF01520 CC0734	0.0 (0/17)	0.0	0.0 (0/0)	0.1	83.2	2
2072	RF02250 Six3os1 5	0.0 (0/0)	0.0	0.0 (0/0)	0.0	83.7	15
2073	RF01130 sR46	0.0 (0/5)	0.0	0.0 (0/0)	1.0	83.4	7
2074	RF02671 Ysr35	0.0 (0/95)	0.0	0.0 (0/0)	0.0	99.2	3
2075	RF01407 STnc560	0.0 (0/39)	0.0	0.0 (0/0)	1.1	95.4	12
2076	RF00921 mir-665	0.0 (0/27)	0.0	0.0 (0/0)	0.7	86.8	6
2077	RF02507 Atu Ti3	0.0 (0/7)	0.0	0.0 (0/0)	1.0	78.0	4
2078	RF02024 mir-1180	0.0 (0/27)	0.0	0.0 (0/0)	0.4	89.4	3
2079	RF02335 GlsR2 miR4	0.0 (0/32)	0.0	0.0 (0/0)	0.1	99.3	3
2080	RF01353 CRISPR-DR44	0.0 (0/4)	0.0	0.0 (0/0)	0.0	83.3	3
2081	RF02867 ncS011	0.0 (0/62)	0.0	0.0 (0/0)	0.4	89.0	4
2082	RF00715 mir-383	0.0 (0/25)	0.0	0.0 (0/0)	0.5	91.1	6
2083	RF00372 sroH	0.0 (0/6)	0.0	0.0 (0/0)	0.0	76.5	2
2084	RF01641 ceN53	0.0 (0/4)	0.0	0.0 (0/0)	0.5	90.4	3
2085	RF01623 ceN23-1	0.0 (0/25)	0.0	0.0 (0/0)	0.0	93.7	3
2086	RF00887 mir-802	0.0 (0/33)	0.0	0.0 (0/0)	1.3	87.3	13
2087	RF00601 SCARNA20	0.0 (0/33)	0.0	0.0 (0/0)	1.0	79.0	5
2088	RF01905 HOTAIR 2	0.0 (0/0)	0.0	0.0 (0/0)	0.0	88.7	11
2089	RF01576 DdR8	0.0 (0/0)	0.0	0.0 (0/0)	0.0	100.0	2

Continued on next page

	RNA family (seed alignment)	Sensitivity annotated bpairs that covary % (cov_bps/bps)	Power average power %	Positive Predictive Value covarying pairs in structure % (cov_bps/cov_pairs)	average substitutions per bpair	avg pairwise identity %	number of sequences
2090	RF01985 ZEB2 AS1 2	0.0 (0/0)	0.0	0.0 (0/0)	0.0	88.2	11
2091	RF00979 mir-553	0.0 (0/19)	0.0	0.0 (0/0)	1.1	79.8	3
2092	RF02213 ZFAT-AS1 3	0.0 (0/0)	0.0	0.0 (0/0)	0.0	83.9	5
2093	RF01474 rli42	0.0 (0/58)	0.0	0.0 (0/0)	0.2	95.0	4
2094	RF01589 plasmodium snoR11	0.0 (0/53)	0.0	0.0 (0/0)	0.1	96.5	2
2095	RF00744 mir-361	0.0 (0/24)	0.0	0.0 (0/0)	0.2	93.3	5
2096	RF01078 PK-PYVV	0.0 (0/15)	0.0	0.0 (0/0)	0.3	84.1	3
2097	RF00806 mir-350	0.0 (0/29)	0.0	0.0 (0/0)	1.6	87.9	11
2098	RF01821 RsaH	0.0 (0/29)	0.0	0.0 (0/0)	1.8	70.9	5
2099	RF02386 sau-19	0.0 (0/27)	0.0	0.0 (0/0)	0.2	92.4	4
2100	RF01861 TB9Cs1H1	0.0 (0/11)	0.0	0.0 (0/0)	1.0	70.2	4
2101	RF02207 WT1-AS 5	0.0 (0/0)	0.0	0.0 (0/0)	0.0	98.2	4
2102	RF02602 BASRCI414	0.0 (0/74)	0.0	0.0 (0/0)	0.2	96.5	3
2103	RF00781 mir-505	0.0 (0/32)	0.0	0.0 (0/0)	0.3	93.5	5
2104	RF02274 AniS	0.0 (0/22)	0.0	0.0 (0/0)	0.3	95.5	5
2105	RF01032 mir-554	0.0 (0/18)	0.0	0.0 (0/0)	1.1	79.4	4
2106	RF02801 PyrR201	0.0 (0/21)	0.0	0.0 (0/0)	0.5	86.5	5
2107	RF02677 NsiR4	0.0 (0/12)	0.0	0.0 (0/0)	0.8	86.6	4
2108	RF01887 DLX6-AS1 1	0.0 (0/0)	0.0	0.0 (0/0)	0.0	90.8	15
2109	RF02647 Cis52 sRNA	0.0 (0/57)	0.0	0.0 (0/0)	0.1	88.3	2
2110	RF02339 GlsR17 miR2	0.0 (0/21)	0.0	0.0 (0/0)	0.6	93.4	4
2111	RF02326 TtnuHACA19	0.0 (0/27)	0.0	0.0 (0/0)	0.0	95.9	2
2112	RF00179 GAIT	0.0 (0/18)	0.0	0.0 (0/0)	0.7	98.8	8
2113	RF02226 sX7	0.0 (0/26)	0.0	0.0 (0/0)	0.5	95.9	8
2114	RF02042 HOTTIP 3	0.0 (0/0)	0.0	0.0 (0/0)	0.0	80.0	23
2115	RF01677 P35	0.0 (0/23)	0.0	0.0 (0/0)	0.0	98.4	2
2116	RF02578 tsr25	0.0 (0/53)	0.0	0.0 (0/0)	0.0	99.6	3
2117	RF01650 ceN68	0.0 (0/29)	0.0	0.0 (0/0)	0.0	100.0	2
2118	RF01556 TB9Cs3H2	0.0 (0/14)	0.0	0.0 (0/0)	1.0	74.9	3
2119	RF00812 mir-83	0.0 (0/33)	0.0	0.0 (0/1)	0.5	85.2	4
2120	RF01108 BMV3 UPD-PK1	0.0 (0/10)	0.0	0.0 (0/0)	0.0	100.0	2
2121	RF01841 veev FSE	0.0 (0/9)	0.0	0.0 (0/1)	1.7	88.5	10
2122	RF02821 V IGR5	0.0 (0/29)	0.0	0.0 (0/0)	0.6	61.3	2
2123	RF02136 HTT-AS1 3	0.0 (0/0)	0.0	0.0 (0/0)	0.0	82.2	4
2124	RF02252 Six3os1 7	0.0 (0/0)	0.0	0.0 (0/2)	0.0	83.4	5
2125	RF02828 Scr3920	0.0 (0/39)	0.0	0.0 (0/0)	0.3	93.7	6
2126	RF01948 KCNQ1OT1 3	0.0 (0/0)	0.0	0.0 (0/0)	0.0	89.8	9
2127	RF00961 mir-581	0.0 (0/34)	0.0	0.0 (0/0)	0.1	94.8	2
2128	RF00439 SNORD87	0.0 (0/7)	0.0	0.0 (0/0)	4.3	90.7	10
2129	RF02128 GNAS-AS1 2	0.0 (0/0)	0.0	0.0 (0/0)	0.0	69.3	16
2130	RF00776 mir-540	0.0 (0/28)	0.0	0.0 (0/0)	0.1	85.5	2
2131	RF02173 SMAD5-AS1 1	0.0 (0/0)	0.0	0.0 (0/0)	0.0	71.5	18
2132	RF02049 STnc460	0.0 (0/47)	0.0	0.0 (0/0)	0.1	79.0	2
2133	RF00385 IBV D-RNA	0.0 (0/15)	0.0	0.0 (0/0)	0.2	93.9	10
2134	RF02468 Ms IGR-4	0.0 (0/10)	0.0	0.0 (0/0)	0.2	94.7	2
2135	RF02878 MH s7	0.0 (0/60)	0.0	0.0 (0/2)	0.9	77.0	3
2136	RF00399 SNORA24	0.0 (0/33)	0.0	0.0 (0/0)	0.7	88.8	5
2137	RF02703 AsxR	0.0 (0/9)	0.0	0.0 (0/0)	0.1	98.1	2
2138	RF01342 CRISPR-DR32	0.0 (0/4)	0.0	0.0 (0/0)	0.0	81.1	2
2139	RF00447 IRES Kv1 4	0.0 (0/51)	0.0	0.0 (0/0)	0.5	89.1	6
2140	RF02596 cyano het sRNA	0.0 (0/15)	0.0	0.0 (0/1)	0.4	90.9	5
2141	RF02315 TtnuHACA8	0.0 (0/38)	0.0	0.0 (0/0)	0.4	88.0	3
2142	RF02571 Mcr7	0.0 (0/124)	0.0	0.0 (0/0)	0.0	96.3	2
2143	RF02427 SpF10 sRNA	0.0 (0/17)	0.0	0.0 (0/0)	0.1	98.2	2
2144	RF02454 ncr982	0.0 (0/14)	0.0	0.0 (0/0)	1.3	79.7	6
2145	RF01127 sR42	0.0 (0/0)	0.0	0.0 (0/0)	0.0	83.1	3
2146	RF02775 SodB thermometer	0.0 (0/24)	0.0	0.0 (0/1)	1.3	85.6	5
2147	RF02727 sno ZL116	0.0 (0/20)	0.0	0.0 (0/0)	0.4	95.0	5
2148	RF02893 RpsF leader	0.0 (0/16)	0.0	0.0 (0/0)	0.2	89.8	10
2149	RF02282 TtnuCD5	0.0 (0/0)	0.0	0.0 (0/0)	0.0	97.4	2
2150	RF02317 TtnuHACA10	0.0 (0/23)	0.0	0.0 (0/0)	0.4	86.5	3
2151	RF01278 SNORD109A	0.0 (0/5)	0.0	0.0 (0/0)	0.6	87.2	7
2152	RF02538 TarB	0.0 (0/18)	0.0	0.0 (0/0)	0.0	94.9	2
2153	RF00985 mir-640	0.0 (0/29)	0.0	0.0 (0/0)	0.0	97.2	3
2154	RF02865 ncS01	0.0 (0/39)	0.0	0.0 (0/0)	0.5	89.2	4
2155	RF02837 Bcj7	0.0 (0/50)	0.0	0.0 (0/0)	0.5	88.7	3
2156	RF00752 mir-14	0.0 (0/22)	0.0	0.0 (0/0)	1.3	80.8	6
2157	RF00769 mir-331	0.0 (0/26)	0.0	0.0 (0/0)	0.1	93.8	5
2158	RF01963 RMST 2	0.0 (0/0)	0.0	0.0 (0/0)	0.0	82.8	16
2159	RF02488 GlsR25	0.0 (0/29)	0.0	0.0 (0/0)	0.7	85.8	3
2160	RF01681 P4	0.0 (0/70)	0.0	0.0 (0/0)	0.0	96.7	2
2161	RF01300 snoU49	0.0 (0/8)	0.0	0.0 (0/0)	0.8	80.4	5
2162	RF02662 BLi r0872	0.0 (0/48)	0.0	0.0 (0/0)	0.0	98.6	2
2163	RF01357 CRISPR-DR31	0.0 (0/10)	0.0	0.0 (0/0)	0.4	91.0	3
2164	RF02309 TtnuHACA1	0.0 (0/37)	0.0	0.0 (0/0)	0.0	96.5	2
2165	RF00217 SNORD20	0.0 (0/4)	0.0	0.0 (0/0)	0.8	88.3	13
2166	RF02251 Six3os1 6	0.0 (0/0)	0.0	0.0 (0/0)	0.0	83.6	4
2167	RF02403 Afu 328	0.0 (0/0)	0.0	0.0 (0/0)	0.0	82.1	4
2168	RF01095 PK-CuYV BPYV	0.0 (0/15)	0.0	0.0 (0/0)	0.0	100.0	2
2169	RF02708 TeloSII ncR40	0.0 (0/47)	0.0	0.0 (0/0)	0.1	93.8	2
2170	RF01973 H19 2	0.0 (0/0)	0.0	0.0 (0/0)	0.0	89.8	23
2171	RF02254 mir-35	0.0 (0/40)	0.0	0.0 (0/0)	0.1	76.0	2
2172	RF02537 TarA	0.0 (0/22)	0.0	0.0 (0/0)	0.0	96.9	2
2173	RF01528 CC3513	0.0 (0/53)	0.0	0.0 (0/0)	0.1	81.3	2

Continued on next page

	RNA family (seed alignment)	Sensitivity annotated bpairs that covary % (cov_bps/bps)	Power average power %	Positive Predictive Value covarying pairs in structure % (cov_bps/cov_pairs)	average substitutions per bpair	avg pairwise identity %	number of sequences
2174	RF00709 mir-455	0.0 (0/29)	0.0	0.0 (0/0)	0.3	93.0	5
2175	RF02026 mir-2833	0.0 (0/23)	0.0	0.0 (0/0)	0.5	94.6	5
2176	RF01967 RMST 6	0.0 (0/0)	0.0	0.0 (0/0)	0.0	84.5	18
2177	RF02220 ZNRD1-AS1 3	0.0 (0/0)	0.0	0.0 (0/0)	0.0	92.3	9
2178	RF01375 CRISPR-DR62	0.0 (0/4)	0.0	0.0 (0/0)	0.0	91.9	2
2179	RF02880 MH s15	0.0 (0/91)	0.0	0.0 (0/0)	0.2	70.2	2
2180	RF00306 snoZ178	0.0 (0/25)	0.0	0.0 (0/0)	0.0	99.7	5
2181	RF02165 PVT1 2	0.0 (0/0)	0.0	0.0 (0/0)	0.0	69.0	17
2182	RF02903 AaHKsRNA96	0.0 (0/38)	0.0	0.0 (0/0)	0.7	74.4	3
2183	RF01445 S pombe snR94	0.0 (0/50)	0.0	0.0 (0/0)	1.2	74.0	3
2184	RF02581 tsr32	0.0 (0/51)	0.0	0.0 (0/0)	0.5	95.7	4
2185	RF00770 mir-330	0.0 (0/32)	0.0	0.0 (0/0)	0.6	88.5	6
2186	RF02102 DISC2	0.0 (0/0)	0.0	0.0 (0/0)	0.0	86.8	5
2187	RF02851 Ysr283	0.0 (0/60)	0.0	0.0 (0/1)	0.7	79.7	3
2188	RF01864 plasmodium snoR21	0.0 (0/4)	0.0	0.0 (0/3)	0.0	83.5	4
2189	RF00857 mir-233	0.0 (0/31)	0.0	0.0 (0/0)	0.6	82.3	4
2190	RF02697 LDH1 5p UTR	0.0 (0/22)	0.0	0.0 (0/0)	0.1	97.4	4
2191	RF00935 mir-876	0.0 (0/32)	0.0	0.0 (0/0)	0.6	91.8	3
2192	RF01103 UPD-PKc	0.0 (0/11)	0.0	0.0 (0/0)	0.1	96.5	2
2193	RF01382 HIV-1 SL4	0.0 (0/5)	0.0	0.0 (0/0)	0.0	91.8	16
2194	RF01566 DdR16	0.0 (0/0)	0.0	0.0 (0/0)	0.0	85.4	4
2195	RF02778 FdoG1 thermometer	0.0 (0/48)	0.0	0.0 (0/0)	0.6	87.3	4
2196	RF02157 NPPA-AS1 2	0.0 (0/0)	0.0	0.0 (0/0)	0.0	77.0	16
2197	RF01366 CRISPR-DR53	0.0 (0/9)	0.0	0.0 (0/0)	0.0	97.3	2
2198	RF02849 Ysr197	0.0 (0/60)	0.0	0.0 (0/0)	1.1	80.0	3
2199	RF02643 Spy491738	0.0 (0/22)	0.0	0.0 (0/0)	0.3	90.9	4
2200	RF00845 MIR158	0.0 (0/33)	0.0	0.0 (0/0)	0.0	90.0	2
2201	RF01236 snoU19	0.0 (0/35)	0.0	0.0 (0/0)	1.1	67.7	3
2202	RF02561 CbSR12	0.0 (0/31)	0.0	0.0 (0/0)	0.0	99.3	2
2203	RF01318 CRISPR-DR5	0.0 (0/7)	0.0	0.0 (0/0)	0.0	78.0	12
2204	RF02006 mir-1253	0.0 (0/38)	0.0	0.0 (0/0)	0.1	95.2	2
2205	RF01567 DdR17	0.0 (0/0)	0.0	0.0 (0/0)	0.0	100.0	2
2206	RF02646 Cis8 sRNA	0.0 (0/40)	0.0	0.0 (0/0)	0.2	75.8	2
2207	RF00869 mir-BART7	0.0 (0/33)	0.0	0.0 (0/0)	0.1	84.5	2
2208	RF02721 sca ncR26	0.0 (0/18)	0.0	0.0 (0/0)	0.3	85.1	2
2209	RF01453 RCNMV TE DR1	0.0 (0/43)	0.0	0.0 (0/0)	0.1	88.7	3
2210	RF02580 tsr31	0.0 (0/18)	0.0	0.0 (0/0)	0.1	94.7	4
2211	RF01381 HIV-1 SL3	0.0 (0/5)	0.0	0.0 (0/0)	0.6	90.3	19
2212	RF01096 PK-HAV	0.0 (0/17)	0.0	0.0 (0/0)	0.0	87.3	2
2213	RF02287 TtnuCD10	0.0 (0/0)	0.0	0.0 (0/0)	0.0	79.5	4
2214	RF01124 sR36	0.0 (0/0)	0.0	0.0 (0/0)	0.0	84.2	5
2215	RF02890 SprC	0.0 (0/36)	0.0	0.0 (0/0)	0.3	64.3	2
2216	RF01986 ZEB2 AS1 3	0.0 (0/0)	0.0	0.0 (0/0)	0.0	90.2	11
2217	RF02833 Scr3202	0.0 (0/28)	0.0	0.0 (0/0)	0.8	82.5	4
2218	RF01450 S pombe snR96	0.0 (0/50)	0.0	0.0 (0/0)	0.0	99.5	2
2219	RF02781 ManX thermometer	0.0 (0/33)	0.0	0.0 (0/0)	0.7	82.3	4
2220	RF02621 BSnc120	0.0 (0/23)	0.0	0.0 (0/0)	0.2	98.5	3
2221	RF01542 TB11Cs5H1	0.0 (0/17)	0.0	0.0 (0/0)	0.1	74.3	2
2222	RF01184 snR79	0.0 (0/0)	0.0	0.0 (0/0)	0.0	75.5	18
2223	RF00067 SNORD15	0.0 (0/4)	0.0	0.0 (0/0)	0.2	60.9	11
2224	RF01873 PISRT1	0.0 (0/0)	0.0	0.0 (0/0)	0.0	88.1	18
2225	RF02476 GlsR7	0.0 (0/3)	0.0	0.0 (0/0)	0.0	88.1	3
2226	RF02715 sno ncR1	0.0 (0/32)	0.0	0.0 (0/0)	0.8	87.1	5
2227	RF02527 SSRC38	0.0 (0/44)	0.0	0.0 (0/0)	0.0	97.6	3
2228	RF02705 B rapa snoR775	0.0 (0/29)	0.0	0.0 (0/0)	0.0	96.1	2
2229	RF00925 MIR1027	0.0 (0/43)	0.0	0.0 (0/0)	0.0	100.0	2
2230	RF02007 mir-1237	0.0 (0/38)	0.0	0.0 (0/0)	0.1	96.1	3
2231	RF02112 DLG2-AS1 1	0.0 (0/0)	0.0	0.0 (0/0)	0.0	80.9	21
2232	RF00266 snoZ17	0.0 (0/6)	0.0	0.0 (0/0)	0.8	76.9	26
2233	RF00941 mir-434	0.0 (0/31)	0.0	0.0 (0/0)	0.1	89.4	2
2234	RF01962 RMST 1	0.0 (0/0)	0.0	0.0 (0/0)	0.0	83.8	14
2235	RF02622 BSnc121	0.0 (0/31)	0.0	0.0 (0/0)	0.0	99.1	2
2236	RF01075 TLS-PK1	0.0 (0/31)	0.0	0.0 (0/0)	0.0	87.5	2
2237	RF01177 snR67	0.0 (0/0)	0.0	0.0 (0/0)	0.0	73.7	16
2238	RF00305 snoZ248	0.0 (0/26)	0.0	0.0 (0/0)	0.0	98.1	5
2239	RF01915 mir-2238	0.0 (0/31)	0.0	0.0 (0/0)	1.2	79.5	4
2240	RF02055 STnc380	0.0 (0/28)	0.0	0.0 (0/0)	0.9	79.8	5
2241	RF01365 CRISPR-DR52	0.0 (0/7)	0.0	0.0 (0/0)	0.0	100.0	2
2242	RF02075 STnc230	0.0 (0/12)	0.0	0.0 (0/0)	1.1	67.4	11
2243	RF01126 sR41	0.0 (0/0)	0.0	0.0 (0/0)	0.0	73.3	7
2244	RF01461 rli24	0.0 (0/35)	0.0	0.0 (0/0)	0.0	94.3	6
2245	RF01618 ceN114	0.0 (0/3)	0.0	0.0 (0/0)	0.0	97.8	3
2246	RF02301 TtnuCD25	0.0 (0/0)	0.0	0.0 (0/0)	0.0	83.3	3
2247	RF01503 Afu 203	0.0 (0/35)	0.0	0.0 (0/0)	0.2	92.1	3
2248	RF01235 snR68	0.0 (0/38)	0.0	0.0 (0/0)	0.2	95.2	5
2249	RF01352 CRISPR-DR43	0.0 (0/0)	0.0	0.0 (0/0)	0.0	86.2	3
2250	RF01021 mir-558	0.0 (0/34)	0.0	0.0 (0/0)	0.4	95.7	3
2251	RF02626 WsnRNA59	0.0 (0/23)	0.0	0.0 (0/0)	0.3	76.1	2
2252	RF01654 ceN82	0.0 (0/30)	0.0	0.0 (0/0)	0.7	87.4	6
2253	RF01584 snoR03	0.0 (0/38)	0.0	0.0 (0/0)	0.2	75.7	2
2254	RF01149 sR10	0.0 (0/0)	0.0	0.0 (0/0)	0.0	85.5	4
2255	RF01586 snoR09	0.0 (0/3)	0.0	0.0 (0/0)	0.0	93.7	3
2256	RF02653 StyR-143	0.0 (0/44)	0.0	0.0 (0/0)	0.0	96.5	2
2257	RF02461 Virus CITE 6	0.0 (0/34)	0.0	0.0 (0/0)	1.3	85.1	3

Continued on next page

	RNA family (seed alignment)	Sensitivity annotated bpairs that covary % (cov_bps/bps)	Power average power %	Positive Predictive Value covarying pairs in structure % (cov_bps/cov_pairs)	average substitutions per bpair	avg pairwise identity %	number of sequences
2258	RF00326 snoZ155	0.0 (0/6)	0.0	0.0 (0/0)	4.2	78.8	8
2259	RF01495 ACAT	0.0 (0/32)	0.0	0.0 (0/0)	1.5	80.8	8
2260	RF02205 WT1-AS 3	0.0 (0/0)	0.0	0.0 (0/0)	0.0	71.5	19
2261	RF01839 eeev FSE	0.0 (0/10)	0.0	0.0 (0/0)	0.5	93.2	6
2262	RF02155 NCRUPAR 2	0.0 (0/0)	0.0	0.0 (0/0)	0.0	92.5	3
2263	RF02845 Ref1C	0.0 (0/34)	0.0	0.0 (0/0)	0.6	59.5	2
2264	RF02825 LPR17	0.0 (0/69)	0.0	0.0 (0/0)	0.2	87.7	2
2265	RF02616 SSRC8 2	0.0 (0/87)	0.0	0.0 (0/0)	0.0	97.2	2
2266	RF02460 Virus CITE 5	0.0 (0/32)	0.0	0.0 (0/0)	0.1	83.7	2
2267	RF01572 DdR4	0.0 (0/0)	0.0	0.0 (0/0)	0.0	86.2	3
2268	RF00898 mir-242	0.0 (0/23)	0.0	0.0 (0/0)	0.0	85.7	2
2269	RF02137 HOXA11-AS1 1	0.0 (0/0)	0.0	0.0 (0/0)	0.0	88.5	15
2270	RF01509 Afu 300	0.0 (0/0)	0.0	0.0 (0/0)	0.0	73.3	10
2271	RF02039 SPRY4-IT1 2	0.0 (0/0)	0.0	0.0 (0/0)	0.0	70.0	24
2272	RF01204 snR65	0.0 (0/0)	0.0	0.0 (0/0)	0.0	92.7	5
2273	RF02193 TCL6 3	0.0 (0/0)	0.0	0.0 (0/0)	0.0	76.6	26
2274	RF00331 snoZ169	0.0 (0/6)	0.0	0.0 (0/0)	1.5	87.4	3
2275	RF01338 CRISPR-DR25	0.0 (0/6)	0.0	0.0 (0/0)	0.7	92.8	5
2276	RF00816 mir-245	0.0 (0/18)	0.0	0.0 (0/0)	0.2	87.3	3
2277	RF02767 Ysr186 sR026 CsrC	0.0 (0/87)	0.0	0.0 (0/0)	0.7	87.3	4
2278	RF02132 HOXB13-AS1 1	0.0 (0/0)	0.0	0.0 (0/0)	0.0	73.4	12
2279	RF01833 astro FSE	0.0 (0/12)	0.0	0.0 (0/0)	0.3	90.3	4
2280	RF00861 mir-488	0.0 (0/30)	0.0	0.0 (0/0)	0.3	92.9	5
2281	RF02209 WT1-AS 7	0.0 (0/0)	0.0	0.0 (0/0)	0.0	84.8	21
2282	RF00087 SNORD26	0.0 (0/4)	0.0	0.0 (0/1)	2.2	79.2	17
2283	RF02126 GHRLOS	0.0 (0/0)	0.0	0.0 (0/0)	0.0	85.5	16
2284	RF02131 GNAS-AS1 5	0.0 (0/0)	0.0	0.0 (0/0)	0.0	81.8	22
2285	RF01428 snoR128	0.0 (0/3)	0.0	0.0 (0/0)	0.0	74.1	5
2286	RF01590 plasmodium snoR14	0.0 (0/3)	0.0	0.0 (0/0)	0.0	86.4	4
2287	RF01534 TB10Cs4H4	0.0 (0/18)	0.0	0.0 (0/0)	0.2	77.8	2
2288	RF02696 Teg49	0.0 (0/49)	0.0	0.0 (0/0)	0.5	93.8	6
2289	RF01100 PK-BYV	0.0 (0/11)	0.0	0.0 (0/0)	0.0	100.0	2
2290	RF00974 mir-607	0.0 (0/41)	0.0	0.0 (0/0)	0.3	94.4	3
2291	RF02217 ZNFX1-AS1 3	0.0 (0/0)	0.0	0.0 (0/0)	0.0	92.9	3
2292	RF02854 Ysr100	0.0 (0/30)	0.0	0.0 (0/0)	0.6	86.4	3
2293	RF01244 snR4	0.0 (0/54)	0.0	0.0 (0/1)	1.5	81.1	5
2294	RF00978 mir-638	0.0 (0/22)	0.0	0.0 (0/0)	0.4	90.7	6
2295	RF00294 snoTBR17	0.0 (0/2)	0.0	0.0 (0/0)	2.5	76.8	6
2296	RF01964 RMST 3	0.0 (0/0)	0.0	0.0 (0/0)	0.0	89.1	16
2297	RF00900 mir-255	0.0 (0/25)	0.0	0.0 (0/0)	0.0	69.9	2
2298	RF02327 TtnuHACA20	0.0 (0/38)	0.0	0.0 (0/0)	0.0	94.3	2
2299	RF01363 CRISPR-DR50	0.0 (0/13)	0.0	0.0 (0/0)	0.2	82.6	2
2300	RF00496 Corona SL-III	0.0 (0/8)	0.0	0.0 (0/0)	0.5	93.3	5
2301	RF01141 sR18	0.0 (0/0)	0.0	0.0 (0/0)	0.0	71.5	5
2302	RF01350 CRISPR-DR41	0.0 (0/8)	0.0	0.0 (0/0)	0.0	93.1	2
2303	RF01223 snR13	0.0 (0/2)	0.0	0.0 (0/0)	0.5	95.1	2
2304	RF01053 Deinococcus Y RNA	0.0 (0/35)	0.0	0.0 (0/0)	0.0	100.0	2
2305	RF01953 SOX2OT exon3	0.0 (0/0)	0.0	0.0 (0/1)	0.0	83.2	11
2306	RF02793 asR3	0.0 (0/17)	0.0	0.0 (0/0)	0.2	79.8	3
2307	RF02014 mir-1178	0.0 (0/28)	0.0	0.0 (0/0)	0.0	94.0	3
2308	RF02686 RAGATH-6	0.0 (0/35)	0.0	0.0 (0/0)	0.0	87.3	2
2309	RF01829 sR6	0.0 (0/8)	0.0	0.0 (0/0)	0.0	100.0	2
2310	RF02208 WT1-AS 6	0.0 (0/0)	0.0	0.0 (0/0)	0.0	78.9	19
2311	RF02493 Gl U4	0.0 (0/13)	0.0	0.0 (0/0)	0.0	92.6	2
2312	RF02183 ST7-OT3 1	0.0 (0/0)	0.0	0.0 (0/0)	0.0	79.3	28
2313	RF02385 sau-13	0.0 (0/25)	0.0	0.0 (0/1)	1.8	79.5	6
2314	RF01142 sR19	0.0 (0/0)	0.0	0.0 (0/0)	0.0	91.5	3
2315	RF00118 rydB	0.0 (0/12)	0.0	0.0 (0/0)	0.2	78.9	7
2316	RF02533 HAV CRE	0.0 (0/35)	0.0	0.0 (0/0)	0.1	91.2	2
2317	RF02601 BASRCI337	0.0 (0/57)	0.0	0.0 (0/0)	0.1	98.4	4
2318	RF02367 Yfr20	0.0 (0/25)	0.0	0.0 (0/0)	1.2	79.5	7
2319	RF02794 Pab19	0.0 (0/31)	0.0	0.0 (0/0)	0.9	80.9	3
2320	RF02835 Bcj11	0.0 (0/39)	0.0	0.0 (0/0)	0.9	80.7	4
2321	RF02814 Sso133	0.0 (0/19)	0.0	0.0 (0/0)	0.6	80.4	3
2322	RF02088 STnc510	0.0 (0/230)	0.0	0.0 (0/0)	0.6	87.7	5
2323	RF01683 P6	0.0 (0/151)	0.0	0.0 (0/0)	0.1	95.4	3
2324	RF01305 sR51	0.0 (0/12)	0.0	0.0 (0/0)	1.3	80.4	4
2325	RF02164 PVT1 1	0.0 (0/0)	0.0	0.0 (0/0)	0.0	74.0	17
2326	RF01933 bxd 5	0.0 (0/0)	0.0	0.0 (0/0)	0.0	96.1	5
2327	RF02065 STnc340	0.0 (0/15)	0.0	0.0 (0/0)	0.4	78.2	4
2328	RF02178 SMCR2 2	0.0 (0/0)	0.0	0.0 (0/0)	0.0	69.7	5
2329	RF01934 bxd 6	0.0 (0/0)	0.0	0.0 (0/0)	0.0	87.3	6
2330	RF00676 mir-127	0.0 (0/31)	0.0	0.0 (0/0)	0.0	98.8	5
2331	RF00829 mir-149	0.0 (0/34)	0.0	0.0 (0/0)	0.3	91.0	3
2332	RF02008 mir-621	0.0 (0/33)	0.0	0.0 (0/0)	0.2	95.8	3
2333	RF01672 P2	0.0 (0/47)	0.0	0.0 (0/0)	0.0	99.2	7
2334	RF01094 RF site6	0.0 (0/16)	0.0	0.0 (0/0)	0.0	87.3	2
2335	RF02611 BSR0653	0.0 (0/181)	0.0	0.0 (0/0)	0.0	99.6	4
2336	RF00119 C0299	0.0 (0/20)	0.0	0.0 (0/0)	0.2	96.7	5
2337	RF02804 PyrR206	0.0 (0/14)	0.0	0.0 (0/0)	0.6	88.7	4
2338	RF00207 K10 TLS	0.0 (0/17)	0.0	0.0 (0/0)	0.0	100.0	5
2339	RF01932 bxd 4	0.0 (0/0)	0.0	0.0 (0/0)	0.0	78.0	5
2340	RF01615 ceN111	0.0 (0/3)	0.0	0.0 (0/0)	0.0	87.5	3
2341	RF02733 ToxT thermometer	0.0 (0/20)	0.0	0.0 (0/0)	0.2	94.4	3

Continued on next page

	RNA family (seed alignment)	Sensitivity annotated bpairs that covary % (cov_bps/bps)	Power average power %	Positive Predictive Value covarying pairs in structure % (cov_bps/cov_pairs)	average substitutions per bpair	avg pairwise identity %	number of sequences
2342	RF00433 Hsp90 CRE	0.0 (0/49)	0.0	0.0 (0/0)	0.5	95.6	6
2343	RF02652 StyR-3	0.0 (0/40)	0.0	0.0 (0/0)	0.0	98.8	5
2344	RF02635 EF0820 EF0821	0.0 (0/117)	0.0	0.0 (0/0)	0.0	99.7	2
2345	RF00790 mir-358	0.0 (0/30)	0.0	0.0 (0/1)	0.7	68.3	3
2346	RF00868 mir-BART15	0.0 (0/27)	0.0	0.0 (0/0)	0.1	79.5	2
2347	RF01190 snR50	0.0 (0/2)	0.0	0.0 (0/0)	0.0	91.3	3
2348	RF01466 rli34	0.0 (0/10)	0.0	0.0 (0/0)	0.4	87.0	5
2349	RF02831 Scr2736	0.0 (0/18)	0.0	0.0 (0/0)	0.0	96.8	4
2350	RF02307 TtnuCD33	0.0 (0/0)	0.0	0.0 (0/0)	0.0	93.2	2
2351	RF02107 DLEU2 3	0.0 (0/0)	0.0	0.0 (0/0)	0.0	82.4	24
2352	RF01040 mir-573	0.0 (0/29)	0.0	0.0 (0/0)	0.1	90.9	2
2353	RF02298 TtnuCD22	0.0 (0/0)	0.0	0.0 (0/0)	0.0	97.4	2
2354	RF02091 DAOA-AS1 2	0.0 (0/0)	0.0	0.0 (0/0)	0.0	70.8	24
2355	RF02182 ST7-AS2 2	0.0 (0/0)	0.0	0.0 (0/0)	0.0	81.7	8
2356	RF01817 RsaB	0.0 (0/15)	0.0	0.0 (0/0)	0.1	98.2	2
2357	RF02648 Cis90 sRNA	0.0 (0/73)	0.0	0.0 (0/0)	0.4	65.7	2
2358	RF02744 Rev39 5p UTR	0.0 (0/93)	0.0	0.0 (0/0)	0.3	92.7	4
2359	RF02249 Six3os1 4	0.0 (0/0)	0.0	0.0 (0/0)	0.0	81.8	7
2360	RF01101 TLS-PK6	0.0 (0/8)	0.0	0.0 (0/0)	0.0	88.3	3
2361	RF01625 ceN28	0.0 (0/4)	0.0	0.0 (0/0)	0.0	90.8	4
2362	RF01972 H19 1	0.0 (0/0)	0.0	0.0 (0/0)	0.0	91.5	35
2363	RF02800 Rp sR47	0.0 (0/88)	0.0	0.0 (0/0)	0.1	92.4	2
2364	RF02632 Hrs10	0.0 (0/42)	0.0	0.0 (0/0)	0.1	94.6	2
2365	RF02333 TtnuHACA27	0.0 (0/17)	0.0	0.0 (0/0)	0.0	95.3	2
2366	RF00992 mir-593	0.0 (0/33)	0.0	0.0 (0/0)	0.0	95.0	2
2367	RF01956 NEAT1 2	0.0 (0/0)	0.0	0.0 (0/0)	0.0	84.1	13
2368	RF00852 mir-231	0.0 (0/26)	0.0	0.0 (0/0)	0.4	83.8	4
2369	RF01511 Afu 304	0.0 (0/0)	0.0	0.0 (0/1)	0.0	73.6	5
2370	RF01595 snoR19	0.0 (0/4)	0.0	0.0 (0/0)	0.0	79.2	4
2371	RF00839 mir-452	0.0 (0/33)	0.0	0.0 (0/0)	0.7	88.8	4
2372	RF02470 Ms IGR-8	0.0 (0/15)	0.0	0.0 (0/0)	1.2	64.5	3
2373	RF01560 DdR10	0.0 (0/0)	0.0	0.0 (0/0)	0.0	84.2	4
2374	RF01245 snR9	0.0 (0/38)	0.0	0.0 (0/0)	0.6	90.5	5
2375	RF01451 S pombe snR97	0.0 (0/26)	0.0	0.0 (0/0)	0.0	100.0	3
2376	RF02429 SpF14 sRNA	0.0 (0/36)	0.0	0.0 (0/0)	1.8	80.1	4
2377	RF02320 TtnuHACA13	0.0 (0/37)	0.0	0.0 (0/0)	0.1	92.5	2
2378	RF02279 TtnuCD1	0.0 (0/0)	0.0	0.0 (0/0)	0.0	96.8	2
2379	RF00803 mir-425	0.0 (0/20)	0.0	0.0 (0/0)	0.7	79.8	5
2380	RF01565 DdR15	0.0 (0/0)	0.0	0.0 (0/0)	0.0	84.7	3
2381	RF02882 MH s36	0.0 (0/28)	0.0	0.0 (0/0)	0.1	82.2	2
2382	RF01611 ceN108	0.0 (0/4)	0.0	0.0 (0/0)	0.0	87.6	3
2383	RF00742 MIR162 2	0.0 (0/25)	0.0	0.0 (0/0)	0.4	78.2	10
2384	RF01312 sR9	0.0 (0/1)	0.0	0.0 (0/0)	2.0	41.8	3
2385	RF01924 mir-2774	0.0 (0/17)	0.0	0.0 (0/0)	0.5	87.3	4
2386	RF00154 SNORD63	0.0 (0/4)	0.0	0.0 (0/0)	1.2	80.7	23
2387	RF02521 Virus CITE 7	0.0 (0/27)	0.0	0.0 (0/0)	0.0	89.4	2
2388	RF01667 rox1	0.0 (0/23)	0.0	0.0 (0/0)	0.0	94.3	3
2389	RF02340 DENV SLA	0.0 (0/22)	0.0	0.0 (0/0)	0.7	86.2	4
2390	RF02765 Ysr209	0.0 (0/8)	0.0	0.0 (0/0)	0.1	95.7	4
2391	RF01875 MIAT exon5 1	0.0 (0/0)	0.0	0.0 (0/0)	0.0	91.0	9
2392	RF02822 Srn266	0.0 (0/32)	0.0	0.0 (0/0)	0.6	85.4	4
2393	RF02484 GlsR21	0.0 (0/31)	0.0	0.0 (0/0)	0.2	87.2	3
2394	RF02618 SSRC34 2	0.0 (0/39)	0.0	0.0 (0/0)	0.0	98.9	3
2395	RF01097 RF site8	0.0 (0/12)	0.0	0.0 (0/0)	0.2	90.1	4
2396	RF01658 ceN81	0.0 (0/40)	0.0	0.0 (0/0)	0.2	90.4	3
2397	RF02204 WT1-AS 2	0.0 (0/0)	0.0	0.0 (0/0)	0.0	73.4	18
2398	RF01600 snoR25	0.0 (0/4)	0.0	0.0 (0/0)	1.0	91.0	4
2399	RF01843 neisseria FSE	0.0 (0/13)	0.0	0.0 (0/0)	0.5	92.9	4
2400	RF02188 ST7-OT4 2	0.0 (0/0)	0.0	0.0 (0/0)	0.0	70.5	29
2401	RF02313 TtnuHACA6	0.0 (0/36)	0.0	0.0 (0/0)	0.1	89.2	2
2402	RF01527 CrfA	0.0 (0/45)	0.0	0.0 (0/0)	0.1	90.4	2
2403	RF00611 SNORD111	0.0 (0/5)	0.0	0.0 (0/0)	2.0	75.5	5
2404	RF02562 CbSR14	0.0 (0/33)	0.0	0.0 (0/0)	0.2	100.0	2
2405	RF01621 ceN126	0.0 (0/35)	0.0	0.0 (0/0)	0.2	89.0	3
2406	RF02445 SpR14 sRNA	0.0 (0/17)	0.0	0.0 (0/0)	1.3	86.6	5
2407	RF01499 Afu 191	0.0 (0/0)	0.0	0.0 (0/0)	0.0	76.1	7
2408	RF02594 NsiR9	0.0 (0/41)	0.0	0.0 (0/0)	0.7	86.3	3
2409	RF01144 sR17	0.0 (0/0)	0.0	0.0 (0/0)	0.0	69.8	5
2410	RF01545 TB3Cs2H1	0.0 (0/15)	0.0	0.0 (0/0)	0.3	77.6	2
2411	RF00312 snoZ206	0.0 (0/5)	0.0	0.0 (0/0)	1.0	89.8	6
2412	RF01121 sR38	0.0 (0/0)	0.0	0.0 (0/0)	0.0	93.8	3
2413	RF02045 CDKN2B-AS 3	0.0 (0/0)	0.0	0.0 (0/0)	0.0	70.6	18
2414	RF01015 mir-885	0.0 (0/30)	0.0	0.0 (0/0)	0.9	77.5	3
2415	RF02180 ST7-AS1 2	0.0 (0/0)	0.0	0.0 (0/0)	0.0	76.8	24
2416	RF01327 CRISPR-DR14	0.0 (0/0)	0.0	0.0 (0/0)	0.0	89.0	5
2417	RF00818 mir-318	0.0 (0/26)	0.0	0.0 (0/0)	0.5	89.1	5
2418	RF01161 SNORD5	0.0 (0/0)	0.0	0.0 (0/0)	0.0	81.9	20
2419	RF01862 TB10Cs4H2	0.0 (0/16)	0.0	0.0 (0/2)	2.2	66.9	4
2420	RF02599 BASRCI408	0.0 (0/133)	0.0	0.0 (0/0)	0.4	98.6	3
2421	RF02776 SodC thermometer	0.0 (0/18)	0.0	0.0 (0/0)	0.0	98.9	2
2422	RF01939 mir-761	0.0 (0/26)	0.0	0.0 (0/0)	0.0	98.5	4
2423	RF02285 TtnuCD8	0.0 (0/0)	0.0	0.0 (0/0)	0.0	87.9	2
2424	RF02738 Rev24	0.0 (0/140)	0.0	0.0 (0/0)	1.0	82.3	4
2425	RF02575 DM SisR1	0.0 (0/107)	0.0	0.0 (0/0)	0.3	87.6	3

Continued on next page

	RNA family (seed alignment)	Sensitivity annotated bpairs that covary % (cov_bps/bps)	Power average power %	Positive Predictive Value covarying pairs in structure % (cov_bps/cov_pairs)	average substitutions per bpair	avg pairwise identity %	number of sequences
2426	RF01162 sn668	0.0 (0/4)	0.0	0.0 (0/0)	0.0	94.4	6
2427	RF02289 TtnuCD13	0.0 (0/0)	0.0	0.0 (0/0)	0.0	98.4	2
2428	RF01922 mir-654	0.0 (0/26)	0.0	0.0 (0/0)	0.3	79.7	6
2429	RF02243 Xoo8	0.0 (0/81)	0.0	0.0 (0/0)	0.7	85.5	4
2430	RF00314 snoZ182	0.0 (0/6)	0.0	0.0 (0/0)	0.5	96.1	6
2431	RF02661 icaR 5p UTR	0.0 (0/22)	0.0	0.0 (0/0)	0.0	98.7	2
2432	RF01226 snoZ5	0.0 (0/2)	0.0	0.0 (0/0)	0.0	84.0	8
2433	RF02522 Virus CITE 8	0.0 (0/21)	0.0	0.0 (0/0)	0.0	94.4	2
2434	RF00322 SNORA31	0.0 (0/38)	0.0	0.0 (0/0)	1.6	85.4	5
2435	RF02558 CbSR2	0.0 (0/80)	0.0	0.0 (0/0)	0.0	99.6	2
2436	RF01384 InvR	0.0 (0/17)	0.0	0.0 (0/0)	0.0	96.0	4
2437	RF01371 CRISPR-DR58	0.0 (0/5)	0.0	0.0 (0/0)	0.2	94.6	2
2438	RF01331 CRISPR-DR18	0.0 (0/5)	0.0	0.0 (0/0)	0.0	80.5	6
2439	RF02302 TtnuCD26	0.0 (0/0)	0.0	0.0 (0/0)	0.0	86.7	2
2440	RF02043 HOTTIP 4	0.0 (0/0)	0.0	0.0 (0/0)	0.0	81.6	19
2441	RF02044 CDKN2B-AS 2	0.0 (0/0)	0.0	0.0 (0/0)	0.0	79.4	6
2442	RF01328 CRISPR-DR17	0.0 (0/6)	0.0	0.0 (0/0)	0.0	94.7	3
2443	RF01133 sR3	0.0 (0/0)	0.0	0.0 (0/0)	0.0	67.2	19
2444	RF00914 mir-674	0.0 (0/32)	0.0	0.0 (0/0)	0.0	98.0	2
2445	RF01167 sn2429	0.0 (0/4)	0.0	0.0 (0/0)	0.5	90.3	4
2446	RF02387 sau-27	0.0 (0/27)	0.0	0.0 (0/0)	0.6	88.3	3
2447	RF02755 ES222	0.0 (0/36)	0.0	0.0 (0/0)	0.2	96.3	5
2448	RF00964 mir-938	0.0 (0/32)	0.0	0.0 (0/0)	0.1	89.2	2
2449	RF01134 sR30	0.0 (0/0)	0.0	0.0 (0/0)	0.0	88.8	3
2450	RF02633 Hrs21	0.0 (0/31)	0.0	0.0 (0/0)	0.0	94.8	2
2451	RF01666 rox2	0.0 (0/18)	0.0	0.0 (0/0)	0.4	86.4	4
2452	RF01374 CRISPR-DR61	0.0 (0/7)	0.0	0.0 (0/0)	0.0	94.6	2
2453	RF02149 MESTIT1 2	0.0 (0/0)	0.0	0.0 (0/0)	0.0	84.7	16
2454	RF02565 YrlB	0.0 (0/24)	0.0	0.0 (0/0)	0.2	75.6	2
2455	RF01521 CC1840	0.0 (0/25)	0.0	0.0 (0/0)	1.0	70.7	3
2456	RF01597 snoR22	0.0 (0/4)	0.0	0.0 (0/0)	0.0	90.7	3
2457	RF01912 mir-2807	0.0 (0/36)	0.0	0.0 (0/0)	0.9	85.8	7
2458	RF02109 DLEU2 5	0.0 (0/0)	0.0	0.0 (0/0)	0.0	81.3	30
2459	RF02308 TtnuCD34	0.0 (0/0)	0.0	0.0 (0/0)	0.0	93.8	2
2460	RF00933 mir-875	0.0 (0/25)	0.0	0.0 (0/1)	0.9	90.1	9
2461	RF02853 Ysr201	0.0 (0/19)	0.0	0.0 (0/0)	0.2	94.3	3
2462	RF02505 Atu L6	0.0 (0/36)	0.0	0.0 (0/0)	0.3	82.7	3
2463	RF02771 CnfY thermometer	0.0 (0/26)	0.0	0.0 (0/0)	0.0	94.6	3
2464	RF01106 SBWMV1 UPD-PKb	0.0 (0/10)	0.0	0.0 (0/0)	0.0	92.0	2
2465	RF02059 STnc50	0.0 (0/12)	0.0	0.0 (0/0)	0.0	90.2	2
2466	RF01682 P8	0.0 (0/25)	0.0	0.0 (0/0)	0.1	92.3	2
2467	RF01333 CRISPR-DR20	0.0 (0/8)	0.0	0.0 (0/0)	0.5	78.7	3
2468	RF00962 mir-586	0.0 (0/37)	0.0	0.0 (0/0)	0.1	94.8	2
2469	RF01765 srg1	0.0 (0/152)	0.0	0.0 (0/0)	0.1	87.7	2
2470	RF00805 mir-351	0.0 (0/30)	0.0	0.0 (0/0)	0.0	91.8	2
2471	RF01446 S pombe snR95	0.0 (0/60)	0.0	0.0 (0/0)	0.0	100.0	2
2472	RF02211 ZFAT-AS1 1	0.0 (0/0)	0.0	0.0 (0/0)	0.0	96.6	3
2473	RF02588 HCV package-SL6067	0.0 (0/9)	0.0	0.0 (0/0)	0.6	93.3	3
2474	RF02336 GlsR1 miR6	0.0 (0/19)	0.0	0.0 (0/0)	0.1	88.2	2
2475	RF02761 sR084	0.0 (0/18)	0.0	0.0 (0/0)	0.3	87.8	3
2476	RF01361 CRISPR-DR48	0.0 (0/7)	0.0	0.0 (0/0)	0.0	97.3	2
2477	RF00870 mir-423	0.0 (0/31)	0.0	0.0 (0/0)	1.2	88.8	4
2478	RF01598 snoR23	0.0 (0/3)	0.0	0.0 (0/0)	0.7	85.5	3
2479	RF02735 Sernc350	0.0 (0/172)	0.0	0.0 (0/0)	0.2	78.5	2
2480	RF02491 Gl U1	0.0 (0/37)	0.0	0.0 (0/0)	0.1	89.2	2
2481	RF02161 PART1 3	0.0 (0/0)	0.0	0.0 (0/0)	0.0	81.8	3
2482	RF01362 CRISPR-DR49	0.0 (0/6)	0.0	0.0 (0/0)	0.5	90.1	3
2483	RF01480 rli52	0.0 (0/26)	0.0	0.0 (0/0)	0.6	94.8	6
2484	RF02071 STnc280	0.0 (0/11)	0.0	0.0 (0/0)	0.4	83.8	3
2485	RF01937 mir-2780	0.0 (0/28)	0.0	0.0 (0/0)	0.3	96.2	4
2486	RF01652 ceN70	0.0 (0/3)	0.0	0.0 (0/0)	0.0	90.3	3
2487	RF01011 mir-605	0.0 (0/36)	0.0	0.0 (0/0)	0.0	94.0	2
2488	RF01340 CRISPR-DR29	0.0 (0/9)	0.0	0.0 (0/0)	0.0	100.0	2
2489	RF02813 PA5194 thermometer	0.0 (0/26)	0.0	0.0 (0/0)	0.3	64.4	2
2490	RF02572 babR 5UTR	0.0 (0/37)	0.0	0.0 (0/0)	0.0	99.0	3
2491	RF00313 snoZ173	0.0 (0/7)	0.0	0.0 (0/0)	0.0	99.4	4
2492	RF01930 bxd 2	0.0 (0/0)	0.0	0.0 (0/0)	0.0	84.1	5
2493	RF01891 TUG1 3	0.0 (0/0)	0.0	0.0 (0/0)	0.0	87.0	24
2494	RF02665 PsiU1-6	0.0 (0/44)	0.0	0.0 (0/0)	0.1	94.5	3
2495	RF01422 snoR116	0.0 (0/3)	0.0	0.0 (0/0)	0.3	74.4	5
2496	RF02490 GlsR27	0.0 (0/31)	0.0	0.0 (0/0)	0.1	91.1	2
2497	RF00743 mir-308	0.0 (0/24)	0.0	0.0 (0/0)	1.0	85.9	11
2498	RF02108 DLEU2 4	0.0 (0/0)	0.0	0.0 (0/0)	0.0	80.2	21
2499	RF01974 H19 3	0.0 (0/0)	0.0	0.0 (0/0)	0.0	94.1	5
2500	RF01448 S pombe snR93	0.0 (0/33)	0.0	0.0 (0/0)	0.0	100.0	2
2501	RF01991 SECIS 5	0.0 (0/20)	0.0	0.0 (0/0)	0.7	83.3	3
2502	RF02160 PART1 2	0.0 (0/0)	0.0	0.0 (0/0)	0.0	78.3	29
2503	RF01339 CRISPR-DR27	0.0 (0/4)	0.0	0.0 (0/0)	0.0	94.7	3
2504	RF00719 mir-326	0.0 (0/35)	0.0	0.0 (0/0)	0.9	92.4	7
2505	RF01150 sR11	0.0 (0/0)	0.0	0.0 (0/0)	0.0	57.8	8
2506	RF01398 isrP	0.0 (0/44)	0.0	0.0 (0/0)	0.3	96.8	7
2507	RF02011 mir-575	0.0 (0/31)	0.0	0.0 (0/0)	0.0	99.3	3
2508	RF01463 rli27	0.0 (0/10)	0.0	0.0 (0/0)	0.0	93.9	3
2509	RF02564 naRNA4	0.0 (0/24)	0.0	0.0 (0/0)	0.0	99.1	3

Continued on next page

	RNA family (seed alignment)	Sensitivity annotated bpairs that covary % (cov_bps/bps)	Power average power %	Positive Predictive Value covarying pairs in structure % (cov_bps/cov_pairs)	average substitutions per bpair	avg pairwise identity %	number of sequences
2510	RF02475 GlsR6	0.0 (0/0)	0.0	0.0 (0/0)	0.0	89.8	3
2511	RF02805 PyrR207	0.0 (0/18)	0.0	0.0 (0/0)	1.1	85.1	4
2512	RF02723 sno ZL1	0.0 (0/60)	0.0	0.0 (0/0)	0.5	89.3	5
2513	RF02511 PYLIS 4	0.0 (0/13)	0.0	0.0 (0/0)	0.2	81.0	2
2514	RF02517 mir-1017	0.0 (0/17)	0.0	0.0 (0/0)	0.5	88.1	7
2515	RF00178 mir-24	0.0 (0/16)	0.0	0.0 (0/0)	1.2	79.6	17
2516	RF02187 ST7-OT4 1	0.0 (0/0)	0.0	0.0 (0/0)	0.0	73.6	24
2517	RF01778 Evf-2 5p	0.0 (0/0)	0.0	0.0 (0/0)	0.0	86.5	13
2518	RF01155 snoR101	0.0 (0/0)	0.0	0.0 (0/0)	0.0	83.6	5
2519	RF01638 ceN48	0.0 (0/32)	0.0	0.0 (0/0)	0.5	90.6	5
2520	RF02444 SpR10 sRNA	0.0 (0/11)	0.0	0.0 (0/0)	0.6	91.8	5
2521	RF01391 isrH	0.0 (0/87)	0.0	0.0 (0/0)	0.3	97.2	6
2522	RF01573 DdR5	0.0 (0/0)	0.0	0.0 (0/0)	0.0	95.1	3
2523	RF02022 mir-1275	0.0 (0/24)	0.0	0.0 (0/0)	0.9	90.4	6
2524	RF02198 TTC28-AS1 1	0.0 (0/0)	0.0	0.0 (0/0)	0.0	67.2	14
2525	RF02536 AEV CRE	0.0 (0/43)	0.0	0.0 (0/0)	0.0	97.8	2
2526	RF00348 snoR9 plant	0.0 (0/3)	0.0	0.0 (0/0)	0.0	82.5	7
2527	RF00412 SNORA21	0.0 (0/24)	0.0	0.0 (0/0)	1.0	87.9	5
2528	RF01153 sn3060	0.0 (0/5)	0.0	0.0 (0/0)	0.2	98.0	3
2529	RF01569 DdR2	0.0 (0/0)	0.0	0.0 (0/0)	0.0	84.6	3
2530	RF01472 rli40	0.0 (0/81)	0.0	0.0 (0/2)	0.7	86.7	4
2531	RF02294 TtnuCD18	0.0 (0/0)	0.0	0.0 (0/0)	0.0	87.5	2
2532	RF02559 CbSR4	0.0 (0/52)	0.0	0.0 (0/0)	0.0	99.5	2
2533	RF02707 TeloSII ncR33	0.0 (0/68)	0.0	0.0 (0/0)	0.3	92.4	3
2534	RF01559 DdR1	0.0 (0/0)	0.0	0.0 (0/0)	0.0	84.8	3
2535	RF01971 RMST 10	0.0 (0/0)	0.0	0.0 (0/0)	0.0	84.2	21
2536	RF00642 mir-23	0.0 (0/14)	0.0	0.0 (0/0)	1.3	70.6	22
2537	RF00038 PrfA	0.0 (0/14)	0.0	0.0 (0/0)	0.9	87.4	11
2538	RF00101 SraC RyeA	0.0 (0/23)	0.0	0.0 (0/1)	0.3	86.2	13
2539	RF02080 STnc170	0.0 (0/77)	0.0	0.0 (0/0)	0.0	97.3	3
2540	RF01207 snR73	0.0 (0/0)	0.0	0.0 (0/0)	0.0	80.5	5
2541	RF01458 rli23	0.0 (0/31)	0.0	0.0 (0/0)	1.7	78.9	7
2542	RF01171 snoR4	0.0 (0/0)	0.0	0.0 (0/0)	0.0	61.5	2
2543	RF02583 Teg23	0.0 (0/124)	0.0	0.0 (0/0)	0.2	97.6	6
2544	RF01785 blv FSE	0.0 (0/9)	0.0	0.0 (0/0)	0.0	88.9	2
2545	RF01642 ceN54	0.0 (0/2)	0.0	0.0 (0/0)	0.0	91.7	3
2546	RF01439 S pombe snR36	0.0 (0/41)	0.0	0.0 (0/0)	0.0	100.0	2
2547	RF01147 sR12	0.0 (0/0)	0.0	0.0 (0/0)	0.0	69.2	5
2548	RF02579 tsr26	0.0 (0/33)	0.0	0.0 (0/0)	0.0	100.0	2
2549	RF00498 EAV LTH	0.0 (0/11)	0.0	0.0 (0/0)	0.0	98.1	6
2550	RF02408 snoR124	0.0 (0/4)	0.0	0.0 (0/0)	0.2	90.8	5
2551	RF02624 BSnc150	0.0 (0/7)	0.0	0.0 (0/0)	0.0	95.5	2
2552	RF00883 MIR820	0.0 (0/76)	0.0	0.0 (0/0)	0.1	93.4	2
2553	RF02758 RhlA thermometer	0.0 (0/36)	0.0	0.0 (0/0)	0.0	98.8	4
2554	RF01447 S pombe snR98	0.0 (0/59)	0.0	0.0 (0/0)	0.0	99.5	2
2555	RF02311 TtnuHACA3	0.0 (0/38)	0.0	0.0 (0/0)	0.1	91.5	2
2556	RF01890 lincRNA-p21 2	0.0 (0/0)	0.0	0.0 (0/0)	0.0	96.2	2
2557	RF01631 ceN41	0.0 (0/34)	0.0	0.0 (0/0)	0.6	78.4	3
2558	RF02210 WT1-AS 8	0.0 (0/0)	0.0	0.0 (0/0)	0.0	79.2	24
2559	RF02272 Vax2os1 3	0.0 (0/0)	0.0	0.0 (0/0)	0.0	70.1	5
2560	RF01354 CRISPR-DR45	0.0 (0/6)	0.0	0.0 (0/0)	0.0	94.4	3
2561	RF02597 shuA thermo	0.0 (0/7)	0.0	0.0 (0/0)	0.0	100.0	5
2562	RF00454 p27 CRE	0.0 (0/14)	0.0	0.0 (0/0)	1.1	93.2	12
2563	RF00117 C0719	0.0 (0/32)	0.0	0.0 (0/0)	0.2	98.5	9
2564	RF02907 patAB	0.0 (0/13)	0.0	0.0 (0/0)	0.0	98.2	2
2565	RF00940 mir-327	0.0 (0/20)	0.0	0.0 (0/0)	0.1	86.9	2
2566	RF02739 Rev41	0.0 (0/156)	0.0	0.0 (0/0)	0.3	90.6	3
2567	RF02365 Yfr17	0.0 (0/74)	0.0	0.0 (0/1)	0.3	85.5	3
2568	RF02331 TtnuHACA24	0.0 (0/37)	0.0	0.0 (0/0)	0.0	92.9	2
2569	RF01243 snR33	0.0 (0/39)	0.0	0.0 (0/0)	0.5	90.5	5
2570	RF02610 BSR0709	0.0 (0/96)	0.0	0.0 (0/0)	0.0	99.8	4
2571	RF01420 snoR113	0.0 (0/3)	0.0	0.0 (0/0)	0.0	72.4	5
2572	RF02719 sca ncR20	0.0 (0/30)	0.0	0.0 (0/0)	0.0	100.0	2
2573	RF02856 30 292	0.0 (0/30)	0.0	0.0 (0/0)	0.6	88.6	3
2574	RF00849 mir-60	0.0 (0/27)	0.0	0.0 (0/0)	0.2	88.7	4
2575	RF01947 KCNQ1OT1 2	0.0 (0/0)	0.0	0.0 (0/0)	0.0	83.6	18
2576	RF02143 HYMAI	0.0 (0/0)	0.0	0.0 (0/0)	0.0	74.5	16
2577	RF02554 ppoRNA	0.0 (0/36)	0.0	0.0 (0/0)	0.0	99.0	2
2578	RF02487 GlsR24	0.0 (0/35)	0.0	0.0 (0/0)	0.4	83.8	3
2579	RF00815 mir-244	0.0 (0/38)	0.0	0.0 (0/0)	0.7	78.3	4
2580	RF00863 mir-BART17	0.0 (0/30)	0.0	0.0 (0/0)	0.2	72.5	2
2581	RF00910 mir-873	0.0 (0/25)	0.0	0.0 (0/0)	0.2	96.5	3
2582	RF01156 SNORD123	0.0 (0/6)	0.0	0.0 (0/0)	1.0	87.9	12
2583	RF00088 SNORD30	0.0 (0/9)	0.0	0.0 (0/0)	3.0	82.0	20
2584	RF02334 TtnuHACA28	0.0 (0/33)	0.0	0.0 (0/0)	0.0	95.5	2
2585	RF01004 mir-557	0.0 (0/23)	0.0	0.0 (0/0)	0.0	93.0	2
2586	RF01490 rli51	0.0 (0/31)	0.0	0.0 (0/0)	0.9	92.6	9
2587	RF00476 snosnR61	0.0 (0/0)	0.0	0.0 (0/0)	0.0	68.7	56
2588	RF01931 bxd 3	0.0 (0/0)	0.0	0.0 (0/1)	0.0	82.5	4
2589	RF01013 mir-577	0.0 (0/36)	0.0	0.0 (0/0)	0.1	95.8	2
2590	RF01035 mir-887	0.0 (0/23)	0.0	0.0 (0/0)	0.4	78.1	3
2591	RF01146 sR15	0.0 (0/0)	0.0	0.0 (0/0)	0.0	52.7	2
2592	RF00701 mir-126	0.0 (0/21)	0.0	0.0 (0/0)	0.3	86.3	12
2593	RF00310 snoZ165	0.0 (0/3)	0.0	0.0 (0/0)	1.7	94.8	5

Continued on next page

	RNA family (seed alignment)	Sensitivity annotated bpairs that covary % (cov_bps/bps)	Power average power %	Positive Predictive Value covarying pairs in structure % (cov_bps/cov_pairs)	average substitutions per bpair	avg pairwise identity %	number of sequences
2594	RF02134 HTT-AS1 1	0.0 (0/0)	0.0	0.0 (0/0)	0.0	90.8	3
2595	RF02430 SpF19 sRNA	0.0 (0/36)	0.0	0.0 (0/0)	0.9	79.4	3
2596	RF02908 rli30	0.0 (0/38)	0.0	0.0 (0/1)	0.9	75.9	3
2597	RF01640 ceN51	0.0 (0/39)	0.0	0.0 (0/0)	0.2	89.2	3
2598	RF00666 mir-32	0.0 (0/30)	0.0	0.0 (0/0)	1.3	90.3	8
2599	RF00184 PVX 3	0.0 (0/8)	0.0	0.0 (0/0)	0.0	96.0	4
2600	RF01577 RNase P	0.0 (0/91)	0.0	0.0 (0/0)	0.0	99.7	2
2601	RF02463 Ms AS-1	0.0 (0/21)	0.0	0.0 (0/0)	1.7	80.0	5
2602	RF01529 CC3552	0.0 (0/28)	0.0	0.0 (0/0)	0.1	71.2	2
2603	RF02318 TtnuHACA11a	0.0 (0/36)	0.0	0.0 (0/0)	0.0	94.3	2
2604	RF01680 P5	0.0 (0/20)	0.0	0.0 (0/0)	0.0	93.0	2
2605	RF02655 BSR0441	0.0 (0/83)	0.0	0.0 (0/0)	0.0	99.2	5
2606	RF02105 DLEU2 1	0.0 (0/0)	0.0	0.0 (0/0)	0.0	79.9	27
2607	RF01404 PinT	0.0 (0/15)	0.0	0.0 (0/0)	0.1	99.3	4
2608	RF00819 mir-246	0.0 (0/33)	0.0	0.0 (0/1)	1.3	65.4	4
2609	RF01951 SOX2OT exon1	0.0 (0/0)	0.0	0.0 (0/0)	0.0	83.4	29
2610	RF02215 ZNFX1-AS1 1	0.0 (0/0)	0.0	0.0 (0/0)	0.0	69.1	15
2611	RF02750 ES003	0.0 (0/15)	0.0	0.0 (0/0)	0.4	94.7	8
2612	RF02286 TtnuCD9	0.0 (0/0)	0.0	0.0 (0/0)	0.0	96.4	2
2613	RF02504 Atu L1	0.0 (0/34)	0.0	0.0 (0/0)	0.2	92.5	4
2614	RF01952 SOX2OT exon2	0.0 (0/0)	0.0	0.0 (0/0)	0.0	86.0	14
2615	RF02702 AgvB	0.0 (0/17)	0.0	0.0 (0/0)	0.1	94.3	3
2616	RF02604 BASRCI153	0.0 (0/73)	0.0	0.0 (0/0)	0.1	92.1	2
2617	RF02304 TtnuCD29	0.0 (0/0)	0.0	0.0 (0/0)	0.0	98.7	2
2618	RF01935 bxd 7	0.0 (0/0)	0.0	0.0 (0/0)	0.0	92.4	3
2619	RF02455 Virus CITE 1	0.0 (0/27)	0.0	0.0 (0/0)	0.4	80.7	2
2620	RF00966 mir-676	0.0 (0/30)	0.0	0.0 (0/0)	0.6	87.7	6
2621	RF02171 RFPL3-AS1 1	0.0 (0/0)	0.0	0.0 (0/0)	0.0	90.7	4
2622	RF01533 TB10Cs4H3	0.0 (0/17)	0.0	0.0 (0/0)	1.8	71.8	3
2623	RF01335 CRISPR-DR22	0.0 (0/7)	0.0	0.0 (0/0)	2.3	79.5	9
2624	RF02585 HCV package	0.0 (0/8)	0.0	0.0 (0/0)	0.1	90.8	3
2625	RF00802 mir-207	0.0 (0/31)	0.0	0.0 (0/0)	1.3	83.5	6
2626	RF01314 mir-1227	0.0 (0/29)	0.0	0.0 (0/0)	0.1	95.5	3
2627	RF00851 mir-230	0.0 (0/37)	0.0	0.0 (0/0)	0.1	72.9	2
2628	RF00292 snoTBR5	0.0 (0/2)	0.0	0.0 (0/0)	0.0	81.0	6
2629	RF01917 MIR2587	0.0 (0/35)	0.0	0.0 (0/0)	0.2	94.1	4
2630	RF02300 TtnuCD24	0.0 (0/0)	0.0	0.0 (0/0)	0.0	98.3	2
2631	RF01583 snoR01	0.0 (0/2)	0.0	0.0 (0/0)	0.0	77.6	3
2632	RF02246 Six3os1 1	0.0 (0/0)	0.0	0.0 (0/0)	0.0	80.4	9
2633	RF00342 snoZ40	0.0 (0/5)	0.0	0.0 (0/0)	0.0	94.4	5
2634	RF01139 sR2	0.0 (0/0)	0.0	0.0 (0/0)	0.0	61.7	8
2635	RF02862 sRNA 1300	0.0 (0/77)	0.0	0.0 (0/0)	0.1	95.2	3
2636	RF01834 fiv FSE	0.0 (0/11)	0.0	0.0 (0/0)	0.0	93.1	5
2637	RF02147 MEG8 3	0.0 (0/0)	0.0	0.0 (0/0)	0.0	77.6	10
2638	RF01368 CRISPR-DR55	0.0 (0/0)	0.0	0.0 (0/0)	0.0	86.5	2
2639	RF02676 Esr41	0.0 (0/18)	0.0	0.0 (0/0)	0.1	98.2	3
2640	RF02620 BSnc119	0.0 (0/19)	0.0	0.0 (0/0)	0.1	97.0	3
2641	RF01636 ceN46	0.0 (0/30)	0.0	0.0 (0/0)	0.3	90.9	3
2642	RF02481 GlsR18	0.0 (0/30)	0.0	0.0 (0/0)	0.5	84.5	3
2643	RF01076 RF site2	0.0 (0/19)	0.0	0.0 (0/0)	0.1	90.3	2
2644	RF02529 SSRC8 1	0.0 (0/38)	0.0	0.0 (0/0)	0.2	94.7	3
2645	RF02698 Avalong thermometer	0.0 (0/36)	0.0	0.0 (0/0)	0.1	86.3	2
2646	RF02634 EF3314 EF3315	0.0 (0/45)	0.0	0.0 (0/0)	0.0	99.3	2
2647	RF00318 snoZ175	0.0 (0/3)	0.0	0.0 (0/0)	0.0	99.1	6
2648	RF00972 mir-651	0.0 (0/34)	0.0	0.0 (0/0)	0.8	81.9	4
2649	RF02407 snoR122	0.0 (0/0)	0.0	0.0 (0/0)	0.0	88.8	4
2650	RF00866 mir-BART3	0.0 (0/29)	0.0	0.0 (0/0)	0.2	81.3	2
2651	RF00901 MIR845 1	0.0 (0/31)	0.0	0.0 (0/0)	0.1	81.2	2
2652	RF00189 SNORD95	0.0 (0/4)	0.0	0.0 (0/0)	0.8	90.2	7
2653	RF02881 MH s25	0.0 (0/36)	0.0	0.0 (0/0)	0.4	67.2	2
2654	RF01501 Afu 199	0.0 (0/0)	0.0	0.0 (0/0)	0.0	71.2	3
2655	RF02785 snoRNA2	0.0 (0/21)	0.0	0.0 (0/0)	0.6	89.6	3
2656	RF01619 ceN115	0.0 (0/40)	0.0	0.0 (0/0)	0.0	100.0	2
2657	RF02550 RnaG	0.0 (0/28)	0.0	0.0 (0/0)	0.0	99.0	4
2658	RF02118 FMR1-AS1 2	0.0 (0/0)	0.0	0.0 (0/0)	0.0	79.6	25
2659	RF01174 snoU43	0.0 (0/0)	0.0	0.0 (0/0)	0.0	75.8	11
2660	RF02146 MEG8 2	0.0 (0/0)	0.0	0.0 (0/1)	0.0	73.0	12
2661	RF01273 sR34	0.0 (0/9)	0.0	0.0 (0/0)	0.0	92.6	4
2662	RF00289 SNORND104	0.0 (0/2)	0.0	0.0 (0/0)	1.0	85.6	18
2663	RF02857 30 255	0.0 (0/46)	0.0	0.0 (0/0)	0.1	92.9	2
2664	RF00462 IRES APC	0.0 (0/11)	0.0	0.0 (0/0)	1.6	85.4	6
2665	RF00472 snosnR55	0.0 (0/0)	0.0	0.0 (0/0)	0.0	74.8	6
2666	RF01965 RMST 4	0.0 (0/0)	0.0	0.0 (0/0)	0.0	84.4	17
2667	RF02212 ZFAT-AS1 2	0.0 (0/0)	0.0	0.0 (0/0)	0.0	69.0	11
2668	RF00355 snoR28	0.0 (0/3)	0.0	0.0 (0/1)	1.7	81.6	9
2669	RF01006 mir-601	0.0 (0/20)	0.0	0.0 (0/0)	0.1	96.6	3
2670	RF02116 FAS-AS1	0.0 (0/0)	0.0	0.0 (0/0)	0.0	68.0	19
2671	RF00796 mir-48	0.0 (0/30)	0.0	0.0 (0/0)	1.0	70.3	4
2672	RF00574 SNORD69	0.0 (0/10)	0.0	0.0 (0/0)	2.3	88.1	11
2673	RF02322 TtnuHACA15	0.0 (0/32)	0.0	0.0 (0/0)	0.0	96.6	2
2674	RF02651 SorY	0.0 (0/24)	0.0	0.0 (0/0)	0.0	97.6	2
2675	RF00592 SNORD78	0.0 (0/0)	0.0	0.0 (0/0)	0.0	82.1	19
2676	RF02038 SPRY4-IT1 1	0.0 (0/0)	0.0	0.0 (0/1)	0.0	78.5	23
2677	RF00687 mir-136	0.0 (0/32)	0.0	0.0 (0/0)	0.0	93.9	2

Continued on next page

	RNA family (seed alignment)	Sensitivity annotated bpairs that covary % (cov_bps/bps)	Power average power %	Positive Predictive Value covarying pairs in structure % (cov_bps/cov_pairs)	average substitutions per bpair	avg pairwise identity %	number of sequences
2678	RF02174 SMAD5-AS1 2	0.0 (0/0)	0.0	0.0 (0/0)	0.0	74.6	20
2679	RF02169 PVT1 6	0.0 (0/0)	0.0	0.0 (0/0)	0.0	71.2	7
2680	RF01485 rli61	0.0 (0/32)	0.0	0.0 (0/0)	0.0	99.5	4
2681	RF00315 snoJ33	0.0 (0/6)	0.0	0.0 (0/0)	2.3	79.5	5
2682	RF02115 FAM13A-AS1 2	0.0 (0/0)	0.0	0.0 (0/0)	0.0	69.3	10
2683	RF01160 sn2841	0.0 (0/4)	0.0	0.0 (0/0)	0.0	95.8	2
2684	RF02151 MIMT1 1	0.0 (0/0)	0.0	0.0 (0/0)	0.0	69.7	21
2685	RF02388 sau-30	0.0 (0/12)	0.0	0.0 (0/0)	0.1	85.9	2
2686	RF02456 Diantho 3 UTR	0.0 (0/32)	0.0	0.0 (0/0)	0.2	81.2	2
2687	RF02672 SprX	0.0 (0/40)	0.0	0.0 (0/0)	0.0	98.7	3
2688	RF01358 CRISPR-DR39	0.0 (0/4)	0.0	0.0 (0/0)	1.5	81.7	3
2689	RF02753 ES173	0.0 (0/13)	0.0	0.0 (0/0)	0.0	95.0	4
2690	RF01525 TB10Cs2H1	0.0 (0/10)	0.0	0.0 (0/2)	0.1	71.7	4
2691	RF02066 STnc320	0.0 (0/12)	0.0	0.0 (0/0)	0.0	93.3	3
2692	RF00275 SNORD56	0.0 (0/7)	0.0	0.0 (0/0)	2.4	80.0	7
2693	RF00864 mir-BART20	0.0 (0/28)	0.0	0.0 (0/0)	0.1	81.2	2
2694	RF01454 RCNMV 5UTR	0.0 (0/23)	0.0	0.0 (0/0)	0.9	81.8	3
2695	RF02154 NCRUPAR 1	0.0 (0/0)	0.0	0.0 (0/0)	0.0	79.3	9
2696	RF02700 HtrA thermometer	0.0 (0/12)	0.0	0.0 (0/0)	0.2	93.8	4
2697	RF02477 GlsR9	0.0 (0/4)	0.0	0.0 (0/0)	0.0	93.3	2
2698	RF02358 hsp17	0.0 (0/12)	0.0	0.0 (0/0)	0.8	73.3	3
2699	RF02122 FTX 5	0.0 (0/0)	0.0	0.0 (0/0)	0.0	71.4	14
2700	RF01783 b55	0.0 (0/22)	0.0	0.0 (0/0)	0.0	100.0	2
2701	RF00330 snoZ43	0.0 (0/7)	0.0	0.0 (0/0)	1.0	83.0	7
2702	RF02359 MS2	0.0 (0/7)	0.0	0.0 (0/0)	0.0	88.5	5
2703	RF01894 PCA3 2	0.0 (0/0)	0.0	0.0 (0/0)	0.0	89.6	20
2704	RF02070 STnc300	0.0 (0/33)	0.0	0.0 (0/0)	0.4	84.6	4
2705	RF00218 SNORD46	0.0 (0/6)	0.0	0.0 (0/0)	2.7	90.9	5
2706	RF01369 CRISPR-DR56	0.0 (0/9)	0.0	0.0 (0/0)	0.0	94.6	2
2707	RF00244 mir-26	0.0 (0/18)	0.0	0.0 (0/0)	0.5	74.4	5
2708	RF02103 DLEU1 1	0.0 (0/0)	0.0	0.0 (0/0)	0.0	72.4	26
2709	RF02827 Scr6106	0.0 (0/32)	0.0	0.0 (0/0)	0.5	90.0	4
2710	RF00338 snoR53	0.0 (0/11)	0.0	0.0 (0/0)	1.1	94.3	5
2711	RF01104 SBWMV2 UPD-PKb	0.0 (0/10)	0.0	0.0 (0/0)	0.2	90.8	3
2712	RF01628 ceN38	0.0 (0/39)	0.0	0.0 (0/0)	0.6	84.7	3
2713	RF01478 rli47	0.0 (0/145)	0.0	0.0 (0/0)	0.3	97.4	9
2714	RF00859 mir-234	0.0 (0/29)	0.0	0.0 (0/0)	0.8	72.6	4
2715	RF02338 GlsR16 miR3	0.0 (0/20)	0.0	0.0 (0/0)	0.5	92.6	3
2716	RF01519 CC0196	0.0 (0/9)	0.0	0.0 (0/0)	0.0	100.0	2
2717	RF02142 HOXA11-AS1 6	0.0 (0/0)	0.0	0.0 (0/0)	0.0	87.9	21
2718	RF00919 mir-874	0.0 (0/21)	0.0	0.0 (0/0)	0.0	99.1	4
2719	RF01538 TB11Cs3H1	0.0 (0/17)	0.0	0.0 (0/0)	0.8	74.8	4
2720	RF02041 HOTTIP 2	0.0 (0/0)	0.0	0.0 (0/0)	0.0	81.6	25
2721	RF01880 Xist exon1	0.0 (0/0)	0.0	0.0 (0/0)	0.0	78.8	24
2722	RF01122 sR39	0.0 (0/0)	0.0	0.0 (0/1)	0.0	82.4	5
2723	RF02130 GNAS-AS1 4	0.0 (0/0)	0.0	0.0 (0/0)	0.0	79.2	18
2724	RF00473 snosnR54	0.0 (0/0)	0.0	0.0 (0/0)	0.0	78.0	8
2725	RF02791 CcsR1	0.0 (0/20)	0.0	0.0 (0/0)	0.5	68.7	2
2726	RF00891 mir-671	0.0 (0/29)	0.0	0.0 (0/0)	0.0	98.2	3
2727	RF02312 TtnuHACA5	0.0 (0/44)	0.0	0.0 (0/0)	0.0	95.4	2
2728	RF02873 AS-traI	0.0 (0/22)	0.0	0.0 (0/0)	0.8	89.2	3
2729	RF02797 PssrA	0.0 (0/24)	0.0	0.0 (0/0)	0.5	91.5	6
2730	RF02629 RmaA	0.0 (0/32)	0.0	0.0 (0/0)	0.0	99.1	2
2731	RF00873 mir-550	0.0 (0/31)	0.0	0.0 (0/0)	0.1	95.9	3
2732	RF01079 RF site3	0.0 (0/8)	0.0	0.0 (0/0)	0.1	94.9	2
2733	RF01323 CRISPR-DR10	0.0 (0/6)	0.0	0.0 (0/0)	0.0	71.1	5
2734	RF02613 BSR0739	0.0 (0/56)	0.0	0.0 (0/0)	0.0	99.1	4
2735	RF01580 RUF3	0.0 (0/46)	0.0	0.0 (0/0)	0.2	92.5	3
2736	RF01634 ceN44	0.0 (0/3)	0.0	0.0 (0/0)	0.0	95.4	3
2737	RF02458 Virus CITE 3	0.0 (0/35)	0.0	0.0 (0/0)	0.5	63.0	2
2738	RF02153 NBR2	0.0 (0/0)	0.0	0.0 (0/0)	0.0	92.7	26
2739	RF02040 HOTTIP 1	0.0 (0/0)	0.0	0.0 (0/1)	0.0	83.4	7
2740	RF01319 CRISPR-DR6	0.0 (0/4)	0.0	0.0 (0/0)	0.0	91.9	7
2741	RF02206 WT1-AS 4	0.0 (0/0)	0.0	0.0 (0/1)	0.0	70.0	12
2742	RF00939 mir-504	0.0 (0/30)	0.0	0.0 (0/0)	0.3	91.7	6
2743	RF02319 TtnuHACA12	0.0 (0/40)	0.0	0.0 (0/0)	0.5	83.0	2
2744	RF01181 snR77	0.0 (0/0)	0.0	0.0 (0/1)	0.0	83.2	15
2745	RF02912 AAC AAD leader	0.0 (0/18)	0.0	0.0 (0/0)	0.8	86.0	4
2746	RF02373 PyrD leader	0.0 (0/6)	0.0	0.0 (0/0)	1.3	80.9	27
2747	RF00967 mir-281	0.0 (0/29)	0.0	0.0 (0/0)	0.1	78.0	2
2748	RF01547 TB6Cs1H3	0.0 (0/17)	0.0	0.0 (0/0)	1.9	78.5	5
2749	RF00739 MIR476	0.0 (0/36)	0.0	0.0 (0/0)	0.1	88.2	2
2750	RF02636 EF1368 EF1369	0.0 (0/215)	0.0	0.0 (0/0)	0.0	99.1	4
2751	RF02474 GlsR5	0.0 (0/0)	0.0	0.0 (0/0)	0.0	96.5	2
2752	RF02638 EF0605 EF0606	0.0 (0/53)	0.0	0.0 (0/0)	0.0	100.0	2
2753	RF02417 VR-RNA	0.0 (0/111)	0.0	0.0 (0/0)	0.1	98.1	3
2754	RF01804 Lambda thermo	0.0 (0/20)	0.0	0.0 (0/0)	1.0	89.8	5
2755	RF01111 SBWMV2 UPD-PKk	0.0 (0/8)	0.0	0.0 (0/0)	0.1	95.2	4
2756	RF01024 mir-944	0.0 (0/37)	0.0	0.0 (0/0)	0.0	94.3	2
2757	RF02350 psRNA6	0.0 (0/103)	0.0	0.0 (0/0)	0.2	71.7	2
2758	RF01627 ceN36-1	0.0 (0/27)	0.0	0.0 (0/0)	0.3	88.4	5
2759	RF01432 snoR143	0.0 (0/38)	0.0	0.0 (0/0)	1.5	60.6	3
2760	RF00793 mir-497	0.0 (0/34)	0.0	0.0 (0/0)	0.7	89.6	8
2761	RF02612 BSR1350	0.0 (0/52)	0.0	0.0 (0/0)	0.0	98.9	3

Continued on next page

	RNA family (seed alignment)	Sensitivity annotated bpairs that covary % (cov_bps/bps)	Power average power %	Positive Predictive Value covarying pairs in structure % (cov_bps/cov_pairs)	average substitutions per bpair	avg pairwise identity %	number of sequences
2762	RF02545 SSU trypano mito	0.0 (0/175)	0.0	0.0 (0/0)	0.7	82.9	4
2763	RF01329 CRISPR-DR15	0.0 (0/9)	0.0	0.0 (0/0)	0.0	80.2	3
2764	RF02141 HOXA11-AS1 5	0.0 (0/0)	0.0	0.0 (0/0)	0.0	78.9	28
2765	RF02631 Hrs13	0.0 (0/21)	0.0	0.0 (0/0)	0.1	87.7	2
2766	RF01630 ceN40	0.0 (0/3)	0.0	0.0 (0/0)	1.7	84.7	4
2767	RF01145 sR14	0.0 (0/0)	0.0	0.0 (0/0)	0.0	67.3	8
2768	RF01810 pntA	0.0 (0/18)	0.0	0.0 (0/0)	0.7	93.1	10
2769	RF00337 snoZ112	0.0 (0/30)	0.0	0.0 (0/0)	0.2	92.7	6
2770	RF02508 Atu Ti4	0.0 (0/20)	0.0	0.0 (0/0)	0.9	88.0	3
2771	RF02745 Rev42 5p UTR	0.0 (0/136)	0.0	0.0 (0/0)	0.4	88.9	3
2772	RF01460 rliH	0.0 (0/119)	0.0	0.0 (0/0)	0.2	92.7	3
2773	RF01438 S pombe snR35	0.0 (0/32)	0.0	0.0 (0/0)	1.1	85.6	4
2774	RF00902 mir-791	0.0 (0/25)	0.0	0.0 (0/0)	0.9	70.7	3
2775	RF02515 AfaR	0.0 (0/51)	0.0	0.0 (0/0)	0.0	98.7	2
2776	RF00205 snoR41	0.0 (0/6)	0.0	0.0 (0/0)	1.8	90.7	7
2777	RF01970 RMST 9	0.0 (0/0)	0.0	0.0 (0/0)	0.0	89.1	28
2778	RF01929 bxd 1	0.0 (0/0)	0.0	0.0 (0/0)	0.0	87.0	6
2779	RF00718 mir-431	0.0 (0/35)	0.0	0.0 (0/0)	0.7	88.9	9
2780	RF01351 CRISPR-DR42	0.0 (0/0)	0.0	0.0 (0/0)	0.0	92.0	3
2781	RF02645 Cis2 sRNA	0.0 (0/52)	0.0	0.0 (0/0)	0.2	80.4	2
2782	RF01326 CRISPR-DR13	0.0 (0/5)	0.0	0.0 (0/0)	0.0	92.8	4
2783	RF02799 CssrB	0.0 (0/53)	0.0	0.0 (0/0)	0.1	98.9	3
2784	RF02259 Vax2os1 1	0.0 (0/0)	0.0	0.0 (0/0)	0.0	88.0	3
2785	RF01081 SBWMV1 UPD-PKe	0.0 (0/9)	0.0	0.0 (0/0)	0.8	84.6	3
2786	RF01602 plasmodium snoR27	0.0 (0/34)	0.0	0.0 (0/0)	0.6	83.5	4
2787	RF01158 SNORD108	0.0 (0/3)	0.0	0.0 (0/0)	0.3	77.9	4
2788	RF02325 TtnuHACA18	0.0 (0/34)	0.0	0.0 (0/0)	0.4	85.6	3
2789	RF02824 LPR10	0.0 (0/16)	0.0	0.0 (0/0)	0.9	79.4	3
2790	RF02706 TeloSII ncR30	0.0 (0/36)	0.0	0.0 (0/0)	0.0	94.2	2
2791	RF02150 MESTIT1 3	0.0 (0/0)	0.0	0.0 (0/0)	0.0	78.7	18
2792	RF02166 PVT1 3	0.0 (0/0)	0.0	0.0 (0/0)	0.0	67.8	25
2793	RF01571 DdR35	0.0 (0/15)	0.0	0.0 (0/0)	0.0	98.9	3
2794	RF01135 sR24	0.0 (0/2)	0.0	0.0 (0/0)	0.0	95.2	3
2795	RF02113 DLG2-AS1 2	0.0 (0/0)	0.0	0.0 (0/0)	0.0	74.6	23
2796	RF02467 Ms IGR-2	0.0 (0/13)	0.0	0.0 (0/0)	0.4	84.4	3
2797	RF01475 rli45	0.0 (0/14)	0.0	0.0 (0/0)	1.3	84.7	6
2798	RF00811 mir-84	0.0 (0/26)	0.0	0.0 (0/0)	0.5	71.5	4
2799	RF02277 Hammerhead HH10	0.0 (0/34)	0.0	0.0 (0/0)	0.4	90.9	18
2800	RF01648 ceN65	0.0 (0/3)	0.0	0.0 (0/0)	0.0	95.9	3
2801	RF01957 NEAT1 3	0.0 (0/0)	0.0	0.0 (0/0)	0.0	87.5	18
2802	RF02200 TTC28-AS1 3	0.0 (0/0)	0.0	0.0 (0/0)	0.0	67.7	6
2803	RF00784 mir-486	0.0 (0/24)	0.0	0.0 (0/1)	0.9	88.8	12
2804	RF02010 mir-3180	0.0 (0/34)	0.0	0.0 (0/0)	0.0	100.0	3
2805	RF01212 sn2524	0.0 (0/3)	0.0	0.0 (0/0)	0.7	70.8	2
2806	RF01901 mir-284	0.0 (0/34)	0.0	0.0 (0/0)	0.3	92.7	10
2807	RF02573 LLnc147	0.0 (0/30)	0.0	0.0 (0/0)	0.1	94.1	2
2808	RF02879 MH s10	0.0 (0/30)	0.0	0.0 (0/1)	0.8	80.0	3
2809	RF01613 ceN109	0.0 (0/3)	0.0	0.0 (0/1)	0.0	83.1	3
2810	RF02861 IR 84	0.0 (0/54)	0.0	0.0 (0/0)	1.0	75.8	3
2811	RF01884 LINC00901	0.0 (0/0)	0.0	0.0 (0/0)	0.0	88.8	30
2812	RF00997 mir-942	0.0 (0/36)	0.0	0.0 (0/0)	0.0	95.3	2
2813	RF02397 sau-5971	0.0 (0/35)	0.0	0.0 (0/0)	0.1	99.3	3
2814	RF02078 STnc210	0.0 (0/24)	0.0	0.0 (0/0)	0.1	80.5	2
2815	RF00227 FIE3	0.0 (0/9)	0.0	0.0 (0/0)	1.7	65.2	5
2816	RF02443 SpR08 sRNA	0.0 (0/17)	0.0	0.0 (0/0)	0.3	97.3	3
2817	RF02377 SurA	0.0 (0/71)	0.0	0.0 (0/0)	0.3	94.5	4
2818	RF02654 MicL	0.0 (0/105)	0.0	0.0 (0/0)	0.0	99.0	2
2819	RF00116 C0465	0.0 (0/23)	0.0	0.0 (0/0)	0.4	98.9	8
2820	RF01936 mir-63	0.0 (0/48)	0.0	0.0 (0/0)	1.4	85.5	5
2821	RF02843 RefA5	0.0 (0/20)	0.0	0.0 (0/0)	0.3	82.8	3
2822	RF02584 KRAS 3UTR	0.0 (0/10)	0.0	0.0 (0/0)	0.0	98.0	2
2823	RF02780 PutA thermometer	0.0 (0/15)	0.0	0.0 (0/1)	1.3	83.8	4
2824	RF01479 rli48	0.0 (0/41)	0.0	0.0 (0/0)	0.2	87.0	3
2825	RF01364 CRISPR-DR51	0.0 (0/6)	0.0	0.0 (0/0)	0.3	78.4	2
2826	RF02637 EF0408 EF0409	0.0 (0/127)	0.0	0.0 (0/0)	0.0	98.4	3
2827	RF00387 IRES FGF1	0.0 (0/51)	0.0	0.0 (0/0)	1.2	87.2	6
2828	RF01443 S pombe snR91	0.0 (0/24)	0.0	0.0 (0/0)	0.0	99.3	2
2829	RF01349 CRISPR-DR40	0.0 (0/10)	0.0	0.0 (0/0)	0.0	91.1	3
2830	RF00799 mir-354	0.0 (0/34)	0.0	0.0 (0/0)	0.1	91.3	4
2831	RF00895 mir-786	0.0 (0/32)	0.0	0.0 (0/0)	0.3	64.4	2
2832	RF02649 Trans44 sRNA	0.0 (0/83)	0.0	0.0 (0/0)	0.2	85.3	2
2833	RF02615 BSR0626	0.0 (0/30)	0.0	0.0 (0/0)	0.0	99.2	2
2834	RF02089 CLRN1-AS1	0.0 (0/0)	0.0	0.0 (0/0)	0.0	73.6	19
2835	RF02894 S35	0.0 (0/95)	0.0	0.0 (0/0)	1.2	61.4	3
2836	RF00569 SNORD19	0.0 (0/4)	0.0	0.0 (0/0)	1.8	83.7	22
2837	RF00903 mir-359	0.0 (0/35)	0.0	0.0 (0/0)	0.2	74.7	2
2838	RF02693 psm mec RNA	0.0 (0/38)	0.0	0.0 (0/0)	0.5	91.8	4
2839	RF02593 NsiR8	0.0 (0/52)	0.0	0.0 (0/0)	0.1	94.8	2
2840	RF02906 sRNA154	0.0 (0/32)	0.0	0.0 (0/0)	0.6	80.5	3
2841	RF00748 mir-433	0.0 (0/22)	0.0	0.0 (0/0)	0.0	94.4	5
2842	RF01966 RMST 5	0.0 (0/0)	0.0	0.0 (0/0)	0.0	87.2	21
2843	RF02236 asX2	0.0 (0/18)	0.0	0.0 (0/0)	0.2	81.2	2
2844	RF02125 JPX 2	0.0 (0/0)	0.0	0.0 (0/1)	0.0	74.8	11
2845	RF02818 V AS5	0.0 (0/38)	0.0	0.0 (0/0)	0.9	69.2	3

Continued on next page

	RNA family (seed alignment)	Sensitivity annotated bpairs that covary % (cov_bps/bps)	Power average power %	Positive Predictive Value covarying pairs in structure % (cov_bps/cov_pairs)	average substitutions per bpair	avg pairwise identity %	number of sequences
2846	RF00159 snoZ168	0.0 (0/4)	0.0	0.0 (0/1)	0.0	84.5	6
2847	RF00987 mir-589	0.0 (0/34)	0.0	0.0 (0/0)	0.1	96.0	2
2848	RF02810 Lst thermometer	0.0 (0/32)	0.0	0.0 (0/0)	0.1	97.1	2
2849	RF01176 snR78	0.0 (0/2)	0.0	0.0 (0/0)	4.5	72.1	8
2850	RF02731 JA03	0.0 (0/78)	0.0	0.0 (0/0)	0.1	93.7	3
2851	RF01978 HOTAIRM1 4	0.0 (0/0)	0.0	0.0 (0/0)	0.0	86.2	10
2852	RF00262 sar	0.0 (0/20)	0.0	0.0 (0/0)	0.8	82.3	4
2853	RF01376 CRISPR-DR63	0.0 (0/8)	0.0	0.0 (0/0)	0.0	97.3	2
2854	RF00824 mir-50	0.0 (0/37)	0.0	0.0 (0/0)	2.0	70.5	5
2855	RF00975 MIR845 2	0.0 (0/30)	0.0	0.0 (0/0)	0.0	91.6	2
2856	RF01343 CRISPR-DR33	0.0 (0/6)	0.0	0.0 (0/0)	0.2	66.7	3
2857	RF01002 mir-936	0.0 (0/30)	0.0	0.0 (0/0)	1.7	78.0	4
2858	RF02176 SMAD5-AS1 4	0.0 (0/0)	0.0	0.0 (0/0)	0.0	82.9	19
2859	RF00955 mir-1829	0.0 (0/23)	0.0	0.0 (0/0)	0.6	87.8	3
2860	RF02886 npcTB 6715	0.0 (0/61)	0.0	0.0 (0/3)	1.1	71.8	3
2861	RF00351 snoR20	0.0 (0/3)	0.0	0.0 (0/0)	2.0	83.7	4
2862	RF00249 mir-46	0.0 (0/22)	0.0	0.0 (0/0)	1.3	73.9	11
2863	RF00899 mir-235	0.0 (0/19)	0.0	0.0 (0/0)	0.1	78.6	2
2864	RF02145 MEG8 1	0.0 (0/0)	0.0	0.0 (0/0)	0.0	74.3	23
2865	RF01882 TUG1 1	0.0 (0/0)	0.0	0.0 (0/0)	0.0	89.9	21
2866	RF02310 TtnuHACA2	0.0 (0/34)	0.0	0.0 (0/0)	0.1	92.4	3
2867	RF02299 TtnuCD23	0.0 (0/0)	0.0	0.0 (0/0)	0.0	93.1	2
2868	RF00960 mir-661	0.0 (0/30)	0.0	0.0 (0/0)	1.5	78.2	4
2869	RF02478 GlsR10	0.0 (0/3)	0.0	0.0 (0/0)	0.0	97.1	2
2870	RF01624 ceN27	0.0 (0/3)	0.0	0.0 (0/0)	0.3	90.0	2
2871	RF01325 CRISPR-DR12	0.0 (0/10)	0.0	0.0 (0/0)	1.0	89.0	5
2872	RF02816 HBV PRE 1151-1410	0.0 (0/82)	0.0	0.0 (0/0)	0.0	94.6	2
2873	RF02743 Sernc389	0.0 (0/210)	0.0	0.0 (0/0)	0.1	86.5	2
2874	RF00965 mir-549	0.0 (0/29)	0.0	0.0 (0/0)	0.0	97.9	2
2875	RF02433 SpF36 sRNA	0.0 (0/14)	0.0	0.0 (0/0)	0.1	91.5	4
2876	RF01180 snoR110	0.0 (0/18)	0.0	0.0 (0/0)	0.0	91.6	2
2877	RF00329 snoZ162	0.0 (0/13)	0.0	0.0 (0/0)	1.2	83.1	9
2878	RF01609 ceN103	0.0 (0/3)	0.0	0.0 (0/0)	0.0	78.2	4
2879	RF01000 mir-580	0.0 (0/40)	0.0	0.0 (0/0)	0.1	95.9	2
2880	RF00893 MIR854	0.0 (0/18)	0.0	0.0 (0/0)	0.0	97.9	3
2881	RF02694 RalA	0.0 (0/48)	0.0	0.0 (0/2)	0.5	94.6	10
2882	RF02764 Ysr190	0.0 (0/21)	0.0	0.0 (0/0)	0.3	92.0	4
2883	RF01310 sR7	0.0 (0/0)	0.0	0.0 (0/0)	0.0	70.9	5
2884	RF00929 mir-574	0.0 (0/32)	0.0	0.0 (0/0)	0.2	93.5	3
2885	RF02480 GlsR14	0.0 (0/0)	0.0	0.0 (0/0)	0.0	93.1	3
2886	RF02047 Sphinx 2	0.0 (0/0)	0.0	0.0 (0/0)	0.0	79.9	37
2887	RF02670 scaDm46E3	0.0 (0/37)	0.0	0.0 (0/0)	0.0	97.8	2
2888	RF01471 rliB	0.0 (0/49)	0.0	0.0 (0/0)	1.2	69.7	4
2889	RF02756 ES239	0.0 (0/34)	0.0	0.0 (0/0)	0.3	96.8	5
2890	RF01561 DdR11	0.0 (0/0)	0.0	0.0 (0/0)	0.0	88.7	3
2891	RF01881 Xist exon4	0.0 (0/0)	0.0	0.0 (0/0)	0.0	87.6	25
2892	RF00984 mir-576	0.0 (0/38)	0.0	0.0 (0/0)	0.1	91.8	2
2893	RF01102 PK1-TEV CVMV	0.0 (0/11)	0.0	0.0 (0/0)	0.2	80.6	2
2894	RF02798 CssrA	0.0 (0/29)	0.0	0.0 (0/0)	0.4	91.2	4
2895	RF02016 mir-1183	0.0 (0/25)	0.0	0.0 (0/0)	0.3	98.5	3
2896	RF01165 sn2317	0.0 (0/3)	0.0	0.0 (0/0)	0.0	85.0	4
2897	RF00909 mir-883	0.0 (0/28)	0.0	0.0 (0/0)	0.4	85.3	3
2898	RF01564 DdR14	0.0 (0/0)	0.0	0.0 (0/0)	0.0	100.0	2
2899	RF02015 mir-1287	0.0 (0/32)	0.0	0.0 (0/0)	0.2	91.8	3
2900	RF02273 FsrA	0.0 (0/20)	0.0	0.0 (0/0)	0.0	96.1	6
2901	RF02741 Rev44	0.0 (0/101)	0.0	0.0 (0/0)	0.3	92.5	3
2902	RF02242 Xoo5	0.0 (0/30)	0.0	0.0 (0/0)	0.4	80.1	3
2903	RF01541 TB11Cs4H3	0.0 (0/13)	0.0	0.0 (0/0)	0.5	76.1	3
2904	RF01798 g2	0.0 (0/21)	0.0	0.0 (0/0)	0.0	100.0	2
2905	RF00453 Cardiovirus CRE	0.0 (0/10)	0.0	0.0 (0/0)	0.8	81.3	4
2906	RF01920 mir-764	0.0 (0/30)	0.0	0.0 (0/0)	0.2	91.0	6
2907	RF01976 HOTAIRM1 2	0.0 (0/0)	0.0	0.0 (0/0)	0.0	86.8	7
2908	RF01950 KCNQ1OT1 5	0.0 (0/0)	0.0	0.0 (0/0)	0.0	88.8	15
2909	RF00158 SNORD82	0.0 (0/5)	0.0	0.0 (0/0)	1.4	80.3	27
2910	RF00393 SNORA8	0.0 (0/37)	0.0	0.0 (0/0)	0.3	89.7	5
2911	RF02494 Gl U6	0.0 (0/19)	0.0	0.0 (0/0)	0.0	97.1	2
2912	RF01800 gadd7	0.0 (0/239)	0.0	0.0 (0/0)	0.0	99.7	2
2913	RF01792 eiav FSE	0.0 (0/5)	0.0	0.0 (0/0)	0.0	92.6	5
2914	RF02630 Hrs12	0.0 (0/23)	0.0	0.0 (0/0)	0.1	88.5	2
2915	RF01114 TMV UPD-PK1	0.0 (0/7)	0.0	0.0 (0/0)	0.0	100.0	2
2916	RF01488 rli49	0.0 (0/31)	0.0	0.0 (0/0)	0.5	92.3	5
2917	RF02479 GlsR13	0.0 (0/0)	0.0	0.0 (0/0)	0.0	92.7	3
2918	RF01645 ceN58	0.0 (0/40)	0.0	0.0 (0/0)	0.3	88.5	3
2919	RF00946 mir-1225	0.0 (0/13)	0.0	0.0 (0/0)	0.2	94.5	7
2920	RF00879 mir-615	0.0 (0/28)	0.0	0.0 (0/0)	0.2	97.2	6
2921	RF01975 HOTAIRM1 1	0.0 (0/0)	0.0	0.0 (0/0)	0.0	88.7	14
2922	RF01406 STnc500	0.0 (0/67)	0.0	0.0 (0/0)	0.1	98.8	8
2923	RF02219 ZNRD1-AS1 2	0.0 (0/0)	0.0	0.0 (0/0)	0.0	77.2	34
2924	RF00382 DnaX	0.0 (0/11)	0.0	0.0 (0/0)	0.4	85.7	5
2925	RF00971 mir-578	0.0 (0/36)	0.0	0.0 (0/0)	0.2	95.6	3
2926	RF01592 plasmodium snoR17	0.0 (0/4)	0.0	0.0 (0/0)	0.2	86.9	5
2927	RF02875 AS-pc01	0.0 (0/28)	0.0	0.0 (0/0)	0.8	72.2	3
2928	RF02617 SSRC10	0.0 (0/34)	0.0	0.0 (0/0)	0.0	97.0	3
2929	RF02280 TtnuCD3	0.0 (0/0)	0.0	0.0 (0/0)	0.0	94.0	3

Continued on next page

	RNA family (seed alignment)	Sensitivity annotated bpairs that covary % (cov_bps/bps)	Power average power %	Positive Predictive Value covarying pairs in structure % (cov_bps/cov_pairs)	average substitutions per bpair	avg pairwise identity %	number of sequences
2930	RF00320 snoZ185	0.0 (0/6)	0.0	0.0 (0/0)	1.8	93.8	5
2931	RF02323 TtnuHACA16	0.0 (0/30)	0.0	0.0 (0/0)	0.0	94.9	2
2932	RF01334 CRISPR-DR21	0.0 (0/8)	0.0	0.0 (0/0)	0.0	97.3	2
2933	RF01274 sR45	0.0 (0/14)	0.0	0.0 (0/0)	0.6	82.1	7
2934	RF01193 snoR20a	0.0 (0/3)	0.0	0.0 (0/0)	0.0	69.2	5
2935	RF00371 sroE	0.0 (0/19)	0.0	0.0 (0/0)	0.8	83.6	4
2936	RF02549 YFV 3UTR	0.0 (0/24)	0.0	0.0 (0/0)	0.0	98.3	3
2937	RF02054 STnc420	0.0 (0/13)	0.0	0.0 (0/0)	0.2	95.4	3
2938	RF01977 HOTAIRM1 3	0.0 (0/0)	0.0	0.0 (0/1)	0.0	88.0	11
2939	RF01678 P37	0.0 (0/23)	0.0	0.0 (0/0)	0.0	95.2	2
2940	RF00068 SNORD21	0.0 (0/4)	0.0	0.0 (0/0)	2.2	71.5	5
2941	RF00352 snoR21	0.0 (0/6)	0.0	0.0 (0/0)	1.3	77.6	6
2942	RF00970 mir-648	0.0 (0/27)	0.0	0.0 (0/0)	0.0	92.5	2
2943	RF02186 ST7-OT3 4	0.0 (0/0)	0.0	0.0 (0/0)	0.0	87.3	8
2944	RF02668 PsiU2-55	0.0 (0/52)	0.0	0.0 (0/0)	0.2	96.6	3
2945	RF01313 AHBV epsilon	0.0 (0/16)	0.0	0.0 (0/0)	0.0	92.7	10
2946	RF02330 TtnuHACA23	0.0 (0/37)	0.0	0.0 (0/0)	0.2	72.9	2
2947	RF01812 Pxr	0.0 (0/37)	0.0	0.0 (0/0)	0.1	93.5	2
2948	RF02663 GmglnB1 5p UTR	0.0 (0/26)	0.0	0.0 (0/0)	0.0	98.8	2
2949	RF00993 mir-1473	0.0 (0/29)	0.0	0.0 (0/0)	0.1	76.9	2
2950	RF02329 TtnuHACA22	0.0 (0/30)	0.0	0.0 (0/0)	0.1	93.9	2
2951	RF01123 sR35	0.0 (0/0)	0.0	0.0 (0/0)	0.0	84.6	3
2952	RF01651 ceN69	0.0 (0/5)	0.0	0.0 (0/0)	0.4	91.6	4
2953	RF01530 CC3664	0.0 (0/43)	0.0	0.0 (0/0)	0.0	100.0	2
2954	RF00463 ApoB 5 CRE	0.0 (0/41)	0.0	0.0 (0/0)	0.8	90.9	5
2955	RF01928 Mico1	0.0 (0/0)	0.0	0.0 (0/0)	0.0	82.9	3
2956	RF00632 sxy	0.0 (0/33)	0.0	0.0 (0/0)	0.0	97.1	2
2957	RF02175 SMAD5-AS1 3	0.0 (0/0)	0.0	0.0 (0/0)	0.0	79.2	18
2958	RF01481 rli53	0.0 (0/53)	0.0	0.0 (0/0)	0.1	95.8	5
2959	RF01403 STnc290	0.0 (0/18)	0.0	0.0 (0/0)	0.3	99.0	6
2960	RF02664 IRES cyp24a1	0.0 (0/96)	0.0	0.0 (0/0)	0.0	100.0	2
2961	RF02729 JA01	0.0 (0/46)	0.0	0.0 (0/0)	0.0	99.6	3
2962	RF02127 GNAS-AS1 1	0.0 (0/0)	0.0	0.0 (0/0)	0.0	80.6	21
2963	RF01356 CRISPR-DR28	0.0 (0/7)	0.0	0.0 (0/0)	0.1	91.7	3
2964	RF02332 TtnuHACA25	0.0 (0/32)	0.0	0.0 (0/0)	0.0	97.3	2
2965	RF02619 BSnc115	0.0 (0/23)	0.0	0.0 (0/0)	0.1	97.1	3
2966	RF01301 snoR4a	0.0 (0/8)	0.0	0.0 (0/0)	0.0	97.4	2
2967	RF02749 Ncrwmel02	0.0 (0/78)	0.0	0.0 (0/0)	0.2	97.1	3
2968	RF00615 LhrA	0.0 (0/82)	0.0	0.0 (0/0)	0.1	99.0	10
2969	RF02156 NPPA-AS1 1	0.0 (0/0)	0.0	0.0 (0/0)	0.0	74.1	19
2970	RF02412 snoR144	0.0 (0/40)	0.0	0.0 (0/0)	0.4	87.7	4
2971	RF01386 isrB	0.0 (0/21)	0.0	0.0 (0/0)	0.5	83.9	3
2972	RF02121 FTX 3	0.0 (0/0)	0.0	0.0 (0/0)	0.0	74.6	14
2973	RF00092 SNORA63	0.0 (0/23)	0.0	0.0 (0/0)	0.9	80.7	8
2974	RF01872 MEG3 2	0.0 (0/0)	0.0	0.0 (0/0)	0.0	87.2	16
2975	RF02306 TtnuCD32	0.0 (0/0)	0.0	0.0 (0/0)	0.0	94.2	3
2976	RF01639 ceN49	0.0 (0/34)	0.0	0.0 (0/0)	0.4	88.6	3
2977	RF01522 TB10Cs1H1	0.0 (0/15)	0.0	0.0 (0/0)	0.9	84.6	4
2978	RF02483 GlsR20	0.0 (0/29)	0.0	0.0 (0/0)	0.3	86.9	3
2979	RF02190 ST7-OT4 4	0.0 (0/0)	0.0	0.0 (0/0)	0.0	77.0	26
2980	RF01293 ACA59	0.0 (0/32)	0.0	0.0 (0/0)	1.3	75.1	3
2981	RF02239 asX6	0.0 (0/31)	0.0	0.0 (0/0)	0.4	94.2	3
2982	RF01266 snR45	0.0 (0/0)	0.0	0.0 (0/1)	0.0	79.3	8
2983	RF02591 Spy1786666	0.0 (0/26)	0.0	0.0 (0/0)	0.0	97.7	2
2984	RF02196 TP53TG1 2	0.0 (0/0)	0.0	0.0 (0/0)	0.0	65.0	12
2985	RF01202 sn2991	0.0 (0/6)	0.0	0.0 (0/0)	0.3	86.7	4
2986	RF02760 sR035	0.0 (0/65)	0.0	0.0 (0/0)	0.2	98.0	3
2987	RF02608 AbsR11	0.0 (0/62)	0.0	0.0 (0/0)	0.0	95.5	2
2988	RF01909 CDKN2B-AS	0.0 (0/0)	0.0	0.0 (0/0)	0.0	79.9	25
2989	RF01780 AS1890	0.0 (0/28)	0.0	0.0 (0/0)	0.0	100.0	2
2990	RF02337 GlsR8 miR10	0.0 (0/20)	0.0	0.0 (0/0)	0.0	98.1	3
2991	RF02603 BASRCII26	0.0 (0/61)	0.0	0.0 (0/0)	0.0	100.0	2
2992	RF01588 snoR13	0.0 (0/4)	0.0	0.0 (0/0)	1.8	85.6	4
2993	RF00714 MIR535	0.0 (0/27)	0.0	0.0 (0/0)	1.0	65.9	4
2994	RF01416 NrrF	0.0 (0/33)	0.0	0.0 (0/0)	0.0	95.6	3
2995	RF02063 STnc350	0.0 (0/13)	0.0	0.0 (0/0)	0.0	85.1	2
2996	RF00441 snoZ242	0.0 (0/19)	0.0	0.0 (0/0)	1.0	88.2	5
2997	RF00500 TCV H5	0.0 (0/12)	0.0	0.0 (0/0)	0.7	97.1	5
2998	RF01877 MIAT exon5 3	0.0 (0/0)	0.0	0.0 (0/0)	0.0	84.6	16
2999	RF02866 ncS16	0.0 (0/19)	0.0	0.0 (0/0)	0.7	85.7	4
3000	RF02769 Ysr202	0.0 (0/86)	0.0	0.0 (0/0)	0.1	94.6	2
3001	RF00896 mir-787	0.0 (0/31)	0.0	0.0 (0/0)	0.4	63.3	2
3002	RF02021 mir-3179	0.0 (0/37)	0.0	0.0 (0/0)	0.0	100.0	3
3003	RF01143 sR16	0.0 (0/0)	0.0	0.0 (0/0)	0.0	100.0	2
3004	RF02525 SSRC30	0.0 (0/40)	0.0	0.0 (0/0)	0.1	98.4	3
3005	RF02720 sca ncR21	0.0 (0/26)	0.0	0.0 (0/0)	0.2	84.3	2
3006	RF01383 GRIK4 3p UTR	0.0 (0/9)	0.0	0.0 (0/0)	0.2	92.4	8
3007	RF02069 STnc70	0.0 (0/37)	0.0	0.0 (0/0)	0.0	93.3	2
3008	RF01064 mir-253	0.0 (0/37)	0.0	0.0 (0/0)	0.8	79.2	4
3009	RF01195 snR52	0.0 (0/0)	0.0	0.0 (0/0)	0.0	96.9	5
3010	RF02704 LcrF thermometer	0.0 (0/39)	0.0	0.0 (0/0)	0.0	100.0	2
3011	RF01090 RF site4	0.0 (0/18)	0.0	0.0 (0/0)	0.8	86.5	7
3012	RF01918 mir-1249	0.0 (0/29)	0.0	0.0 (0/0)	0.0	97.5	4
3013	RF00626 Gurken	0.0 (0/24)	0.0	0.0 (0/0)	0.1	98.1	3

Continued on next page

	RNA family (seed alignment)	**Sensitivity** annotated bpairs that covary % (cov_bps/bps)	**Power** average power %	**Positive Predictive Value** covarying pairs in structure % (cov_bps/cov_pairs)	average substitutions per bpair	avg pairwise identity %	number of sequences
3014	RF01025 mir-934	0.0 (0/36)	0.0	0.0 (0/0)	0.1	94.0	2
3015	RF01194 sn2903	0.0 (0/4)	0.0	0.0 (0/0)	0.0	98.7	4
3016	RF01289 snoR17	0.0 (0/10)	0.0	0.0 (0/0)	0.2	70.4	2
