# Supplementary material for: Estimating the power of sequence covariation for detecting conserved RNA structure
Source: Bioinformatics. 2020 Feb 7;36(10):3072–6. doi: 10.1093/bioinformatics/btaa080 (PMC7214042; doi:10.1093/bioinformatics/btaa080)
Supplement: btaa080_Supplementary_Data [file btaa080_supplementary_data.zip › btaa080-Suppl_Data/Tables/Table_S2.pdf]

	**RNA family** (seed alignment)	observed bps in structure	power %	expected bps to covary	average substitutions per bpair	avg pairwise identity %	number of sequences
1032	RF01419 IsrR	11	77.3	9	157.2	66.9	308
1033	RF01942 mir-1937	11	66.4	7	84.6	65.8	171
1034	RF00523 Prion pknot	6	50.0	3	53.0	85.0	148
1035	RF00485 K chan RES	24	41.2	10	45.1	68.7	85
1036	RF00468 HCV SLVII	17	38.8	7	42.9	74.3	110
1037	RF00693 mir-147	15	29.3	4	28.5	68.5	64
1038	RF00480 HIV FE	10	27.0	3	32.2	84.0	145
1039	RF00093 SNORD18	4	22.5	1	21.5	69.9	16
1040	RF00376 HIV GSL3	8	21.2	2	22.0	81.4	72
1041	RF01753 psbNH	12	20.8	3	21.4	76.6	39
1042	RF00469 HCV SLIV	15	20.7	3	21.3	86.3	110
1043	RF00047 mir-2	21	20.0	4	20.4	66.6	56
1044	RF00550 HepE CRE	41	17.1	7	16.7	84.2	46
1045	RF00535 snoMe28S-Am982	3	16.7	1	16.3	76.2	13
1046	RF00104 mir-10	24	16.7	4	16.6	68.1	36
1047	RF00736 mir-320	19	16.3	3	17.1	68.0	55
1048	RF00654 mir-216	18	16.1	3	15.8	61.5	33
1049	RF00134 snoZ196	7	15.7	1	16.7	68.4	22
1050	RF00490 S-element	22	15.4	3	15.7	75.8	29
1051	RF02027 MIR2907	16	15.0	2	15.6	76.9	52
1052	RF02002 mir-720	26	15.0	4	15.1	81.2	35
1053	RF02510 PYLIS 3	8	15.0	1	16.5	63.0	23
1054	RF00651 mir-221	21	14.8	3	14.7	73.9	47
1055	RF00041 Entero OriR	35	14.0	5	14.1	88.0	60
1056	RF01803 GABA3	21	13.8	3	14.0	84.5	52
1057	RF01518 pRNA	22	13.6	3	14.0	57.2	23
1058	RF00665 mir-290	25	13.6	3	13.7	65.9	27
1059	RF00424 SCARNA16	54	13.0	7	14.3	75.9	37
1060	RF00451 mir-395	30	12.7	4	12.9	65.0	25
1061	RF00679 mir-210	27	12.6	3	12.4	61.6	26
1062	RF00670 mir-105	29	12.4	4	13.3	67.3	20
1063	RF00357 snoR44 J54	5	12.0	1	11.2	72.1	29
1064	RF02447 SpR19 sRNA	30	12.0	4	13.0	74.8	23
1065	RF01982 PYLIS 1	15	11.3	2	10.9	71.7	20
1066	RF00639 mir-515	19	11.1	2	12.4	80.2	40
1067	RF02516 mir-393	29	11.0	3	10.9	63.8	27
1068	RF02031 tpke11	16	10.6	2	10.3	68.9	28
1069	RF00691 mir-146	17	10.6	2	10.4	63.1	33
1070	RF00034 RprA	18	10.6	2	10.4	66.8	13
1071	RF00446 mir-133	20	10.5	2	10.7	67.6	46

Table S2: **Rfam RNA families with sufficient power but no covariations.** List of 40 Rfam (v14.1) RNA families with more than 10% power but no covariations, ranked by decreasing power. The expected number of basepair to covary is calculated using R-scape with E-value < 0.05.
