# Supplementary material for: Estimating the power of sequence covariation for detecting conserved RNA structure
Source: Bioinformatics. 2020 Feb 7;36(10):3072–6. doi: 10.1093/bioinformatics/btaa080 (PMC7214042; doi:10.1093/bioinformatics/btaa080)
Supplement: btaa080_Supplementary_Data [file btaa080_supplementary_data.zip › btaa080-Suppl_Data/Tables/Table_S3.pdf]

Structural Alignment	# of seqs	avg % identity	avg seq length	proposed pairs	expected cov pairs	observed cov pairs
**HOTAIR RNA** [9]						
HOTAIR_Pyle15 D1	37	74	450	149	**58.4**	0
HOTAIR_Pyle15 D2	31	74	505	134	**23.0**	0
HOTAIR_Pyle15 D3	34	68	407	125	**41.4**	0
HOTAIR_Pyle15 D4	31	69	601	165	**38.6**	0
**Steroid Receptor Activator RNA** [10]						
SRA_Sanbonmatsu12	76	78	764	234	**49.7**	0
**Xist RNA - Repeat A**						
Xist_Branlant12 XIST_A.S0 [11]	10	81	438	53	0.0	0
Xist_Branlant12 XIST_A.S1 [11]	10	81	439	90	0.5	0
Xist_Branlant12 XIST_A.S2 [11]	10	81	439	72	0.4	0
Xist_Branlant12 XIST_A.S3 [11]	10	81	439	83	0.4	0
Xist_Simon15 Fig.5 [12]	13	75	423	99	2.5	0
**Xist RepA lncRNA - Repeat A + Repeat F**						
Xist_Pyle17 [18]	57	68	1,320	334	**108.6**	1
Xist_Rivas19 h1	65	68	1,076	254	**100.7**	0
**Xist RNA - other conserved regions**						
Xist_Rivas19 h2 (exon1, repC frag)	52	68	197	41	**25.1**	0
Xist_Rivas19 h3 (exon1)	55	68	257	53	**16.2**	0
Xist_Rivas19 h4 (exon1)	61	60	254	64	**28.6**	0
Xist_Rivas19 h5 (exon3)	55	70	275	63	**18.4**	0
Xist_Rivas19 h6 (exon4)	56	79	360	112	**20.7**	0
Xist_Rivas19 h7 (exon5)	73	61	354	123	**50.4**	0
Xist_Rivas19 h8 (exon6, repE frag)	60	66	323	89	**34.3**	0
Xist_Rivas19 h9 (exon6)	47	70	129	32	**7.2**	0
Xist_Rivas19 h10 (exon6)	59	73	262	61	**15.9**	0
Xist_Rivas19 h1-h10 (concatenated)	32	72	3528	980	**141.3**	0
**COOLAIR antisense RNA**						
COOLAIR_Sanbonmatsu16 [21]	6	78	394	114	0.9	6
COOLAIR_Rivas19	6	80	547	166	0.8	1

Table S3: **Power of covariation versus observed covariations for several lncRNAs.** No observed covariations for an adequate expected number of them constitutes negative evidence for a conserved RNA structure. Expected covariations larger than 10% the number of proposed basepairs representing alignments with sufficient power are displayed in bold. The proposed pairs are the annotated basepairs in the source alignments. The label "Rivas19" indicates alignments produced in this work using nhmmer[19] (for Xist) or Infernal[22] (for COOLAIR). The structures for "Rivas19" alignments are produced with R-scape. The expected and observed covarying pairs were obtained with R-scape v1.2.3 using default parameters (E-value < 0.05). The expected number of covarying pairs is the sum of the power of the basepairs in the proposed structures (Methods, Eq. 2). The average pairwise percentage identity is calculated after removing positions with more than 50% gaps. All alignments are included in the supplemental materials.
