# Supplementary material for: Estimating the power of sequence covariation for detecting conserved RNA structure
Source: Bioinformatics. 2020 Feb 7;36(10):3072–6. doi: 10.1093/bioinformatics/btaa080 (PMC7214042; doi:10.1093/bioinformatics/btaa080)
Supplement: btaa080_Supplementary_Data [file btaa080_supplementary_data.zip › btaa080-Suppl_Data/src/rscape_v1.2.3.tar.gz!/rscape_v1.2.3/share/man/R-scape_userguide.pdf]

R-scape User's Guide

RNA Significant Covariation Above Phylogenetic Expectation

http://eddylab.org/R-scape/
Version v1.2.3; February 2019

Elena Rivas
elenarivas@fas.harvard.edu
Department of Molecular and Celullar Biology
Harvard University
16 Divinity Avenue
Cambridge MA 02138 USA
http://eddylab.org/

Copyright (C) 2016 Howard Hughes Medical Institute.

Permission is granted to make and distribute verbatim copies of this manual provided the copyright notice and this permission notice are retained on all copies.

R-scape is licensed and freely distributed under the GNU General Public License version 3 (GPLv3). For a copy of the License, see `http://www.gnu.org/licenses/`.

Contents

1 Introduction **4**
How to avoid reading this manual 4
How do I cite R-scape? 4

2 Installation **5**
Quick installation instructions 5
System requirements 5
Makefile targets 6
Why is the output of 'make' so clean? 6
What gets installed by 'make install', and where? 6

3 Tutorial **7**
Modes of R-scape 7
Option –cyk 7
Files used in the tutorial 7
Running R-scape on one alignment file 8
The –cyk option 9
Example of an RNA with pseudoknots 12
Single sequence structure prediction 13
Default parameters 13

4 Outputs **15**
Tabular output per input file 15
Other tabular outputs 16
Outputs per alignment 16
Default 16
Details about outputs per alignment 17
Using option `R-scape --cyk` 17
Graphical outputs per alignment 19

5 Options **22**
Covariation statistic options 22
`-E <x>` 22
`--GT, --MI, --MIr, --MIg, --CHI, --OMES, --RAF, --RAFS,` 22
`--C2, --C16` 23
Search options 23
`-s` 23
`--cyk` 23
`--naive` 23
`--tstart <n>` 23
`--tend <n>` 23
`--window <n>` 23
`--slide <n>` 23
`--vshuffle` 23
`--cshuffle` 23
Input alignment options 24
`-I <x>` 24
`--gapthresh <x>` 24
`--consensus` 24
`--submsa <n>` 24

`--treefile <f>` . . . 24
Options for importing a structure . . . 24
`--pdbfile <s>` . . . 25
`--cntmaxD <x>` . . . 25
`--cntmind <n>` . . . 25
`--onlypdb` . . . 25
Options for type of pairs tested . . . 26
`--samplecontacts` . . . 26
`--samplebp` . . . 26
`--samplewc` . . . 26
Output options . . . 27
`--roc` . . . 27
`--outmsa <f>` . . . 27
`--outtree <f>` . . . 27
Plotting options . . . 27
`--nofigures` . . . 27
`--r2rall` . . . 27
Other options . . . 27
`--seed <n>` . . . 27

6 Some other topics **28**
How do I cite R-scape? . . . 28
How do I report a bug? . . . 28

7 Acknowledgments **29**

1 Introduction

R-scape (RNA Significant Covariation Above Phylogenetic Expectation) is a program that given a multiple sequence alignment (MSA) of RNA sequences, finds the pairs of positions that show a pattern of significant covariation. Each covariation score has an E-value associated to it. E-values are determined using a null model of covariation due to phylogeny but independent of any structural constraints.

How to avoid reading this manual

- Follow the quick installation instructions on page 5.
- Go to the tutorial section on page 7, which walks you through some examples of using R-scape on real data.

Everything else, you can read later.

How do I cite R-scape?

Rivas, E. *et al.*, *"A statistical test for conserved RNA structure shows lack of evidence for structure in lncRNAs"*, Nature Methods 14, 4548 (2017).

2 Installation

Quick installation instructions

Download `R-scape.tar.gz` from `http://eddylab.org/`; unpack it, configure, and make:

```
> tar xf R-scape.tar.gz
> cd R-scape
> ./configure
> make
> make install
```

The newly compiled binary (`R-scape`) is in the `R-scape/bin` directory. You can run it from there, as in this example:

```
> bin/R-scape tutorial/updated_Arisong.sto
```

That's it. You can keep reading if you want to know more about customizing a R-scape installation, or you can skip ahead to the next chapter, the tutorial.

System requirements

Operating system: R-scape is designed to run on POSIX-compatible platforms, including UNIX, Linux and Mac OS/X. The POSIX standard essentially includes all operating systems except Microsoft Windows. We have tested most extensively on Linux and MacOS/X because these are the machines we develop on.

Compiler: The source code is C conforming to POSIX and ANSI C99 standards. It should compile with any ANSI C99 compliant compiler, including the GNU C compiler `gcc`, and the C++ compiler `g++`. We test the code using the `gcc` and `g++` compilers.

Libraries and other installation requirements: R-scape includes two software libraries:

- the Easel library package (`http://bioeasel.org/`),
- the HMMER library package (`http://hmmer.org/`),

and three independent programs:

- FastTree (Price et al., 2010) (for building phylogenetic trees),
- R2R (Weinberg and Breaker, 2011) (for drawing consensus RNA structures),
- RNAVIEW (Yang et al., 2003) (for identifying different types of basepairs in nucleic acid alignments).

All libraries and independent programs will automatically compile during R-scape's installation process. By default, R-scape does not require any additional libraries to be installed by you, other than standard ANSI C99 libraries that should already be present on a system that can compile C code.

Executables for the three independent programs will appear in the `R-scape/bin` directory.

Makefile targets

all Builds everything. Same as just saying `make`.

install Installs the binaries (`R-scape`, `FastTree`, `r2r`).

By default, programs are installed in `R-scape_version/bin`. You can customize the location of the binaries by replacing

> ./configure

with

> ./configure --prefix=/the/directory/you/want

The newly compiled binaries are now in the `/the/directory/you/want/bin` directory.

uninstall Reverses the steps of make install.

clean Removes all files generated by compilation (by `make`). Configuration (files generated by `./configure`) is preserved.

distclean Removes all files generated by configuration (by `./configure`) and by compilation (by `make`).

Why is the output of 'make' so clean?

Because we're hiding what's really going on with the compilation with a wrapper. If you want to see what the command lines really look like, pass a `V=1` option (V for "verbose") to `make`, as in:

> make V=1

What gets installed by 'make install', and where?

The top-level configure file has a variable RSCAPE_HOME that specifies the directory where `make install` will install things: `RSCAPE_HOME/bin`.

By default RSCAPE_HOME is assigned to the current directory R-scape.

The best way to change this default is when you use `./configure`, and the most important variable to consider changing is `--prefix`. For example, if you want to install R-scape in a directory hierarchy all of its own, you might want to do something like:

> ./configure --prefix=/usr/local/rscape

That would keep R-scape out of your system-wide directories like `/usr/local/bin`, which might be desirable. Of course, if you do it that way, you'd also want to add `/usr/local/rscape/bin` to your `$PATH`.

3 Tutorial

Here's a tutorial walk-through of how to use R-scape. This should suffice to get you started.

Modes of R-scape

For an input alignment, R-scape reports all pairs that have covariation scores with E-values smaller than a target E-value.

The E-values are calculated in one of two ways:

A one-set statistical test: *default*

E-values are calculated assuming that all pairs are possible.
This is the default behavior of R-scape.

A two-set statistical test: `option -s`

If the alignment has associated a *given structure*, **`option -s`** performs two independent statistical tests:
one for the pairs included in the structure, a different one for all the remaining possible pairs.
It also draws the given consensus structure annotated with the significantly covarying base pairs.

Option –cyk

After performing one of the two statistical tests, this option:

Builds the best consensus structure that includes the largest possible number of significantly covarying pairs, *the maximum-covariation optimal consensus structure*. The algorithm identifies pseudoknots and other not nested interactions by running a cascade of cyk nested algorithms until all covarying pairs are taken into account.

Draws the *maximum-covariation optimal consensus structure* annotated with the significantly covarying base pairs.

It also returns the alignment in Stockholm format annotated with the max-cov optimal consensus structure.

I'll show examples of running each mode, using examples in the `tutorial/` subdirectory of the distribution.

Files used in the tutorial

The subdirectory `/tutorial` in the R-scape distribution contains the files used in the tutorial.
The tutorial provides several examples of RNA structural alignments, all in Stockholm format:

`updated_Arisong.sto` Structural alignment of the ciliate Arisong RNA. This alignment is an updated version of the one published in (Jung et al., 2011).

`ar14.sto` Structural alignment of the α-proteobacteria ncRNA ar14. This alignment is an updated version of the one published in (del Val et al., 2012).

`manA.sto` Alignment of the manA RNA motif (Weinberg et al., 2009, 2010) provided in the Zasha Weinberg database (ZWD) (Weinberg, 2018).

`RF00005.sto` Rfam v12.0 (Nawrocki et al., 2015) seed alignment of tRNA.

`RF00001-noss.sto` Rfam v12.0 seed alignment of 5S rRNA, after removing the consensus secondary structure.

Running R-scape on one alignment file

To run R-scape with default parameters on alignment file `tutorial/updated_Arisong.sto` use:

> bin/R-scape tutorial/updated_Arisong.sto

The output is a list of the significantly covarying positions under the one-set test

```
# R-scape :: RNA Structural Covariation Above Phylogenetic Expectation
# R-scape 1.2.0 (January 2019)
# Copyright (C) 2016-2019 Howard Hughes Medical Institute.
# Freely distributed under the GNU General Public License (GPLv3).
# - - - - - - - - - - - - - - - - - - - - - - - - - - - - - - - - - - - -
# One-set statistical test (all pairs are tested as equivalent)
#
# MSA updated_Arisong_1 nseq 95 (95) alen 65 (150) avgid 66.35 (64.97) nbpairs 20 (20)
#
# Method Target_E-val [cov_min,conv_max] [FP | TP True Found | Sen PPV F]
# GTp    0.05         [-9.82,121.80]     [0 | 4 20 4 | 20.00 100.00 33.33]
#
#       left_pos       right_pos        score          E-value       substitutions      power
#-------------------------------------------------------------------------------------------------------
*            98             106       121.80433        3.95915e-08        44                0.44
*           122             137       91.75573        8.34366e-05        59                0.55
*            96             108       89.46430        0.000148586        25                0.26
*           120             139       75.03790        0.00537656        85                0.70
```

A star "*" in the first column indicates that the pair is part of the annotated structure in the `updated_Arisong.sto` file. A blank indicates a pair that is not compatible with the structure. A "∼" indicates an interaction not in the annotated structure but compatible with it (none in this example).

The Arisong RNA in `tutorial/updated_Arisong.sto` has a proposed secondary structure. Instead of testing all pairs as equivalent, we may want to test the significance of the given structure as a one set of pairs, and independently that of the rest of all possible pairs. In order to do a two-set test use:

> bin/R-scape -s tutorial/updated_Arisong.sto

The output is a list of the significantly covarying positions under the two-set test.

```
# R-scape :: RNA Structural Covariation Above Phylogenetic Expectation
# R-scape 1.2.0 (January 2019)
# Copyright (C) 2016-2019 Howard Hughes Medical Institute.
# Freely distributed under the GNU General Public License (GPLv3).
# - - - - - - - - - - - - - - - - - - - - - - - - - - - - - - - - - - - -
# Two-set statistical test (one test for annotated basepairs, another for all other pairs)
#
# Structure obtained from the msa
# SS_cons ::::::::::::::::::::::::::::::::::::::::::::::::::::::::::::
#
# SS_cons :::::::::::::::::::::::::::::::::<<<<<<<___>>>>>>><<<<<<--<<
#
# SS_cons <<<<<_______>>>->>>>-->>>>>>::
#
# left_pos      right_pos    substitutions      power
#-------------------------------------------------------
# 94              110              34               0.35
# 95              109              29               0.30
# 96              108              25               0.26
# 97              107              58               0.55
# 98              106              44               0.44
# 99              105              15               0.14
# 100              104              20               0.20
# 111              148              0               0.00
# 112              147              18               0.18
# 113              146              1               0.00
# 114              145              9               0.07
# 115              144              48               0.47
# 116              143              110               0.80
# 119              140              88               0.72
# 120              139              85               0.70
```

```
# 121              138              97               0.76
# 122              137              59               0.55
# 123              135              73               0.64
# 124              134              27               0.28
# 125              133              31               0.32
#
# BPAIRS 20
# avg substitutions per BP  43.5
# BPAIRS expected to covary 7.7
# BPAIRS observed to covary 12
#
#
# Method Target_E-val [cov_min,cov_max] [FP | TP True Found | Sen PPV F]
# GTp    0.05         [-9.82,121.80]     [0 | 12 20 12 | 60.00 100.00 75.00]
#
#       left_pos       right_pos        score          E-value       substitutions      power
#-------------------------------------------------------------------------------------------------------
*            98             106       121.80433       3.80688e-10        44               0.44
*           122             137       91.75573       8.02275e-07        59               0.55
*            96             108       89.46430       1.42871e-06        25               0.26
*           120             139       75.03790       5.16977e-05        85               0.70
*           119             140       58.25176       0.00255411        88               0.72
*           121             138       57.96915       0.00265136        97               0.76
*            94             110       56.91065       0.00330664        34               0.35
*           124             134       55.84207       0.00409643        27               0.28
*           123             135       55.50367       0.00439184        73               0.64
*            99             105       53.86423       0.00611505        15               0.14
*            97             107       44.72409       0.0293269        58               0.55
*           115             144       41.87792       0.0490385        48               0.47
```

The scores of the pairs are identical to those in the one-set test. The E-values have changed relative to those of the one-set test.

The –cyk option

> bin/R-scape --cyk tutorial/updated_Arisong.sto

The output includes first the same output as default R-scape alone, followed by R-scape's proposed structure that under the heading "# The predicted cyk-cov structure" as follows,

```
# R-scape :: RNA Structural Covariation Above Phylogenetic Expectation
# R-scape 1.2.0 (January 2019)
# Copyright (C) 2016 Howard Hughes Medical Institute.
# Freely distributed under the GNU General Public License (GPLv3).
#-------------------------------------------------------------------------------------------------------
# MSA updated_Arisong_1 nseq 95 (95) alen 65 (150) avgid 66.35 (64.97) nbpairs 20 (20)
# One-set statistical test (all pairs are tested as equivalent)
#
#
# Method Target_E-val [cov_min,cov_max] [FP | TP True Found | Sen PPV F]
# GTp    0.05         [-9.82,121.80]     [0 | 4 20 4 | 20.00 100.00 33.33]
#
#       left_pos       right_pos        score          E-value       substitutions      power
#-------------------------------------------------------------------------------------------------------
*            98             106       121.80433       3.95915e-08        44               0.47
*           122             137       91.75573       8.34366e-05        59               0.60
*            96             108       89.46430       0.000148586        25               0.27
*           120             139       75.03790       0.00537656        85               0.75

# The predicted cyk-cov structure
# SS_cons ::::::::::::::::::::::::::::::::::::::::::::::::::::::::::::
#
# SS_cons :::::::::::::::::::::::::::::::::<<<<<_____>>>>>:<<<<<---<<
#
# SS_cons <<<<<_______>>>->>>>--->>>>>::
#
# left_pos      right_pos    substitutions      power
#-------------------------------------------------------
# 95              109              29               0.32
# 96              108              25               0.27
# 97              107              58               0.59
# 98              106              44               0.47
```

```
# 99              105              15              0.14
# 111              148              0              0.00
# 112              147              18              0.18
# 113              146              1              0.00
# 114              145              9              0.06
# 115              144              48              0.51
# 119              140              88              0.77
# 120              139              85              0.75
# 121              138              97              0.81
# 122              137              59              0.60
# 123              135              73              0.69
# 124              134              27              0.30
# 125              133              31              0.34
#
# BPAIRS 17
# avg substitutions per BP  41.6
# BPAIRS expected to covary 6.8
# BPAIRS observed to covary 4
#
#
# Method Target_E-val [cov_min,cov_max] [FP | TP True Found | Sen PPV F]
# GTp    0.05         [-9.82,121.80]     [0 | 4 17 4 | 23.53 100.00 38.10]
#
# in_cyk in_given   left_pos       right_pos      score          E-value    substitutions      power
#------------------------------------------------------------------------------------------------------------
*        *              98              106        121.80433        3.95915e-08        44              0.47
*        *             122              137        91.75573        8.34366e-05        59              0.60
*        *              96              108        89.46430        0.000148586        25              0.27
*        *             120              139        75.03790        0.00537656        85              0.75
```

The structure predicted by R-scape includes all the basepairs reported as covarying, provided that those can be arranged into one single structure (including pseudoknots and other non Watson-Crick interactions). The R-scape folding algorithm cannot deal with residues that covary with more than one other residue, such as is the case for alternative structures or triplets.
Similarly using

> bin/R-scape -s --cyk tutorial/updated_Arisong.sto

The output includes first the same output as **option -s** of R-scape alone, followed by R-scape's proposed structure including a maximal number of the covariations obtained under the two-set test.

```
# R-scape :: RNA Structural Covariation Above Phylogenetic Expectation
# R-scape 1.2.0 (January 2019)
# Copyright (C) 2016 Howard Hughes Medical Institute.
# Freely distributed under the GNU General Public License (GPLv3).
#-------------------------------------------------------------------------------------------------------
# MSA updated_Arisong_1 nseq 95 (95) alen 65 (150) avgid 66.35 (64.97) nbpairs 20 (20)
# Two-set statistical test (one test for annotated basepairs, another for all other pairs)
#
# Structure obtained from the msa
# SS_cons ::::::::::::::::::::::::::::::::::::::::::::::::::::::::::
#
# SS_cons :::::::::::::::::::::::::::::::::<<<<<<<___>>>>>>><<<<<<--<<
#
# SS_cons <<<<<_______>>>->>>>-->>>>>>::
#
# left_pos      right_pos    substitutions      power
#-------------------------------------------------------
# 94              110              34              0.37
# 95              109              29              0.32
# 96              108              25              0.27
# 97              107              58              0.59
# 98              106              44              0.47
# 99              105              15              0.14
# 100              104              20              0.21
# 111              148              0              0.00
# 112              147              18              0.18
# 113              146              1              0.00
# 114              145              9              0.06
# 115              144              48              0.51
# 116              143              110              0.86
# 119              140              88              0.77
# 120              139              85              0.75
```

```
# 121              138              97              0.81
# 122              137              59              0.60
# 123              135              73              0.69
# 124              134              27              0.30
# 125              133              31              0.34
#
# BPAIRS 20
# avg substitutions per BP  43.5
# BPAIRS expected to covary 8.2
# BPAIRS observed to covary 12
#
#
# Method Target_E-val [cov_min,cov_max] [FP | TP True Found | Sen PPV F]
# GTp    0.05         [-9.82,121.80]     [0 | 12 20 12 | 60.00 100.00 75.00]
#
#       left_pos       right_pos        score          E-value       substitutions      power
#-------------------------------------------------------------------------------------------------------
*             98             106       121.80433       3.80688e-10       44                0.47
*            122             137       91.75573       8.02275e-07       59                0.60
*             96             108       89.46430       1.42871e-06       25                0.27
*            120             139       75.03790       5.16977e-05       85                0.75
*            119             140       58.25176       0.00255411       88                0.77
*            121             138       57.96915       0.00265136       97                0.81
*             94             110       56.91065       0.00330664       34                0.37
*            124             134       55.84207       0.00409643       27                0.30
*            123             135       55.50367       0.00439184       73                0.69
*             99             105       53.86423       0.00611505       15                0.14
*             97             107       44.72409       0.0293269       58                0.59
*            115             144       41.87792       0.0490385       48                0.51

# The predicted cyk-cov structure
# SS_cons ::::::::::::::::::::::::::::::::::::::::::::::::::::::::::::
#
# SS_cons :::::::::::::::::::::::::::::::::<<<<<<_____>>>>>><<<<<---<<
#
# SS_cons <<<<<_______>>>->>>>--->>>>>::
#
# left_pos      right_pos    substitutions      power
#---------------------------------------------------------
# 94              110              34              0.37
# 95              109              29              0.32
# 96              108              25              0.27
# 97              107              58              0.59
# 98              106              44              0.47
# 99              105              15              0.14
# 111              148              0              0.00
# 112              147              18              0.18
# 113              146              1              0.00
# 114              145              9              0.06
# 115              144              48              0.51
# 119              140              88              0.77
# 120              139              85              0.75
# 121              138              97              0.81
# 122              137              59              0.60
# 123              135              73              0.69
# 124              134              27              0.30
# 125              133              31              0.34
#
# BPAIRS 18
# avg substitutions per BP  41.2
# BPAIRS expected to covary 7.2
# BPAIRS observed to covary 12
#
#
# Method Target_E-val [cov_min,cov_max] [FP | TP True Found | Sen PPV F]
# GTp    0.05         [-9.82,121.80]     [0 | 12 18 12 | 66.67 100.00 80.00]
#
# in_cyk in_given   left_pos       right_pos      score          E-value    substitutions      power
#----------------------------------------------------------------------------------------------------------------
*        *              98              106       121.80433       3.80688e-10       44                0.47
*        *             122              137       91.75573       8.02275e-07       59                0.60
*        *              96              108       89.46430       1.42871e-06       25                0.27
*        *             120              139       75.03790       5.16977e-05       85                0.75
*        *             119              140       58.25176       0.00255411       88                0.77
*        *             121              138       57.96915       0.00265136       97                0.81
*        *              94              110       56.91065       0.00330664       34                0.37
*        *             124              134       55.84207       0.00409643       27                0.30
```

```
*      *          123          135      55.50367      0.00439184      73           0.69
*      *           99          105      53.86423      0.00611505      15           0.14
*      *           97          107      44.72409      0.0293269      58           0.59
*      *          115          144      41.87792      0.0490385      48           0.51
```

Example of an RNA with pseudoknots

After version 1.2.0, R-scape annotates pseudoknots. R-scape's folding algorithm is capable of predicting pseudoknots using a cascade of dynamic programming algorithms. In addition, if the structure given with the input alignment includes pseudoknots, R-scape uses the program R2R to automatically include the pseudoknots in the display of the consensus structure.

Consider the manA RNA motif. Both the proposed structure for manA RNA and the predicted R-scape structure have 2 pseudoknots with covariation support:

> bin/R-scape -s --cyk tutorial/manA.sto

```
# R-scape :: RNA Structural Covariation Above Phylogenetic Expectation
# R-scape 1.2.0 (January 2019)
# Copyright (C) 2016-2019 Howard Hughes Medical Institute.
# Freely distributed under the GNU General Public License (GPLv3).
#-------------------------------------------------------------------------------------------------------
# MSA manA nseq 193 (193) alen 189 (350) avgid 74.06 (71.38) nbpairs 62 (93)
# Two-set statistical test (one test for annotated basepairs, another for all other pairs)
#
# Structure obtained from the msa
# SS_cons   <<<<<__________________________________>>>>>::::[[[[[,,,<<--
# SS_cons_1 ::::::::::::::::::::::::::::::::::::::::::::::::::::::::::::
# SS_cons_2 ::::::::::::::::::::::::::::::::::::::::::::::::::::::::::::
#
# SS_cons   -----------<<<<<<__________________>>>>>>---->>,,,,((-((((,,
# SS_cons_1 ::::::::::::::::::::::::::::::::::::::::::::::::::::::::::::
# SS_cons_2 ::::::::::::::::::::::::::::::::::::::::::::::::::::::::::::
#
# SS_cons   ,,,,,,,,,,,,,,,,,,,,,,,,,,,,,<<<_______>>>,<<<<<<___________
# SS_cons_1 ::::::::::::::::::::::::::::::::::<<<<______________________
# SS_cons_2 ::::::::::::::::::::::::::::::::::::::::::::::::::::::::::::
#
# SS_cons   ____________________>>>>>>,,,<<<<<____________>>>>>,,))))--)
# SS_cons_1 _______________________________________>>>>:::::::::::::::::
# SS_cons_2 ::::::::::::::::::::::::::::::::::::::::::::::::::::::::::::
#
# SS_cons   ),,,,<<<<<<<------------<<<<____________________________>>>>
# SS_cons_1 ::::::::::::::::::::::::::::::::::::::::::::::::::::::::::::
# SS_cons_2 :::::::::::::::::::::::::::::::::<<<-<______________________
#
# SS_cons   ------->>>>>>>,,<<<<<_____________>>>>>]]]]]::::::
# SS_cons_1 ::::::::::::::::::::::::::::::::::::::::::::::::::
# SS_cons_2 _________________________>>>>:::::::::::::::::::::
#

  ...

  ...

# The predicted cyk-cov structure
# SS_cons   <<<<<_________________________________>>>>>:[---[[[[[,,,<<--
# SS_cons_1 ::::::::::::::::::::::::::::::::::::::::::::::::::::::::::::
# SS_cons_2 ::::::::::::::::::::::::::::::::::::::::::::::::::::::::::::
#
# SS_cons   -----------<<<<<<__________________>>>>>>---->>,(--((-((((,,
# SS_cons_1 ::::::::::::::::::::::::::::::::::::::::::::::::::::::::::::
# SS_cons_2 ::::::::::::::::::::::::::::::::::::::::::::::::::::::::::::
#
# SS_cons   ,,,,,,,,,,,,,,,,,,,,,,,,,,,,,<<<_______>>>,<<<<<<___________
# SS_cons_1 ::::::::::::::::::::::::::::::::::<<<<______________________
# SS_cons_2 ::::::::::::::::::::::::::::::::::::::::::::::::::::::::::::
#
# SS_cons   ____________________>>>>>>,,,<<<<<---<____>--->>>>>,,))))--)
# SS_cons_1 _______________________________________>>>>:::::::::::::::::
# SS_cons_2 ::::::::::::::::::::::::::::::::::::::::::::::::::::::::::::
#
```

```
# SS_cons    )--),<<<<<<<------------<<<<______________________________>>>>
# SS_cons_1  :::::::::::::::::::::::::::::::::::::::::::::::::::::::::::::::
# SS_cons_2  ::::::::::::::::::::::::::::::::::<<<-<<______________________
#
# SS_cons    -------->>>>>>>,,<<<<<______________>>>>>]]]]]-]::::
# SS_cons_1  ::::::::::::::::::::::::::::::::::::::::::::::::::::
# SS_cons_2  _________________________>->>>>::::::::::::::::::::
#
```

The “SS_cons_1” and “SS_cons_2” lines describe the interactions that are not nested relative to the main “SS_cons” structure.

R-scape uses R2R to produce figures of the consensus structures where pseudoknots are also annotated. R-scape] option -s produces the file **tutorial/manA.R2R.sto.**{**pdf,svg**} with the structure annotated in the input alignment. R-scape] option –cyk produces the file **tutorial/manA.cyk.R2R.sto.**{**pdf,svg**} with the structure produced by R-scape. See Figure 1.

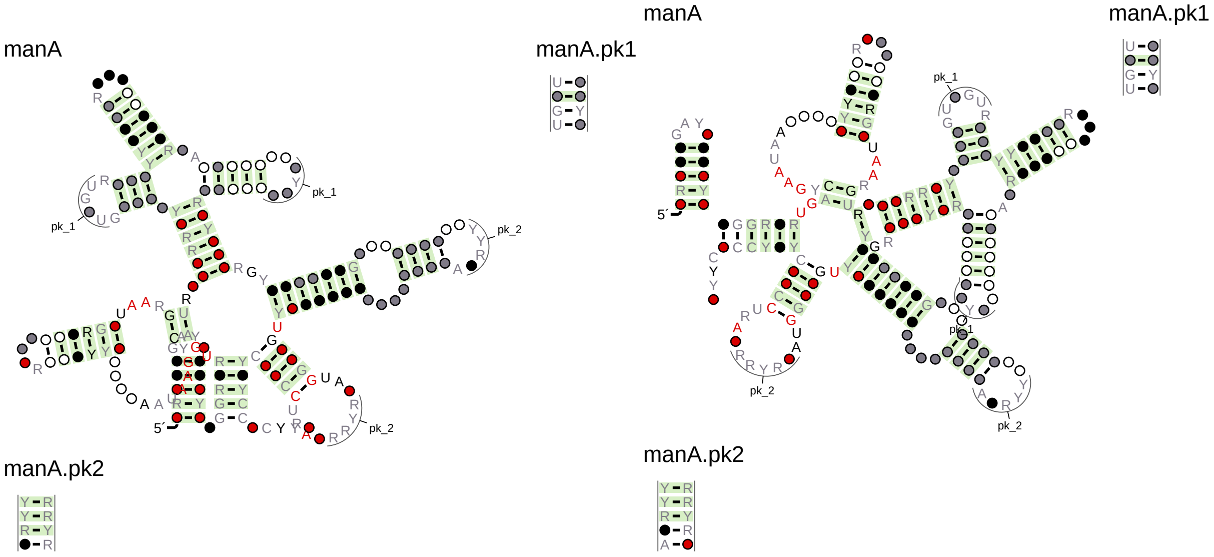

Figure 1: **Left: tutorial/manA.R2R.sto.**{**pdf,svg**}, the consensus secondary structure given in the input alignment, depicted by R-scape, using the program R2R. **Right: tutorial/manA.cyk.R2R.sto.**{**pdf,svg**}, The consensus structure produced by R-scape (option –cyk). Base pairs with covariation scores equal or below the target E-value (0.05 as default) are depicted in green.

Single sequence structure prediction

If the alignment includes only one sequence, no statistical test is performed.

```
> bin/R-scape --cyk tutorial/manA-oneseq.sto
```

reports the best structure given the sequence. No covariation support is possible for any of the basepairs reported from this analysis. Structures produced this way have to taken with great skepticism. See file `tutorial/manA-oneseq.cyk.sto` for the predicted structure, and `tutorial/manA-oneseq.cyk.R2R.sto.pdf` for an R2R drawing of the predicted structure.

Default parameters

Default parameters are:

`Target E-value:` default is 0.05. R-scape reports pairs which covariation score has E-value smaller or equal to the target value. The target E-value can be changed with option **`-E <x>`**, $x >= 0$.

`Sequence weighting:` Sequences are weighted according to the Gerstein/Sonnhammer/Chothia (GSC) algorithm (Gerstein et al., 1994). This algorithm is time consuming. For alignments with more than 1000 sequences, we use the faster position-based weighting algorithm (Henikoff and Henikoff, 1994). Both weighting algorithms are implemented as part of the easel library.

`Gaps in columns:` Columns with more than 50% gaps are removed. The gap threshold for removing columns can be modified using option **`--gapthresh <x>`** , $0 < x <= 1$.

`Covariation statistic:` The default covariation statistic is the average product corrected G-Test (equivalent to option **`--GTp`**).

`Covariation Class:` R-scape uses the 16 component covariation statistic (C16), unless the number of sequences in the alignment is ≤ 8 or the length of the alignment is ≤ 50, in which case it uses the two-class covariation statistic (C2). A particular covariation class can be selected using either **`--C16`** or **`--C2`**.

The threshold for the minimum number of sequences can be changed with option `--nseqthresh <n>`. The threshold for the minimum alignment length can be changed with option `--alenthresh <n>`.

`Null alignments:` In order to estimate E-values, R-scape produces 20 null alignments, unless the product of the number of sequences by the length of the alignment $<$ 10,000 in which case the number of null alignments is 50; or $<$ 1,000 in which case it is 100. The number of null alignments can be controlled with option **`--nshuffle <n>`**.

A full list of the R-scape options is found by using

```
> R-scape -h
```

4 Outputs

For each alignment file `rnafile.sto`, R-scape produces the following output files:

rnafile.out Tabular output with the significant pairs, with their score and E-value.

rnafile.sorted.out Tabular output sorted from highest to lowest E-value.

rnafile.sum Tabular output with a line summary statistics per alignment in the file.

Tabular output per input file

The distribution includes in the directory tutorials/ examples of output files. If you run R-scape, the outputs will go into your current working directory (not necessarily tutorials/).

The output file **tutorial/updated_Arisong.out** looks like this: **> more tutorial/updated_Arisong.out**

```
# MSA updated_Arisong_1 nseq 95 (95) alen 65 (150) avgid 66.35 (64.97) nbpairs 20 (20)
# Two-set statistical test (one test for annotated basepairs, another for all other pairs)
#
# Structure obtained from the msa
# Method Target_E-val [cov_min,conv_max] [FP | TP True Found | Sen PPV F]
# GTp    0.05          [-9.82,121.80]    [0 | 12 20 12 | 60.00 100.00 75.00]
#
#       left_pos       right_pos        score          E-value       substitutions      power
#-----------------------------------------------------------------------------------------------------
*              94             110      56.91065        0.00330664      34              0.35
*              96             108      89.46430        1.42871e-06     25              0.26
*              97             107      44.72409        0.0293269       58              0.55
...
```

The output file is a tabular list of significant pairs sorted by sequence positions:

`First column` indicates whether the significant pair is part of the given structure (*), or not. If the pair is not in the structure, we distinguish whether the pair is compatible with the given structure ($\sim$) or not (blank).

In addition, if the structure is provided by a PDB file (using the option `--pdbfile`), a non Watson-Crick/Watson-Crick base pair is designated by "**". A contact that is not a basepair is designated by: "$c\sim$" if compatible with all the basepairs, or by "c" otherwise.

`Second and third columns` are the two positions of the pair, $i \leq j$ respectively. Positions are relative to the input alignment.

`Fourth column` is the covariation score.

`Fifth column` is the E-value. Significant positions have E-values $<< 1$.

The output file also includes two comment lines per alignment in the file:

`First comment line` describes properties of the alignment: number of sequence (nseq), alignment length (alen), average percentage identity (avgid), and number of base pairs (nbpairs). Values in parentheses correspond to the alignment as given. Values not in parentheses correspond to the analyzed alignment after the filters (for redundant sequences and gapped columns) have been applied.

`Second comment line` describes properties of the R-scape search: the covariation method (GTp), the E-value threshold (0.05), the range of scores for all pairs in the alignments (from -9.7 to 89.1), the number of covarying non base pairs (0), the number of covarying base pairs (11), the number of base pairs (20), and the total number of covarying pairs (11). Lastly we provide the sensitivity (SEN=55.00=11/20), positive predictive value (PPV=100.00=11/11), and F-measure (F=70.97 = 2 * SEN * PPV / (SEN+PPV)).

Other tabular outputs

R-scape produces two more tabular outputs per input file that are more relevant for benchmarking purposes, those are:

File **tutorial/updated_Arisong.sum** looks like:
> more tutorial/updated_Arisong.sum

```
#target_E-val   MSA      nseq    alen    avgid   method  TP      True    Found   SEN      PPV
0.05            updated_Arisong_1       95      65      66.35    GTp 12  20  12  60.00  100.00
```

This file produces a one line output per alignment in the file.

`Column 1` Target E-value.

`Column 2` Alignment name.

`Column 3` Number of sequence in the analyzed alignment.

`Column 4` Number of columns analyzed.

`Column 5` Average percentage identity in the analyzed alignment.

`Column 6` Covariation statistic.

`Column 7` Number of significant base pairs, TP (true positives).

`Column 8` Number of base pairs, T (True).

`Column 9` Number of significant pairs, F (Found).

`Column 10` Sensitivity = TP/T.

`Column 11` Positive predictive value = TP/F.

Outputs per alignment

A Stockholm alignment file can include several different multiple sequence alignments (MSAs). R-scape produces the following output files, one for each individual alignment in the input Stockholm file:

Default

By default, the following files are produced

rnafile_msaname.R2R.sto Stockholm file annotated by a modified version of the R2R program. This file includes the information necessary to draw the consensus structure, and to annotate the significantly covarying base pairs.

rnafile_msaname.R2R.sto.{pdf,svg} Drawing of the R-scape-annotated consensus secondary structure.

rnafile_msaname.surv A two column file with the survival functions (surv) for the covariation scores.

rnafile_msaname.surv.ps Plot of the score's survival function $P(X > \text{score})$. Drawing this file requires that program **gnuplot** is installed somewhere in the `${PATH}`, or that the environmental variable GNUPLOT pointing to a gnuplot executable is defined.

rnafile_msaname.dplot.{ps,svg} Dot plot of the consensus secondary structure annotated according to covariation. Drawing of this file requires that program **gnuplot** is installed somewhere in the `${PATH}`, or that the environmental variable GNUPLOT pointing to a gnuplot executable is defined.

For each alignment, **msaname** is given by `<ACC>_<ID>`, the combination of the accession `#=GF AC <ACC>` and name `#=GF ID <ID>` in the Stockholm-format markups (or one of two if the other in not defined). If none of those fields are defined, **msaname** is a number describing the order in the file of the given alignment.

Details about outputs per alignment

Two files are produced per alignment in the input file:

File **tutorial/updated_Arisong_1.R2R.sto** is a Stockholm formatted alignment that includes the input alignment annotated with the consensus structure. This Stockholm file also includes the additional annotation required to use the drawing program R2R.

It is possible that the resulting drawing will show parts of the secondary structure occluded from each other (especially for long RNAs). Using this file, one can customize a different drawing of the structure using the R2R documentation, provided in `lib/R2R/R2R-manual.pdf`.

File **tutorial/updated_Arisong_1.surv** looks like this:

> more tutorial/updated_Arisong.surv

```
121.795428      0.05
95.862635       0.1
89.113004       0.15
...
 &
63.890698       0.000485437
58.917286       0.000970874
47.904730       0.00145631
...
 &
81.652885       2.40385e-06
77.745204       4.80769e-06
77.034717       7.21154e-06
...
 &
256.788050      2.64342e-17
256.432807      2.7899e-17
256.077563      2.94449e-17
...
 &
```

The first column is a covariation score (x). The second column is the survival function $P(X > x)$, that is the frequency of pairs having score larger than x. The file includes four survival functions separated by a "&" line. The three survival functions correspond to:

`First functions:` the given alignment, proposed base pairs. (This section is empty if no secondary structure is proposed.)

`Second functions:` the given alignment, not proposed pairs.

`Third function:` the aggregation of all null alignments, all possible pairs.

`Fourth function:` the expected null survival function according to the tail Gamma fit.

Using option **R-scape --cyk**

If the option `--cyk` is used, R-scape produces the following additional files describing the maximal-covariation optimal secondary structure:

rnafile_msaname.cyk.R2R.sto

rnafile_msaname.cyk.R2R.sto

rnafile_msaname.cyk.R2R.sto.{pdf,svg}

rnafile_msaname.cyk.surv

rnafile_msaname.cyk.surv.{ps.svg}

rnafile_msaname.cyk.dplot.{ps,svg}

These files are formatted identically to those describing the given consensus structure.

Graphical outputs per alignment

Three plots are produced per alignment in the input file:

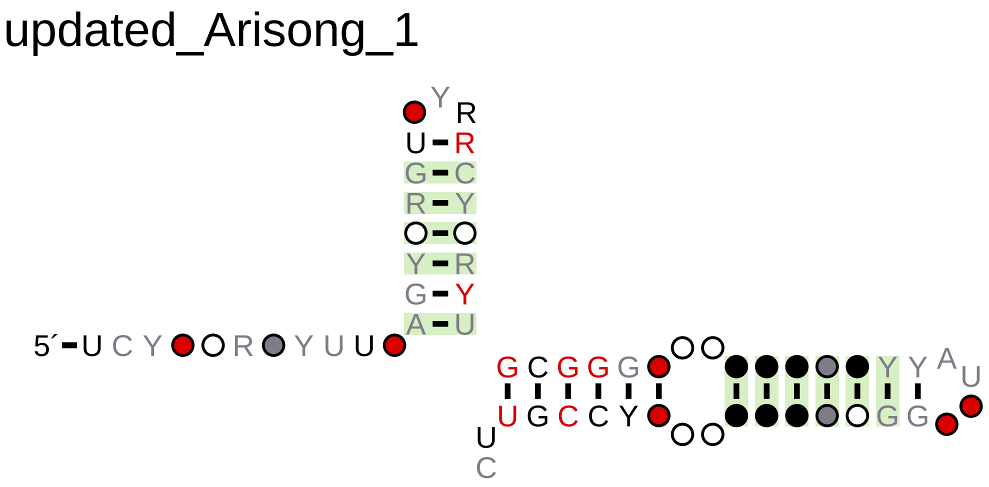

Figure 2: **`tutorial/updated_Arisong_1.R2R.sto.`{`pdf,svg`}`:` annotated consensus secondary structure.** Base pairs with covariation scores equal or below the target E-value (0.05 as default) are depicted in green. By default only positions in the alignment with more than 50% occupancy are depicted (unless they form a base pair). Option `--r2rall` forces the depiction of all positions in the alignment.

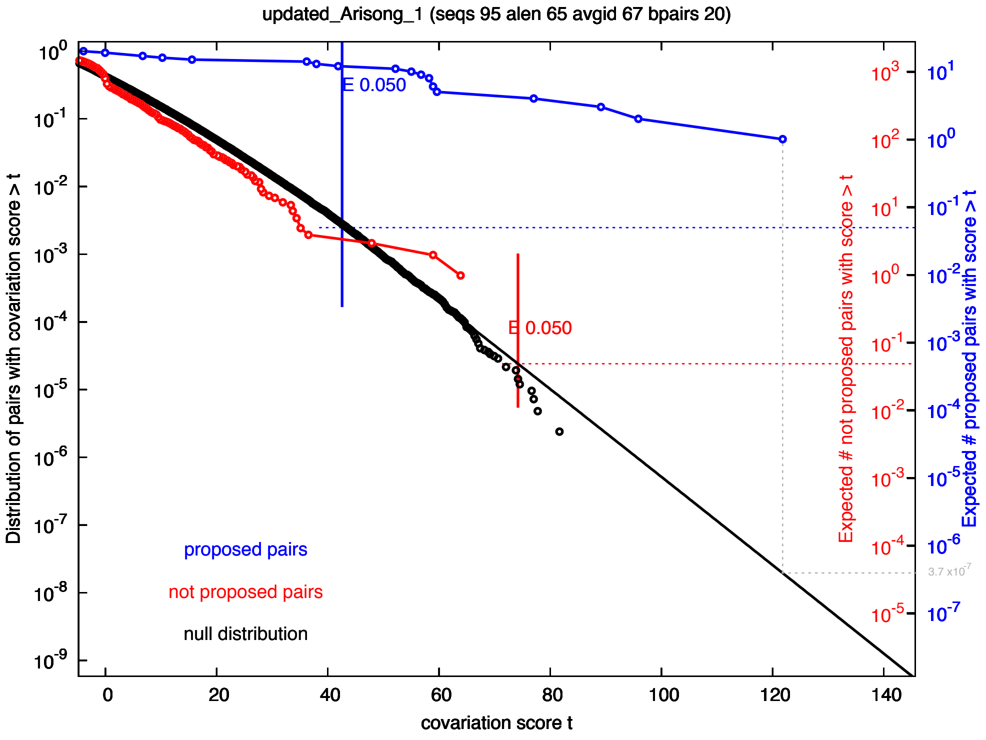

Figure 3: **tutorial/updated_Arisong_1.surv.{ps,svg}: covariation scores survival function** $P(X > x)$**.** The survival function of scores for all pairs in the given alignment is depicted in blue. The survival function for the null alignments is depicted in black. A black line indicates to fit to a truncated Gamma distribution of the tail of the null distribution. In red, we plot the survival function of scores for the pairs in the given alignment excluding those proposed as base pairs. For a particular pair, as an example the highest scoring one from the distribution of proposed pairs (blue), we obtain its E-value by drawing a vertical (gray) line from the point to the null distribution (black). The corresponding value in the blue scale gives us the E-value for that pair (in this example, $3.7 \cdot 10^{-7}$).

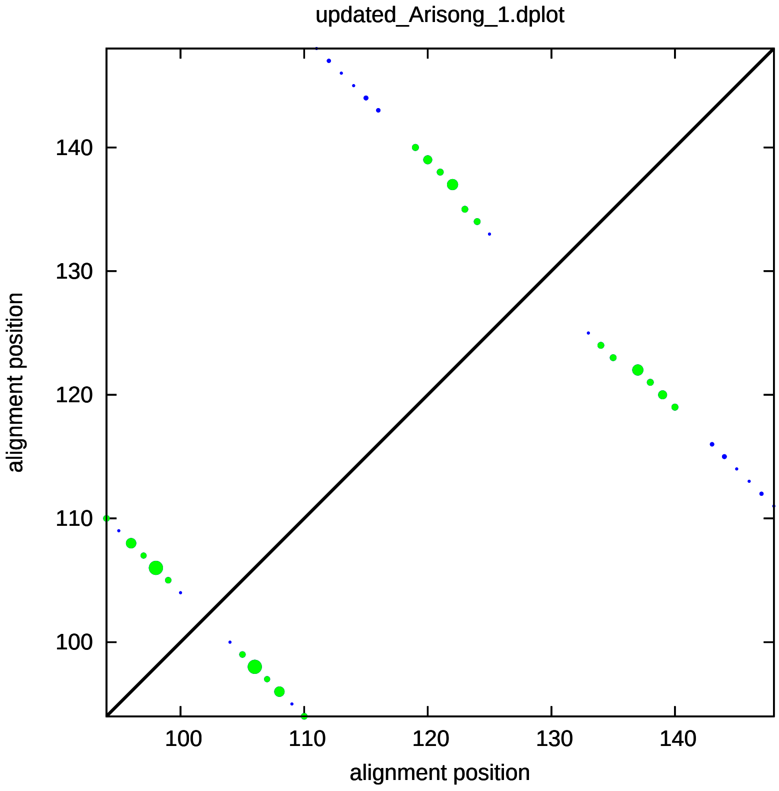

Figure 4: **`tutorial/updated_Arisong_1.dplot.{ps,svg}`: dotplot.** Dot size is proportional to the covariation score. In blue we depict the consensus base pairs; in green, the consensus base pairs that show significant covariation; in orange (none shown in this plot), we depict other pairs that have significant covariation, are not part of the consensus secondary structure but are compatible with it; in black we depict other significant pairs. Position are relative to the original input alignment (before any gapped column is removed).

5 Options

The whole list of options can be found using

```
> R-scape -h
```

Some important options are:

Covariation statistic options

-E <x>

Target E-value is $x \geq 0$.

--GT, --MI, --MIr, --MIg, --CHI, --OMES, --RAF, --RAFS,

We favor the G-test covariation statistic, but a total of eight covariation statistics are currently implemented in R-scape. For each covariation statistic (GT, for instance), R-scape can also calculate its average product correction (GTp) and its average sum corrections (GTa). For each option above, appending "p" or "a" chooses one of the corrections. For example, `--GT` does the G-test statistic, `--GTp` does the APC-corrected G-test statistic, `--GTa` does the ASC-corrected G-test statistic.

The R-scape default is `--GTp`.

Details of the definition and provenance of the different covariation statistics can be found in the R-scape manuscript: Rivas, E. & Eddy S. E., *"A statistical test for conserved RNA structure shows lack of evidence for structure in lncRNAs"*.
In a nutshell, given two alignment columns i, j,

G-test:(Woolf, 1957)	$\mathrm{GT}(i,j)$	$=$	$2\sum_{a,b} \mathrm{Obs}_{ij}^{ab} \log \frac{\mathrm{Obs}_{ij}^{ab}}{\mathrm{Exp}_{ij}^{ab}}$,
Pearson's chi-square:	$\mathrm{CHI}(i,j)$	$=$	$\sum_{a,b} \frac{\left(\mathrm{Obs}_{ij}^{ab}-\mathrm{Exp}_{ij}^{ab}\right)^2}{\mathrm{Exp}_{ij}^{ab}}$,
Mutual information:(Shannon, 1948; Gutell et al., 1994)	$\mathrm{MI}(i,j)$	$=$	$\sum_{a,b} P_{ij}^{ab} \log \frac{P_{ij}^{ab}}{p_i^a\, p_j^b}$,
MI normalized:(Martin et al., 2005)	$\mathrm{MIr}(i,j)$	$=$	$\frac{\mathrm{MI}(i,j)}{H(i,j)} = \frac{\mathrm{MI}(i,j)}{-\sum_{a,b} P_{ij}^{ab} \log P_{ij}^{ab}}$,
MI with gap penalty:(Lindgreen et al., 2006)	$\mathrm{MIg}(i,j)$	$=$	$\mathrm{MI}(i,j) - \frac{N_{ij}^G}{N}$,
Obs-Minus-Exp-Squared:(Fodor and Aldrich, 2004)	$\mathrm{OMES}(i,j)$	$=$	$\sum_{a,b} \frac{\left(\mathrm{Obs}_{ij}^{ab}-\mathrm{Exp}_{ij}^{ab}\right)^2}{N_{ij}}$,
RNAalifold (RAF):(Hofacker et al., 2002)	$\mathrm{RAF}(i,j)$	$=$	$\mathrm{B}_{i,j}$,
RNAalifold Stacking (RAFS):(Lindgreen et al., 2006)	$\mathrm{RAFS}(i,j)$	$=$	$\frac{1}{4}\left(\mathrm{B}_{i-1,j+1} + 2\,\mathrm{B}_{i,j} + \mathrm{B}_{i+1,j-1}\right)$.

where a, b are (non-gap) residues; N is the total number of aligned sequences; Obs_{ij}^{ab} is the observed count of $a : b$ pairs in columns i, j (only counting when both a,b are residues); N_{ij} is the total number of residue pairs in columns i, j (only counting when both a,b are residues); P_{ij}^{ab} is the observed frequency of pair $a : b$ in columns i, j ($P_{ij}^{ab} = \frac{Obs_{ij}^{ab}}{N_{ij}}$); $\mathrm{Exp}_{ij}^{ab} = N_{ij} p_i^a p_j^b$ is the expected frequency of pair $a : b$ assuming i, j are independent, where p_i^a are the marginal frequencies of a residues in column i (averaged to all other positions) ($p_i^a = \frac{1}{L-1} \sum_{j \neq i} \sum_b P_{ij}^{ab}$); $N_{ij}^G = N - N_{ij}$ is the number of pairs involving at least one gap symbol; the definition of $\mathrm{B}_{i,j}$ used in the RAF and RAFS statistics is involved, a concise definition can be found elsewhere (Lindgreen et al., 2006).

The background corrections (Dunn et al., 2007) for a given covariation statistic above $\mathrm{COV}(i,j)$ are,

Average product correction	$\mathrm{COVp}(i,j)$	$=$	$\mathrm{COV}(i,j) - \frac{\mathrm{COV}(i)\mathrm{COV}(j)}{\mathrm{COV}}$,
Average sum correction	$\mathrm{COVa}(i,j)$	$=$	$\mathrm{COV}(i,j) - \left(\mathrm{COV}(i) + \mathrm{COV}(j) - \mathrm{COV}\right)$.

--C2, --C16

For all the covariation statistics (except RAF and RAFS), one can do a 16-component (C16) or a two-component (C2) calculation, depending on whether it uses the 16 possible pair combinations, or those are group in two classes depending on whether they form a Watson-Crick pair (6 cases, including U:G and G:U), or whether they do not (10 cases).

R-scape's default is the 16 component covariation statistic, unless the number of sequences in the alignment is $\leq$ 8 or the length of the alignment is $\leq$ 50, in which case it uses the two-class covariation statistic.

Search options

-s

The "two-set test" option. This option requires that a structure is provided with the alignment. If option `-s` is used, R-scape performs two independent test, one for the given structure, another for all other possible pairs. The default is a "one-set test" in which all possible pairs in the alignment are tested equivalently.

--cyk

An optimal secondary structure is computed that includes all significant base pairs. The files for this maximum-covariation optimal structure all include the suffix `.cyk`.

When option `--cyk` is used, a file with the original alignment annotated with the R-scape structure in Stockholm format is produced. This alignment has the suffix `.cyk.sto`.

--naive

Reports the laundry list of all covariation scores, without any statistical significance (E-value) associated to them. No null alignments are created.

--tstart <n>

Analyze starting from position $n >= 1$ in the alignment.

--tend <n>

Analyze ending at position $n <= L$ in the alignment.

--window <n>

R-scape can be run in a window scanning version for long alignments. The window size is $n > 0$.

--slide <n>

In scanning mode, this options sets the number of positions to move from window to window, $n > 0$.

--vshuffle

Vertical shuffle, a developers tool. Before performing any analysis, it shuffles all residues in each alignment column independently.

--cshuffle

Column shuffle, a developers tool. Before performing any analysis, it shuffles all columns in the alignment.

Input alignment options

-I <x>

Only sequences with less than $0 < x \leq 1$ pairwise similarity are considered in the analysis. Pairwise $\%$ identity is defined as the ratio of identical positions divided by the minimum length of the two sequences. If this option is not used all (weighted) sequences are used in the analysis.

--gapthresh <x>

Only columns with less than $0 < x \leq 1$ fraction of gaps are considered in the analysis.

--consensus

If the alignment has a GC "seq_cons" field, only consensus positions will be analyzed.

--submsa <n>

Analyzes a random subset of the input alignment.

--treefile <f>

A phylogenetic tree in Newick format can be given (by default a tree is created from the alignment using the program FastTree (Price et al., 2010)). R-scape checks that the number of taxa and the names of the taxa matches for all alignments analyzed.

Options for importing a structure

R-scape does not require to input a structure (either a RNA structure or a protein contact map). By default R-scape analyzes all possible pairs in the alignment.

There are two ways to provide a contact map (or structure):

- By providing the alignment in Stockholm format with a "ss_cons" field including the consensus structure for the alignment. (For RNA alignments only.)
- By analyzing a 3D structure provided in a PDB file. (For either RNA or peptide alignments.)

These two methods can be combined together. For a nucleotide alignment, if both a consensus structure is present in the alignment, and a PDB file is provided (using option `--pdbfile`), the consensus structure will be extended by the information provided by the pdbfile. To ignore the consensus structure use option `--onlypdb`.

From the PDB file we obtain three types of structural pairs:

- **Contacts:** defined as those two residues at a close spatial distance (specified by the user with option `--cntmaxD`).
- **Basepair:** RNA basepairs.
  RNA basepairs are calculated using the program `rnaview` (Yang et al., 2003).

  These RNA basepairs can be further classified in two types:

  - **Watson-Crick basepairs:** the canonical RNA basepairs. mostly A:U, G:C, or G:U pairs. (H-bond interactions between two W-C faces in cis).
  - **Other basepairs:** the non-canonical RNA basepairs (all other types of H-bond interactions, 12 different types).

Contacts and RNA basepairs are extracted as follows:

- The spatial distance between any two residues is calculated as the minimal Euclidean distance between any two atoms (excluding H atoms). Any two pairs at a distance not larger than a maximum value (`contmaxD`) are called a "contact".
- RNA basepairs are obtained using the program `rnaview` (Yang et al., 2003) (http://ndbserver.rutgers.edu/ndbmodule/services/download/rnaview.html).
  The RNA basepair annotation takes precedent over the annotation as "contact".

The options that control the input of a structure or contact map are:

--pdbfile <s>

Reads a pdbfile associated to the alignment, and extracts the contacts from it.
A ".cmap" file is produced reporting the structure obtained from the PDB file.
Option `--pdbfile` is incompatible with `--cyk`.

--cntmaxD <x>

Maximum distance (in Angstroms) allowed between two residues to define a "contact" is $\langle x \rangle$.

--cntmind <n>

Minimum distance (in residue positions) in the backbone between two residues required to define a "contact" is $\langle n \rangle$.

--onlypdb

Reads the structure from the pdbfile and ignores the alignment consensus structure (if provided).

Example of reading a structure from a PDB file for the FMN riboswitch:

> bin/R-scape --cntmaxD 4 --cntmind 3 --pdbfile tutorial/3f2q.pdb -s --onlypdb tutorial/RF00050

This command line extracts contacts from the pdb file that are at a Euclidean distance $\leq 4\mathring{A}$ in the PDB structure, and such that they are at least 3 residues apart in the backbone.
The output is

```
# R-scape :: RNA Structural Covariation Above Phylogenetic Expectation
# R-scape 0.8.1 (Jul 2018)
# Copyright (C) 2016 Howard Hughes Medical Institute.
# Freely distributed under the GNU General Public License (GPLv3).
# - - - - - - - - - - - - - - - - - - - - - - - - - - - - - - - - - - - - -
# Two-set statistical test (one test for annotated basepairs, another for all other pairs)
#
# Structure obtained from the pdbfile
# ij in alignment | ij in pdbsequence | basepair type
# 3 218 | 1 112 | WWc
# 4 216 | 2 110 | CONTACT
# 4 217 | 2 111 | WWc
# 4 218 | 2 112 | CONTACT
# 5 216 | 3 110 | WWc
# 5 217 | 3 111 | CONTACT
# 6 215 | 4 109 | WWc
# 6 216 | 4 110 | CONTACT
# .
# .
# .
# 192 202 | 87 96 | WWc
```

```
# 192 203 | 87 97 | CONTACT
# 193 198 | 88 92 | CONTACT
# 193 201 | 88 95 | WWc
# 193 202 | 88 96 | CONTACT
# 195 197 | 89 91 | CONTACT
# 195 198 | 89 92 | WHt
# 198 200 | 92 94 | CONTACT
# 198 201 | 92 95 | CONTACT
# 205 207 | 99 101 | CONTACT
# PDB:      versions/rscape/rscape_v0.8/tutorial/3f2q.pdb
# contacts  169 (49 bpairs 35 wc bpairs)
# maxD      4.00
# mind      3
# distance  MIN
# L         139
# alen      221
# pdblen    112
# ::[[[[[[[[,,,,,,<<<_______>>>,((((<<<<_________________AA>>>>,,<<<----------<____>>>>,,,<<<<<_______>>>>>aa))AAAA----))a
# MSA RF00050_FMN.3f2q nseq 144 (144) alen 139 (221) avgid 69.18 (68.15) nbpairs 49 (0)
#
# Method Target_E-val [cov_min,conv_max] [FP | TP True Found | Sen PPV F]
# GTp    0.05         [-9.78,216.11]     [1 | 14 49 15 | 28.57 93.33 43.75]
#
#       left_pos       right_pos        score           E-value
#------------------------------------------------------------------
*             171            183        216.11095        1.6421e-10
*             170            184        211.69081        2.76699e-10
*             192            202        168.72417        4.95548e-08
*               8            213        149.71776        4.89982e-07
*             172            182        138.66664        1.84675e-06
*             169            185        137.23189        2.21548e-06
**             16             30         133.44999        3.53772e-06
*               5            216        131.02575        4.70876e-06
*              84            186        125.60806        9.0169e-06
*              17             29        112.04610        4.62895e-05
*               7            214        111.12654        5.13519e-05
*               6            215        96.43781        0.00029929
*              36             87        96.32752        0.00029929
*              94            163        78.81578        0.0024303
                7            213        107.68588        0.0147937
```

All coordinates are relative to the input alignment. The annotation of all types of RNA basepairs (WWc, WWt, WHc,...) is produced by the program `rnaview` (Yang et al., 2003).

Options for type of pairs tested

When performing the two-class statistical test (option `-s`) using a pdbfile to read the structure, there are different options as to which types of basepairs are used to define the sample size for the basepairs test.

The options are:

`--samplecontacts`

The basepair statistical test includes all the contacts identified in a PDB or/and as a RNA secondary structure included with a input alignment in Stockholm format. This is the default option for amino acid alignments if a PDB file is provided.

`--samplebp`

For RNA alignments with only. The basepair statistical test includes basepairs of all 12 possible types. This is the default option for RNA/DNA alignments if a PDB file is provided.

`--samplewc`

For RNA alignments only. The basepair statistical test includes only the canonical (Watson-Crick/Watson-Crick type) basepairs (A:U, G:C, G:U). This is the default option for RNA/DNA alignments if a consensus secondary structure is provided.

Output options

--roc

Produces a tabular output that provides statistics for each score value.

File **tutorial/updated_Arisong.roc** looks like:

> more tutorial/updated_Arisong.roc

```
# MSA nseq 95 alen 65 avgid 66.352419 nbpairs 20 (20)
# Method: GTp
#cov_score  FP  TP Found  True  Negatives  Sen   PPV     F       E-value
121.79543   0   2  2      20    2060       10.00 100.00  18.18   4.07104e-05
121.44018   0   2  2      20    2060       10.00 100.00  18.18   4.29443e-05
121.08494   0   2  2      20    2060       10.00 100.00  18.18   4.53006e-05
120.72970   0   2  2      20    2060       10.00 100.00  18.18   4.53006e-05
...
```

This file produces a tabular output for each alignment as a function of the covariation score, for plotting ROC curves. The values in the file are described by the comment line. Notice that the number of Trues (column 5) and Negatives (column 6) are fixed for a given secondary structure and do not change.

--outmsa <f>

The actual alignment analyzed can be saved in Stockholm format to file <f>.

--outtree <f>

The phylogenetic tree (created using the program FastTree) can be saved in Newick format to file <f>.

Plotting options

--nofigures

None of the graphical outputs are produced using this option.

--r2rall

Forces R2R to draw all positions in the alignment. By default only those that are more than 50% occupied or are base paired are depicted.

Other options

--seed <n>

Sets the seed of the random number generator to <n>. Use n = 0 for a random seed.

6 Some other topics

How do I cite R-scape?

Rivas, E. *et al.*, *"A statistical test for conserved RNA structure shows lack of evidence for structure in lncRNAs"*, Nature Methods 14, 4548 (2017).

You should also cite what version of the software you used. We archive all old versions, so anyone should be able to obtain the version you used, when exact reproducibility of an analysis is an issue.

The version number is in the header of most output files. To see it quickly, do something like `R-scape -h` to get a help page, and the header will say:

```
# R-scape :: RNA Structural Covariation Above Phylogenetic Expectation
# R-scape 0.8.1 (July 2018)
# Copyright (C) 2016 Howard Hughes Medical Institute.
# Freely distributed under the GNU General Public License (GPLv3).
# - - - - - - - - - - - - - - - - - - - - - - - - - - - - - - - - - - - - -
```

So (from the second line there) this is from R-scape v0.8.1.

How do I report a bug?

Email us, at `elenarivas@fas.harvard.edu`.

Before we can see what needs fixing, we almost always need to reproduce a bug on one of our machines. This means we want to have a small, reproducible test case that shows us the failure you're seeing. So if you're reporting a bug, please send us:

- A brief description of what went wrong.
- The command line(s) that reproduce the problem.
- Copies of any files we need to run those command lines.
- Information about what kind of hardware you're on, what operating system, and what compiler and version you used, with what configuration arguments.

7 Acknowledgments

We thank S.E. Roian Egnor for suggesting the name R-scape, and the Centro de Ciencias de Benasque Pedro Pascual in Spain, for their hospitality, over numerous and wonderful summers.

References

del Val, C., Romero-Zaliz, R., Torres-Quesada, O., Peregrina, A., Toro, N., and Jiménez-Zurdo, J. I. (2012). A survey of sRNA families in α-proteobacteria. *RNA Biol*, 9:119–129.

Dunn, S. D., Wahl, L. M., and Gloor, G. B. (2007). Mutual information without the influence of phylogeny or entropy dramatically improves residue contact predictions. *Bioinformatics*, 24:333–340.

Fodor, A. A. and Aldrich, R. W. (2004). Influence of conservation on calculations of amino acid covariance in multiple sequence alignments. *Proteins: Structure, Function, and Bioinformatics*, 56(2):211–221.

Gerstein, M., Sonnhammer, E. L. L., and Chothia, C. (1994). Volume changes in protein evolution. *J. Mol. Biol.*, 235:1067–1078.

Gutell, R. R., Larsen, N., and Woese, C. R. (1994). Lessons from an evolving rRNA: 16S and 23S rRNA structures from a comparative perspective. *Microbiol. Rev.*, 58:10–26.

Henikoff, S. and Henikoff, J. G. (1994). Position-based sequence weights. *J. Mol. Biol.*, 243:574–578.

Hofacker, I. L., Fekete, M., and Stadler, P. F. (2002). Secondary structure prediction for aligned RNA sequences. *J. Mol. Biol.*, 319:1059–1066.

Jung, S., Swart, E. C., Minx, P. J., Magrini, V., Mardis, E. R., Landweber, L. F., and Eddy, S. R. (2011). Exploiting *Oxytricha trifallax* nanochromosomes to screen for noncoding RNA genes. *Nucl. Acids Res.*, 39:7529–7547.

Lindgreen, S., P.P., G., and Krogh, A. (2006). Measuring covariation in RNA alignments: physical realism improves information measures. *Bioinformatics*, 22:2988–2995.

Martin, L., Gloor, G., Dunn, S., and Wahl, L. (2005). Using information theory to search for co-evolving residues in proteins. *Bioinformatics*, 21:4116–4124.

Nawrocki, E. P., Burge, S. W., Bateman, A., Daub, J., Eberhardt, R. Y., Eddy, S. R., Floden, E. W., Gardner, P. P., Jones, T. A., Tate, J., and Finn, R. D. (2015). Rfam 12.0: updates to the RNA families database. *Nucl. Acids Res.*, 43:D130–D137.

Price, M. N., Dehal, P. S., and Arkin, A. P. (2010). FastTree 2 - approximately maximum-likelihood trees for large alignments. *PLOS ONE*, 5:e9490.

Shannon, C. (1948). A note on the concept of entropy. *Bell System Tech. J*, 27:379–423.

Weinberg, Z. (2018). Zasha Weinberg Database. [https://bitbucket.org/zashaw/zashaweinbergdata/].

Weinberg, Z. and Breaker, R. R. (2011). R2R – software to speed the depiction of aesthetic consensus RNA secondary structures. *BMC Bioinformatics*, 12:3.

Weinberg, Z., Perreault, J., Meyer, M. M., and Breaker, R. R. (2009). Exceptional structured noncoding RNAs revealed by bacterial metagenome analysis. *Nature*, 462:656–659.

Weinberg, Z., Wang, J. X., Bogue, J., Yang, J., Corbino, K., Moy, R. H., and Breaker, R. R. (2010). Comparative genomics reveals 104 candidate structured RNAs from bacteria, archaea and their metagenomes. *Genome Biol*, 11 (3): R31.

Woolf, B. (1957). The log likelihood ratio test (the G-test). *Annals of Human Genetics*, 21:397–409.

Yang, H., Jossinet, F., Leontis, N., Chen, L., Westbrook, J., Berman, H. M., and Westhof, E. (2003). Tools for the automatic identification and classification of RNA base pairs. *Nucl. Acids Res.*, 31.13:3450–3460.
