# Supplementary material for: Estimating the power of sequence covariation for detecting conserved RNA structure
Source: Bioinformatics. 2020 Feb 7;36(10):3072–6. doi: 10.1093/bioinformatics/btaa080 (PMC7214042; doi:10.1093/bioinformatics/btaa080)
Supplement: btaa080_Supplementary_Data [file btaa080_supplementary_data.zip › btaa080-Suppl_Data/src/rscape_v1.2.3.tar.gz!/rscape_v1.2.3/lib/R2R/R2R-current/R2R-manual/figures/demo-breakpair-varlen.pdf]

A

```
# STOCKHOLM 1.0
human          ACACGCGAAA.GCGCAA.CAAACGUGCACGG
chimp          GAAUGUGAAAACACCA.CUCUUGAGGACCU
bigfoot        UUGAG.UUCG..CUCGUUUUCUCGAGUACAC
#=GC SS_cons   ...<<<.....>>>....<<....>>.....
#=GC R2R_LABEL ......................1...2...
#=GF R2R var_backbone_range 1 2
//
```

B

```
ERROR: there was a problem drawing motif
"demo-breakpair-varlen": One or more pairs
is getting broken by one side getting
deleted (presumably because it's not con-
served), while the other one stays.
Because of command on line #7.  Consider
using #=GF R2R keep allpairs.  (Note:
another explanation is that you've used
#=GF R2R var_backbone_range on columns that
includes a pair.   (text columns in align-
ment [33,40], left=keep,right=chuck, left
context=nR-nCnCnn-Y) (text columns in
alignment [34,39], left=keep,right=chuck,
left context=R-nCnCnn-Yn)
```
