# Supplementary material for: Estimating the power of sequence covariation for detecting conserved RNA structure
Source: Bioinformatics. 2020 Feb 7;36(10):3072–6. doi: 10.1093/bioinformatics/btaa080 (PMC7214042; doi:10.1093/bioinformatics/btaa080)

A

```
# STOCKHOLM 1.0
lemur          AAACUACCCCUAUUGG
blueberry      AAACAUCCCCAU-UGG
smurf          AAGCAACCCCUU-CGG
#=GC SS_cons   ..<<<<....>>>>..
//
```

(Column numbers 0–28 shown above the alignment)

B

```
ERROR: there was a problem drawing motif "demo-
breakpair": One or more pairs is getting broken by one
side getting deleted (presumably because it's not
conserved), while the other one stays.  Because of
generic process (probably RemoveGaps), and not the
result of any specific command.  Consider using #=GF
R2R keep allpairs.  (Note: another explanation is that
you've used #=GF R2R var_backbone_range on columns
that includes a pair.   (text columns in alignment
[16,25], left=keep,right=chuck, left context=AARC)
```

C

```
# STOCKHOLM 1.0
lemur          AAACUACCCCUAUUGG
blueberry      AAACAUCCCCAU-UGG
smurf          AAGCAACCCCUU-CGG
#=GC SS_cons   ..<.<<....>>.>..
//
```

D

```
# STOCKHOLM 1.0
lemur            AAACUACCCCUAUUGG
blueberry        AAACAUCCCCAU-UGG
smurf            AAGCAACCCCUU-CGG
#=GC SS_cons     ..<<<<....>>>>..
#=GC R2R_LABEL   ...-........-...
//
```

E

```
# STOCKHOLM 1.0
lemur            AAACUACCCCUAUUGG
blueberry        AAACAUCCCCAU-UGG
smurf            AAGCAACCCCUU-CGG
#=GC SS_cons     ..<<<<....>>>>..
#=GC R2R_LABEL   ...p........p...
#=GF R2R depair p
//
```

F

```
# STOCKHOLM 1.0
lemur            AAACUACCCCUAUUGG
blueberry        AAACAUCCCCAU-UGG
smurf            AAGCAACCCCUU-CGG
#=GC SS_cons     ..<<<<....>>>>..
#=GC R2R_LABEL   ...p........p...
#=GF R2R keep p
//
```

G

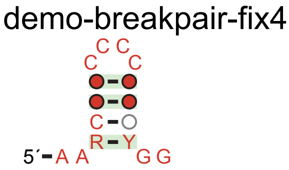

Supplement: btaa080_Supplementary_Data [file btaa080_supplementary_data.zip › btaa080-Suppl_Data/src/rscape_v1.2.3.tar.gz!/rscape_v1.2.3/lib/R2R/R2R-current/R2R-manual/figures/demo-breakpair.pdf]
