# Supplementary material for: Estimating the power of sequence covariation for detecting conserved RNA structure
Source: Bioinformatics. 2020 Feb 7;36(10):3072–6. doi: 10.1093/bioinformatics/btaa080 (PMC7214042; doi:10.1093/bioinformatics/btaa080)
Supplement: btaa080_Supplementary_Data [file btaa080_supplementary_data.zip › btaa080-Suppl_Data/src/rscape_v1.2.3.tar.gz!/rscape_v1.2.3/lib/R2R/R2R-current/R2R-manual/figures/demo-ii.pdf]

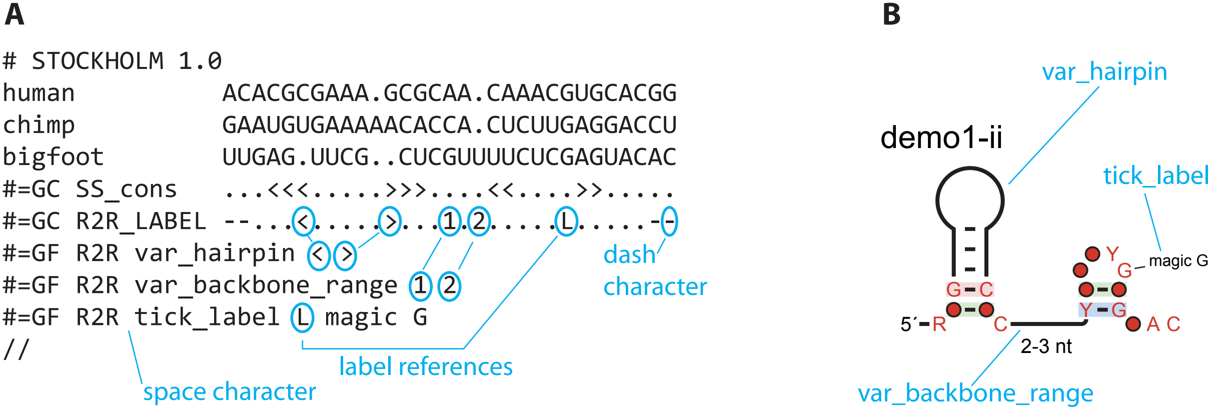
A
STOCKHOLM 1.0
human ACACGCGAAA.GCGCAA.CAAACGUGCACGG
chimp GAAUGUGAAAACACCA.CUCUUGAGGACCU
bigfoot UUGAG.UUCG..CUCGUUUCUCGAGUACAC
#=GC SS_cons ...<<<.....>>>....<<....>>.....
#=GC R2R_LABEL --...<.....>...1.2.....L.....--
#=GF R2R var_hairpin < >
#=GF R2R var_backbone_range 1 2
#=GF R2R tick_label L magic G
//
dash character
label references
space character
B
demo1-ii
var_hairpin
tick_label
magic G
Y
G
G-C
Y-G
5´-R
C
A C
2-3 nt
var_backbone_range
