# Supplementary material for: Estimating the power of sequence covariation for detecting conserved RNA structure
Source: Bioinformatics. 2020 Feb 7;36(10):3072–6. doi: 10.1093/bioinformatics/btaa080 (PMC7214042; doi:10.1093/bioinformatics/btaa080)

A

```
intermediate/demo1-iii.cons.sto
demo1-iii.sto        oneseq bigfoot
intermediate/demo1-iii.cons.sto        skeleton-with-pairbonds
```

tab character

B

```
# STOCKHOLM 1.0
human                     ACACGCGAAA.GCGCAA.CAAACGUGCACGG
chimp                     GAAUGUGAAAACACCA.CUCUUGAGGACCU
bigfoot                   UUGAG.UUCG..CUCGUUUCUCGAGUACAC
#=GR bigfoot DEL_COLS     .........................-----
#=GC SS_cons              ...<<<.....>>>....<<....>>.....
#=GC R2R_LABEL            --...<.....>...1.2.....Lsssss--
#=GC R2R_XLABEL_bf        ......UUUU.....................
#=GC R2R_XLABEL_bfl       ........U......................
#=GF R2R var_hairpin < >
#=GF R2R var_backbone_range 1 2
#=GF R2R tick_label L magic G
#=GF R2R_oneseq bigfoot outline_nuc bf:U
#=GF R2R_oneseq bigfoot tick_label bfl:U bigfoot UNCG loop
#=GF R2R if_skeleton SetDrawingParam pairBondWidth 0.5pt
#=GF R2R if_skeleton shade_along_backbone s rgb:255,0,0
//
```

C

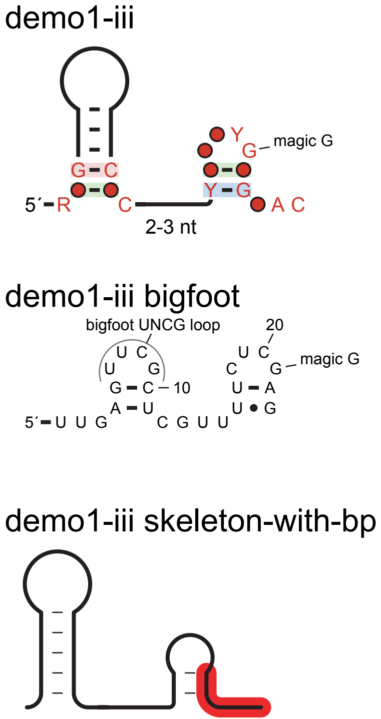

Supplement: btaa080_Supplementary_Data [file btaa080_supplementary_data.zip › btaa080-Suppl_Data/src/rscape_v1.2.3.tar.gz!/rscape_v1.2.3/lib/R2R/R2R-current/R2R-manual/figures/demo-iii.pdf]
