# Supplementary material for: Estimating the power of sequence covariation for detecting conserved RNA structure
Source: Bioinformatics. 2020 Feb 7;36(10):3072–6. doi: 10.1093/bioinformatics/btaa080 (PMC7214042; doi:10.1093/bioinformatics/btaa080)

A

```
# STOCKHOLM 1.0
rope                         AGGCAUUUGAACCAUAUUGUGCGCCUAACAUC..GCCAAAGCACAA
speed                        GGGUAUUUGAACUGUAUUAUGCACCCAGCAUAAUGUGGAACCAUAA
topology                     AGGUAUUUGAACCGUAUUGUGCACCUAGCAUGA.GUUAAAGCACAA
#=GC SS_cons                 <<<<..................>>>>....................
#=GC SS_cons_1               ..................<<<<..................>>>>..
#=GC R2R_LABEL               ...............................1.2...3........
#=GC SUBFAM_pknot_R2R_LABEL  ------------------....------------------....--

#=GF R2R ignore_ss_except_for_pairs _1 outline
#=GF R2R var_backbone_range 1 2
#=GF R2R place_explicit 3 3-- -45 1 0 0 0 -90

#=GF SUBFAM_PERL_PRED pknot return 1;
#=GF SUBFAM_pknot_R2R subst_ss _1 primary
#=GF SUBFAM_pknot_R2R no5
#=GF SUBFAM_pknot_R2R outline_nuc all
#=GF SUBFAM_pknot_R2R set_dir pos0 90 f
//
```

B

demo-pknot-callout

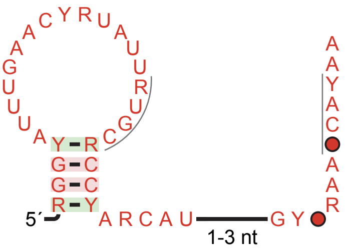

demo-pknot-callout-pknot

subfam_weight=1

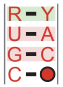

C

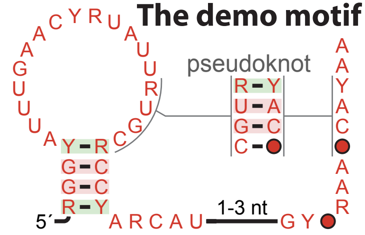

Supplement: btaa080_Supplementary_Data [file btaa080_supplementary_data.zip › btaa080-Suppl_Data/src/rscape_v1.2.3.tar.gz!/rscape_v1.2.3/lib/R2R/R2R-current/R2R-manual/figures/demo-pknot-callout.pdf]
