# Supplementary material for: Estimating the power of sequence covariation for detecting conserved RNA structure
Source: Bioinformatics. 2020 Feb 7;36(10):3072–6. doi: 10.1093/bioinformatics/btaa080 (PMC7214042; doi:10.1093/bioinformatics/btaa080)

A

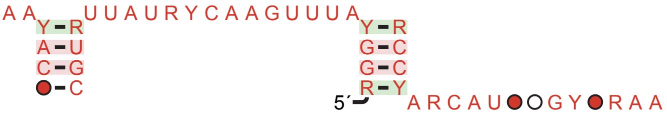

B

```
# STOCKHOLM 1.0
rope                     AGGCAUUUGAACCAUAUUGUGCGCCUAACAUC..GCCAAAGCACAA
speed                    GGGUAUUUGAACUGUAUUAUGCACCCAGCAUAAUGUGGAACCAUAA
topology                 AGGUAUUUGAACCGUAUUGUGCACCUAGCAUGA.GUUAAAGCACAA
#=GC SS_cons             <<<<..................>>>>....................
#=GC SS_cons_1           ..................<<<<..................>>>>..
#=GC R2R_LABEL           ....a.................t...b....1.2..........3.

#=GF R2R place_explicit t-- t 0 -1 0 0 0 0 f
#=GF R2R place_explicit 3 3-- +45 1 0 0 0 +90 f
#=GF R2R var_backbone_range 1 2
#=GF R2R bulge a
#=GF R2R bulge_flip b
//
```

C demo-pknot-inline

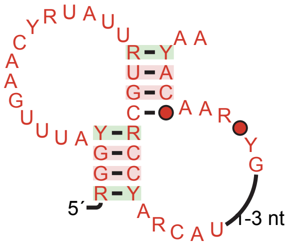

D

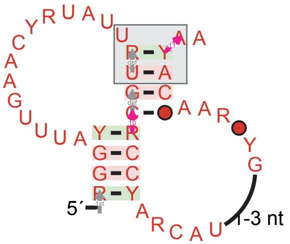

E

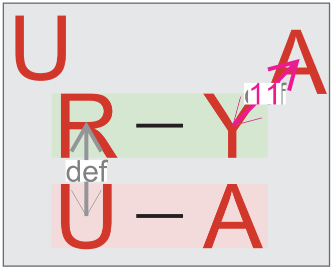

Supplement: btaa080_Supplementary_Data [file btaa080_supplementary_data.zip › btaa080-Suppl_Data/src/rscape_v1.2.3.tar.gz!/rscape_v1.2.3/lib/R2R/R2R-current/R2R-manual/figures/demo-pknot-inline.pdf]
