# Supplementary material for: Estimating the power of sequence covariation for detecting conserved RNA structure
Source: Bioinformatics. 2020 Feb 7;36(10):3072–6. doi: 10.1093/bioinformatics/btaa080 (PMC7214042; doi:10.1093/bioinformatics/btaa080)
Supplement: btaa080_Supplementary_Data [file btaa080_supplementary_data.zip › btaa080-Suppl_Data/src/rscape_v1.2.3.tar.gz!/rscape_v1.2.3/lib/R2R/R2R-current/R2R-manual/figures/demo1.pdf]

A

```
# STOCKHOLM 1.0
human          ACACGCGAAA.GCGCAA.CAAACGUGCACGG
chimp          GAAUGUGAAAAACACCA.CUCUUGAGGACCU
bigfoot        UUGAG.UUCG..CUCGUUUUCUCGAGUACAC
#=GC SS_cons ...<<<.....>>>....<<....>>.....
//
```

B

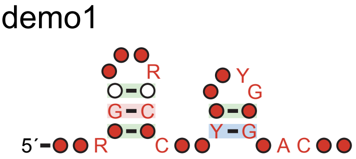

C

```
# STOCKHOLM 1.0
human          ACACGCGAAA.GCGCAA.CAAACGUGCACGG
chimp          GAAUGUGAAAAACACCA.CUCUUGAGGACCU
bigfoot        UUGAG.UUCG..CUCGUUUUCUCGAGUACAC
#=GC SS_cons ...<<<.....>>>....<<....>>.....
//
```

All stockhom files begin with "# STOCKHOLM 1.0"

A "hit id", which identifies this sequence by the name "chimp".

A gap within this sequence.

This identifies the line as being the secondary structure consensus.

pair

pair

pair

pair

pair

An unpaired column

All stockhom files end with "//".

This column is not represented in the drawing in part B, because it is mostly gaps.

D

```
# STOCKHOLM 1.0
human              ACACGCGAAA.GCGCAA.CAAACGUGCACGG
chimp              GAAUGUGAAAAACACCA.CUCUUGAGGACCU
bigfoot            UUGAG.UUCG..CUCGUUUUCUCGAGUACAC
#=GC SS_cons       ...<<<.....>>>....<<....>>.....
#=GC cons          nnRnGnnnnR-nCnCnn-YnnnYGnGnACnn
#=GC conss         1111141111041111101111111111111
#=GC cov_SS_cons   ...202.....202....12....21.....
//
```

The consensus line inferred by R2R. 'n' corresponds to a circle in the diagrams. '-' is a gap. The numbers below define the degree of conservation (colors in the diagrams).

This column is classified as a gap by R2R.

Covariation

No mutation observed

Compatible mutation

E

```
intermediate/demo1.cons.sto
```
