# Supplementary material for: Estimating the power of sequence covariation for detecting conserved RNA structure
Source: Bioinformatics. 2020 Feb 7;36(10):3072–6. doi: 10.1093/bioinformatics/btaa080 (PMC7214042; doi:10.1093/bioinformatics/btaa080)
Supplement: btaa080_Supplementary_Data [file btaa080_supplementary_data.zip › btaa080-Suppl_Data/src/rscape_v1.2.3.tar.gz!/rscape_v1.2.3/lib/R2R/R2R-current/R2R-manual/figures/manA-cover.pdf]

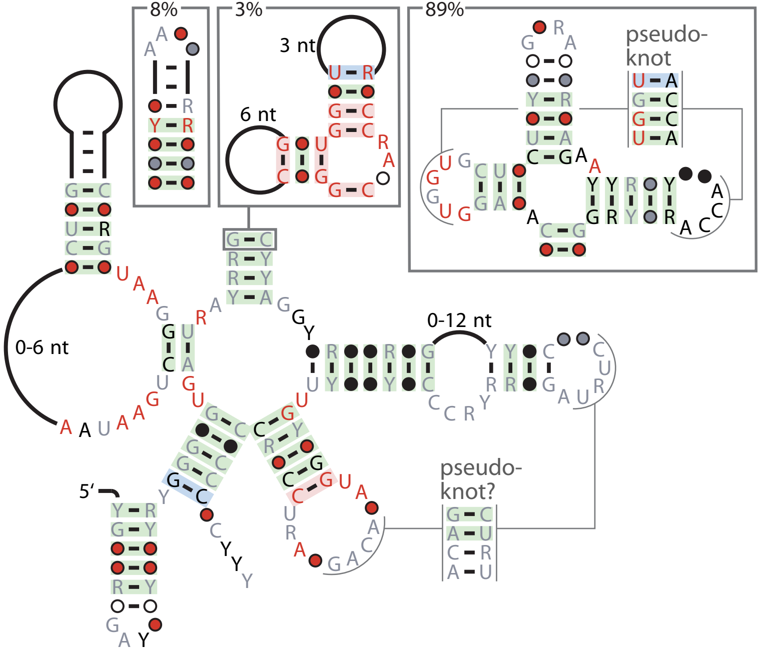
8%
3%
89%
3 nt
6 nt
pseudo-
knot
0-6 nt
0-12 nt
5′
pseudo-
knot?
