# Supplementary material for: Estimating the power of sequence covariation for detecting conserved RNA structure
Source: Bioinformatics. 2020 Feb 7;36(10):3072–6. doi: 10.1093/bioinformatics/btaa080 (PMC7214042; doi:10.1093/bioinformatics/btaa080)

A

```
# STOCKHOLM 1.0
one                          GAGAAA.UCAACCCUUGGGGGAGCA
two                          AGUUCG.CUAAUCCUAGGAGGAGCA
three                        GGGAAACCCAA........GGAGCA
four                         AGCAAC.CUAA........GGAGCA
#=GC SS_cons                 <<.....>>..<<....>>......
#=GC R2R_LABEL               .........................
#=GC SUBFAM_LABEL_TERM       ..xxxxx..................
#=GC SUBFAM_LABEL_OPT        .............x...........
#=GC SUBFAM_GNRA_R2R_LABEL   -.......-----------------
#=GC SUBFAM_UNCG_R2R_LABEL   -.......-----------------
#=GC SUBFAM_OPT_R2R_LABEL    -----------.......------

#=GF SUBFAM_REGEX_PRED HAS_GNRA TERM G[A-Z][AG]A
#=GF SUBFAM_REGEX_PRED HAS_UNCG TERM U[A-Z]CG
#=GF SUBFAM_REGEX_PRED FOUR TERM ^[.]*[A-Z][A-Z][A-Z][A-Z][.]*$
#=GF SUBFAM_PERL_PRED GNRA return $predValue{HAS_GNRA} && $predValue{FOUR};
#=GF SUBFAM_PERL_PRED UNCG return $predValue{HAS_UNCG} && $predValue{FOUR};
#=GF SUBFAM_REGEX_PRED OPT OPT [A-Z]

#=GF SUBFAM_GNRA_R2R no5
#=GF SUBFAM_GNRA_R2R set_dir pos0 -90
#=GF SUBFAM_UNCG_R2R no5
#=GF SUBFAM_UNCG_R2R set_dir pos0 -90
#=GF SUBFAM_OPT_R2R no5
#=GF SUBFAM_OPT_R2R set_dir pos0 -90
//
```

B

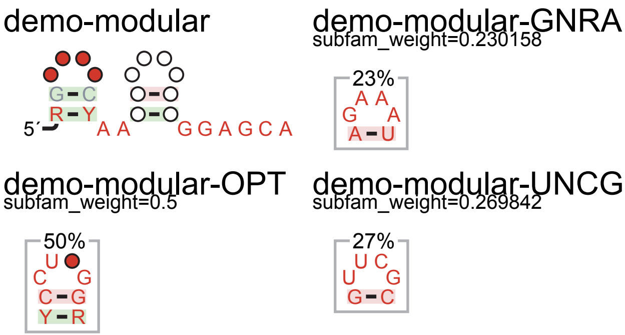

C

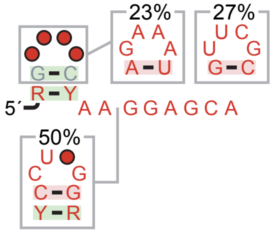

Supplement: btaa080_Supplementary_Data [file btaa080_supplementary_data.zip › btaa080-Suppl_Data/src/rscape_v1.2.3.tar.gz!/rscape_v1.2.3/lib/R2R/R2R-current/R2R-manual/figures/modular-demo.pdf]
