# Supplementary material for: Estimating the power of sequence covariation for detecting conserved RNA structure
Source: Bioinformatics. 2020 Feb 7;36(10):3072–6. doi: 10.1093/bioinformatics/btaa080 (PMC7214042; doi:10.1093/bioinformatics/btaa080)
Supplement: btaa080_Supplementary_Data [file btaa080_supplementary_data.zip › btaa080-Suppl_Data/src/rscape_v1.2.3.tar.gz!/rscape_v1.2.3/lib/R2R/R2R-current/R2R-manual/figures/multistem-solver-HEARO2.pdf]

A

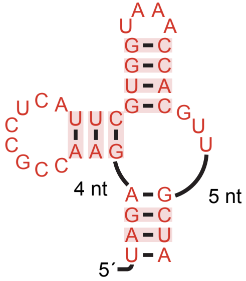


```
multistem_junction_circular_solver
 J s0 0 ai s1 -90 ai s2 0 ai
 align_stem_horiz 0 2
```

B

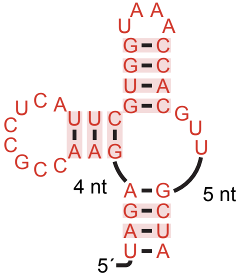


```
multistem_junction_circular_solver
 J s0 0 ai s1 -90 ai=0 s2 0 ai=1
 align_stem_horiz 0 2
```
