# Supplementary material for: Estimating the power of sequence covariation for detecting conserved RNA structure
Source: Bioinformatics. 2020 Feb 7;36(10):3072–6. doi: 10.1093/bioinformatics/btaa080 (PMC7214042; doi:10.1093/bioinformatics/btaa080)
Supplement: btaa080_Supplementary_Data [file btaa080_supplementary_data.zip › btaa080-Suppl_Data/src/rscape_v1.2.3.tar.gz!/rscape_v1.2.3/lib/R2R/R2R-current/R2R-manual/figures/multistem-solver-IMES-1.pdf]

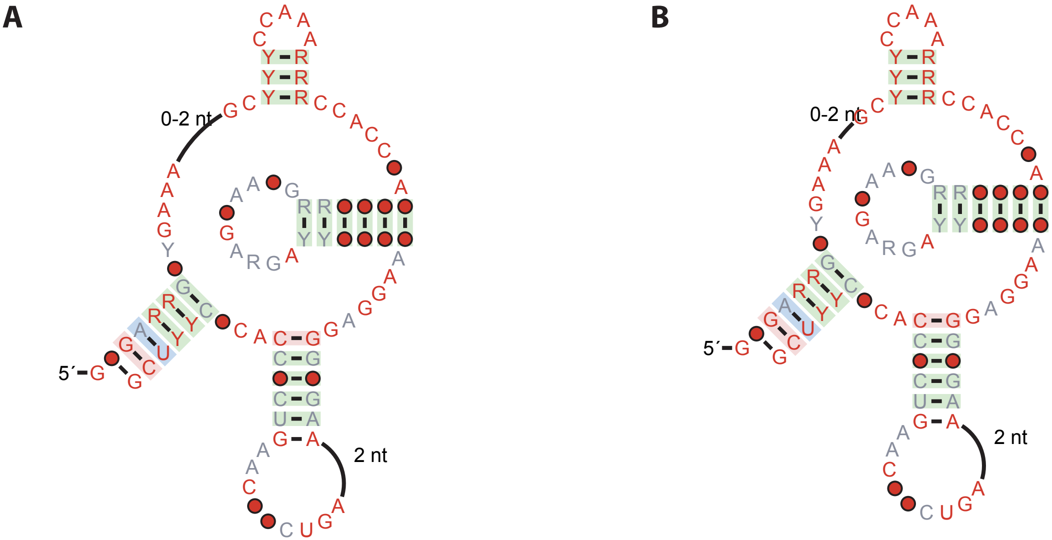


```
multistem_junction_circular_solver
 j s0 0 m s1 -45 m s2 +45 m
 s3 +135 m flipstem 2
```

```
multistem_junction_bulgecircleynormals_solver
 j s0 0 m s1 -45 m s2 +45 m
s3 +135 m flipstem 2
```
