# Supplementary material for: Estimating the power of sequence covariation for detecting conserved RNA structure
Source: Bioinformatics. 2020 Feb 7;36(10):3072–6. doi: 10.1093/bioinformatics/btaa080 (PMC7214042; doi:10.1093/bioinformatics/btaa080)

A

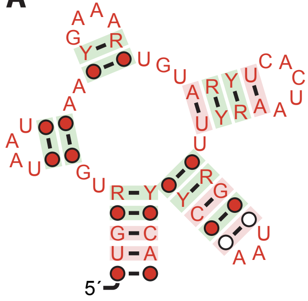

B

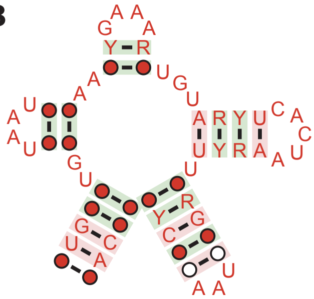

```
multistem_junction_bulgecircley_solver
  j s0 -15 ai s1 -135 ai s2 -45 ai
  s3 45 ai s4 105 ai
```

C

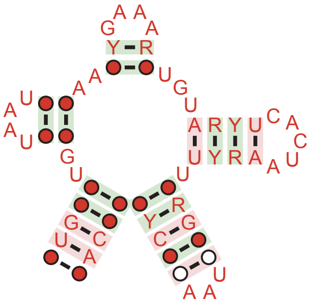

```
multistem_junction_bulgecircley_solver
  j s0 -15 ai s1 -135 ai s2 -45 ai s3 45 ai
  s4 105 ai align_nuc_centers_angle 60 align:X .
  align:Y . align_angle -30 0 4
```

D

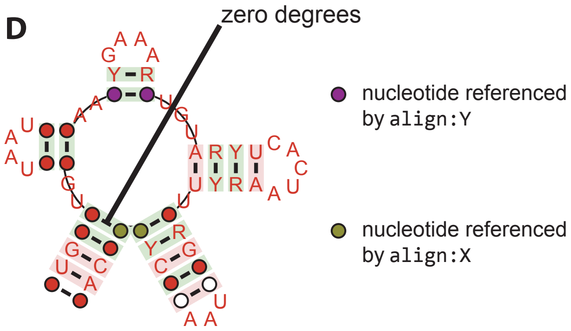

Supplement: btaa080_Supplementary_Data [file btaa080_supplementary_data.zip › btaa080-Suppl_Data/src/rscape_v1.2.3.tar.gz!/rscape_v1.2.3/lib/R2R/R2R-current/R2R-manual/figures/multistem-solver-manA.pdf]
