# Supplementary material for: Estimating the power of sequence covariation for detecting conserved RNA structure
Source: Bioinformatics. 2020 Feb 7;36(10):3072–6. doi: 10.1093/bioinformatics/btaa080 (PMC7214042; doi:10.1093/bioinformatics/btaa080)
Supplement: btaa080_Supplementary_Data [file btaa080_supplementary_data.zip › btaa080-Suppl_Data/src/rscape_v1.2.3.tar.gz!/rscape_v1.2.3/lib/R2R/R2R-current/R2R-manual/figures/multistem-solver.pdf]

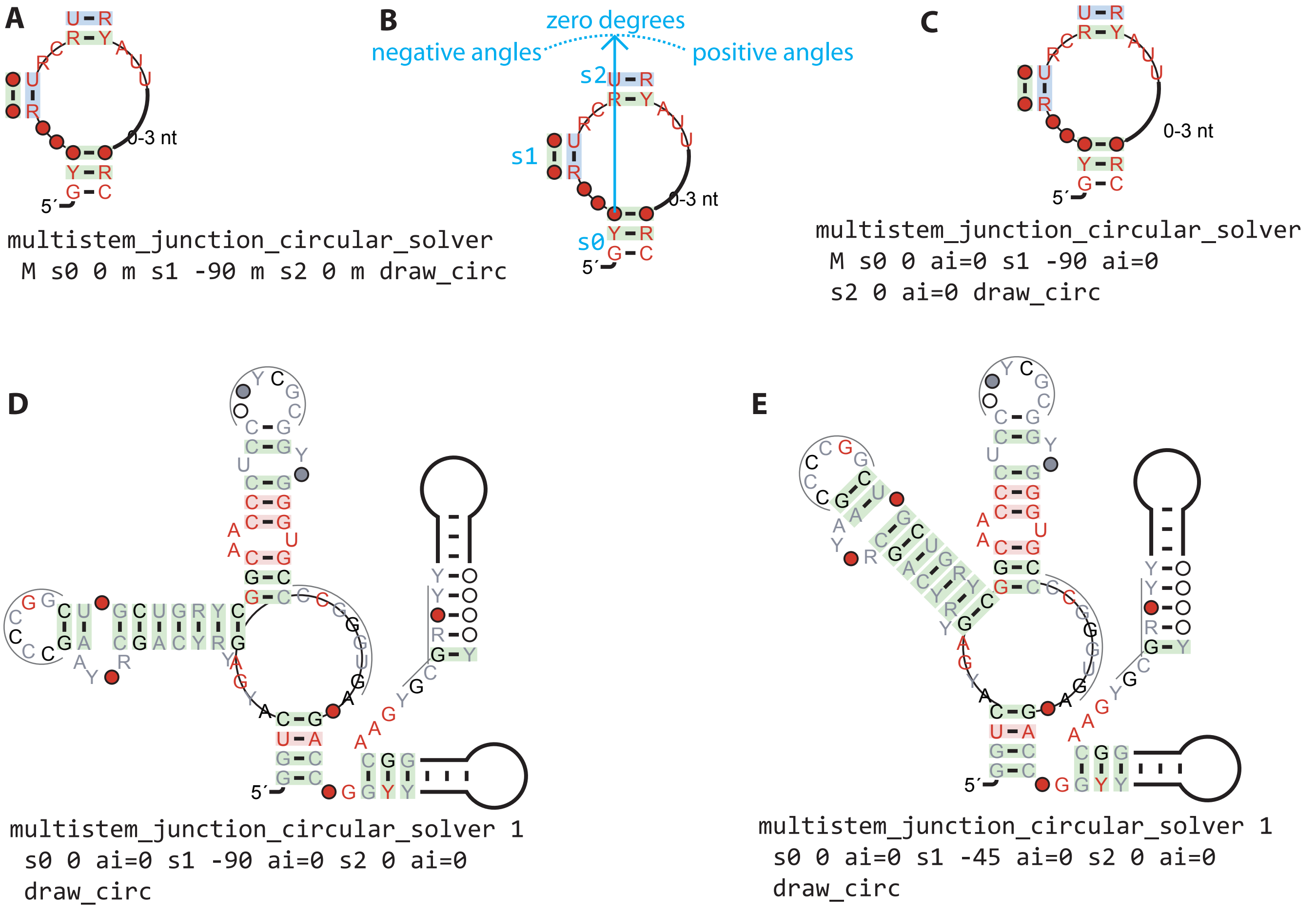
A
0-3 nt
5´
multistem_junction_circular_solver
M s0 0 m s1 -90 m s2 0 m draw_circ
B
zero degrees
negative angles
positive angles
s2
s1
s0
0-3 nt
5´
C
0-3 nt
5´
multistem_junction_circular_solver
M s0 0 ai=0 s1 -90 ai=0
s2 0 ai=0 draw_circ
D
5´
multistem_junction_circular_solver 1
s0 0 ai=0 s1 -90 ai=0 s2 0 ai=0
draw_circ
E
5´
multistem_junction_circular_solver 1
s0 0 ai=0 s1 -45 ai=0 s2 0 ai=0
draw_circ
