# Supplementary material for: Estimating the power of sequence covariation for detecting conserved RNA structure
Source: Bioinformatics. 2020 Feb 7;36(10):3072–6. doi: 10.1093/bioinformatics/btaa080 (PMC7214042; doi:10.1093/bioinformatics/btaa080)

A

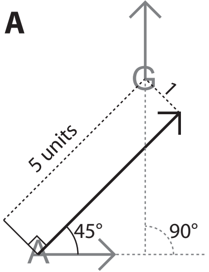

B

```
# STOCKHOLM 1.0
biologist        AACCCUUCGGAAACGGAGUAA
chemist          AACCCUUGCUUCGGCGAGUAA
physicist        AUCCCAUUUCUUGAAGAGAAA
#=GC SS_cons     <<...>><<....>>......
#=GC R2R_LABEL   .......a.......b..c..
#=GF R2R place_explicit a a-- 0 1 0 0 0 0 f
#=GF R2R place_explicit b b-- +45 1 0 0 0 +90 f
#=GF R2R place_explicit c c-- -45 1 0 0 0 -90
//
```

C

demo-pe

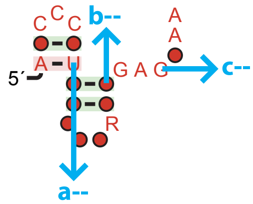

D mini-ykkC

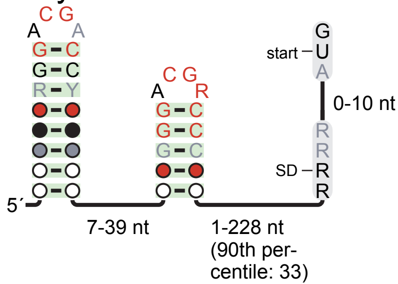

Supplement: btaa080_Supplementary_Data [file btaa080_supplementary_data.zip › btaa080-Suppl_Data/src/rscape_v1.2.3.tar.gz!/rscape_v1.2.3/lib/R2R/R2R-current/R2R-manual/figures/place_explicit.pdf]
