# Supplementary material for: Estimating the power of sequence covariation for detecting conserved RNA structure
Source: Bioinformatics. 2020 Feb 7;36(10):3072–6. doi: 10.1093/bioinformatics/btaa080 (PMC7214042; doi:10.1093/bioinformatics/btaa080)
Supplement: btaa080_Supplementary_Data [file btaa080_supplementary_data.zip › btaa080-Suppl_Data/src/rscape_v1.2.3.tar.gz!/rscape_v1.2.3/lib/R2R/R2R-current/R2R-manual/figures/turnsteminternal.pdf]

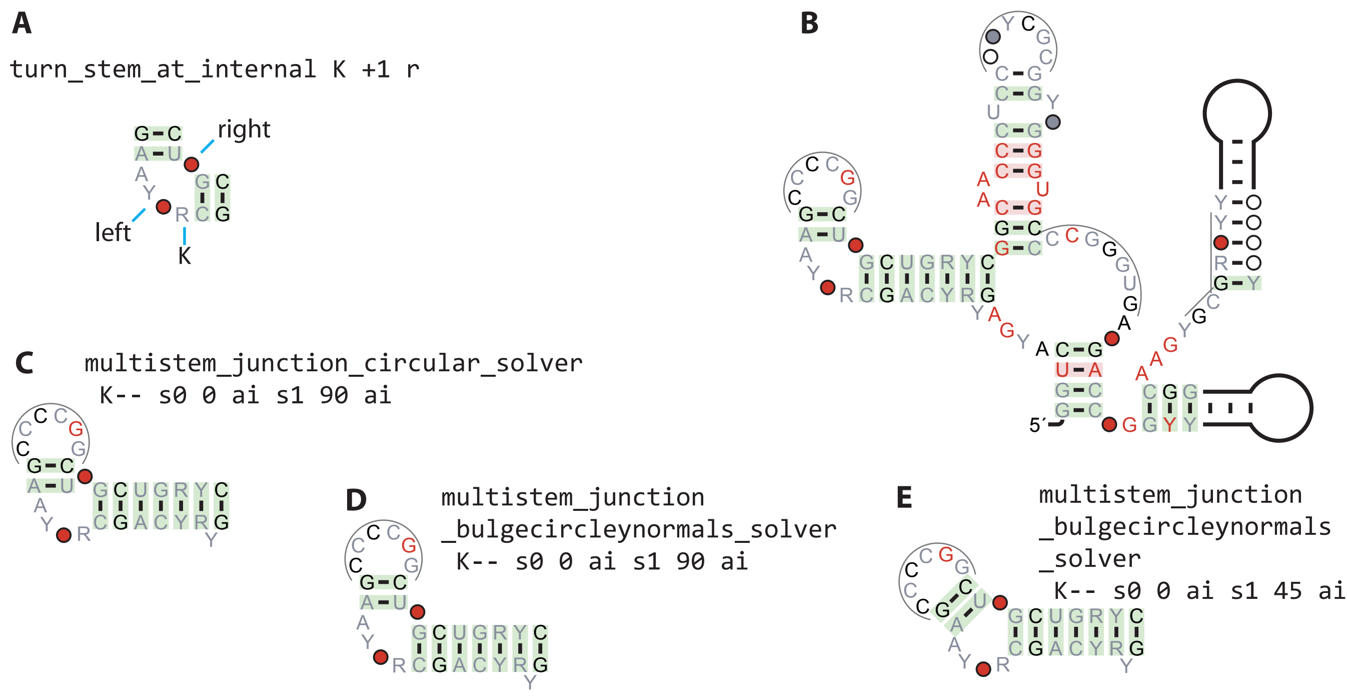
A
turn_stem_at_internal K +1 r
right
left
K
B
5′
C
multistem_junction_circular_solver
K-- s0 0 ai s1 90 ai
D
multistem_junction
_bulgecircleynormals_solver
K-- s0 0 ai s1 90 ai
E
multistem_junction
_bulgecircleynormals
_solver
K-- s0 0 ai s1 45 ai
